# A genomic survey of the fish parasite *Spironucleus salmonicida *indicates genomic plasticity among diplomonads and significant lateral gene transfer in eukaryote genome evolution

**DOI:** 10.1186/1471-2164-8-51

**Published:** 2007-02-14

**Authors:** Jan O Andersson, Åsa M Sjögren, David S Horner, Colleen A Murphy, Patricia L Dyal, Staffan G Svärd, John M Logsdon, Mark A Ragan, Robert P Hirt, Andrew J Roger

**Affiliations:** 1 Institute of Cell and Molecular Biology, Uppsala University, Biomedical Center, Uppsala, Sweden; 2 The Canadian Institute for Advanced Research, Program in Evolutionary Biology, Department of Biochemistry and Molecular Biology, Dalhousie University, Halifax, Nova Scotia, Canada; 3 Department of Zoology, The Natural History Museum, London, UK; 4 Institute for Marine Biosciences, National Research Council of Canada, Halifax, Nova Scotia, Canada; 5 Roy J. Carver Center for Comparative Genomics, Department of Biological Sciences, University of Iowa, Iowa City, USA; 6 Department of Microbiology, Swedish University of Agricultural Sciences, Uppsala, Sweden; 7 Dipartimento di Scienze Biomolecolare e Biotecnologie, University of Milan, Milan, Italy; 8 ARC Centre in Bioinformatics, and Institute for Molecular Bioscience, The University of Queensland, Brisbane, Australia; 9 School of Biology, The Devonshire building, The University of Newcastle upon Tyne, UK

## Abstract

**Background:**

Comparative genomic studies of the mitochondrion-lacking protist group Diplomonadida (diplomonads) has been lacking, although *Giardia lamblia *has been intensively studied. We have performed a sequence survey project resulting in 2341 expressed sequence tags (EST) corresponding to 853 unique clones, 5275 genome survey sequences (GSS), and eleven finished contigs from the diplomonad fish parasite *Spironucleus salmonicida *(previously described as *S. barkhanus*).

**Results:**

The analyses revealed a compact genome with few, if any, introns and very short 3' untranslated regions. Strikingly different patterns of codon usage were observed in genes corresponding to frequently sampled ESTs versus genes poorly sampled, indicating that translational selection is influencing the codon usage of highly expressed genes. Rigorous phylogenomic analyses identified 84 genes – mostly encoding metabolic proteins – that have been acquired by diplomonads or their relatively close ancestors via lateral gene transfer (LGT). Although most acquisitions were from prokaryotes, more than a dozen represent likely transfers of genes between eukaryotic lineages. Many genes that provide novel insights into the genetic basis of the biology and pathogenicity of this parasitic protist were identified including 149 that putatively encode variant-surface cysteine-rich proteins which are candidate virulence factors. A number of genomic properties that distinguish *S. salmonicida *from its human parasitic relative *G. lamblia *were identified such as nineteen putative lineage-specific gene acquisitions, distinct mutational biases and codon usage and distinct polyadenylation signals.

**Conclusion:**

Our results highlight the power of comparative genomic studies to yield insights into the biology of parasitic protists and the evolution of their genomes, and suggest that genetic exchange between distantly-related protist lineages may be occurring at an appreciable rate in eukaryote genome evolution.

## Background

Diplomonads are a diverse group of small mitochondrion-lacking diplokaryotic flagellates found in anaerobic or micro-aerophilic environments [1]. Most research on diplomonads has focused on *Giardia lamblia *(syn. *Giardia intestinalis*, *Giardia duodenalis*), which is a major cause of water-borne enteric disease in humans in both industrialised and developing countries [2]. However, there are important variations in lifestyles among diplomonads; although many are endocommensals or parasites associated with animals, there are also several free-living species, mainly within the genera *Trepomonas *and *Hexamita*, that are found in aquatic environments rich in organic matter and deficient in oxygen [1]. Most members of the genus *Spironucleus *are parasites, typically of fish but also of birds and mice; several *Spironucleus *species have been shown to cause disease in their hosts [1], although essentially nothing is known about the virulence mechanisms of *Spironucleus *species. *Spironucleus salmonicida*, the focus of this study, can cause systemic and organ infections in cultivated salmon, posing a significant problem for the aquaculture industry [3,4]. This isolate was previously known as *Spironucleus barkhanus *[5], but pathogenic isolates of this species were recently redescribed as *S. salmonicida *to distinguish them from morphologically identical, but genetically distinct, fish commensal isolates of *S. barkhanus *[6].

Diplomonads were once thought to belong to the earliest-diverging lineage within the eukaryotes [7]. Accordingly, they were described as 'biological fossils', true eukaryotes with many peculiarities (e.g. two nuclei, different genetic code, lack of aerobic mitochondria) that retained some ancestral prokaryotic properties [8,9]. However, advances in molecular phylogenetics and cell biology during the last decade strongly suggest this view is incorrect [10]. The current interpretation of the phylogeny of eukaryotes lends no support for diplomonads as the earliest eukaryotic branch [11,12]. Indeed, a sister-group relationship between diplomonads and parabasalids to the exclusion of other eukaryotic lineages and the root has recently been demonstrated, based both on phylogenetic analysis of concatenated protein-coding sequences [13-15], and on shared gene acquisitions [16,17]. Diplomonads and parabasalids are now classified within the eukaryotic supergroup Excavata [18]. Furthermore, diplomonads seem to have all features previously thought to be lacking in "primitive" eukaryotes, including an organelle with mitochondrial ancestry (mitosome) [19] and intron-containing genes [20,21], while some prokaryotic properties are probably the result of lateral gene transfers from the prokaryotic realm [22].

Recently, enteromonads (monokaryotic protists traditionally regarded as closely related to diplomonads) were surprisingly found to branch robustly with *Trepomonas *and *Hexamita *in phylogenetic analyses to the exclusion of *Spironucleus *[23]. These results suggest that the diplokaryotic state of diplomonads arose multiple times independently, or that the monokaryon of enteromonads is a derived feature. Large genetic and biological variation also exists within diplomonads. For example, *Spironucleus*, *Trepomonas *and *Hexamita*, form a monophyletic clade to the exclusion of *Giardia *in phylogenetic trees [24], and use an alternative genetic code whereby TAA and TAG, rather than being stop codons, encode glutamine [25,26].

To gain insights into the evolutionary history and genomic architecture of diplomonads in general and *Spironucleus *in particular, we initiated a genome survey project in *S. salmonicida*. To maximize gene discovery we obtained expressed sequence tag (EST) and genomic survey sequences (GSS), and completely sequenced eleven contigs. Here, we present analyses of the complete set of sequences obtained in the genome survey project. Although we present data that only partially covers the *S. salmonicida *genome, these data provide key insights into the genome-level properties of *S. salmonicida *such as its coding content, base compositional biases, gene content, gene architecture, and patterns of gene acquisitions. From these analyses, we are able to make inferences about the biology of a poorly-understood fish parasite, as well as gain a first glimpse into genome evolution within the enigmatic protist phylum Diplomonadida.

## Results and discussion

### The first sequence data from *Spironucleus *on the genomic level

This sequence survey of *S. salmonicida *has been the starting point for several projects regarding different aspects of the genetics and molecular evolution of this diplomonad [16,27-33]. Together with a few other studies, these have resulted in about thirty *S. salmonicida *genes in the public databases. The present analysis of the complete set of 2408 EST and 5275 GSS sequences, combined with a the complete sequence of eleven contigs corresponding to 80 kbp unique sequence (Additional file 1), extend this information to include 1738 unique protein coding genes, two ribosomal RNA genes and 20 tRNA genes (Table 1 and Additional file 2). Altogether our sequence data cover more than 2.5 Mbp of the *S. salmonicida *genome. The only genome size reported from a diplomonad is 12 Mbp for the genome of *G. lamblia *[34]. The genome size of *S. salmonicida *is unknown, but the degree of sequence overlap between independent clones in our random genomic shotgun library yielded a very rough estimate of approximately 7 Mbp of unique sequence (see Methods for details of the calculation). Interestingly, the observation that 33% of the EST sequences have matches within the GSS sequences suggests a genome size in a similar range, unless the same genes are preferentially cloned in both the EST and GSS sequences. Thus, there are no indications within our sequence survey project that the *S. salmonicida *genome is larger than the *G. lamblia *genome. These observations indicate that our obtained sequences potentially represent one third of the complete genome.

**Table 1:** Summary of genes detected in the *S. salmonicida *genome.

	EST	GSS	contigs	published	TOTAL
Cellular processes	26	46	1	1	74
Cell communication	6	1			7
Cell growth and death	6	14			20
Cell motility	1	6			7
Development		1			1
unclassified	13	24	1	1	39
Environmental information processing	34	53	3		92
Ligand-receptor interaction	2	2			4
Membrane transport	1	5	2		8
Signal transduction	28	44	1		73
unclassified	3	4			7
Genetic information processing	126	58		5	189
Folding, sorting and degradation	30	16		2	47
Replication and repair	9	13			22
Transcription		11			11
Translation	82	13		1	96
unclassified	5	5		2	12
Metabolism	99	110	6	21	236
Amino acid metabolism	17	16	1	4	38
Carbohydrate metabolism	28	29	1	9	67
Energy metabolism	18	12		5	35
Glycan biosynthesis and metabolism		4	1		5
Lipid metabolism	5	8		1	14
Metabolism of cofactors and vitamins	6		1		7
Metabolism of other amino acids	1	1			2
Nucleotide metabolism	19	38	2	1	60
unclassified	4	2		1	7
Conserved hypothetical protein	189	528	15		732
Hypothetical protein			13		13
Ribosomal RNA			2		2
tRNA		20			20

Total	473	817	40	27	1357

### Variable G+C content along the genome

**Figure 1:**
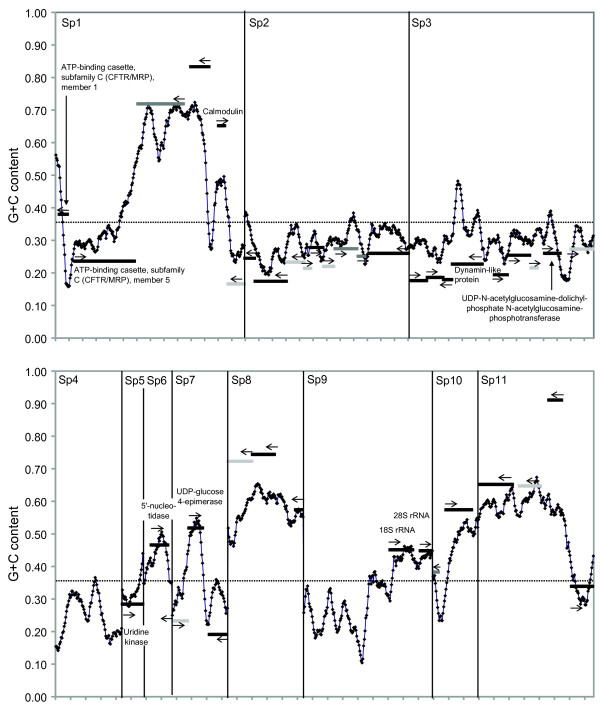
**A plot of G+C content along the eleven finished contigs**. G+C content calculated in 500-bp sliding windows in 50-bp steps along the finished contigs. The marks on the x axis indicate 1 kbp. Black lines, dark grey and light grey lines indicate annotated genes with sequence similarities in the public databases, hypothetical genes with matches in the EST data, and hypothetical genes without matches in the public databases or EST data, respectively. The position of the line on the y axis indicates the GC3_s _value of the gene, an arrow indicates its direction (strand), and the annotated gene function is indicated. Average genomic G+C contents in the GSS clones are indicated by a dotted line. The order of the contigs is arbitrary.

On the whole, the *S. salmonicida *genome was found to be G+C poor; the average G+C contents were 35.9%, and 39.3% within the 5070 GSSs and the eleven finished sequences, respectively. However, the eleven finished contigs showed large variation in G+C content, with average G+C contents ranging from 24.9% to 58.3% (named Sp1-11, Figure 1). This probably cannot be explained by the presence within each contig of genes with the higher values; the coding content of Sp2 is 96% and its G+C content is only 31%, while the coding content of Sp8 is 75% and its G+C content is 58% (Additional file 1). In fact, the G+C content sometimes varies drastically within each contig. For example, the average G+C content in the region between 1–5 kbp from the 5' end of Sp1 is around 30%, while between 6–11 kbp its G+C content is almost 70% (Figure 1). Similar sharp shifts in G+C content along the genome are observed in some other contigs (e.g. Sp7, Sp10, and Sp11: Figure 1). These shifts in G+C content do not seem to be correlated with the presence and absence of genes: both G+C rich and poor regions are fairly dense with genes, with the exception of the G+C poor contig Sp4 and the 5' end of Sp9. However, these observations are consistent with the existence of different mutational biases in different regions of the *S. salmonicida *genome; the G+C contents of both the third synonymous position of protein coding genes and the non-coding regions varies as expected if mutational bias shapes the G+C content [35], with the GC3 values of genes higher than the average G+C content for genes in G+C rich regions and lower in G+C poor regions (Figure 1). A similar pattern is observed for non-coding regions (Additional file 1), and the codon usage analysis indicates large variations of the G+C contents among genes (see below).

### *S. salmonicida *has a compact genome

Analysis of the eleven completely sequenced contigs showed that the coding density varies significantly between contigs (Figure 1 and Additional file 1). Some parts of the genome appear to be very gene-dense, with short intergenic regions. In fact, six cases of putative overlapping open reading frames (ORFs) were identified, similar to earlier findings in *G. lamblia *[36]. The lengths of the overlapping regions range from 1 to 101 bp; in four cases they are encoded in the same direction, while in the other two cases the 5' regions overlap. Experimental studies are needed to determine which initiation codons actually are used in these putative genes to determine if they are truly overlapping transcribed ORFs that encode functional proteins. Nevertheless, the whole genome does not seem to be packed with genes; no protein coding sequence could be detected in the almost 5-kbp contig Sp4, nor in a long region upstream of the 18S ribosomal RNA gene in Sp9 (Figure 1). In *G. lamblia*, the genes for ribosomal RNA have been found to be associated with telomeres [37]. However, we were unable to detect any sequence similarity to genes that are associated with telomeres in *G. lamblia*, or any telomere repeats, in the long region upstream of the rRNA genes in *S. salmonicida *Sp9.

**Figure 2:**
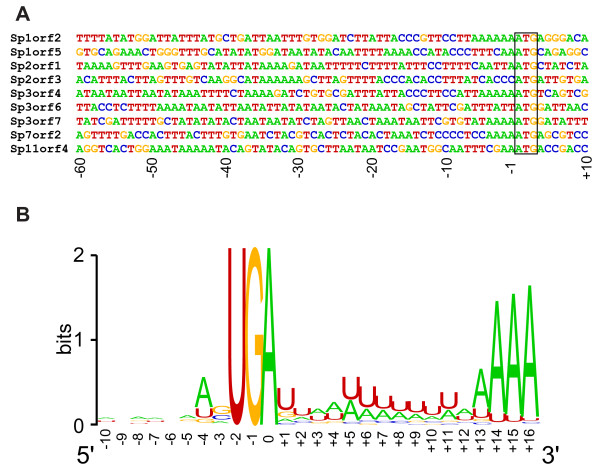
**5' and 3' untranslated regions in *S. salmonicida***. A: 5' regions of full-length genes from the completely sequenced contigs. The initiation codons of nine genes in the contigs (Figure 1) could be precisely identified based on sequence conservation compared to orthologs. The 5' sequences are aligned based on the initiation codons (indicated by a box). B: Sequence logo [107] around the termination codon of 134 *Spironucleus *cDNA sequences.

The transcription of protein-encoding genes in *Giardia *is atypical for eukaryotes. The promoters are short; less than 70 bp is needed for efficient expression of most genes, even if they are stage-specific [38]. The 5'-UTRs of the transcripts are usually only 1–10 nt, and the genes lack TATA boxes or other *cis*-acting promoter elements characteristic of typical eukaryotic promoters; AT-rich sequences around the *G. lamblia *ATG initiation codons determine the transcriptional start site and are essential and sufficient for promoter activity [39]. The *S. salmonicida *promoters apparently lack TATA-boxes and other typical eukaryotic promoter motifs (Figure 2A). However, AT-rich stretches are found close to the translational start sites, or in the first 50 bp upstream (Figure 2A) and the intergenic regions are very short (Figure 1). Thus, the *S. salmonicida *transcriptional machinery appears to resemble the 'atypical' machinery previously characterized in *G. lamblia *[39].

The position of the polyA tail in relation to the termination codon could be mapped in 134 EST clones. The 3' untranslated regions (UTR) of *S. salmonicida *genes appear to be short; the average distance between the termination codon and the first A in the polyA tail is 13.2 bases and in 122 of the 134 cases the distance is between 9 and 14 nucleotides (Figure 2B). Such very short 3' UTRs have previously been observed in *G. lamblia*, but this is in stark contrast to most other studied eukaryotes where 3' UTRs have been proposed to play important roles in regulating messenger RNA (mRNA) stability and translation [40]. In *G. lamblia*, AGTRAAY has been identified as a consensus polyadenylation signal [41]. Our analyses do not identify any strong consensus sequence outside the conserved unique termination codon TGA, although we observe a preference for A in the position two bases upstream of the termination codon, and a T immediately downstream (Figure 2B). Interestingly, the three most conserved nucleotides in the *G. lamblia *polyadenylation signals are the central TRA nucleotides, which is similar to the situation in *S. salmonicida*. Furthermore, a preference for a U-rich region close to the polyA tail is also apparent, a pattern conserved in many eukaryotes but not *Giardia *[41,42]. It has been reported that a fraction (~20%) of complementary DNAs (cDNAs) from *G. lamblia *represent polyadenylated sterile anti-sense transcripts that may result from a loose control of transcription [43]. The rarity of such sterile transcripts in our cDNA library (~1% among 2341 EST) indicates that this phenomenon is not universal for diplomonads. Indeed, our data indicate considerable variation in the specificity of the transcription and polyadenylation machineries within diplomonads.

The availability of both EST and genomic sequences was used to identify putative introns. An intron should be detected as a sequence gap in an alignment between the EST and the corresponding genomic sequence because the EST sequences typically represent the spliced version of the gene sequence. This approach was used to identify potential introns using similarity searches with the EST sequences as probes against the GSS and finished contig genomic sequences. 290 out of 885 ESTs matched with an e < e^-10 ^(*i.e.*, almost all were identical sequences) and manual inspection failed to detect any introns. The *S. salmonicida *genome appears to be very intron-poor. Given that only a few introns have been detected within the diplomonad *G. lamblia *[20,21], a paucity of introns could represent the ancestral state of the diplomonads. However, two introns were detected in one of the first genes sequenced from *Carpediemonas *[44], an excavate taxon and possible sister lineage to diplomonads. Thus, introns may have been more frequent in a common ancestor of diplomonads and *Carpediemonas*, and that the ancestral diplomonad lineage experienced genome-wide intron loss, an evolutionary phenomenon that is not uncommon [45]. A basic spliceosomal organisation is present in *Giardia *suggesting that the spliceosome is ancestral to extant eukaryotes [46]. Although our analyses did not identify any proteins likely to be involved in splicing in *S. salmonicida*, further sequencing and more detailed analyses are needed to determine whether introns and a functional spliceosomal apparatus are present in *S. salmonicida*.

### Coding capacity of *S. salmonicida*

In this study we have identified more than 1300 novel genes, distributed among all functional categories, representing a twenty-fold increase of the number of genes with assigned functions identified in the *S. salmonicida *genome (Table 1). Among these were genes involved in translation: 74 genes encoding ribosomal proteins, many translation factors, and fifteen different tRNA synthetases were identified (Additional file 2). Twenty tRNA genes were identified, which cover 24 of the 63 sense codons allowing for the normal wobble rules for codon-anticodon pairing. No tRNA was identified that decodes UAG or UAA codons, which encode glutamine in *Spironucleus*, although such tRNA genes have been identified previously [25]. Interestingly, all 64 codons appear to have the potential to code for incorporation of amino acids into proteins in *S. salmonicida*; both tRNA identification programs used in our analyses identified a putative tRNA with the anticodon UCA, annotated as a selenocysteine (Sec) tRNA. The identification of a putative Sec tRNA, which is a central component of selenoprotein biosynthesis [47], in our data set suggests that *Spironucleus *is able to use the single stop codon (UGA) to incorporate this rare amino acid into selenoproteins. Indeed, the usage of selenocysteine seems to be widespread feature in protists; Sec tRNAs have recently been identified in *Dictyostelium*, *Tetrahymena*, and *Plasmodium *[48,49].

The identified proteins within the cellular, environmental information, and genetic information process categories in our data set clearly reflect a coding potential similar to a typical eukaryote, including eukaryotic translation and transcription machineries, many proteins involved in eukaryotic signal transduction pathways, a large family of dynein proteins, and five genes encoding members of the Rab small GTPase family (Additional file 2). This is indeed expected from the current view of diplomonad phylogeny and cell biology (see Background section). Interestingly, only eight Rab genes were found in *G. lamblia*, while a large number of Rab GTPases were found in *Trichomonas vaginalis*, most likely related to the apparently more-complex endomembrane system in this parabasalid [50]. Two of the five Rab proteins identified in our survey lack identifiable orthologs in the *G. lamblia *genome, but branch with *T. vaginalis *Rab sequences in phylogenetic trees (data not shown), suggesting that *S. salmonicida *has retained Rab proteins that have been lost in the lineage leading to *G. lamblia*.

Absences of genes are very difficult to infer from partial genome data, but some general trends may be observed. For example, relatively few enzymes involved in amino acid metabolism were detected (Additional file 2). Aminoacyl-tRNA synthetases are classified into this category, but they are atypical since they are essential for protein synthesis. Furthermore, only a single protein (malate dehydrogenase) associated with the tricarboxylic acid (TCA) cycle was found. However, malate dehydrogenase actually functions in a pyruvate synthesis pathway in *G. lamblia *[2], rather than in the TCA cycle, suggesting a similar role in *S. salmonicida*. In contrast, several glycolytic proteins are present in our data set (Additional file 2). This pattern of metabolic proteins is expected from a fermentative phagotrophic heterotroph which has access to organic compounds from its host, and indeed is very similar to the pattern found in *Entamoeba histolytica *[51]. These similarities between *S. salmonicida *and *E. histoytica *are almost certainly due to independent adaptations to such an environment in the two lineages. Indeed, a considerable fraction of the metabolic proteins was found to be more closely related to prokaryotic rather than eukaryotic homologs in the phylogenomic analyses, suggesting acquisition of these genes by gene transfer (see further discussion below).

### Does *S. salmonicida *possess mitosomes?

A new organelle, the mitosome, has recently been identified in *G. lamblia*, that is probably a remnant of a mitochondrion [19]. Currently, the only known function of mitosomes in *Giardia *is iron-sulfur (Fe-S) cluster synthesis [52,53]. The phylogenetic relationship between *G. lamblia *and *S. salmonicida*, the likely mitochondrial origin of the organelle, together with mitochondria and their derived organelles currently being thought to be universally present in all extent eukaryotes [10] strongly suggest that the common ancestor of the two species contained an organelle with mitochondrial ancestry. Here we identified two strong cases of candidate mitosomal proteins in *S. salmonicida*, a chaperon GroEL (or Hsp60 or Cpn60) a key protein for mediating protein folding in mitochondria [28] and a cysteine desulfurase (called Nifsp in *Saccharomyces cerevisiae*), a key enzyme of the Fe-S cluster synthesis pathway [54] (Additional file 2). Orthologous proteins of Hsp60 and Nifsp are known to localize and function in *S. cerevisiae *mitochondria and *G. lamblia *mitosomes [52,53]. Localization studies of these two proteins should indeed be very useful to investigate whether *S. salmonicida *contains mitosomes.

A full-length ORF encoding a dynamin-like protein that is found on contig Sp3 (Figure 1) is another protein potentially linked with the mitosome. The *G. lamblia *genome encodes a single dynamin-like protein and phylogenetic analyses recover the two diplomonad sequences as monophyletic (data not shown). The function of the *G. lamblia *single dynamin-like protein is currently unknown. Yet, a single dynamin-like protein encoding gene is also found in three kinetoplastid genomes (two *Trypanosoma *and one *Leishmania*) [55] and the microsporidium *Encephalitozoon cuniculi *[56], whereas most eukaryotic genomes encode several dynamin-like paralogues that function either in membrane trafficking or organelle division (mitochondria and plastids) [57]. Interestingly, the single dynamin-like protein from *Trypanosoma brucei *was shown to be involved in mitochondrial division [58]. Hence the single dynamin-like proteins in *Spironucleus*, *Giardia *and *Encephalitozoon *could all be involved in mitosome division.

### A large family of cysteine-rich proteins

**Figure 3:**
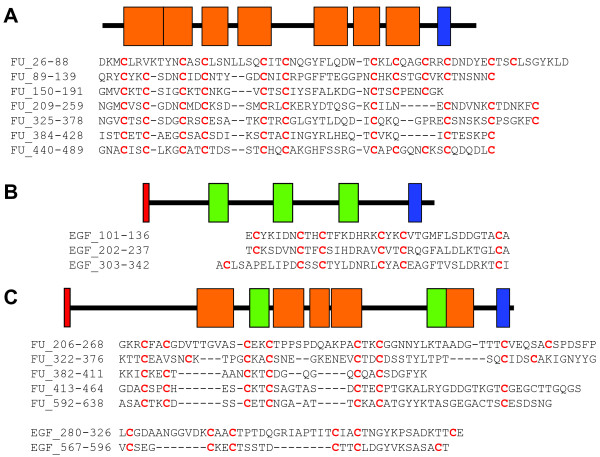
**Structural organization of the two identified full-length candidate surface proteins with cysteine rich segments**. A: Domain organization of the putative protein encoded by Sp10orf2 (573 residues) [GenBank:DQ812527]. The diagram shows the position of the seven furin-like domains (SM00261 – orange boxes) relative the transmembrane domain (blue box) as determined by SMART4.0 [125]. Several variations were inferred by SMART including some where furin-like domains corresponded to the related EGF-like domain (SM00181 – green boxes, see below). Due to the overlap between these inferences only the furin-like domains are shown for simplicity. The orientation of the protein in the membrane is N-terminus outside and C-terminus inside as inferred by TMHMM2.0 [126]. This is consistent with the hypothesis that cysteine-rich domains are facing the extracellular milieu where they could interact with other proteins. The alignment of the seven inferred furin-like domains as inferred by T-COFFEE [127] is shown below the diagram, with the positions of the first and last residue of each domain indicated on the left. Cysteines are highlighted in red and bold. No putative signal peptide was found with SignalP3.0 [128]. B: Domain organization of the putative protein encoded by Sp1orf4 (453 residues) [GenBank:DQ812518]. This protein has a similar structural organization as the one shown in A with three EGF-like domains inferred to face the external milieu. The three EGF-like domains were aligned manually. In addition, this sequence may have a signal peptide (indicated by a red box) since the S-score is positive in SignalP3.0 [128]. C: Domain organization of *G. lamblia *VSP417-6 protein (704 residues) [GenBank:AAF02907] [129] is shown for comparison. Four furin-like and two EGF-like domains identified by SMART4.0 [125] were aligned with T-COFFEE [127]. The *G. lamblia *sequence possesses a signal peptide according to SignalP3.0 [128]. All diagrams are drawn to scale.

The predicted amino acid sequences of 149 genes were found to contain more than 10% cysteine and were classified as cysteine-rich proteins. Most of these were annotated as conserved hypothetical proteins, although some could be assigned to a functional category (Additional file 2). Most of the cysteine residues were found as CXXC motifs (Figure 3 and data not shown). Such an arrangement is similar to the large protein family of variant-specific surface proteins (VSP) found in *G. lamblia *[59], which is estimated to comprise 2.4% of the genome [60]. However, less than 10% of the cysteine-rich proteins in *S. salmonicida *showed highest sequence similarities to *G. lamblia *cysteine-rich proteins, and the two conserved motifs of *G. lamblia *VSP proteins, CRGKA and GGCY [59], were not found within any of the amino acid sequence of *S. salmonicida *cysteine-rich proteins. Indeed, in similarity searches the majority were most similar to putative proteins discovered in the ciliate *Tetrahymena thermophila *genome project (data not shown) [61]. These observations indicate that diplomonads vary greatly in their cysteine-rich proteins; the gene families indeed appear to have expanded independently in the *Giardia *and *Spironucleus *lineages. The biological role of VSP proteins is not well understood, but VSP proteins are immuno-dominant in *G. lamblia *and expressed on the cell surface [59]. Among the 149 cysteine-rich proteins coding sequences two are likely to represent full-length ORFs. These are strong candidate surface proteins since they possess a transmembrane (TM) domain and a cysteine-rich domain made of furin-like and/or epidermal growth factor (EGF)-like domains typical of surface or secreted proteins, a feature shared with *G. lamblia *VSPs (Figure 3). A total of 14 sequences encoding partial proteins with cysteine-rich domains possess similar structural organization as the one shown in Figure 3, i.e. they possess a TM domain with the cysteine-rich domains likely to face the extra-cellular milieu. No TM domains could be found in the remaining cysteine-rich proteins within our dataset. However, in several cases these partial cysteine-rich proteins show high similarity to cysteine-rich proteins containing TM domains, suggesting that they could represent partial surface protein sequences (derived from EST or GSS). This observation is consistent with the genome of *S. salmonicida *encoding a large gene family of cysteine-rich variant surface proteins, as established for the VSP protein family in *G. lamblia *where only one of the 150 VSP genes is expressed per cell. In *G. lamblia *the VSP gene family displays antigenic variation and new VSP genes are induced at a relatively high frequency, which is important for escaping the host's adaptive immune system [2,59,62]. Further sequence data and analyses, as well as expression and cellular localization studies are needed to understand the function of this large protein family in *S. salmonicida*.

### Insights into the molecular basis of *S. salmonicida *pathogenicity

*Spironucleus *species have been described as a cause of disease in hosts that include birds, fish and mice [1]; *S. salmonicida*, for example, is a problem for the fish industry [3,4]. However, essentially nothing is known about virulence factors in *Spironucleus*. In healthy fish, the parasite *Spironucleus vortens *is commonly found in the flagellated stage (trophozoites) in the lumen of the upper intestine, where it remains attached to the intestinal mucosa, controlled by the mucosal immune system of the host. This stage of infection can cause diarrhea, but villus atrophy, ulceration or inflammation are thought to be absent [63], which is similar to observations during *G. lamblia *infections [62]. Antigenic variation among the identified cysteine-rich proteins in *S. salmonicida *could potentially protect the parasite against secretory antibodies at the mucosal surfaces. Nitric oxide (NO) and reactive oxygen species (ROS) are important factors in the host's protection against mucosal pathogens [62]. *S. salmonicida *encodes at least two rubrerythrins, two A-type flavoproteins and arginine deiminase (Additional file 2). These proteins, which are coded by genes putatively acquired from prokaryotes via gene transfer (see below), protect other microbes against ROS and NO [62,64,65], and similar roles are likely in *S. salmonicida*.

In the laboratory, *Spironucleus muris *is transmitted as cysts in fecal material like *Giardia *[66]. Cysts of the genus *Spironucleus *share many morphological features with those of *Giardia *including the presence of two to four nuclei, flagellar axonemes, and a distinct cyst wall. Cysts of *S. muris *even display the same immunostaining as *Giardia *cysts when labeled with antibodies specific for *Giardia *cyst wall [66]. We identified one homolog of *G. lamblia *cyst wall proteins, and two enzymes involved in the production of the sugar components of the cyst wall (glucosamine-6-phosphate isomerase and UDP-N-acetylglucosamine pyrophosphorylase – Additional file 2). The presence of these three genes indicate that *S. salmonicida *may also be able to form cysts, an interesting possibility since nothing yet is known of how *S. salmonicida *is spread and how it initiates infection in fish. These genes are stage-specifically expressed in the *G. lamblia *life cycle, suggesting that studies of their expression might identify regulatory regions of stage-specific genes in *Spironucleus*. Characteristic features of *Giardia *cysts include fragments of the adhesive disk but this structure, involved in attachment of *Giardia*, is conspicuously absent from *Spironucleus *cells. None of the genes encoding disc-specific proteins in *G. lamblia *were identified in this genomic survey, consistent with the absence of this structure in *S. salmonicida*.

In contrast to *G. lamblia*, trophozoites of *S. salmonicida *can enter the blood causing systemic and organ infections [67]. Cysteine proteases are essential for pathogenicity and invasion of the intestine of *E. histolytica *[68]. Therefore the several cysteine proteases that were found in our genomic survey are potential virulence factors possibly involved in invasion/tissue destruction by *S. salmonicida*. Secretion of cysteine proteases was indeed recently shown to occur from trophozoites of *G. lamblia *upon interaction with epithelial cells [69]. Cysteine protease activities are also important for excystation and encystation of *Giardia *[70,71], suggesting yet another potential role for these enzymes in *S. salmonicida*.

### Distinct codon usage patterns in highly versus weakly expressed genes

Genome-wide mutational processes have been identified as the main determinant for codon-usage variations among genomes in all three domains of life, while variations within genomes may be explained by selection [72,73]. We analyzed sequences of 1153 genes to examine the variations of codon usage in the *S. salmonicida *genome (Table 2). The random sequencing of ESTs from a non-normalized cDNA library provided a rough estimate of the expression levels of *S. salmonicida *genes, since the number of occurrences of a specific gene among the sampled clones should be correlated with the amount of mRNA in the cells that the cDNA library were created from. Analysis of the genes represented by more than ten EST clones ("highly expressed") showed a strong codon-usage bias where one or two codons dominate for each amino acid, while some codons are very rare (Table 2). A distinct pattern was found for genes never found in the EST library ("weakly expressed"), for which codon usage is more uniform, with no codons strongly dominating. To compare our results with a previous study from *Giardia *[74], 438 *G. lamblia *homologs to the *S. salmonicida *genes included in the codon-usage analysis were extracted. In the absence of information on expression levels, the *G. lamblia *genes were categorized according to the number of ESTs for their *S. salmonicida *homologs. The analysis revealed a pattern very similar to the previous study on a much smaller dataset [74]: codons ending with C or G dominate over codons ending with A or U in putatively highly expressed genes, while the putatively weakly expressed genes have a more uniform amino acid usage.

**Table 2:** Codon usage in highly and weakly expressed genes

		***Spiro *hi^1^**		***Spiro *weak^2^**		***Giardia *hi^3^**		***Giardia *weak^4^**				***Spiro *hi^1^**		***Spiro *weak^2^**		***Giardia *hi^3^**		***Giardia *weak^4^**	
		**#**	**RSCU**^5^	**#**	**RSCU**^5^	**#**	**RSCU**^5^	**#**	**RSCU**			**#**	**RSCU**^5^	**#**	**RSCU**^5^	**#**	**RSCU**^5^	**#**	**RSCU**^5^
**Phe**	**UUU**	80	*0.62*	3520	**1.21**	51	*0.41*	3584	*0.99*	**Ser**	**UCU**	208	**3.11**	2235	**1.51**	75	1.20	3962	**1.53**
	**UUC**	180	**1.38**	2317	*0.79*	197	**1.59**	3689	**1.01**		**UCC**	114	1.71	1009	*0.68*	125	**2.01**	2559	0.99
**Leu**	**UUA**	62	0.77	3475	**1.84**	4	*0.05*	1394	*0.44*		**UCA**	38	0.57	2035	1.38	22	0.35	2218	0.86
	**UUG**	17	0.21	1639	0.87	27	0.32	2466	0.77		**UCG**	7	*0.10*	1027	0.69	77	1.24	1825	0.70
	**CUU**	184	2.30	2163	1.15	123	1.44	5060	**1.59**	**Pro**	**CCU**	142	**2.27**	1372	1.31	49	0.65	2177	1.05
	**CUC**	210	**2.62**	1470	0.78	239	**2.80**	4307	1.35		**CCC**	87	1.39	650	0.62	118	**1.56**	2010	0.97
	**CUA**	0	*0.00*	1220	*0.65*	15	0.18	2302	0.72		**CCA**	20	0.32	1570	**1.50**	41	*0.54*	2369	**1.14**
	**CUG**	7	0.09	1366	0.72	105	1.23	3601	1.13		**CCG**	1	*0.02*	608	*0.58*	94	1.25	1720	*0.83*
**Ile**	**AUU**	155	0.96	4600	**1.52**	92	0.62	4220	1.09	**Thr**	**ACU**	194	**1.90**	2206	**1.36**	45	*0.53*	2757	0.97
	**AUC**	302	**1.87**	1977	*0.66*	302	**2.05**	4272	**1.11**		**ACC**	170	1.66	1322	0.81	103	1.22	2811	0.98
	**AUA**	28	*0.17*	2476	0.82	48	*0.33*	3086	*0.80*		**ACA**	34	0.33	1929	1.19	57	0.68	3544	**1.24**
**Met**	**AUG**	164	1.00	2280	1.00	181	1.00	4180	1.00		**ACG**	11	*0.11*	1047	*0.64*	132	**1.57**	2306	*0.81*
**Val**	**GUU**	261	**2.09**	2782	**1.61**	98	0.77	3608	**1.27**	**Ala**	**GCU**	321	**2.48**	2448	**1.27**	118	0.82	3529	1.00
	**GUC**	140	1.12	1252	0.72	297	**2.32**	3375	1.18		**GCC**	143	1.11	1570	0.82	228	**1.59**	3738	1.06
	**GUA**	81	0.65	1668	0.97	27	*0.21*	1591	*0.56*		**GCA**	46	0.36	2233	1.16	96	*0.67*	4588	**1.30**
	**GUG**	18	*0.14*	1210	*0.70*	90	0.70	2819	0.99		**GCG**	7	*0.05*	1437	*0.75*	132	0.92	2310	*0.65*
**Tyr**	**UAU**	145	**1.34**	2703	**1.31**	59	*0.55*	3095	*1.00*	**Cys**	**UGU**	45	*0.58*	1943	*0.80*	28	*0.39*	1437	*0.80*
	**UAC**	71	*0.66*	1430	*0.69*	156	**1.45**	3110	**1.00**		**UGC**	110	**1.42**	2893	**1.20**	116	**1.61**	2137	**1.20**
**Gln/TER**^6^	**UAA**	58	0.83	3576	**1.67**	21	1.91	54	0.94	**TER**	**UGA**	30	1.00	140	1.00	6	0.55	62	1.08
	**UAG**	44	0.63	1697	0.79	6	0.55	56	0.98	**Trp**	**UGG**	50	1.00	752	1.00	57	1.00	1458	1.00
**His**	**CAU**	70	*0.85*	1239	**1.22**	17	*0.22*	1595	*0.85*	**Arg**	**CGU**	235	**3.37**	764	0.99	100	1.18	1661	1.04
	**CAC**	95	**1.15**	796	*0.78*	136	**1.78**	2151	**1.15**		**CGC**	30	0.43	781	1.02	253	**2.98**	2403	**1.51**
**Gln**	**CAA**	30	*0.43*	1500	*0.70*	25	*0.23*	2676	*0.73*		**CGA**	1	*0.01*	392	0.51	19	0.22	1115	*0.70*
	**CAG**	147	**2.11**	1791	0.84	192	**1.77**	4640	**1.27**		**CGG**	1	*0.01*	352	*0.46*	7	*0.08*	1324	0.83
**Asn**	**AAU**	106	*0.68*	4502	**1.28**	61	*0.43*	3571	*0.91*	**Ser**	**AGU**	10	0.15	1269	0.86	15	*0.24*	1818	*0.70*
	**AAC**	208	**1.32**	2544	*0.72*	226	**1.57**	4291	**1.09**		**AGC**	24	0.36	1296	0.88	60	0.96	3171	1.22
**Lys**	**AAA**	191	*0.59*	5117	**1.28**	50	*0.16*	2778	*0.53*	**Arg**	**AGA**	141	2.02	1694	**2.20**	39	0.46	1575	0.99
	**AAG**	458	**1.41**	2902	*0.72*	565	**1.84**	7623	**1.47**		**AGG**	11	0.16	628	0.82	91	1.07	1459	0.92
**Asp**	**GAU**	208	**1.28**	3993	**1.26**	100	*0.55*	5063	*0.97*	**Gly**	**GGU**	345	**3.03**	2029	**1.24**	88	0.73	1923	*0.79*
	**GAC**	116	*0.72*	2333	*0.74*	264	**1.45**	5424	**1.03**		**GGC**	71	0.62	1672	1.02	215	**1.77**	2699	1.11
**Glu**	**GAA**	325	**1.53**	4444	**1.24**	53	*0.24*	4072	*0.71*		**GGA**	35	0.31	1816	1.11	59	*0.49*	2797	**1.15**
	**GAG**	101	*0.47*	2716	*0.76*	389	**1.76**	7431	**1.29**		**GGG**	4	*0.04*	1029	*0.63*	123	1.01	2342	0.96

^1)^*S. salmonicida *genes represented by more than 10 ESTs (35 genes, 6948 codons)

^2)^*S. salmonicida *genes unrepresented among the ESTs (682 genes, 122846 codons)

^3)^*G. lamblia *homologs to *S. salmonicida *genes represented by more than 10 ESTs (33 genes, 7004 codons)

^4)^*G. lamblia *homologs to *S. salmonicida *genes unrepresented among the ESTs (172 genes, 184988 codons)

^5)^Relative synonymous codon usage; the most and least abundant codons for each amino acid are indicated with boldface and italic font, respectively.

^6)^UAA and UAG encode glutamine in *Spironucleus*, and are termination codons in *Giardia *[25, 26].

### Selection on codon usage in highly expressed *S. salmonicida *genes

**Figure 4:**
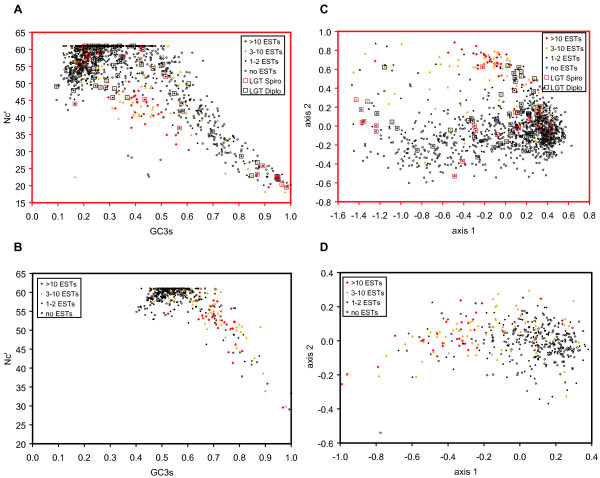
**Comparisons of codon usage in *S. salmonicida *and *G. lamblia***. A and B: The effective number of codons, N_c_', plotted against the synonymous G+C content in the third codon position (GC3_s_) of 1153 *S. salmonicida *genes (A) and 438 homologs in *G. lamblia *(B). Genes are represented by different colors and symbols according to the number of times they were sampled within the EST data. Genes identified to be involved in gene transfer and are shared with *G. lamblia *(LGT Diplo), or unique to *S. salmonicida *(LGT Spiro), are indicated by black and red squares, respectively (see Additional file 3). If the observed codon usage pattern is more uniform than expected by chance, the N_c_' value is set to 61 [75, 117]. C and D: Correspondence analysis of the relative synonymous codon usage (RSCU) values for the same genes as in A and B for *S. salmonicida *(C) and *G. lamblia *(D). The candidate LGTs unique to *S. salmonicida*, and shared with *G. lamblia*, are indicated by red and black open squares, respectively.

To investigate the codon usage in more detail, we plotted the effective number of codons calculated with a method that accounts for variations in the nucleotide composition (N_c_') [75] against the G+C content in the third synonymous position (GC3_s_), and performed correspondence analyses on the relative synonymous codon usage, for both the *Spironuclues *and *Giardia *datasets (Figure 4). The highly expressed *S. salmonicida *genes are centered in the plot with GC3_s _values around 0.4, and show lower N_c_' values than the weakly expressed genes with similar GC3_s _values, indicative of non-random synonymous codon usage (Figure 4A). For *G. lamblia*, the putatively highly expressed genes exhibit slightly higher GC3_s _and lower N_c_' values than the majority of the genes (Figure 4B). However, a separation of the genes into different categories can be observed: the vast majority of the weakly expressed genes have GC3_s _values between 0.4 and 0.7, while the highly expressed genes have slightly higher values (Figure 4B). Correspondence analyses showed that the G+C content in the third synonymous position is the main determinant of the codon usage in both *S. salmonicida *and *G. lamblia*; GC3_s _showed a strong correlation with the first axis (R^2 ^= 0.969 and 0.944, respectively: data not shown). Expression levels strongly influence the second axis in the correspondence analysis for *S. salmonicida*; the highly expressed genes are located at the top separated from the majority of the weakly expressed genes, when axis 2 is plotted against axis 1 (Figure 4C). No such trend is observed for *G. lamblia*, for which the putative expression levels seem mainly to be correlated with axis 1 (GC3_s_) (Figure 4D). These analyses clearly indicate that there is selection on codon usage in *S. salmonicida *genes classified as putatively highly expressed, and suggest that their *G. lamblia *homologs also are under selection (Figure 4 and Table 2). Translation efficiency has previously been suggested as the cause of the selection in highly expressed genes in *G. lamblia*, based on a much smaller data set [74], even though only estimates of expression levels were available for a few of the genes. Our study provides, for a much larger dataset, a connection between the subset of genes with a strong codon bias, and an indirect indication of expression levels (cDNA abundance), corroborating the earlier conclusion and extending it to the distantly related diplomonad *S. salmonicida*.

### A shift in codon usage within diplomonads

Interestingly, the favored codons differed between *S. salmonicida *and *G. lamblia *for ten amino acids, while the same codon is most abundant for eight (two amino acids are encoded by only a single codon) (Table 2). In nine of the cases where there is a difference between the two species, *S. salmonicida *preferentially uses a codon ending with U, while *G. lamblia *uses a C-ending codon in eight cases and a G-ending codon once. The tenth case is a two-fold degenerate amino acid where *S. salmonicida *and *G. lamblia *prefer A- and C-ending codons, respectively (Table 2). Thus, in general *S. salmonicida *favors A+U rich codons, while *G. lamblia *prefers codons with G or C in the third position in putatively highly expressed genes. There is a distinction between the base compositional biases in *G. lamblia *and *S. salmonicida*; the average G+C content of the *S. salmonicida *sequences in our dataset is 36%, and the majority of the weakly expressed genes have GC3_s _values around 20%, while the weakly expressed *G. lamblia *genes have GC3_s _around 50% close to the genomic G+C content of *G. lamblia *[60] (Figure 4A, B). This is an expected pattern if mainly mutational processes have shaped the codon usage [72,73]. However, the genomic G+C content differences seem to have influenced the codon preferences in the two diplomonad lineages in the highly expressed genes as well. In the absence of information about codon usage in a closely related outgroup, it is difficult to determine in which of the two lineages reassignment of the optimal codons has occurred. At any rate, the usage of an alternative genetic code in *Spironucleus *but not *Giardia *[26] indicates that codon usage have been remodeled in the lineage leading to *Spironucleus*, at least for the stop and glutamine codons. The canonical stop codon UAA is indeed the most common codon for glutamine in our dataset (Table 2), as expected in a G+C poor genome if the codon usage is close to mutational equilibrium [72,73].

### Unusual codon usage in a subset of *S. salmonicida *genes

We observed large variation among GC3_s _values for weakly expressed genes in *S. salmonicida*. Unexpectedly, the N_c_' values for genes with GC3_s _above 0.5 are negatively correlated with GC3_s_ (Figure 4A); if only the background nucleotide composition shaped the codon usage for these genes, the N_c_' values should be close to the maximum. This pattern indicates that there is selection on the codon usage in the group of weakly expressed genes with high GC3_s _values. However, selection for the set of optimal codons for highly expressed genes does not explain this pattern, as they include both G+C rich and G+C poor codons (Table 2). Also, the large spread of the genes to the left on axis 1 (which is strongly correlated with GC3_s  _– Figure  4C) argues against selection for an alternative set of optimal codons in these genes. Furthermore, the deviating GC3_s _values in these genes are probably not due to recent introductions into the *S. salmonicida *genome from heterogeneous sources by lateral gene transfer (LGT). Although some genes with high GC3_s _values do show indications of gene transfer in the phylogenomic analyses, the majority do not (Figure 4A, C). In fact, many of these genes have homologs in *Giardia*, indicating that they were present in the common ancestor of the two lineages, strongly suggesting that the deviated GC3_s _and N_c_' values arose during the evolution of diplomonads on the branch leading to *S. salmonicida*.

Analyses of the finished contigs also yielded unexpected results; the base compositional bias in segments of the *S. salmonicida *genome seems to be markedly towards G+C, while the largest part of the genome shows a compositional bias towards A+T (Figure 1). This pattern is in contrast with the A+T rich genomes of *D. discoideum*, *E. histolytica*, and *Plasmodium falciparum*, which show a relatively uniform nucleotide composition across all chromosomal regions [76,77]. The genes with deviating G+C content are correlated with these genomic regions of high G+C content (Figure 1). Furthermore, genes in G+C rich regions have higher GC3_s _values than the average G+C content for the genes (their bars are located above the sliding window analysis in Figure 1). Conversely, as expected, genes in G+C poor regions have low GC3_s _values. Thus, genes with unexpected codon usage (i.e. high GC3_s _values) are located in specific regions of the genome. However, neither mutational or selection scenarios can easily explain their non-random codon usage, nor the fluctuations in G+C content along the genome. Heterogeneity in overall G+C content (and/or GC3s) across the genome has been also observed in *Saccharomyces cerevisiae *[78] and in vertebrate genomes (e.g. isochores) [79]. For *S. cerevisiae *(a unicellular eukaryote with a compact genome, that is more directly comparable to *S. salmonicida *than vertebrates) a number of possible explanations have been advanced such as regional variation in propensities for mutation or recombination and partitioning of the genome into "distinct replicational and transcriptional domains" during specific cell cycle phases that may experience different chemical environments [see [78] and references therein]. Longer genomic fragments, as well as comparative data from more-closely related diplomonads, will be needed to characterize the pattern further in *S. salmonicida *and to tease apart possible causes for this peculiar phenomenon.

### Phylogenomic analysis reveals frequent lateral gene transfer

**Figure 5:**
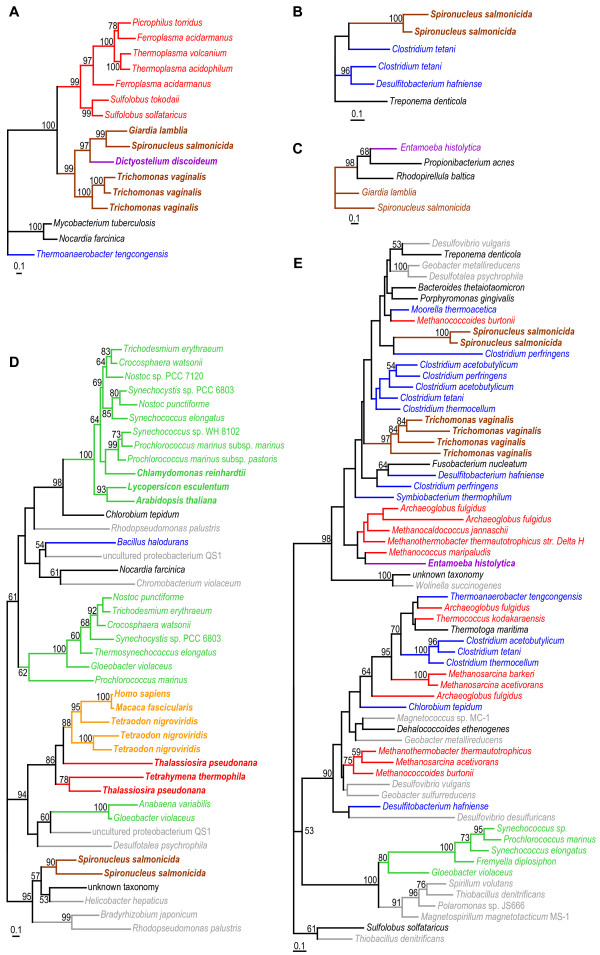
**Phylogenetic trees of five *S. salmonicida *genes from the phylogenomic analysis**. ML tree of conceptually translated, aligned amino acid positions of (A) arginine deiminase, (B) conserved hypothetical protein, (C) conserved hypothetical protein, (D) carotenoid isomerase, and (E) rubrerythrin. Bootstrap support values > 50% are shown. Details about the phylogenetic analyses are found in the Methods section, and complete accession numbers and complete species names are found in Additional files 4 and 6. The unrooted trees are arbitrarily rooted for the presentation. The branches and species names are labeled according to their phylogenetic classification: Archaea (red), proteobacteria (grey), low G+C Gram positives (blue), cyanobacteria (green), and other eubacterial lineages (black). Eukaryotes are in boldface and labeled according to their classification into super-groups [18]: Opisthokonta (orange), Amoebozoa (purple), Chromalveolata (red), Archaeplastida (green), and Excavata (brown).

We and others have previously studied the occurrence of LGT in diplomonads on a single-gene basis [16,17,29-31], [80-82]. Here we present a systematic phylogenomic analysis of the complete EST and GSS data sets with the goal of identifying *S. salmonicida *genes that have been involved in LGT. We used the PhyloGenie package [83] to assemble aligned amino acid data sets automatically from databases including both published sequences and data from ongoing eukaryotic genome projects (see Methods). Among the 711 genes with three or more identified homologs for which more than 100 amino acid positions could be aligned, 84 were retained as genes putatively involved in LGT affecting the *S. salmonicida *lineage, on the basis of unexpected phylogenetic positions (Figure 5, Additional files 3, 4, 5, 6). Cases where two *S. salmonicida *genes branched as a monophyletic group in the phylogenetic tree to the exclusion of other sequence were interpreted as gene duplication event after the gene transfer. Taking this into account, the 84 genes putatively involved in LGT corresponded to 68 unique gene transfer events at most; multiple genes may indeed have been introduced in a single event in some cases.

In principle, contamination in our libraries could falsely indicate a pattern of frequent gene transfer affecting the *S. salmonicida *genome. However, we strongly believe this not to be case. 57 of the 84 putatively transferred genes formed a monophyletic group with *G. lamblia *homologs in the phylogenetic analyses indicating that these genes were present in the common ancestor of the two species (Additional Files 3, 4, 5, 6). Furthermore, on average five in-frame UAG and UAA sense codons (which are termination codons in all characterized prokaryotes [84]) were detected in 21 of the remaining 27 putatively transferred genes, strongly suggesting a true *S. salmonicida *origin also for these genes. Four of the six genes without UAG or UAA codons formed a monophyletic group with a *S. salmonicida *gene which does utilize such codons, indicating a likely origin by gene duplication. We cannot formally exclude contamination on the basis of presence of alternative codons for the remaining two sequences (gTor1213bT7 and gZap260bT7, trees #59 and #64 in Additional file 6), but notice that the absence of in-frame UAG and UAA codons in these genes could likely be due to a recent introduction from a donor utilizing the canonical genetic code. At any rate, together these observations indicate that the phylogenetic pattern we interpret as putative LGT events are unlikely to result from substantial contamination of our libraries.

The strength and type of support for the inferred gene transfer events varied between genes in the phylogenomic analysis. Some *Spironucleus *genes were strongly associated with genes from distantly related organisms. For example, the *S. salmonicida *carotenoid isomerases were found to be nested within proteobacterial sequences (Figure 5D). Such anomalous positions of the diplomonads genes strongly suggest gene transfer events. Many datasets contain sequences from only one or a few eukaryotic taxa other than diplomonads (Figure 5A, C, E), and in some, diplomonads are the only eukaryotes present (Figure 5B–D). Although the separation of diplomonad from the other eukaryotic sequences is sometimes weak (i.e. Figure 5E), we suggest that LGT is a more likely explanation for their origin than differential gene losses and/or phylogenetic artefacts, as we have argued previously for genes with a patchy phylogenetic distribution among eukaryotes [80].

There are indeed examples of inferences of LGT based on unexpected patterns of phyletic distribution that have previously been shown to be wrong. Re-analyses of the putative bacteria-to-vertebrate gene transfer events in the human genome using phylogenetic analyses instead of similarity searches showed that more parsimonious alternative explanations existed for the vast majority, if not all, of the reported cases [85]. Phylogenetic analyses may also lead to false positive interpretations of LGT. For example, phylogenetic analysis indicated an LGT origin of the *T. vaginalis *hydrogenosomal NuoF protein distinct from the mitochondrial homologs [86], a conclusion shown likely to be incorrect upon re-analysis using more realistic models of sequence evolution [87]. On the other hand, re-analyses using extended taxon sampling and more advanced models of sequence evolution of four genes putatively affected by prokaryote-to-protist LGT resulted in support for a larger number of inter-domain gene transfer events, rather than a fewer number as expected if differential gene loss, and/or phylogenetic artefacts caused the initial indications of gene transfer [30,80]. Obviously, inferences about LGT from the phylogenetic analyses depend on the accuracy of the phylogenetic method, as well as on the breadth of organismal representation in sequence databases. Therefore, it is inevitable that the list of putative LGTs will include both false positives and false negatives (Additional files 3, 4, 5, 6). Nevertheless, our phylogenomic analysis indicates that vertical inheritance combined with gene duplication and gene loss is rather unlikely to have produced the observed phylogenetic relationships and patchy taxonomic distributions and that gene transfer is the simplest hypothesis currently available to explain these trees. Hence our analyses indicate that LGT has been an important mechanism in the evolution of the *S. salmonicida *genome.

### Acquisitions of prokaryotic metabolic genes in the evolution of protist genomes

The vast majority of genes inferred to be involved in gene-transfer events are metabolic genes (74%) or encode conserved hypothetical proteins (20%) (Additional file 3). This bias is not unexpected, as a higher rate of transfer for metabolic genes compared to informational genes has been observed for prokaryotes [88,89] and, on a smaller scale, in diplomonads [30] and *E. histolytica *[51]. In the latter study it was suggested that this protist parasite had expanded its metabolic repertoire via gene acquisitions from prokaryotes present in the environment, and the same may apply for *S. salmonicida*. For example, amino acids have been identified to be a source of energy in diplomonads via the arginine dihydrolase pathway [2] especially under limited oxygen conditions [90]. Among eukaryotes, this pathway has been detected only in *Trichomonas *and diplomonads. The genes for two enzymes in the pathway, arginine deiminase and ornithine transcarbamoylase, appear to have been acquired from prokaryotes in a common ancestor of diplomonads and parabasalids, whereas carbamate kinase may have distinct LGT origins in the two groups (Figure 5A and trees #10 and #30 in Additional file 4). Similarly, *S. salmonicida *encodes rubrerythrins and A-type flavoproteins, proteins putatively involved in the protection against the host's defense (see above) [62,64,65] that are also found in anaerobic prokaryotes and eukaryotes including *Entamoeba *(Figure 5E and Additional files 3, 4, 5, 6) [51,80]. The phylogenetic distributions of these genes suggest a role related to an anaerobic lifestyle, consistent with the hypothesis that these acquisitions provided metabolic advantages to the recipient protist lineages. Indeed, distantly related lineages that live in the same environment appear to frequently transfer genes [89]. Increased taxon sampling and more detailed studies of the ecology and metabolism/biochemistry of the organisms represented by sequences in our trees will likely recognize shared environments between the donor and recipient lineages for additional acquired metabolic genes (Additional file 3).

### Continuous exchange of genes with all domains of life

In this study we have identified 84 genes of putatively lateral origin, and in previous phylogenomic studies 96 *E. histoytica *genes, almost 50 kinetoplastid genes, 24 in *Cryptosporidium parvum *and 148 genes in anaerobic ciliates were found to represent candidate LGT genes [51,55,91,92]. Thus, numerous inter-domain gene transfer events have been identified in four divergent protist lineages – *Spironucleus*, *Entamoeba*, kinetoplastids (*Trypanosoma *and *Leishmania*) and anaerobic ciliates – supporting the idea that gene acquisition from prokaryotic organisms is a common evolutionary mechanism in unicellular eukaryotes [22,93-95]. In all these studies, metabolic genes have been found to be the most common functional category among the genes implicated in LGT genes. This observation, which is consistent with the complexity hypothesis [88], could indicate that metabolic adaptation is a selective force for inter-domain gene transfer events from prokaryotes to protists [96,97].

*G. lamblia *homologs that form clades with the *S. salmonicida *sequences were found in 49 of the 68 putative LGT events, while no closely related *G. lamblia *homologs were found in the remaining 19 cases (Figure 5 and Additional file 3). Thus, at least 72% of the putative LGT events happened before the divergence of the *Spironucleus *and *Giardia *lineages. The remaining 19 events could have taken place in the *S. salmonicida *lineage after the divergence from the lineage leading to *G. lamblia*, although gene losses in the latter could also explain the observed pattern. If these represented relatively recent gene transfers, they should show a codon-usage pattern distinct from that of other *S. salmonicida *genes. Some in fact do show deviant codon usage indices (Figure 5A, B) suggesting a more recent acquisition compared to the other cases. However, there are many other *S. salmonicida *genes with deviant codon usage that do not show any indication of LGT (Figure 4A, B), suggesting that codon usage pattern alone is a poor indicator of LGT in *S. salmonicida*, as shown in other systems [98]. In 12 cases where both *Spironucleus *and *Giardia *sequences are present, *T. vaginalis *sequences are found as a sister group to the diplomonad cluster. This pattern has previously been observed for candidate laterally transferred genes [16,17,80] and almost certainly reflects a common ancestry of diplomonads and parabasalids to the exclusion of other sampled eukaryotic lineages, although the possibility of gene transfer between the two lineages cannot be formally excluded. However, a diplomonad-parabasalia relationship has recently been shown to be robust in several phylogenetic analyses of concatenated protein sequences [13-15]. As some acquired genes are unique to *S. salmonicida*, others shared with *G. lamblia*, and some are also shared with *T. vaginalis*, this strongly suggests that genes have been acquired by LGT in these excavate lineages continuously throughout their evolutionary history. Further characterization of the phylogenetic distribution of these genes may provide information about organismal relationships within Excavata [99].

In the four previous phylogenomic studies of LGT in protists [51,55,91,92] only prokaryote-to-eukaryote transfer events were examined. Our selection procedure allowed us to detect additional evolutionary scenarios where a prokaryotic gene is transferred to a eukaryote, and then possibly further transferred to a second eukaryotic lineage. For example, the arginine deaminase gene was likely acquired from a prokaryote by an ancestor of diplomonads and parabasalids, and then transferred to the lineage leading to *Dictyostelium *(Figure 5A). Seventeen genes were putatively affected by intra-domain LGT, corresponding to 13 unique events (Additional file 3). Twelve of these cases are exchanges between the lineage leading to *S. salmonicida *and amoebozoan lineages; five cases involve gene exchange with the *Dictyostelium *lineage, six cases with the *Entamoeba *lineage, and a single case with the *Mastigamoeba *lineage (Figure 5A, Additional files 3, 4, 5, 6). In sharp contrast, only a single intra-domain gene transfer event representing other eukaryotes was detected, although opisthokonts are comparatively well sampled with genome sequences, and several diverse and complete, or nearly complete, chromalveolate sequences were present in our data sets (Additional files 3, 4, 5, 6). It is noteworthy that only genes that have been acquired from prokaryotes and have subsequently been transferred within the eukaryotic domain will be detected using our selection procedure. Additional eukaryote-to-eukaryote transfers may actually have occurred among the genes that did not show any indication of prokaryote-to-eukaryote transfer. A few examples of gene transfers between unicellular eukaryotic organisms have indeed been published recently [22,80,100,101]. The 13 putative intra-domain transfers identified here further suggest that LGT between protists is an evolutionary mechanism that should not be neglected.

## Conclusion

The vast majority of eukaryotic diversity is represented by protists [18], yet only a few protist genome sequencing projects have been published. Our sequence survey study has indicated that a combined approach using both random sampling from the genome (GSSs) and ESTs is successful in identifying genes (Table 1). We identified 817 genes from the GSS sequences, while 473 genes with homologies in other organisms were detected among the EST sequences. As we have collected more than twice as many GSS as EST sequences, EST sequencing would appear to be slightly more effective for gene discovery, if only quantity is considered, as expected. However, EST sequencing is biased towards identification of highly expressed genes, such as genes involved in genetic information processing, especially translation, while GSS sequencing detects a more random selection of genes (Table 1). This functional bias of the genes detected in EST surveys may be an advantage if the objective of the study is mainly to identify genes previously identified in other organisms using the same approach, but is a limitation if sampling gene diversity is the aim.

We found that a combined approach of both GSS and EST sequencing can be successful in detecting both highly expressed (and probably also often widely distributed) genes and a more diverse gene set. In combination with complete sequencing of a few contigs, this approach was efficient in revealing much about the *S. salmonicida *genome. Although we could identify more than 600 genes with annotated functions, conserved hypothetical proteins still represent the largest category (Table 1), indicating that the genes with annotated functions give only a partial picture of the true coding potential. Furthermore, 13 among the 38 genes identified within the contigs did not show any significant sequence similarity to genes in the databases, and 81% and 45% of the GSS and EST sequences, respectively, failed to show significant similarity to any known genes. This suggests that a large fraction of the genes in *S. salmonicida *genome lack sequence similarity to known genes, despite the fact that a nearly complete *G. lamblia *gene complement is included in the public database. Thus, the *S. salmonicida *genome has a significant, and mostly unknown, coding potential. Still, the analyses of the genes that could be annotated not only identified individual *S. salmonicida *genes and metabolic pathways that provided insight into the biology and evolution of the organism. In addition, these analyses revealed several lineage-specific properties suggestive of a large genomic diversity between *S. salmonicida *and other studied eukaryotes, including its closest intensively studied relative, the diplomonad *G. lamblia*.

Our analyses indeed indicate that diplomonad genomes are diverse. For example, in the *S. salmonicida *genome we identified gene acquisitions, a base compositional bias that varies along the genome, a codon usage distinct from that of *G. lamblia*, and differences in basic molecular biological processes such as polyadenylation. *G. lamblia *and *S. salmonicida *represent only two species within diplomonads, a paraphyletic group which may also include enteromonads and retortamonads, organisms with distinct morphological features [23,24,102]. The understanding of these interesting groups of protists is very limited on the genomic level, and the genome projects of *G. lamblia *[103] and *Spironucleus vortens *[104], another fish pathogen, will make major contributions. Still, diplomonads are very diverse, as manifested by a large degree of sequence divergence between members of the group, and, as indicated here, large variation in the genomic structure and content. Circumstantial evidence for a relatively small genome (see above), together with an overall G+C content of 36% and a low frequency of repeats, makes *S. salmonicida *an ideal candidate for a whole-genome sequencing project. Such an effort would yield further insights into the parasitic lifestyle of this organism, the fascinatingly diverse biology of diplomonads, and expand our appreciation of genome diversity and evolution among eukaryotes.

## Methods

### Sources of RNA and DNA, library construction, and sequencing

*S. salmonicida *(ATCC 50380) [6] (previously known as *S. barkhanus *[5,105]) was grown in axenic culture following the ATCC protocol. Messenger RNA from approximately 10^8 ^cells of *S. salmonicida *was isolated using the Dynabead mRNA system (Dynal), and cDNA was synthesized and cloned into the lambda Uni-Zap XR vector (Stratagene), according to the manufacturer's instructions. This procedure requires that the polyA tail is present in the mRNA to be cloned. Infection of SolR cells was carried out and plated on selective LB media containing carbenicillin, isopropyl β-D-1-thiogalactopyranoside (IPTG) and 5-bromo-4-chloro-3-indolyl-beta-D-galactopyranoside (X-gal) for blue/white selection. Positive colonies were picked manually and plasmid DNA was purified using Perfectprep Plasmid Isolation Kit with the Perfectprep Plasmid 96 Vac system (Eppendorf). The DNA was quantified by electrophoresis on a 1% agarose gel. Sequencing was carried out on an ABI 377 (Applied Biosystems) using the ABI Big Dye Terminator Cycle Sequencing Ready Reaction Kit (Applied Biosystems) and the SK primer.

Genomic DNA was purified using standard protocols. A genomic DNA library was constructed for GSS sequencing. The DNA was partially digested using *Sau*3AI and ligated to lambda Zap Express vector pre-digested with *BamH*I (Stratagene) according to the instructions. Mass *in vivo *excision of the pBK-CMV phagemid from the Zap Express vector was performed using XL-1 Blue MRF' cells and ExAssist phage with the XLOLR strain, and the cells were plated on LB with kanamycin, IPTG and X-gal for blue/white selection. Cells were picked and the plasmids were purified and sequenced as described above using the T3 and T7 primers. Roughly half of the sequencing was done from this library. However, since it was revealed that the insert sizes of this library were too short for efficient random sequencing, a second GSS library was constructed using 20 μg genomic DNA. The DNA was physically sheared using a nebulizer, blunt ended, ligated to the insert DNA and cloned into plasmid vectors using the TOPO Shotgun Subcloning Kit (Invitrogen). One Shot chemically competent cells (Invitrogen) were transformed with an aliquot of the ligation mix and spread on LB plates containing kanamycin and X-gal for blue/white selection. Positive clones were manually picked and plasmids were purified using 96-well Plasmid Preparation Kit (Millipore). The plasmids were quantified and sequenced as described above using T3 and T7 primers.

Finally, *S. salmonicida *DNA was partially digested with *Bam*HI and cloned into the lambda DASHII vector (Stratagene) according to the manufacturer's instructions, to make a library with larger inserts (8–28 kbp). The library was grown in MRA cells and spread on eight 150-mm plates each containing approximately 70,000 plaques. Three plaques were purified and the inserts were amplified by PCR, nebulized and the fragments were shotgun cloned into puc18 *Sma*I/BPA plasmids (Amersham Pharmacia). The ligations were transformed into XL2-Blue MRF' ultracompetent cells. Plasmids were purified and sequenced as described above using M13f and M13r primers.

### Sequence analysis

Sequencing reactions were initially screened manually for exclusion of unsuccessful reactions. Sequences from 2408, 3204 and 3684 clones from the Uni-Zap XR EST, the Zap Express GSS and TOPO Shotgun Subcloning GSS libraries, respectively, passed this initial screen. The average read lengths of these were 540, 399 and 366 bp, respectively.

The 2408 EST sequences were screened against the vector sequences and the *Escherichia coli *genome sequence using the Phred software [106] to check for contamination of DNA from the library host, and clustered using the Phrap assembler [106] with the default settings. The contigs were quality trimmed manually, while the singletons were trimmed using the Phred software [106] with the default quality cut-off. Hybrid clones were identified as containing stretches of nine or more As within the insert, and only the 5' end of the hybrid was retained. 27 clones with inserts in the opposite direction were identified as starting with a stretch of Ts (15), or as having significant matches in the wrong direction to known genes (12); these were reversed. Finally, nine EST sequences were trimmed according to putative frame-shifts detected in similarity searches. After removal of contigs shorter than 100 bases 884 sequences (298 contigs and 586 singletons) corresponding to 502589 unique bp and based on 2341 clones remained for further analysis. 20 and 11 of these overlapped with published genes or genes from the finished contigs (see below), respectively, and were excluded, leaving 853 ESTs for further analyses.

300 ESTs ended with a stretch of eight or more As, indicative of mRNA polyadenylation, were identified. Of these, 134 were annotated as coding based on sequence similarity to genes in the databases (see below). The region around the 3' end of the gene was aligned based on the position of the termination codons (UGA), and a sequence logo was created using WebLogo [107].

The 6888 GSS sequences were trimmed using the Phred software [106] with the default quality cut-off, and the vector sequences were masked and removed. All sequences were screened against the *E. coli *genome sequence. The 5275 GSS chromatograms that passed this screen (no host contamination was detected), and represented sequences longer than 100 bp, were included in the assembly. The average read lengths of the clones from the Zap Express GSS and TOPO Shotgun Subcloning GSS libraries that passed this screening were 543 and 472 bp, respectively. The Phrap assembler was used with the default settings, yielding 1008 contigs consisting of two or more sequences and 2208 singletons. Some of the longer contigs were selected for complete sequencing. Regions that required additional sequencing were identified within these contigs, and amplified from genomic DNA using PCR with primers designed based on the GSS assembled sequence. The PCR was performed under standard conditions using the following parameters: denaturing at 95°C for 5 min, followed by forty cycles of denaturing at 94°C for 30 s, annealing at 48°C for 1 min, and extension at 72°C for 2 min, and a final extension at 72°C for 10 min. To extend the contigs, sequence gaps covered by clones were identified and amplified using PCR with specific primers based on the obtained shotgun sequences. The PCR products were purified using the Qiaquick PCR Purification Kit (Qiagen) and sequenced using the PCR primers as described above. This procedure resulted in eight continuous stretches of genomic sequence between 2365 and 9005 bp in length. Together with the three sequenced lambda clones these yielded 80504 bp of finished genomic sequence in eleven contigs which were covered by at least one-fold high quality sequence in each direction (Additional file 1). 205 of the 5275 GSS sequences overlapped with the completely sequence contigs and were excluded from further analysis. The remaining 5070 sequences were treated as single reads, and covered 2539160 bp of non-unique sequence after quality trimming.

To estimate the amount of unique high-quality sequence within our survey project, we performed assemblies with only the quality trimmed sequence using the Staden package [108]. The 5275 GSS clones yielded 2070953 bp in 3566 contigs with one or more sequences, and the addition of the EST clones gave 2549915 bp of unique high-quality sequence. Lander and Waterman proposed that the number of "islands" (E) in a genomic project with perfectly representative libraries with equal length of clones (sequences) is E = Ne^-cσ ^[109], where N is the number of clones sampled, c is the redundancy of coverage = LN/G, σ = 1 - T/L, L is the clone length (500.8 bp in our case), G is the haploid genome size in bp, and T is the amount of overlap in bp needed to detect overlap (20 bp). G is the only unknown parameter, and the equation can be solved: G = N(L-T)/ln(N/E). Using the information from our assembly (N = 5275; L = 500.8 bp; T = 20 bp; E = 3566), the genome of *S. salmonicida *was estimated to be 6.5 Mbp in size.

### Databases

All databases used in the analyses were downloaded in January 2005. In addition to the non-redundant protein database downloaded from the National Center for Biotechnology Information (NCBI) [110], protein databases from various nearly completed genome sequencing projects of diverse eukaryotes were downloaded: *T. vaginalis *(parabasalid), *Tetrahymena thermophila *(ciliate), *Trypanosoma brucei*, and *Trypanosoma cruzi *(kinetoplastids) from the Institute for Genomic Research (TIGR) [111]; *Dictyostelium discoideum *(mycetozoan) from dictyBase [112]; *Chlamydomonas reinhardtii *(green alga), *Phytophthora sojae *(oomycete), and *Thalassiosira pseudonana *(diatom) from the DOE Joint Genome Institute (JGI) [104]. The Kyoto Encyclopedia of Genes and Genomes (KEGG) databases were downloaded from the Kyoto University Bioinformatics Center [113] for functional annotation purposes.

### Gene identification

Various approaches were used to identify coding genes among the sequences. Similarity searches using BLASTx, version 2.2.13 [114], with the default settings with the nucleotide sequences from the single-read GSS and the EST contigs against the amino acid sequence databases were performed. 1179 single-read GSS sequences and 502 EST contigs yielded matches with E values better than e^-5^, which were considered a significant indication of a coding sequence. Using the indication of the frame and direction from the alignment between the query and database sequence in the BLAST result files, the putative coding DNA and amino acid sequences were extracted from these sequences. The 3891 single-read GSS sequences and 380 EST contigs that failed to yield a match better than e^-5 ^were not analyzed further.

To identify coding sequences in the finished contigs, similarity searches against the amino acid sequence databases and the EST sequences were performed using the same cutoff as above. Similarity searches on the nucleotide level against the non-redundant nucleotide databases were performed at NCBI to identify non-protein coding sequences. Finally, open reading frames longer than 450 bp with the expected GC pattern in the three coding positions were annotated as putatively coding and included in subsequent analyses. This procedure identified 38 protein-coding genes in the eleven finished contigs.

tRNA genes were detected using two programs: tRNAscanSE version 1.23 [115] with the maximum sensitivity (-C option), and ARAGORN version 1.1 [116] with the default parameters. 20 tRNA genes were identified with both methods, all within GSS clones. For 19 of these, both programs assigned the same anticodon, while one differed in anticodon identity (see Results). Two tRNAs were found to encode putative introns. No tmRNA was found. Finally, similarity searches at the nucleotide level identified two genes encoding ribosomal RNA within one of the finished contigs.

### Creation of a non-redundant dataset of *S. salmonicida *protein-coding genes

In order to explore the coding potential of *S. salmonicida*, we created a non-redundant dataset of *S. salmonicida *genes that contained only the longest amino acid sequence from each gene among the GSS, EST, finished contigs and previously published genes. Three of the 30 previously published genes were represented by long ESTs and were excluded. This procedure resulted in538 unique protein-coding genes. 175 of the 1179 genes identified within the GSSs were excluded because they were represented by longer ESTs or published sequences, while 30 were excluded because the extracted ORF was shorter than 100 bp. Furthermore, 176 of the remaining 974 genes identified within GSS sequences were excluded because they overlapped with a longer identical gene within the GSSs, leaving 798 unique protein coding genes identified within the GSS data. Thus, in total 1335 unique protein-coding genes were identified and included in further analyses.

### Codon-usage analysis

1153 of the 1335 unique protein-coding sequences were 300 bp or longer and were included in the codon-usage analysis. Codon-usage indices were calculated for each gene. N_c _is a measure of the effective number of codons used in a gene [117]. A modified version, N_c_', has been developed which also account for the background nucleotide composition of the gene; this is advantageous in situations where the composition varies among the genes analyzed [75]. The N_c_' values were calculated using the software INCA [118]. The GC3_s _values – the frequency of G+C in synonymously third codon positions (i.e. Met, Trp and termination codons are excluded) – were calculated using the program CodonW [119]. The variation of codon usage among genes was explored using the correspondence analysis tool within CodonW. To avoid identification of trends in codon usage due to biased amino acid usage among the genes, the correspondence analysis was performed on the relative synonymous codon usage (RSCU) values for each gene. The RSCU value for a codon is the observed frequency divided by the frequency expected if all synonyms codons for that amino acid were used equally. This should remove the effects of amino acid composition on codon usage. RSCU values close to 1 indicate a lack of bias, while much higher and much lower values indicate preference and avoidance of that particular codon, respectively. The correspondence analysis plots genes according to their RSCU values in a 61-dimensional space, and then identifies the major trends as the axes through this multidimensional hyperspace which account for the largest fractions of variation among genes.

To examine the variation of codon usage within diplomonads, similarity searches of the 1153 *S. salmonicida *amino acid sequences were performed against the *G. lamblia *protein sequences. 533 *S. salmonicida *genes gave matches with E values < e^-20^, corresponding to 438 unique genes. These were considered homologs (not necessarily orthologs) and the nucleotide sequences were obtained for codon-usage analysis as described above.

The number of times each gene was detected in the EST library was used as an indication of expression level in the cell. 35 genes were represented by more than ten individual EST sequences and were considered highly expressed, while 682 genes were not detected in the EST data and were considered weakly expressed. Codon-usage tables were calculated for each of these groups, both for *S. salmonicida *and the *G. lamblia *homologs. *G. lamblia *genes represented by several *S. salmonicida *genes were assigned the highest number of ESTs among the genes.

### Gene annotation

The Kyoto Encyclopedia of Genes and Genomes (KEGG) project [113], a bioinformatic knowledge base for systematic analysis of gene functions [120], was used for gene annotation. The KEGG system links genomic information with higher-order functional information using a system with KEGG Orthology (KO) numbers. Gene sequences in the GENES database, which includes collection of gene catalogs from completely sequenced genomes, are assigned KO numbers which are used to extract higher-order information, such as functional categories and pathways, from the KO database [120].

Similarity searches using BLASTx, version 2.2.13 [114], with the default settings were performed for the 1335 unique *S. salmonicida *protein-coding genes against the GENES database, using the E value cutoff e^-5^. 249 returned a best match to which a KO -number had been assigned, and an additional 301 had non-best hits with assigned KO numbers. Functional annotations were extracted from the KO database for these 550 genes. However, 27 genes were annotated in the functional category "Human Disease"; five of these could be manually assigned to functional categories, while 22 were classified as conserved hypothetical proteins. Thus, 528 genes could be assigned a function using the KEGG databases. Similarity searches against all databases were performed for the remaining 785 genes, using the E value cutoff e^-20^. 219 of these had matches, 63 of which could be used for functional annotation. No meaningful functional information could be found for remaining 156; these were annotated as conserved hypothetical proteins. In addition, the 553 genes which had best hits with E values between e^-20 ^and e^-5 ^were annotated as conserved hypothetical proteins, and the remaining 13 genes that did not have any match better than e^-5 ^were annotated as hypothetical proteins. To summarize: 591 protein-coding genes could be assigned a putative function, 731 were conserved hypothetical proteins, and 13 hypothetical proteins. The annotations for all proteins mentioned in the text have been refined manually.

The fraction of cysteine within the conceptually translated amino acid sequences was found to vary considerably between genes. While the majority of genes would encode a protein with cysteine content below 5%, 149 of the 1335 unique genes would encode a protein with more than 10% cysteine. These were annotated as cysteine-rich proteins. 122 are conserved hypothetical proteins, while 27 were assigned to functional categories.

A data set of 1044 yeast proteins localized in the mitochondrion [121] was used to search against the identified *S. salmonicida *proteins using BLASTp, version 2.2.13 [114], with the default settings. The matches with e values below e^-5 ^were searched against the total yeast proteome with the same cutoff, which identified 71 reciprocal best matches between the datasets. Based on the quality of the BLASTp hit (or protein length, to short to give any confidence in the identified HSP), phylogenetic trees and functional annotation of BLAST hits, only two entries were considered as strong candidate mitochondrial/mitosomal proteins and one as possible candidate proteins (see main text).

### Phylogenomic and phylogenetic analyses

One goal of this project was to identify genes in the *S. salmonicida *genome that have been affected by LGT. We used phylogenetic analyses to identify gene transfers, as similarity searches are only poor indicators of such events [122]. We used the PhyloGenie package [83] to perform efficient phylogenetic analyses for a large number of genes. It takes amino acid sequences in standard (fasta) format, and performs similarity searches (BLAST) against a protein sequence database consisting of publicly available databases and/or sequence data released from ongoing genome projects. The program uses HMMER [123] to build a Hidden Markov Model (HMM) profile from the alignment in the BLAST result file, and uses it to search the full-length BLAST hits; sequences scoring better than a selected threshold are included in the dataset. Finally, an alignment of these sequences is made using the profile. No attempt was made to exclude putative paralogs from the datasets.

Cysteine-rich proteins were excluded from the phylogenomic analysis, since they were found to be problematic to align probably due to their frequent possession of highly repetitive sequences. PhyloGenie was run on the 1174 annotated non-cysteine-rich genes with homologs in the databases, using default settings except that coverage of the query sequence compared to the database sequence was not used as a selection criteria (coverage = -1) since coverage is meaningless with partial gene sequences such as EST and GSSs, and the maximum number of sequences in the dataset was set, for practical reasons, to 200 (seqs = 200). Where more than 200 sequences pass the E-value cutoff, the 200 with the lowest E-values in the HMM-based search will be retained. A custom-implemented function (T. Frickey, personal communication) was used that allows for exclusion of similar sequences from the dataset based on the taxonomic level (i.e. only one sequence is retained as a representative of all *Bacillus *sequences above a set identity). To reduce the size of the dataset as much as possible without losing too much information, an identity cutoff of 80% was used regardless of the taxonomic description in the sequence (taxlevel = 0, maxsim = 0.8).

These settings yielded 932 datasets with four sequences or more, among which 711 included 100 or more aligned amino acid positions assessed on the *S. salmonicida *input sequence. Only these were analyzed further, to avoid misleading results based on too-short alignments. A Perl script provided in the PhyloGenie package [83] was modified to use the fast maximum likelihood program PHYML version 2.4.4 [124]. Trees were obtained for each of the 711 datasets using this script and the default PHYML settings, except that the Whelan and Goldman (WAG) substitution model [124] was used together with a mixed four-category discrete-gamma model of among-site rate variation plus invariable sites (WAG + Γ + Inv).

The trees were automatically rooted using the approach implemented in Phylogenie; taxonomic information for all sequences in each tree is used to put the root at the most-basal node that is most-distant from the sequence that was used to select the dataset [see Figure 4 in [83] for details]. Phatg, the tree-browsing program within the PhyloGenie package [83], was used to identify 136 putative cases of LGT among the optimal trees, using the criterion that a diplomonad sequence was required to group with homologs from up to four different non-diplomonad eukaryotes within a prokaryotic clade. Manual inspection of the positive trees excluded from further analysis 31 trees for which the input *S. salmonicida *sequence failed to show convincing indications of LGT, or the *G. lamblia *homolog branched with a prokaryotic sequence. Bootstrap analyses were performed for the remaining 105 datasets, using 100 replicates and the methods and settings described above. Visual inspection of the consensus bootstrap trees yielded 84 that showed indications of LGT affecting *S. salmonicida*. Among these, two appear to be clones derived from different parts of the same *S. salmonicida *gene, and 14 are part of diplomonad gene clusters which may have formed via gene duplication events after a putative transfer event. Thus, our phylogenomic analysis identified 68 putative LGT events (Additional files 3, 4, 5, 6).

### Nucleotide sequence accession numbers

The sequences reported here were deposited in GenBank at the National Center for Biotechnology Information [Genbank:EC585128-EC586011, GenBank:DX913336-DX918405, GenBank:DQ812518-DQ812528].

## Authors' contributions

JOA co-constructed the second GSS library, finished the sequence of seven contigs, carried out the EST and GSS assemblies, all phylogenetic and most bioinformatic analyses, and drafted and coordinated the editing of the manuscript. ÅMS co-constructed the second GSS library, carried out the majority of the GSS sequencing, and performed initial bioinformatic analyses. DSH grew the organism, purified nucleic acids, constructed the EST library, and carried out some of the initial EST sequencing. CAM constructed the lambda and the initial GSS libraries, carried out the majority of the EST sequencing, the initial GSS sequencing and the sequencing and assembly of three lambda clones. PLD carried out some of the initial EST sequencing. SGS provided advice on the analyses of molecular biological aspects and drafted these parts of the manuscript. RPH performed part of the analyses of cysteine rich and putative mitosomal proteins. JML, MAR, RPH, and AJR co-initiated and supervised the project in their respective laboratories, provided advice on the analyses and edited the manuscript. All authors read and approved the final manuscript.

## Supplementary Material

Additional file 1 A table showing the characteristics of the finished contigs. Click here for file

Additional file 2 Complete list of gene annotations. Click here for file

Additional file 3 A table showing the genes putatively involved in LGT events. Click here for file

Additional file 4 Phylogenetic trees 1–25 for genes putatively involved in LGT events and listed in Additional file 3. Click here for file

Additional file 5 Phylogenetic trees 26–50 for genes putatively involved in LGT events and listed in Additional file 3. Click here for file

Additional file 6 Phylogenetic trees 51–72 for genes putatively involved in LGT events and listed in Additional file 3. Click here for file
