# Supplementary material for: A genomic survey of the fish parasite Spironucleus salmonicida indicates genomic plasticity among diplomonads and significant lateral gene transfer in eukaryote genome evolution
Source: BMC Genomics. 2007 Feb 14;8:51. doi: 10.1186/1471-2164-8-51 (PMC1805757; doi:10.1186/1471-2164-8-51)
Supplement: Additional file 1 — A table showing the characteristics of the finished contigs. [file 1471-2164-8-51-S1.pdf]

A genomic survey of the fish parasite *Spironucleus salmonicida* indicates genomic plasticity among diplomonads and significant lateral gene transfer in eukaryote genome evolution

Jan O. Andersson, Åsa M. Sjögren, David S. Horner, Colleen A. Murphy, Patricia L. Dyal, Staffan G. Svärd, John M. Logsdon, Jr., Mark A. Ragan, Robert P. Hirt, and Andrew J. Roger

Additional file 1

Characteristics of the *Spironucleus salmonicida* finished contigs.

	overall				coding			non-coding	
	length	GC	#genes	coding	length	GC	$GC3_s$	length	GC
Sp1	14440	47,4%	6	82%	11901	45,6%	45,8%	2539	47,3%
Sp2	12550	30,7%	9	96%	12093	30,0%	23,7%	457	20,8%
Sp3	14129	31,2%	9	84%	11853	31,2%	22,5%	2276	25,5%
Sp4	4892	24,9%	0	-	0	-	-	4892	24,9%
Sp5	1856	35,9%	1	100%	1851	34,5%	28,4%	3	0%
Sp6	2365	42,3%	1	46%	1092	45,7%	46,6%	1273	37,9%
Sp7	4163	36,4%	3	88%	3651	36,6%	28,8%	512	25,3%
Sp8	5489	58,3%	3	75%	4113	57,5%	70,3%	1376	58,2%
Sp9	9005	32,6%	2[1]	31%	2823	45,0%	-[1]	6182	27,0%
Sp10	3418	44,8%	2	60%	2058	45,5%	54,3%	1360	42,1%
Sp11	8197	54,3%	4	82%	6681	53,1%	53,1%	1516	54,0%
TOTAL	80504	39,3%	40	72,2%	58116	39,9%	37,3%	22388	37,9%

[1)]This contig encodes 18S and 28S ribosomal RNA.
