# Supplementary material for: A genomic survey of the fish parasite Spironucleus salmonicida indicates genomic plasticity among diplomonads and significant lateral gene transfer in eukaryote genome evolution
Source: BMC Genomics. 2007 Feb 14;8:51. doi: 10.1186/1471-2164-8-51 (PMC1805757; doi:10.1186/1471-2164-8-51)
Supplement: Additional file 2 — Complete list of gene annotations. [file 1471-2164-8-51-S2.pdf]

A genomic survey of the fish parasite *Spironucleus salmonicida* indicates genomic plasticity among diplomonads and significant lateral gene transfer in eukaryote genome evolution

Jan O. Andersson, Åsa M. Sjögren, David S. Horner, Colleen A. Murphy, Patricia L. Dyal, Staffan G. Svärd, John M. Logsdon, Jr., Mark A. Ragan, Robert P. Hirt, and Andrew J. Roger

Additional file 2

Genes identified in the *Spironucleus salmonicida* genome survey.

Clone	L_{aa}[1]	C[2]	TM[3]	LGT[4]	Protein	Classification
gSpZar1039gT3	164				synovial sarcoma, X breakpoint 2 interacting protein	Cellular Processes; Cell Communication; Adherens junction
SpESTDH110	200				cingulin	Cellular Processes; Cell Communication; Tight junction
SpESTC112	197				Rab GTPase	Cellular Processes; Cell Communication; Tight junction
SpESTC208	205				Rab GTPase	Cellular Processes; Cell Communication; Tight junction
SpESTC44	211				Rab GTPase	Cellular Processes; Cell Communication; Tight junction
SpESTC106	203				Rab GTPase	Cellular Processes; Cell Communication; Tight junction
SpESTZap199	73				Rab GTPase	Cellular Processes; Cell Communication; Tight junction
SpESTC67	197		TM		cAMP-dependent protein kinase regulator	Cellular Processes; Cell Growth and Death; Apoptosis
gSpZar984gT3	204				cell cycle arrest protein BUB2	Cellular Processes; Cell Growth and Death; Cell cycle
gSpZap189bT7	172				cell division control protein CDC54	Cellular Processes; Cell Growth and Death; Cell cycle
gSpTor126bT7	229				cyclin-dependent kinase	Cellular Processes; Cell Growth and Death; Cell cycle
gSpZap342bT7	134				cyclin-dependent kinase	Cellular Processes; Cell Growth and Death; Cell cycle
gSpZar649bT7	247				cyclin-dependent kinase	Cellular Processes; Cell Growth and Death; Cell cycle
SpESTZap1774	165				cyclin-dependent kinase 2	Cellular Processes; Cell Growth and Death; Cell cycle
gSpZar325gT3	205				minichromosome maintenance protein MCM2	Cellular Processes; Cell Growth and Death; Cell cycle
gSpZal621bMF	247				minichromosome maintenance protein MCM3	Cellular Processes; Cell Growth and Death; Cell cycle
gSpTor508bT7	165				ser/thr/tyr protein kinase RAD53	Cellular Processes; Cell Growth and Death; Cell cycle
SpESTC69	171				serine/threonine-protein kinase HSL1	Cellular Processes; Cell Growth and Death; Cell cycle
SpESTC88	218				ATP-binding protein involved in chromosome partitioning	Cellular Processes; Cell Growth and Death; Cell division
gSpTor587bT7	191				chromosome segregation protein	Cellular Processes; Cell Growth and Death; Cell division
gSpZap14gT3	192				chromosome segregation protein	Cellular Processes; Cell Growth and Death; Cell division
gSpZar1022bT7	204				chromosome segregation protein	Cellular Processes; Cell Growth and Death; Cell division
gSpZar333gT3	187				chromosome segregation protein	Cellular Processes; Cell Growth and Death; Cell division
gSpZar828gT3	234				chromosome segregation protein	Cellular Processes; Cell Growth and Death; Cell division
gSpTor651gT3	245				chromosome segregation protein	Cellular Processes; Cell Growth and Death; Cell division
SpESTZap1222	251				chromosome segregation protein	Cellular Processes; Cell Growth and Death; Cell division
SpESTZap839	209				chromosome segregation protein	Cellular Processes; Cell Growth and Death; Cell division
gSpZap25bT7	127				actin, beta/gamma, cytoplasmic	Cellular Processes; Cell Motility; Regulation of actin cytoskeleton
gSpTor118gT3	212				actinin, alpha	Cellular Processes; Cell Motility; Regulation of actin cytoskeleton
gSpTor1552bT7	214	C			integrin, beta	Cellular Processes; Cell Motility; Regulation of actin cytoskeleton
gSpTor874bT7_2	172	C			integrin, beta	Cellular Processes; Cell Motility; Regulation of actin cytoskeleton
gSpZap165gT3	178	C			integrin, beta	Cellular Processes; Cell Motility; Regulation of actin cytoskeleton
SpESTZap1171	210	C			integrin, beta	Cellular Processes; Cell Motility; Regulation of actin cytoskeleton
gSpZar519gT3	70				slingshot	Cellular Processes; Cell Motility; Regulation of actin cytoskeleton
gSpTol229bMF	253	C			gurken receptor precursor (torpedo)	Cellular Processes; Development; Dorso-ventral axis formation
SpESTDH444	187				adaptor protein complex medium subunit	Cellular Processes; unclassified
gSpTor930gT3_2	108				cyst wall protein	Cellular Processes; unclassified
gSpTol317bMF	207				cysteine protease	Cellular Processes; unclassified
gSpZar78bT7	110				cysteine protease	Cellular Processes; unclassified
SpESTC243	225				cysteine protease	Cellular Processes; unclassified
SpESTDH301	233				cysteine protease	Cellular Processes; unclassified
SpESTZap1687	216				dynactin	Cellular Processes; unclassified
Sp3orf4	746				dynamin-like protein	Cellular Processes; unclassified
gSpTol128bMF	178				dynein	Cellular Processes; unclassified
gSpTol141bMF	153				dynein	Cellular Processes; unclassified
gSpTor135gT3	230				dynein	Cellular Processes; unclassified
gSpTor169bT7	249				dynein	Cellular Processes; unclassified
gSpTor186bT7	235				dynein	Cellular Processes; unclassified
gSpTor186gT3	220				dynein	Cellular Processes; unclassified
gSpTor238gT3	210				dynein	Cellular Processes; unclassified
gSpTor253bT7	189				dynein	Cellular Processes; unclassified
gSpTor253gT3	235				dynein	Cellular Processes; unclassified
gSpTor569bT7	201				dynein	Cellular Processes; unclassified
gSpTor982bT7	215				dynein	Cellular Processes; unclassified
gSpZal387bMF	143				dynein	Cellular Processes; unclassified
gSpZap275bT7	97				dynein	Cellular Processes; unclassified
gSpZap451bT7	183				dynein	Cellular Processes; unclassified
gSpZap451gT3	160				dynein	Cellular Processes; unclassified
gSpZap513gT3	200				dynein	Cellular Processes; unclassified
gSpZap79bT7	164				dynein	Cellular Processes; unclassified
gSpZap806gT3	180				dynein	Cellular Processes; unclassified
gSpZar528gT3	198				dynein	Cellular Processes; unclassified
gSpZar684gT3	193				dynein	Cellular Processes; unclassified
SpESTC151	108				dynein	Cellular Processes; unclassified
SpESTC185	233				dynein	Cellular Processes; unclassified
gSpZap349gT3	197				guanine dissociation inhibitor (GDI)	Cellular Processes; unclassified
SpESTDH002	72				histone	Cellular Processes; unclassified
SpESTDH406	194				histone acetyltransferase	Cellular Processes; unclassified
SpESTC285	240				peptidylprolyl isomerase	Cellular Processes; unclassified
SpESTZap1298	230				phosphotyrosyl phosphatase activator protein	Cellular Processes; unclassified
1322213	395				tubulin	Cellular Processes; unclassified
SpESTC312	448				tubulin	Cellular Processes; unclassified
SpESTZap1216	190				tubulin	Cellular Processes; unclassified
SpESTZap1633	219				Tubulin tyrosine ligase	Cellular Processes; unclassified
SpESTC172	245				annexin	Environmental Information Processing
SpESTC206	293				annexin	Environmental Information Processing

SpESTC56	293				annexin	Environmental Information Processing
gSpZal608bMF	94				GTP-binding protein; COG0012 and pfam06071	Environmental Information Processing
gSpZap37gT3	200				GTP-binding protein	Environmental Information Processing
SpESTDH259	175				GTP-binding protein	Environmental Information Processing
gSpZal472bMF	111				protein C inhibitor	Environmental Information Processing
gSpZap444bT7	114				olfactory receptor	Environmental Information Processing; Ligand-Receptor Interaction; G-protein coupled receptors
gSpZap528bT7	43				olfactory receptor	Environmental Information Processing; Ligand-Receptor Interaction; G-protein coupled receptors
gSpZap596gT3	38				olfactory receptor	Environmental Information Processing; Ligand-Receptor Interaction; G-protein coupled receptors
SpESTC238	214				gamma-aminobutyric acid (GABA) A receptor, epsilon	Environmental Information Processing; Ligand-Receptor Interaction; Neuroactive ligand-receptor interaction
gSpTor556bT7	84		TM		Na+/H+ exchanger/antiporter; pfam00999	Environmental Information Processing; Membrane Transport
gSpZap219gT3	174				ABC-2 type transport system ATP-binding protein	Environmental Information Processing; Membrane Transport; ABC transporters, ABC-2 and other types
Sp1orf1	223		TM		ATP-binding cassette, subfamily C (CFTR/MRP), member 1	Environmental Information Processing; Membrane Transport; ABC transporters, eukaryotic
Sp1orf2	1514		TM		ATP-binding cassette, subfamily C (CFTR/MRP), member 5	Environmental Information Processing; Membrane Transport; ABC transporters, eukaryotic
gSpZar394bT7	78				simple sugar transport system ATP-binding protein	Environmental Information Processing; Membrane Transport; ABC transporters, prokaryotic
gSpZar824bT7	120				sulfonate/nitrate transport system ATP-binding protein	Environmental Information Processing; Membrane Transport; ABC transporters, prokaryotic
SpESTZap1389	238		TM	LGT	drug:H+ antiporter-2, DHA2 family	Environmental Information Processing; Membrane Transport; Major facilitator superfamily (MFS)
gSpTor1167gT3	132		TM		sugar porter, SP family	Environmental Information Processing; Membrane Transport; Major facilitator superfamily (MFS)
gSpTor978bT7	164		TM		Ca2+ transporting ATPase, plasma membrane	Environmental Information Processing; Signal Transduction; Calcium signaling pathway
gSpTor978gT3	161				Ca2+ transporting ATPase, plasma membrane	Environmental Information Processing; Signal Transduction; Calcium signaling pathway
gSpZap127bT7	183		TM		Ca2+ transporting ATPase, plasma membrane	Environmental Information Processing; Signal Transduction; Calcium signaling pathway
gSpTor39bT7	85				calmodulin	Environmental Information Processing; Signal Transduction; Calcium signaling pathway
Sp1orf5	147				Calmodulin	Environmental Information Processing; Signal Transduction; Calcium signaling pathway
SpESTZap862	171		TM		Calmodulin	Environmental Information Processing; Signal Transduction; Calcium signaling pathway
gSpTor927gT3_2	192				cyclin B	Environmental Information Processing; Signal Transduction; Calcium signaling pathway
SpESTZap933	152				cyclin-dependent kinase 1	Environmental Information Processing; Signal Transduction; Calcium signaling pathway
gSpZap949gT3	141				guanine nucleotide binding protein (G protein), alpha 15	Environmental Information Processing; Signal Transduction; Calcium signaling pathway
gSpZal266bMF	81				peptidylprolyl isomerase D (cyclophilin D)	Environmental Information Processing; Signal Transduction; Calcium signaling pathway
SpESTDH065	160				peptidylprolyl isomerase D (cyclophilin D)	Environmental Information Processing; Signal Transduction; Calcium signaling pathway
gSpZar353bT7	112				extracellular signal-regulated kinase	Environmental Information Processing; Signal Transduction; MAPK signaling pathway
SpESTDH126	240				extracellular signal-regulated kinase	Environmental Information Processing; Signal Transduction; MAPK signaling pathway
SpESTZap854	230				extracellular signal-regulated kinase	Environmental Information Processing; Signal Transduction; MAPK signaling pathway
gSpZar658bT7	189				inhibitor of kappa light polypeptide gene enhancer in B-cells,kinase	Environmental Information Processing; Signal Transduction; MAPK signaling pathway
SpESTZap869	183				misshapen/NIK-related kinase	Environmental Information Processing; Signal Transduction; MAPK signaling pathway
SpESTZap978	241				misshapen/NIK-related kinase	Environmental Information Processing; Signal Transduction; MAPK signaling pathway
gSpZap536bT7	112				mitogen and stress response kinase 1	Environmental Information Processing; Signal Transduction; MAPK signaling pathway
gSpZap1011gT3	138				mitogen-,stress activated protein kinases	Environmental Information Processing; Signal Transduction; MAPK signaling pathway
gSpZar938bT7	208				mitogen-,stress activated protein kinases	Environmental Information Processing; Signal Transduction; MAPK signaling pathway
gSpZar494gT3	200				mitogen-activated protein kinase kinase kinase 13	Environmental Information Processing; Signal Transduction; MAPK signaling pathway
gSpTor797bT7	132				mitogen-activated protein kinase kinase kinase 2	Environmental Information Processing; Signal Transduction; MAPK signaling pathway
gSpTor951gT3_2	235				mitogen-activated protein kinase kinase kinase 2	Environmental Information Processing; Signal Transduction; MAPK signaling pathway
gSpTor707gT3	162				mitogen-activated protein kinase kinase kinase 9	Environmental Information Processing; Signal Transduction; MAPK signaling pathway
gSpZar667gT3	173				mitogen-activated protein kinase kinase kinase kinase 1	Environmental Information Processing; Signal Transduction; MAPK signaling pathway
gSpTor1559bT7	132				p21-activated kinase 1	Environmental Information Processing; Signal Transduction; MAPK signaling pathway
SpESTDH364	181				p21-activated kinase 1	Environmental Information Processing; Signal Transduction; MAPK signaling pathway
gSpZar828bT7	133				p38 MAP kinase	Environmental Information Processing; Signal Transduction; MAPK signaling pathway
gSpZar201bT7	146		TM		p90 ribosomal S6 kinase	Environmental Information Processing; Signal Transduction; MAPK signaling pathway
gSpZap793bT7	151				protein kinase A	Environmental Information Processing; Signal Transduction; MAPK signaling pathway
gSpZap816gT3	108				protein kinase A	Environmental Information Processing; Signal Transduction; MAPK signaling pathway
SpESTZap368	189				protein kinase A	Environmental Information Processing; Signal Transduction; MAPK signaling pathway
SpESTZap819	188				protein kinase C	Environmental Information Processing; Signal Transduction; MAPK signaling pathway
gSpZap837bT7	153				protein phosphatase 1A (formerly 2C)	Environmental Information Processing; Signal Transduction; MAPK signaling pathway
SpESTC175	232				protein phosphatase 1A (formerly 2C)	Environmental Information Processing; Signal Transduction; MAPK signaling pathway
gSpZap256gT3	121				protein phosphatase 1B (formerly 2C)	Environmental Information Processing; Signal Transduction; MAPK signaling pathway
SpESTC153	111				protein phosphatase 1B (formerly 2C)	Environmental Information Processing; Signal Transduction; MAPK signaling pathway
gSpZar560gT3	236				protein phosphatase 5	Environmental Information Processing; Signal Transduction; MAPK signaling pathway
SpESTC209	215				protein phosphatase 5	Environmental Information Processing; Signal Transduction; MAPK signaling pathway
SpESTC270	367				protein phosphatase 5	Environmental Information Processing; Signal Transduction; MAPK signaling pathway
SpESTZap1875	214				protein phosphatase 5	Environmental Information Processing; Signal Transduction; MAPK signaling pathway
SpESTZap191	161				protein phosphatase 5	Environmental Information Processing; Signal Transduction; MAPK signaling pathway
SpESTZap54	225				protein phosphatase 5	Environmental Information Processing; Signal Transduction; MAPK signaling pathway
gSpZar609bT7	149				RAP1, member of RAS oncogene family	Environmental Information Processing; Signal Transduction; MAPK signaling pathway
SpESTC180	195				Rho superfamily, small GTP binding protein Rac	Environmental Information Processing; Signal Transduction; MAPK signaling pathway
SpESTZap52	208				serine/threonine kinase 4	Environmental Information Processing; Signal Transduction; MAPK signaling pathway
gSpZap77gT3	196	C			tumor necrosis factor receptor superfamily, member 1A	Environmental Information Processing; Signal Transduction; MAPK signaling pathway
gSpZap289bT7	105				v-akt murine thymoma viral oncogene homolog	Environmental Information Processing; Signal Transduction; MAPK signaling pathway
gSpTor570bT7	222	C			jagged	Environmental Information Processing; Signal Transduction; Notch signaling pathway
gSpTor757bT7	125	C			Notch	Environmental Information Processing; Signal Transduction; Notch signaling pathway
gSpTor957gT3_2	223	C			Notch	Environmental Information Processing; Signal Transduction; Notch signaling pathway
gSpZap443bT7	117	C			Notch	Environmental Information Processing; Signal Transduction; Notch signaling pathway
gSpZap601gT3	148	C			Notch	Environmental Information Processing; Signal Transduction; Notch signaling pathway
gSpZar633gT3	210	C			Notch	Environmental Information Processing; Signal Transduction; Notch signaling pathway
SpESTC104	226	C			Notch	Environmental Information Processing; Signal Transduction; Notch signaling pathway
SpESTZap901	190	C	TM		Notch	Environmental Information Processing; Signal Transduction; Notch signaling pathway
gSpZap337gT3	175				p300/CBP-associated factor	Environmental Information Processing; Signal Transduction; Notch signaling pathway
gSpZar604bT7	92				p300/CBP-associated factor	Environmental Information Processing; Signal Transduction; Notch signaling pathway
gSpTor764bT7	142				type III PIP kinase	Environmental Information Processing; Signal Transduction; Phosphatidylinositol signaling system
gSpZap650gT3	219				casein kinase 1	Environmental Information Processing; Signal Transduction; Wnt signaling pathway
SpESTZap333	244				casein kinase 1	Environmental Information Processing; Signal Transduction; Wnt signaling pathway
gSpTor256gT3	115				glycogen synthase kinase 3 beta	Environmental Information Processing; Signal Transduction; Wnt signaling pathway
gSpZar932bT7	194				glycogen synthase kinase 3 beta	Environmental Information Processing; Signal Transduction; Wnt signaling pathway
gSpTor88bT7	76				protein phosphatase 2 (formerly 2A), catalytic subunit	Environmental Information Processing; Signal Transduction; Wnt signaling pathway
gSpTor951bT7_2	92				protein phosphatase 2 (formerly 2A), catalytic subunit	Environmental Information Processing; Signal Transduction; Wnt signaling pathway
SpESTC129	194				protein phosphatase 2 (formerly 2A), catalytic subunit	Environmental Information Processing; Signal Transduction; Wnt signaling pathway
SpESTC201	235				protein phosphatase 2 (formerly 2A), catalytic subunit	Environmental Information Processing; Signal Transduction; Wnt signaling pathway
SpESTDH019	106				protein phosphatase 2 (formerly 2A), catalytic	Environmental Information Processing; Signal Transduction; Wnt signaling pathway

				subunit	
SpESTZap135	130			protein phosphatase 2 (formerly 2A), catalytic subunit	Environmental Information Processing; Signal Transduction; Wnt signaling pathway
SpESTZap1619	220			protein phosphatase 2 (formerly 2A), catalytic subunit	Environmental Information Processing; Signal Transduction; Wnt signaling pathway
gSpZap320gT3	83			protein phosphatase 2 (formerly 2A), regulatory subunit B	Environmental Information Processing; Signal Transduction; Wnt signaling pathway
gSpTor487bT7	184			Rho-associated, coiled-coil containing protein kinase	Environmental Information Processing; Signal Transduction; Wnt signaling pathway
SpESTC234	289			RuvB-like protein 1 (pontin 52)	Environmental Information Processing; Signal Transduction; Wnt signaling pathway
gSpZap222bT7	132			hect domain ubiquitin protein ligase E3 component	Genetic Information Processing; Folding, Sorting and Degradation; Genetic Information Processing; Folding, Sorting and Degradation
gSpZap912bT7	91			hect domain ubiquitin protein ligase E3 component	Genetic Information Processing; Folding, Sorting and Degradation; Genetic Information Processing; Folding, Sorting and Degradation
SpESTZap2092	172			hect domain ubiquitin protein ligase E3 component	Genetic Information Processing; Folding, Sorting and Degradation; Genetic Information Processing; Folding, Sorting and Degradation
SpESTZap279	202			20S proteasome subunit alpha1	Genetic Information Processing; Folding, Sorting and Degradation; Proteasome
SpESTC101	170			20S proteasome subunit alpha3	Genetic Information Processing; Folding, Sorting and Degradation; Proteasome
SpESTZap1656	169			20S proteasome subunit alpha7	Genetic Information Processing; Folding, Sorting and Degradation; Proteasome
gSpTor2gT3	100			20S proteasome subunit beta1	Genetic Information Processing; Folding, Sorting and Degradation; Proteasome
SpESTC215	228			20S proteasome subunit beta5	Genetic Information Processing; Folding, Sorting and Degradation; Proteasome
SpESTC166	188			20S proteasome subunit beta6	Genetic Information Processing; Folding, Sorting and Degradation; Proteasome
SpESTZap889	129			20S proteasome subunit beta7	Genetic Information Processing; Folding, Sorting and Degradation; Proteasome
SpESTC119	165			26S proteasome regulatory subunit N11	Genetic Information Processing; Folding, Sorting and Degradation; Proteasome
SpESTC146	209			26S proteasome regulatory subunit N8	Genetic Information Processing; Folding, Sorting and Degradation; Proteasome
SpESTZap1256	211			26S proteasome regulatory subunit T2	Genetic Information Processing; Folding, Sorting and Degradation; Proteasome
gSpZap532gT3	88			26S proteasome regulatory subunit T4	Genetic Information Processing; Folding, Sorting and Degradation; Proteasome
SpESTC107	233			26S proteasome regulatory subunit T5	Genetic Information Processing; Folding, Sorting and Degradation; Proteasome
gSpTor154bT7	159			26S proteasome regulatory subunit T6	Genetic Information Processing; Folding, Sorting and Degradation; Proteasome
gSpZap115gT3	176			proteasome alpha subunit	Genetic Information Processing; Folding, Sorting and Degradation; Proteasome
gSpZap497gT3	166			Mg-dependent DNase	Genetic Information Processing; Folding, Sorting and Degradation; Protein export
SpESTZap2118	155	TM		preprotein translocase SecY subunit	Genetic Information Processing; Folding, Sorting and Degradation; Protein export
SpESTZap2097	94			signal recognition particle, subunit SRP19	Genetic Information Processing; Folding, Sorting and Degradation; Protein export
gSpTor27gT3	139			ATP-dependent Zn protease, cell division protein	Genetic Information Processing; Folding, Sorting and Degradation; Protein folding and associated processing
15990567	512			chaperonin GroEL (Hsp60)	Genetic Information Processing; Folding, Sorting and Degradation; Protein folding and associated processing
gSpZar636gT3	130			chaperonin GroEL (Hsp60)	Genetic Information Processing; Folding, Sorting and Degradation; Protein folding and associated processing
SpESTC121	201			chaperonin GroEL (Hsp60)	Genetic Information Processing; Folding, Sorting and Degradation; Protein folding and associated processing
SpESTC170	209			chaperonin GroEL (Hsp60)	Genetic Information Processing; Folding, Sorting and Degradation; Protein folding and associated processing
SpESTC73	341			chaperonin GroEL (Hsp60)	Genetic Information Processing; Folding, Sorting and Degradation; Protein folding and associated processing
SpESTDH392	191			chaperonin GroEL (Hsp60)	Genetic Information Processing; Folding, Sorting and Degradation; Protein folding and associated processing
SpESTZap392	225			chaperonin GroEL (Hsp60)	Genetic Information Processing; Folding, Sorting and Degradation; Protein folding and associated processing
23306653	657			heat shock protein 70, Hsp70 family	Genetic Information Processing; Folding, Sorting and Degradation; Protein folding and associated processing
gSpZal522bMF	164			heat shock protein 70, Hsp70 family	Genetic Information Processing; Folding, Sorting and Degradation; Protein folding and associated processing
SpESTC184	221			heat shock protein 70, Hsp70 family	Genetic Information Processing; Folding, Sorting and Degradation; Protein folding and associated processing
SpESTC236	230			heat shock protein 70, Hsp70 family	Genetic Information Processing; Folding, Sorting and Degradation; Protein folding and associated processing
SpESTZap1064	205			heat shock protein 70, Hsp70 family	Genetic Information Processing; Folding, Sorting and Degradation; Protein folding and associated processing
SpESTC148	303			Heat shock protein 90; pfam00183	Genetic Information Processing; Folding, Sorting and Degradation; Protein folding and associated processing
SpESTC18	183		LGT	membrane protease subunit HflK	Genetic Information Processing; Folding, Sorting and Degradation; Protein folding and associated processing
gSpZar958bT7	125		LGT	membrane protease subunit HflK	Genetic Information Processing; Folding, Sorting and Degradation; Protein folding and associated processing
gSpZap739gT3	111			prefoldin alpha subunit	Genetic Information Processing; Folding, Sorting and Degradation; Protein folding and associated processing
SpESTC227	228			protein disulfide-isomerase	Genetic Information Processing; Folding, Sorting and Degradation; Protein folding and associated processing
SpESTC258	220			protein disulfide-isomerase	Genetic Information Processing; Folding, Sorting and Degradation; Protein folding and associated processing
SpESTZap295	92			protein disulfide-isomerase	Genetic Information Processing; Folding, Sorting and Degradation; Protein folding and associated processing
SpESTZap885	95			protein disulfide-isomerase	Genetic Information Processing; Folding, Sorting and Degradation; Protein folding and associated processing
SpESTZap970	223			ubiquilin 1	Genetic Information Processing; Folding, Sorting and Degradation; Ubiquitin mediated proteolysis
gSpZar929bT7	165			ubiquitin-activating enzyme E1	Genetic Information Processing; Folding, Sorting and Degradation; Ubiquitin mediated proteolysis
gSpTor755bT7	81			ubiquitin-conjugating enzyme	Genetic Information Processing; Folding, Sorting and Degradation; Ubiquitin mediated proteolysis
SpESTDH402	166			ubiquitin-conjugating enzyme	Genetic Information Processing; Folding, Sorting and Degradation; Ubiquitin mediated proteolysis
SpESTZap86	125			ubiquitin-conjugating enzyme	Genetic Information Processing; Folding, Sorting and Degradation; Ubiquitin mediated proteolysis
gSpZap808gT3	130			ubiquitin-conjugating enzyme E2	Genetic Information Processing; Folding, Sorting and Degradation; Ubiquitin mediated proteolysis
gSpZap913gT3	102			ubiquitin-conjugating enzyme E2	Genetic Information Processing; Folding, Sorting and Degradation; Ubiquitin mediated proteolysis
gSpTol292bMF	186			DNA polymerase	Genetic Information Processing; Replication and Repair; DNA polymerase
SpESTDH269	187			DNA polymerase	Genetic Information Processing; Replication and Repair; DNA polymerase
gSpZap446bT7	163			DNA polymerase alpha, subunit A	Genetic Information Processing; Replication and Repair; DNA polymerase
gSpZap813gT3	197			DNA polymerase delta, subunit A	Genetic Information Processing; Replication and Repair; DNA polymerase
gSpZap553gT3	47	C		DNA polymerase IV	Genetic Information Processing; Replication and Repair; DNA polymerase
gSpZap842bT7	83			ATP-dependent DNA helicase RecQ	Genetic Information Processing; Replication and Repair; Other replication, recombination and repair factors
SpESTC164	225			ATP-dependent RNA helicase DeaD	Genetic Information Processing; Replication and Repair; Other replication, recombination and repair factors
SpESTZap741	217			ATP-dependent RNA helicase DeaD	Genetic Information Processing; Replication and Repair; Other replication, recombination and repair factors
gSpTor801bT7	152			ATP-dependent RNA helicase RhlB	Genetic Information Processing; Replication and Repair; Other replication, recombination and repair factors
gSpTor162gT3	185			ATP-dependent RNA helicase SrmB	Genetic Information Processing; Replication and Repair; Other replication, recombination and repair factors
SpESTZap1984	120			ATP-independent RNA helicase DbpA	Genetic Information Processing; Replication and Repair; Other replication, recombination and repair factors
SpESTC43	188		LGT	DNA glycosylase	Genetic Information Processing; Replication and Repair; Other replication, recombination

					and repair factors
gSpZap869bT7	149			DNA mismatch repair protein MutS	Genetic Information Processing; Replication and Repair; Other replication, recombination and repair factors
gSpZar426bT7	126			DNA repair protein Rad51	Genetic Information Processing; Replication and Repair; Other replication, recombination and repair factors
SpESTDH274	130			DNA-(apurinic or apyrimidinic site) lyase	Genetic Information Processing; Replication and Repair; Other replication, recombination and repair factors
gSpZal070bMF	156			exodeoxyribonuclease I	Genetic Information Processing; Replication and Repair; Other replication, recombination and repair factors
SpESTZap438	181			exodeoxyribonuclease III	Genetic Information Processing; Replication and Repair; Other replication, recombination and repair factors
gSpTor235gT3	214			exonuclease SbcC	Genetic Information Processing; Replication and Repair; Other replication, recombination and repair factors
SpESTC237	234			exonuclease SbcC	Genetic Information Processing; Replication and Repair; Other replication, recombination and repair factors
SpESTZap1967	102			flap endonuclease-1	Genetic Information Processing; Replication and Repair; Other replication, recombination and repair factors
gSpTor13gT3	195		TM	helicase	Genetic Information Processing; Replication and Repair; Other replication, recombination and repair factors
gSpZar813gT3	226			DNA topoisomerase II	Genetic Information Processing; Replication and Repair; Replication complex
gSpZap319gT3	109			transcription initiation factor TFIID subunit D4	Genetic Information Processing; Transcription; Basal transcription factors
gSpZap288bT7	167			DNA-directed RNA polymerase I subunit A1	Genetic Information Processing; Transcription; RNA polymerase
gSpZar356gT3	122			DNA-directed RNA polymerase I subunit A2	Genetic Information Processing; Transcription; RNA polymerase
gSpZal444bMF	121			DNA-directed RNA polymerase II subunit A	Genetic Information Processing; Transcription; RNA polymerase
gSpTor153bT7	222			DNA-directed RNA polymerase II subunit B	Genetic Information Processing; Transcription; RNA polymerase
gSpTor153gT3	120			DNA-directed RNA polymerase II subunit B	Genetic Information Processing; Transcription; RNA polymerase
gSpZar943bT7	108			DNA-directed RNA polymerase II subunit J	Genetic Information Processing; Transcription; RNA polymerase
gSpTor141bT7	231			DNA-directed RNA polymerase III subunit C1	Genetic Information Processing; Transcription; RNA polymerase
gSpTor141gT3	227			DNA-directed RNA polymerase III subunit C1	Genetic Information Processing; Transcription; RNA polymerase
gSpZap239bT7	162			DNA-directed RNA Polymerase III subunit C5	Genetic Information Processing; Transcription; RNA polymerase
gSpTor660gT3	69			DNA-directed RNA polymerase subunit N	Genetic Information Processing; Transcription; RNA polymerase
gSpZar821gT3	229			tRNA-dihydrouridine synthase 3	Genetic Information Processing; Translation; Other translation factors
SpESTZap366	197			tRNA-dihydrouridine synthase A	Genetic Information Processing; Translation; Other translation factors
SpESTC286	214			large subunit ribosomal protein L10Ae	Genetic Information Processing; Translation; Ribosome
SpESTC287	204			large subunit ribosomal protein L10e	Genetic Information Processing; Translation; Ribosome
SpESTC303	169			large subunit ribosomal protein L11e	Genetic Information Processing; Translation; Ribosome
SpESTC283	177			large subunit ribosomal protein L12e	Genetic Information Processing; Translation; Ribosome
SpESTC296	191			large subunit ribosomal protein L13Ae	Genetic Information Processing; Translation; Ribosome
SpESTZap1021	192			large subunit ribosomal protein L13e	Genetic Information Processing; Translation; Ribosome
SpESTC250	124			large subunit ribosomal protein L14e	Genetic Information Processing; Translation; Ribosome
SpESTC302	201			large subunit ribosomal protein L15e	Genetic Information Processing; Translation; Ribosome
SpESTC229	162			large subunit ribosomal protein L17e	Genetic Information Processing; Translation; Ribosome
SpESTC187	167			large subunit ribosomal protein L18Ae	Genetic Information Processing; Translation; Ribosome
SpESTC291	178			large subunit ribosomal protein L18e	Genetic Information Processing; Translation; Ribosome
SpESTC256	175			large subunit ribosomal protein L19e	Genetic Information Processing; Translation; Ribosome
SpESTC244	156			large subunit ribosomal protein L21e	Genetic Information Processing; Translation; Ribosome
SpESTC168	110			large subunit ribosomal protein L22e	Genetic Information Processing; Translation; Ribosome
SpESTC68	133			large subunit ribosomal protein L23e	Genetic Information Processing; Translation; Ribosome
SpESTZap1971	126			large subunit ribosomal protein L24	Genetic Information Processing; Translation; Ribosome
SpESTDH056	95			large subunit ribosomal protein L24e	Genetic Information Processing; Translation; Ribosome
SpESTC15	145			large subunit ribosomal protein L27Ae	Genetic Information Processing; Translation; Ribosome
SpESTC95	124			large subunit ribosomal protein L27e	Genetic Information Processing; Translation; Ribosome
SpESTC297	106			large subunit ribosomal protein L30e	Genetic Information Processing; Translation; Ribosome
SpESTC232	100			large subunit ribosomal protein L31e	Genetic Information Processing; Translation; Ribosome
SpESTC194	130			large subunit ribosomal protein L32e	Genetic Information Processing; Translation; Ribosome
SpESTC160	110			large subunit ribosomal protein L34e	Genetic Information Processing; Translation; Ribosome
SpESTZap1267	89			large subunit ribosomal protein L35Ae	Genetic Information Processing; Translation; Ribosome
SpESTC310	127			large subunit ribosomal protein L35e	Genetic Information Processing; Translation; Ribosome
SpESTC220	86			large subunit ribosomal protein L37Ae	Genetic Information Processing; Translation; Ribosome
SpESTZap1647	76			large subunit ribosomal protein L37e	Genetic Information Processing; Translation; Ribosome
SpESTZap673	111			large subunit ribosomal protein L3e	Genetic Information Processing; Translation; Ribosome
SpESTC306	126			large subunit ribosomal protein L40e	Genetic Information Processing; Translation; Ribosome
SpESTC274	104			large subunit ribosomal protein L44e	Genetic Information Processing; Translation; Ribosome
SpESTC309	109			large subunit ribosomal protein L44e	Genetic Information Processing; Translation; Ribosome
SpESTC131	207			large subunit ribosomal protein L4e	Genetic Information Processing; Translation; Ribosome
SpESTC290	240			large subunit ribosomal protein L5e	Genetic Information Processing; Translation; Ribosome
gSpTor643gT3	121			large subunit ribosomal protein L7Ae	Genetic Information Processing; Translation; Ribosome
SpESTC314	227			large subunit ribosomal protein L7Ae	Genetic Information Processing; Translation; Ribosome
SpESTZap830	116		TM	large subunit ribosomal protein L7Ae	Genetic Information Processing; Translation; Ribosome
SpESTC216	210			large subunit ribosomal protein L7e	Genetic Information Processing; Translation; Ribosome
SpESTC163	164			large subunit ribosomal protein L8e	Genetic Information Processing; Translation; Ribosome
SpESTC20	133			large subunit ribosomal protein L9e	Genetic Information Processing; Translation; Ribosome
SpESTZap665	171			large subunit ribosomal protein L9e	Genetic Information Processing; Translation; Ribosome
SpESTC304	244		TM	large subunit ribosomal protein LP0	Genetic Information Processing; Translation; Ribosome
SpESTC205	103			large subunit ribosomal protein LP1	Genetic Information Processing; Translation; Ribosome
SpESTZap844	118			small subunit ribosomal protein S10e	Genetic Information Processing; Translation; Ribosome
SpESTC277	148			small subunit ribosomal protein S11e	Genetic Information Processing; Translation; Ribosome
SpESTZap1447	119			small subunit ribosomal protein S12e	Genetic Information Processing; Translation; Ribosome
SpESTC262	152			small subunit ribosomal protein S13e	Genetic Information Processing; Translation; Ribosome
SpESTZap1202	136			small subunit ribosomal protein S14e	Genetic Information Processing; Translation; Ribosome
SpESTC313	127			small subunit ribosomal protein S15Ae	Genetic Information Processing; Translation; Ribosome
SpESTC299	133			small subunit ribosomal protein S16e	Genetic Information Processing; Translation; Ribosome
SpESTC288	113			small subunit ribosomal protein S17e	Genetic Information Processing; Translation; Ribosome
SpESTC292	150			small subunit ribosomal protein S18e	Genetic Information Processing; Translation; Ribosome
SpESTC240	143			small subunit ribosomal protein S19	Genetic Information Processing; Translation; Ribosome
SpESTC183	131			small subunit ribosomal protein S19e	Genetic Information Processing; Translation; Ribosome
SpESTZap828	107			small subunit ribosomal protein S20e	Genetic Information Processing; Translation; Ribosome
SpESTDH098	74			small subunit ribosomal protein S21e	Genetic Information Processing; Translation; Ribosome
SpESTC294	139			small subunit ribosomal protein S23e	Genetic Information Processing; Translation; Ribosome
SpESTC241	126			small subunit ribosomal protein S24e	Genetic Information Processing; Translation; Ribosome
SpESTC211	91			small subunit ribosomal protein S25e	Genetic Information Processing; Translation; Ribosome
SpESTC264	100			small subunit ribosomal protein S26e	Genetic Information Processing; Translation; Ribosome
SpESTC218	130			small subunit ribosomal protein S27Ae	Genetic Information Processing; Translation; Ribosome
SpESTC224	77			small subunit ribosomal protein S27e	Genetic Information Processing; Translation; Ribosome
SpESTC193	63			small subunit ribosomal protein S28e	Genetic Information Processing; Translation; Ribosome
SpESTC62	47	C		small subunit ribosomal protein S29e	Genetic Information Processing; Translation; Ribosome
SpESTC279	230			small subunit ribosomal protein S2e	Genetic Information Processing; Translation; Ribosome
SpESTZap358	74			small subunit ribosomal protein S30e	Genetic Information Processing; Translation; Ribosome
SpESTC284	240			small subunit ribosomal protein S3Ae	Genetic Information Processing; Translation; Ribosome
SpESTC308	204			small subunit ribosomal protein S3e	Genetic Information Processing; Translation; Ribosome
SpESTC255	210			small subunit ribosomal protein S4e	Genetic Information Processing; Translation; Ribosome
SpESTC298	188			small subunit ribosomal protein S5e	Genetic Information Processing; Translation; Ribosome
SpESTC272	225			small subunit ribosomal protein S6e	Genetic Information Processing; Translation; Ribosome
SpESTDH084	138			small subunit ribosomal protein S7e	Genetic Information Processing; Translation; Ribosome
SpESTC278	167			small subunit ribosomal protein S8e	Genetic Information Processing; Translation; Ribosome
SpESTC231	165			small subunit ribosomal protein S9e	Genetic Information Processing; Translation; Ribosome
SpESTC311	224			small subunit ribosomal protein SAe	Genetic Information Processing; Translation; Ribosome

gSpTor722bT7	143			elongation factor EF-1 alpha subunit	Genetic Information Processing; Translation; Translation factors
gSpZar859bT7	68			elongation factor EF-1 alpha subunit	Genetic Information Processing; Translation; Translation factors
SpESTC305	435			elongation factor EF-1 alpha subunit	Genetic Information Processing; Translation; Translation factors
SpESTZap1479	234			elongation factor EF-1 alpha subunit	Genetic Information Processing; Translation; Translation factors
32816837	224			elongation factor EF-1 beta subunit	Genetic Information Processing; Translation; Translation factors
SpESTDH009	104			elongation factor EF-2	Genetic Information Processing; Translation; Translation factors
gSpZap36gT3	148			peptide chain release factor eRF subunit 1	Genetic Information Processing; Translation; Translation factors
SpESTZap940	218			translation initiation factor eIF-2 beta subunit	Genetic Information Processing; Translation; Translation factors
gSpZap92bT7	179			translation initiation factor eIF-2 gamma subunit	Genetic Information Processing; Translation; Translation factors
gSpTor822bT7_2	220			translation initiation factor eIF-2B epsilon subunit	Genetic Information Processing; Translation; Translation factors
SpESTC57	185			translation initiation factor eIF-3 subunit 2	Genetic Information Processing; Translation; Translation factors
gSpTor973gT3	105			translation initiation factor eIF-4A	Genetic Information Processing; Translation; Translation factors
gSpZap278bT7	141			translation initiation factor eIF-4A	Genetic Information Processing; Translation; Translation factors
gSpZap373gT3	191			translation initiation factor eIF-4A	Genetic Information Processing; Translation; Translation factors
SpESTC84	235			translation initiation factor eIF-4A	Genetic Information Processing; Translation; Translation factors
SpESTZap768	228			translation initiation factor eIF-4A	Genetic Information Processing; Translation; Translation factors
SpESTC301	148			translation initiation factor eIF-5A	Genetic Information Processing; Translation; Translation factors
gSpZap490gT3	91			translation initiation factor eIF-6	Genetic Information Processing; Translation; Translation factors
gSpTor308bT7	168			translation initiation factor IF-2	Genetic Information Processing; Translation; Translation factors
gSpTor587gT3	239			translation initiation factor IF-2 unclassified subunit	Genetic Information Processing; Translation; Translation factors
gSpZap790gT3	160			CCTbeta	Genetic Information Processing; unclassified
SpESTZap1495	230			CCTtheta	Genetic Information Processing; unclassified
gSpZar107bT7	111			clathrin	Genetic Information Processing; unclassified
34148554	326			coatomer-like protein beta subunit	Genetic Information Processing; unclassified
SpESTZap1250	145			exoribonuclease	Genetic Information Processing; unclassified
gSpTor1287bT7	135			N2,N2-dimethylguanosine tRNA methyltransferase	Genetic Information Processing; unclassified
gSpZap780gT3	136			Ribosomal RNA adenine dimethylase	Genetic Information Processing; unclassified
gSpTor397gT3	214			snoRNA binding protein	Genetic Information Processing; unclassified
SpESTZap1982	222			snoRNA binding protein	Genetic Information Processing; unclassified
SpESTZap276	247	TM		sulfurase	Genetic Information Processing; unclassified
SpESTZap1340	107		LGT	tRNA (5-methylaminomethyl-2-thiouridylate) -methyltransferase	Genetic Information Processing; unclassified
34148556	174			vacuolar protein sorting 26-like	Genetic Information Processing; unclassified
27982008	623		LGT	alanyl-tRNA synthetase	Metabolism; Amino Acid Metabolism; Alanine and aspartate Metabolism
SpESTDH287	214		LGT	aminoacylhistidine dipeptidase	Metabolism; Amino Acid Metabolism; Alanine and aspartate Metabolism
SpESTZap149	259		LGT	aminoacylhistidine dipeptidase	Metabolism; Amino Acid Metabolism; Alanine and aspartate Metabolism
SpESTC149	412		LGT	aminoacylhistidine dipeptidase	Metabolism; Amino Acid Metabolism; Alanine and aspartate Metabolism
SpESTC143	444			asparaginyl-tRNA synthetase	Metabolism; Amino Acid Metabolism; Alanine and aspartate Metabolism
SpESTC26	192			aspartyl-tRNA synthetase	Metabolism; Amino Acid Metabolism; Alanine and aspartate Metabolism
SpESTC31	172		LGT	5'-methylthioadenosine nucleosidase	Metabolism; Amino Acid Metabolism; Arginine and proline Metabolism
SpESTC83	385		LGT	arginine deiminase	Metabolism; Amino Acid Metabolism; Arginine and proline Metabolism
SpESTC221	314		LGT	ornithine carbamoyltransferase	Metabolism; Amino Acid Metabolism; Arginine and proline Metabolism
gSpZar652bT7	94			ornithine decarboxylase	Metabolism; Amino Acid Metabolism; Arginine and proline Metabolism
27981826	299		LGT	prolyl-tRNA synthetase	Metabolism; Amino Acid Metabolism; Arginine and proline Metabolism
gSpTol076bMF	117			cysteinyl-tRNA synthetase	Metabolism; Amino Acid Metabolism; Cysteine Metabolism
gSpTor417bT7	243			glutaminyl-tRNA synthetase	Metabolism; Amino Acid Metabolism; Glutamate Metabolism
SpESTDH021	95			glycyl-tRNA synthetase, class II	Metabolism; Amino Acid Metabolism; Glycine, serine and threonine Metabolism
gSpZap391gT3	101	TM		L-serine dehydratase	Metabolism; Amino Acid Metabolism; Glycine, serine and threonine Metabolism
gSpZar602gT3	218		LGT	sarcosine oxidase	Metabolism; Amino Acid Metabolism; Glycine, serine and threonine Metabolism
gSpZap30gT3	172		LGT	sarcosine oxidase	Metabolism; Amino Acid Metabolism; Glycine, serine and threonine Metabolism
gSpZar953bT7	62			sarcosine oxidase	Metabolism; Amino Acid Metabolism; Glycine, serine and threonine Metabolism
gSpZar496gT3	78			seryl-tRNA synthetase	Metabolism; Amino Acid Metabolism; Glycine, serine and threonine Metabolism
SpESTDH141	104			seryl-tRNA synthetase	Metabolism; Amino Acid Metabolism; Glycine, serine and threonine Metabolism
27982357	303		LGT	threonine dehydratase	Metabolism; Amino Acid Metabolism; Glycine, serine and threonine Metabolism
gSpTor190gT3	223			threonyl-tRNA synthetase	Metabolism; Amino Acid Metabolism; Glycine, serine and threonine Metabolism
SpESTZap946	122		LGT	histidinol-phosphatase (PHP family)	Metabolism; Amino Acid Metabolism; Histidine Metabolism
gSpZap859bT7	183			histidyl-tRNA synthetase	Metabolism; Amino Acid Metabolism; Histidine Metabolism
gSpZar416gT3	174			histidyl-tRNA synthetase	Metabolism; Amino Acid Metabolism; Histidine Metabolism
gSpZap345gT3	178			lysyl-tRNA synthetase, class II	Metabolism; Amino Acid Metabolism; Lysine biosynthesis
SpESTC19	205			lysyl-tRNA synthetase, class II	Metabolism; Amino Acid Metabolism; Lysine biosynthesis
Sp7orf3	480			histone-lysine N-methyltransferase	Metabolism; Amino Acid Metabolism; Lysine degradation
gSpZap292gT3	159	TM		methionyl-tRNA synthetase	Metabolism; Amino Acid Metabolism; Methionine Metabolism
SpESTZap947	94			methionyl-tRNA synthetase	Metabolism; Amino Acid Metabolism; Methionine Metabolism
SpESTZap902	196			tyrosyl-tRNA synthetase	Metabolism; Amino Acid Metabolism; Phenylalanine, tyrosine and tryptophan biosynthesis
gSpZar1005bT7	100			tryptophanyl-tRNA synthetase	Metabolism; Amino Acid Metabolism; Tryptophan Metabolism
SpESTZap1526	182			tryptophanyl-tRNA synthetase	Metabolism; Amino Acid Metabolism; Tryptophan Metabolism
SpESTC86	198			alanine aminotransferase - aspartate/tyrosine/aromatic aminotransferase; COG0436	Metabolism; Amino Acid Metabolism; unclassified
gSpTor628bT7	200			isoleucyl-tRNA synthetase	Metabolism; Amino Acid Metabolism; Valine, leucine and isoleucine biosynthesis
SpESTZap2033	183			leucyl-tRNA synthetase	Metabolism; Amino Acid Metabolism; Valine, leucine and isoleucine biosynthesis
27982171	352			branched-chain amino acid aminotransferase	Metabolism; Amino Acid Metabolism; Valine, leucine and isoleucine degradation
gSpZar678bT7	154			branched-chain amino acid aminotransferase	Metabolism; Amino Acid Metabolism; Valine, leucine and isoleucine degradation
27982840	124			glucosamine-6-phosphate isomerase	Metabolism; Carbohydrate Metabolism; Aminosugars Metabolism
27983037	234			glucosamine-6-phosphate isomerase	Metabolism; Carbohydrate Metabolism; Aminosugars Metabolism
gSpTor1448bT7	117			glucosamine-6-phosphate isomerase	Metabolism; Carbohydrate Metabolism; Aminosugars Metabolism
SpESTZap1594	245			N-acetylglucosamine kinase	Metabolism; Carbohydrate Metabolism; Aminosugars Metabolism
gSpZar522gT3	149			UDP-N-acetylglucosamine pyrophosphorylase	Metabolism; Carbohydrate Metabolism; Aminosugars Metabolism
gSpZal255bMF	77			malate dehydrogenase	Metabolism; Carbohydrate Metabolism; Citrate cycle (TCA cycle)
gSpZar147bT7	45			malate dehydrogenase	Metabolism; Carbohydrate Metabolism; Citrate cycle (TCA cycle)
SpESTC228	228			malate dehydrogenase	Metabolism; Carbohydrate Metabolism; Citrate cycle (TCA cycle)
gSpZap180bT7	113			phosphomannomutase	Metabolism; Carbohydrate Metabolism; Fructose and mannose Metabolism
SpESTZap1908	224			Phosphomannomutase; pfam02878, COG1109	Metabolism; Carbohydrate Metabolism; Fructose and mannose Metabolism
gSpZap60gT3	212			pyrophosphate--fructose-6-phosphate 1-phosphotransferase	Metabolism; Carbohydrate Metabolism; Fructose and mannose Metabolism
gSpZap424gT3	180			beta-galactosidase	Metabolism; Carbohydrate Metabolism; Galactose Metabolism
SpESTZap1666	216			beta-galactosidase	Metabolism; Carbohydrate Metabolism; Galactose Metabolism
gSpTor1113bT7	47			UDP-glucose 4-epimerase	Metabolism; Carbohydrate Metabolism; Galactose Metabolism
Sp7orf2	306			UDP-glucose 4-epimerase	Metabolism; Carbohydrate Metabolism; Galactose Metabolism
SpESTZap1567	240			acetyl-CoA synthetase	Metabolism; Carbohydrate Metabolism; Glycolysis / Gluconeogenesis
27983189	880		LGT	alcohol dehydrogenase	Metabolism; Carbohydrate Metabolism; Glycolysis / Gluconeogenesis
27983404	397		LGT	alcohol dehydrogenase	Metabolism; Carbohydrate Metabolism; Glycolysis / Gluconeogenesis
SpESTDH325	235		LGT	alcohol dehydrogenase	Metabolism; Carbohydrate Metabolism; Glycolysis / Gluconeogenesis
SpESTZap588	229		LGT	alcohol dehydrogenase	Metabolism; Carbohydrate Metabolism; Glycolysis / Gluconeogenesis
gSpZap84gT3	122		LGT	alcohol dehydrogenase	Metabolism; Carbohydrate Metabolism; Glycolysis / Gluconeogenesis
SpESTZap1364	88			alcohol dehydrogenase (NADP+)	Metabolism; Carbohydrate Metabolism; Glycolysis / Gluconeogenesis
SpESTZap998	152			aldose 1-epimerase	Metabolism; Carbohydrate Metabolism; Glycolysis / Gluconeogenesis
SpESTC268	226			enolase	Metabolism; Carbohydrate Metabolism; Glycolysis / Gluconeogenesis
23266714	327		LGT	fructose-bisphosphate aldolase, class II	Metabolism; Carbohydrate Metabolism; Glycolysis / Gluconeogenesis
SpESTZap404	235		LGT	glucokinase	Metabolism; Carbohydrate Metabolism; Glycolysis / Gluconeogenesis
18030018	507		LGT	glucose-6-phosphate isomerase	Metabolism; Carbohydrate Metabolism; Glycolysis / Gluconeogenesis
1263024	297			glyceraldehyde 3-phosphate dehydrogenase	Metabolism; Carbohydrate Metabolism; Glycolysis / Gluconeogenesis
SpESTC202	258			phosphoglycerate kinase	Metabolism; Carbohydrate Metabolism; Glycolysis / Gluconeogenesis
gSpTol047bMF	193		LGT	phosphoglycerate mutase	Metabolism; Carbohydrate Metabolism; Glycolysis / Gluconeogenesis
SpESTC266	339			phosphoglycerate mutase	Metabolism; Carbohydrate Metabolism; Glycolysis / Gluconeogenesis

SpESTC275	213		LGT	pyruvate kinase	Metabolism; Carbohydrate Metabolism; Glycolysis / Gluconeogenesis
gSpTor696gT3	153			hydrogenase	Metabolism; Carbohydrate Metabolism; Glyoxylate and dicarboxylate Metabolism
gSpTor983gT3	146		LGT	phosphoglycolate phosphatase	Metabolism; Carbohydrate Metabolism; Glyoxylate and dicarboxylate Metabolism
gSpTor707bT7	144			phosphatidylinositol 3-kinase	Metabolism; Carbohydrate Metabolism; Inositol phosphate Metabolism
gSpZap388gT3	197			phosphatidylinositol 4-kinase	Metabolism; Carbohydrate Metabolism; Inositol phosphate Metabolism
SpESTC177	187			type I PIP kinase	Metabolism; Carbohydrate Metabolism; Inositol phosphate Metabolism
SpESTC118	236			dTDP-glucose 4,6-dehydratase	Metabolism; Carbohydrate Metabolism; Nucleotide sugars Metabolism
SpESTZap89	232			aldehyde reductase	Metabolism; Carbohydrate Metabolism; Pentose and glucuronate interconversions
gSpTor589bT7	122			L-arabinose isomerase	Metabolism; Carbohydrate Metabolism; Pentose and glucuronate interconversions
SpESTZap1761	186			UTP--glucose-1-phosphate uridylyltransferase	Metabolism; Carbohydrate Metabolism; Pentose and glucuronate interconversions
27982678	138			deoxyribose-phosphate aldolase	Metabolism; Carbohydrate Metabolism; Pentose phosphate pathway
SpESTDH419	204			ribokinase	Metabolism; Carbohydrate Metabolism; Pentose phosphate pathway
SpESTC23	142		LGT	ribose 5-phosphate isomerase B	Metabolism; Carbohydrate Metabolism; Pentose phosphate pathway
SpESTC198	341			ribose-phosphate pyrophosphokinase	Metabolism; Carbohydrate Metabolism; Pentose phosphate pathway
SpESTZap360	197		LGT	transketolase	Metabolism; Carbohydrate Metabolism; Pentose phosphate pathway
gSpZal591bMF	58			acetyl-CoA carboxylase carboxyl transferase subunit alpha	Metabolism; Carbohydrate Metabolism; Pyruvate Metabolism
gSpZap797bT7	131		LGT	acetyl-CoA synthetase (ADP-forming)	Metabolism; Carbohydrate Metabolism; Pyruvate Metabolism
gSpZap472bT7	164		LGT	hydroxyacylglutathione hydrolase	Metabolism; Carbohydrate Metabolism; Pyruvate Metabolism
SpESTZap1028	202		LGT	hydroxyacylglutathione hydrolase	Metabolism; Carbohydrate Metabolism; Pyruvate Metabolism
SpESTZap934	199			lactoylglutathione lyase	Metabolism; Carbohydrate Metabolism; Pyruvate Metabolism
gSpZap625gT3	181	TM		malate dehydrogenase (oxaloacetate-decarboxylating)	Metabolism; Carbohydrate Metabolism; Pyruvate Metabolism
5911348	1182			putative pyruvate-flavodoxin oxidoreductase	Metabolism; Carbohydrate Metabolism; Pyruvate Metabolism
gSpTor106bT7	281			putative pyruvate-flavodoxin oxidoreductase	Metabolism; Carbohydrate Metabolism; Pyruvate Metabolism
gSpTor106gT3	211			putative pyruvate-flavodoxin oxidoreductase	Metabolism; Carbohydrate Metabolism; Pyruvate Metabolism
gSpTor1241bT7	130			putative pyruvate-flavodoxin oxidoreductase	Metabolism; Carbohydrate Metabolism; Pyruvate Metabolism
SpESTC94	190		LGT	putative pyruvate-flavodoxin oxidoreductase	Metabolism; Carbohydrate Metabolism; Pyruvate Metabolism
SpESTZap870	181			putative pyruvate-flavodoxin oxidoreductase	Metabolism; Carbohydrate Metabolism; Pyruvate Metabolism
gSpZar1013gT3	238			1,4-alpha-glucan branching enzyme	Metabolism; Carbohydrate Metabolism; Starch and sucrose Metabolism
gSpZar359gT3	151			4-alpha-glucanotransferase	Metabolism; Carbohydrate Metabolism; Starch and sucrose Metabolism
gSpZal497bMF	122			beta-phosphoglucomutase	Metabolism; Carbohydrate Metabolism; Starch and sucrose Metabolism
gSpZap573bT7	140			beta-phosphoglucomutase	Metabolism; Carbohydrate Metabolism; Starch and sucrose Metabolism
SpESTC136	183			beta-phosphoglucomutase	Metabolism; Carbohydrate Metabolism; Starch and sucrose Metabolism
gSpZap510gT3	134		LGT	glycogen(starch) synthase	Metabolism; Carbohydrate Metabolism; Starch and sucrose Metabolism
SpESTDH093	134			glycogen(starch) synthase	Metabolism; Carbohydrate Metabolism; Starch and sucrose Metabolism
gSpTor1138gT3	97			starch phosphorylase	Metabolism; Carbohydrate Metabolism; Starch and sucrose Metabolism
gSpZap151gT3	78			starch phosphorylase	Metabolism; Carbohydrate Metabolism; Starch and sucrose Metabolism
SpESTC145	175	TM		V-type H+-transporting ATPase 16kDa proteolipid subunit	Metabolism; Energy Metabolism; ATP synthesis
SpESTC212	171	TM		V-type H+-transporting ATPase 16kDa proteolipid subunit	Metabolism; Energy Metabolism; ATP synthesis
SpESTC245	173	TM		V-type H+-transporting ATPase 16kDa proteolipid subunit	Metabolism; Energy Metabolism; ATP synthesis
SpESTC248	199	TM		V-type H+-transporting ATPase 21kDa proteolipid subunit	Metabolism; Energy Metabolism; ATP synthesis
SpESTC247	488			V-type H+-transporting ATPase subunit A	Metabolism; Energy Metabolism; ATP synthesis
SpESTC79	212			V-type H+-transporting ATPase subunit AC39	Metabolism; Energy Metabolism; ATP synthesis
gSpTor19gT3	163			V-type H+-transporting ATPase subunit B	Metabolism; Energy Metabolism; ATP synthesis
SpESTC71	99			V-type H+-transporting ATPase subunit G	Metabolism; Energy Metabolism; ATP synthesis
SpESTC113	198			aspartate aminotransferase	Metabolism; Energy Metabolism; Carbon fixation
30692540	200			peroxidase	Metabolism; Energy Metabolism; Methane Metabolism
SpESTC281	196			peroxidase	Metabolism; Energy Metabolism; Methane Metabolism
SpESTC239	273		LGT	carbamate kinase	Metabolism; Energy Metabolism; Nitrogen Metabolism
gSpZap126bT7	170		LGT	cystathionine beta-lyase	Metabolism; Energy Metabolism; Nitrogen Metabolism
gSpZap126gT3	185		LGT	cystathionine beta-lyase	Metabolism; Energy Metabolism; Nitrogen Metabolism
SpESTC259	128		LGT	cytochrome c biogenesis protein CcmG, thiol:disulfide interchange protein DsbE	Metabolism; Energy Metabolism; Nitrogen Metabolism
32480570	446			glutamate dehydrogenase (NADP+)	Metabolism; Energy Metabolism; Nitrogen Metabolism
20530910	634		LGT	glutamate synthase (NADPH) small chain	Metabolism; Energy Metabolism; Nitrogen Metabolism
27983817	477		LGT	hydroxylamine reductase	Metabolism; Energy Metabolism; Nitrogen Metabolism
gSpTor500gT3	90			L-asparaginase	Metabolism; Energy Metabolism; Nitrogen Metabolism
gSpTol313bMF	193		LGT	tryptophanase	Metabolism; Energy Metabolism; Nitrogen Metabolism
SpESTC204	242		LGT	tryptophanase	Metabolism; Energy Metabolism; Nitrogen Metabolism
gSpTor227bT7	219	TM		H+/K+-exchanging ATPase	Metabolism; Energy Metabolism; Oxidative phosphorylation
gSpTor604bT7	217	TM		H+/K+-exchanging ATPase	Metabolism; Energy Metabolism; Oxidative phosphorylation
gSpZap62bT7	197	TM		H+/K+-exchanging ATPase	Metabolism; Energy Metabolism; Oxidative phosphorylation
gSpZap8gT3	190			H+/K+-exchanging ATPase	Metabolism; Energy Metabolism; Oxidative phosphorylation
gSpTor671gT3	191			NADH dehydrogenase	Metabolism; Energy Metabolism; Oxidative phosphorylation
gSpZal229bMF	105		LGT	NADH dehydrogenase	Metabolism; Energy Metabolism; Oxidative phosphorylation
SpESTC219	319		LGT	NADH dehydrogenase	Metabolism; Energy Metabolism; Oxidative phosphorylation
SpESTDH113	217		LGT	NADH dehydrogenase	Metabolism; Energy Metabolism; Oxidative phosphorylation
11127702	467		LGT	NADH dehydrogenase	Metabolism; Energy Metabolism; Oxidative phosphorylation
gSpTor1143gT3	172		LGT	NADH dehydrogenase	Metabolism; Energy Metabolism; Oxidative phosphorylation
SpESTC115	206		LGT	cysteine synthase	Metabolism; Energy Metabolism; Sulfur Metabolism
SpESTZap1933	223	TM	LGT	cysteine synthase	Metabolism; Energy Metabolism; Sulfur Metabolism
SpESTC190	144			sulfite reductase, alpha subunit (flavoprotein),; COG0369	Metabolism; Energy Metabolism; Sulfur Metabolism
SpESTC233	146			sulfite reductase (NADPH) flavoprotein alpha-component	Metabolism; Energy Metabolism; Sulfur Metabolism
gSpZar995gT3	136			Acyl-CoA-dependent ceramide synthase	Metabolism; Glycan Biosynthesis and Metabolism; Glycosphingolipid Metabolism
Sp3orf8	381	TM		UDP-N-acetylglucosamine--dolichyl-phosphateN-acetylglucosaminephosphstransfer ase	Metabolism; Glycan Biosynthesis and Metabolism; N-Glycan
gSpTol251bMF	131			dolichyl-diphosphooligosaccharide--protein glycosyltransferase	Metabolism; Glycan Biosynthesis and Metabolism; N-Glycan biosynthesis
gSpZap856bT7	149			neuraminidase	Metabolism; Glycan Biosynthesis and Metabolism; N-Glycan degradation
gSpZap856gT3	156			neuraminidase	Metabolism; Glycan Biosynthesis and Metabolism; N-Glycan degradation
gSpZap471gT3	148		LGT	fatty acid desaturase	Metabolism; Lipid Metabolism; Androgen and estrogen Metabolism
SpESTC37	216	TM		3-oxo-5alpha-steroid 4-dehydrogenase	Metabolism; Lipid Metabolism; Bile acid biosynthesis
SpESTZap110	165		LGT	NAD(P)H dehydrogenase (quinone)	Metabolism; Lipid Metabolism; Biosynthesis of steroids
27983595	710		LGT	long-chain fatty-acid-CoA ligase	Metabolism; Lipid Metabolism; Fatty acid Metabolism
gSpTor1293bT7	153			long-chain fatty-acid-CoA ligase	Metabolism; Lipid Metabolism; Fatty acid Metabolism
gSpTor244bT7	159			long-chain fatty-acid-CoA ligase	Metabolism; Lipid Metabolism; Fatty acid Metabolism
gSpZap134gT3	129			long-chain fatty-acid-CoA ligase	Metabolism; Lipid Metabolism; Fatty acid Metabolism
gSpZar537gT3	134			long-chain fatty-acid-CoA ligase	Metabolism; Lipid Metabolism; Fatty acid Metabolism
gSpTor97gT3	134			glycerol kinase	Metabolism; Lipid Metabolism; Glycerolipid Metabolism
SpESTZap1131	164			triacylglycerol lipase	Metabolism; Lipid Metabolism; Glycerolipid Metabolism
gSpTor1417bT7	149	TM		CDP-diacylglycerol--glycerol-3-phosphate 3-phosphatidyltransferase	Metabolism; Lipid Metabolism; Glycerophospholipid Metabolism
SpESTZap2148	243		LGT	glycerol-3-phosphate dehydrogenase	Metabolism; Lipid Metabolism; Glycerophospholipid Metabolism
SpESTZap1753	205			phosphatidylserine decarboxylase; pfam02666.	Metabolism; Lipid Metabolism; Glycerophospholipid Metabolism
gSpTor927gT3	196	TM	LGT	phosphatidylserine synthase	Metabolism; Lipid Metabolism; Glycerophospholipid Metabolism
SpESTC27	297			NAD diphosphatase	Metabolism; Metabolism of Cofactors and Vitamins; Nicotinate and nicotinamide Metabolism
SpESTZap1290	183			pantothenate kinase	Metabolism; Metabolism of Cofactors and Vitamins; Pantothenate and CoA biosynthesis

Sp8orf2	518				phosphopantothenoylcysteine decarboxylase	Metabolism; Metabolism of Cofactors and Vitamins; Pantothenate and CoA biosynthesis
SpESTDH423	105				riboflavin kinase	Metabolism; Metabolism of Cofactors and Vitamins; Riboflavin Metabolism
SpESTC199	167				4-methyl-5(b-hydroxyethyl)-thiazole monophosphate biosynthesis	Metabolism; Metabolism of Cofactors and Vitamins; Thiamine Metabolism
SpESTDH245	161				4-methyl-5(b-hydroxyethyl)-thiazole monophosphate biosynthesis	Metabolism; Metabolism of Cofactors and Vitamins; Thiamine Metabolism
SpESTDH050	146				3-demethylubiquinone-9 3-methyltransferase	Metabolism; Metabolism of Cofactors and Vitamins; Ubiquinone biosynthesis
gSpZar919bT7	227			LGT	tripeptide aminopeptidase	Metabolism; Metabolism of Other Amino Acids; Glutathione Metabolism
SpESTDH279	90				Cysteine desulfurase (Nifsp in yeast); COG1104	Metabolism; Metabolism of Other Amino Acids
gSpZap418bT7	162				5'-nucleotidase	Metabolism; Nucleotide Metabolism; Purine Metabolism
SpESTZap1020	202				5'-nucleotidase	Metabolism; Nucleotide Metabolism; Purine Metabolism
gSpTor224gT3	91	C			5'-nucleotidase	Metabolism; Nucleotide Metabolism; Purine Metabolism
gSpZal311bMF	129	C			5'-nucleotidase	Metabolism; Nucleotide Metabolism; Purine Metabolism
gSpZap178gT3	160	C			5'-nucleotidase	Metabolism; Nucleotide Metabolism; Purine Metabolism
gSpZap35gT3	178	C			5'-nucleotidase	Metabolism; Nucleotide Metabolism; Purine Metabolism
gSpZap46gT3	188	C			5'-nucleotidase	Metabolism; Nucleotide Metabolism; Purine Metabolism
gSpZap882bT7	145	C			5'-nucleotidase	Metabolism; Nucleotide Metabolism; Purine Metabolism
gSpZap910bT7	115	C			5'-nucleotidase	Metabolism; Nucleotide Metabolism; Purine Metabolism
gSpZar313bT7	192	C			5'-nucleotidase	Metabolism; Nucleotide Metabolism; Purine Metabolism
Sp6orf1	363	C			5'-nucleotidase	Metabolism; Nucleotide Metabolism; Purine Metabolism
SpESTC46	180	C			5'-nucleotidase	Metabolism; Nucleotide Metabolism; Purine Metabolism
SpESTZap1999	177	C			5'-nucleotidase	Metabolism; Nucleotide Metabolism; Purine Metabolism
SpESTC154	173			LGT	adenine phosphoribosyltransferase	Metabolism; Nucleotide Metabolism; Purine Metabolism
gSpTol186bMF	190				adenosinetriphosphatase	Metabolism; Nucleotide Metabolism; Purine Metabolism
gSpTor1000bT7_2	130				adenosinetriphosphatase	Metabolism; Nucleotide Metabolism; Purine Metabolism
gSpTor1115gT3	187				adenosinetriphosphatase	Metabolism; Nucleotide Metabolism; Purine Metabolism
gSpTor1519bT7	218				adenosinetriphosphatase	Metabolism; Nucleotide Metabolism; Purine Metabolism
gSpTor657bT7	160				adenosinetriphosphatase	Metabolism; Nucleotide Metabolism; Purine Metabolism
gSpZap189gT3	186				adenosinetriphosphatase	Metabolism; Nucleotide Metabolism; Purine Metabolism
gSpZap289gT3	199				adenosinetriphosphatase	Metabolism; Nucleotide Metabolism; Purine Metabolism
gSpZap313gT3	203				adenosinetriphosphatase	Metabolism; Nucleotide Metabolism; Purine Metabolism
gSpZap56gT3	193				adenosinetriphosphatase	Metabolism; Nucleotide Metabolism; Purine Metabolism
gSpZap613bT7	108				adenosinetriphosphatase	Metabolism; Nucleotide Metabolism; Purine Metabolism
gSpZap626gT3	188				adenosinetriphosphatase	Metabolism; Nucleotide Metabolism; Purine Metabolism
gSpZap748gT3	170				adenosinetriphosphatase	Metabolism; Nucleotide Metabolism; Purine Metabolism
gSpZap846bT7	187				adenosinetriphosphatase	Metabolism; Nucleotide Metabolism; Purine Metabolism
gSpZap88bT7	141				adenosinetriphosphatase	Metabolism; Nucleotide Metabolism; Purine Metabolism
gSpZar174bT7	101				adenosinetriphosphatase	Metabolism; Nucleotide Metabolism; Purine Metabolism
gSpZar493gT3	160				adenosinetriphosphatase	Metabolism; Nucleotide Metabolism; Purine Metabolism
gSpZar538gT3	114				adenosinetriphosphatase	Metabolism; Nucleotide Metabolism; Purine Metabolism
gSpZar597gT3	205				adenosinetriphosphatase	Metabolism; Nucleotide Metabolism; Purine Metabolism
gSpZar607gT3	119				adenosinetriphosphatase	Metabolism; Nucleotide Metabolism; Purine Metabolism
gSpZar878gT3	243				adenosinetriphosphatase	Metabolism; Nucleotide Metabolism; Purine Metabolism
gSpZar999bT7	203				adenosinetriphosphatase	Metabolism; Nucleotide Metabolism; Purine Metabolism
SpESTZap140	175				adenosinetriphosphatase	Metabolism; Nucleotide Metabolism; Purine Metabolism
gSpZap101gT3	148				adenylate cyclase	Metabolism; Nucleotide Metabolism; Purine Metabolism
SpESTC235	203				adenylate kinase	Metabolism; Nucleotide Metabolism; Purine Metabolism
SpESTZap1939	160				ADP-ribose pyrophosphatase	Metabolism; Nucleotide Metabolism; Purine Metabolism
SpESTC251	228			LGT	deoxyguanosine kinase	Metabolism; Nucleotide Metabolism; Purine Metabolism
SpESTC254	207			LGT	deoxyguanosine kinase	Metabolism; Nucleotide Metabolism; Purine Metabolism
gSpTor745bT7	188		TM		guanylate cyclase	Metabolism; Nucleotide Metabolism; Purine Metabolism
SpESTC75	196				hypoxanthine phosphoribosyltransferase	Metabolism; Nucleotide Metabolism; Purine Metabolism
gSpTor125bT7	164			LGT	myosin ATPase	Metabolism; Nucleotide Metabolism; Purine Metabolism
SpESTC48	236				myosin ATPase	Metabolism; Nucleotide Metabolism; Purine Metabolism
gSpZar989gT3	245				nucleoside-diphosphate kinase	Metabolism; Nucleotide Metabolism; Purine Metabolism
SpESTC203	147				nucleoside-diphosphate kinase	Metabolism; Nucleotide Metabolism; Purine Metabolism
SpESTZap1122	132				nucleoside-triphosphate pyrophosphatase	Metabolism; Nucleotide Metabolism; Purine Metabolism
SpESTZap518	214				phosphoribosylformylglycinamidine cyclo-ligase	Metabolism; Nucleotide Metabolism; Purine Metabolism
gSpTor95gT3	138				polyribonucleotide nucleotidyltransferase	Metabolism; Nucleotide Metabolism; Purine Metabolism
27982526	517			LGT	CTP synthase	Metabolism; Nucleotide Metabolism; Pyrimidine Metabolism
gSpTor189bT7	84				CTP synthase	Metabolism; Nucleotide Metabolism; Pyrimidine Metabolism
gSpZap129bT7	165				CTP synthase	Metabolism; Nucleotide Metabolism; Pyrimidine Metabolism
SpESTZap137	127				cytidine deaminase	Metabolism; Nucleotide Metabolism; Pyrimidine Metabolism
SpESTC213	186				cytidylate kinase	Metabolism; Nucleotide Metabolism; Pyrimidine Metabolism
gSpZar448gT3	186				pseudouridylate synthase	Metabolism; Nucleotide Metabolism; Pyrimidine Metabolism
SpESTZap864	182				pseudouridylate synthase	Metabolism; Nucleotide Metabolism; Pyrimidine Metabolism
SpESTC217	305				thioredoxin reductase (NADPH)	Metabolism; Nucleotide Metabolism; Pyrimidine Metabolism
Sp5orf1	616				uridine kinase	Metabolism; Nucleotide Metabolism; Pyrimidine Metabolism
SpESTC192	220				uridine kinase	Metabolism; Nucleotide Metabolism; Pyrimidine Metabolism
27984028	401			LGT	A-type flavoprotein	Metabolism; unclassified
SpESTZap2025	189			LGT	A-type flavoprotein	Metabolism; unclassified
SpESTC196	228			LGT	carotenoid isomerase	Metabolism; unclassified
gSpZap998gT3	191			LGT	carotenoid isomerase	Metabolism; unclassified
gSpTor315gT3	210				deoxyhypusine synthase	Metabolism; unclassified
SpESTC267	192			LGT	rubrerythrin	Metabolism; unclassified
SpESTC282	210			LGT	rubrerythrin	Metabolism; unclassified
422 GSSs			47	17	conserved hypothetical protein	
105 GSSs		105	18		conserved hypothetical protein	
1 GSS					conserved hypothetical protein	
12 ORFs in contigs			4		conserved hypothetical protein	
3 ORFs in contigs		3	2		conserved hypothetical protein	
173 ESTs			28		conserved hypothetical protein	
14 ESTs		14	1		conserved hypothetical protein	
2 ESTs					conserved hypothetical protein	
13 ORFs in contigs			4		hypothetical protein	

[1]) Indicates the length of the putative amino acid sequence.
[2]) Indicates cysteine-rich (>10%) proteins that were excluded from the phylogenomic analysis.
[3]) Indicates proteins with one or more transmembrane (TM) helices predicted by TMHMM server version 2.0 (http://www.cbs.dtu.dk/services/TMHMM/).
[4]) Indicates proteins encoded by genes with a putative LGT origin (see Additional files 3-6 for details).
