# Supplementary material for: A genomic survey of the fish parasite Spironucleus salmonicida indicates genomic plasticity among diplomonads and significant lateral gene transfer in eukaryote genome evolution
Source: BMC Genomics. 2007 Feb 14;8:51. doi: 10.1186/1471-2164-8-51 (PMC1805757; doi:10.1186/1471-2164-8-51)
Supplement: Additional file 3 — A table showing the genes putatively involved in LGT events. [file 1471-2164-8-51-S3.pdf]

A genomic survey of the fish parasite *Spironucleus salmonicida* indicates genomic plasticity among diplomonads and significant lateral gene transfer in eukaryote genome evolution

Jan O. Andersson, Åsa M. Sjögren, David S. Horner, Colleen A. Murphy, Patricia L. Dyal, Staffan G. Svärd, John M. Logsdon, Jr., Mark A. Ragan, Robert P. Hirt, and Andrew J. Roger

Additional file 3

Candidate LGT genes in the *Spironucleus salmonicida* genome survey.

Clone	Protein	Classification	Tree#[3]	#aa positions	Monophyletic diplomonads	Monophyletic diplomonads and parabasalids	Eukaryote-to-eukaryote LGT event[6]
SpESTZap1389	drug:H+ antiporter-2, DHA2 family	Environmental information processing; membrane transport; major facilitator superfamily (MFS)	1	170	+		
SpESTC18[1]	membrane protease subunit HflK	Genetic information processing; folding, sorting and degradation; protein folding and associated processing	2	160	-		DD
gZar958bT7[1]	membrane protease subunit HflK	Genetic information processing; folding, sorting and degradation; protein folding and associated processing	3	125	-		DD
SpESTC43	DNA glycosylase	Genetic information processing; replication and repair; other replication, recombination and repair factors	4	165	+		DD
SpESTZap1340	tRNA (5-methylaminomethyl-2-thiouridylate)-methyltransferase	Genetic information processing; unclassified	5	107	-		
27982008	alanyl-tRNA synthetase	Metabolism; amino acid metabolism; alanine and aspartate metabolism	6	451	+[4]	+[5]	EH[5]
SpESTC149[1]	aminoacylhistidine dipeptidase	Metabolism; amino acid metabolism; alanine and aspartate metabolism	7	247	+	+	
SpESTDH287[1]	aminoacylhistidine dipeptidase	Metabolism; amino acid metabolism; alanine and aspartate metabolism	7	247	+	+	
SpESTZap149[1]	aminoacylhistidine dipeptidase	Metabolism; amino acid metabolism; alanine and aspartate metabolism	7	247	+	+	
SpESTC31	5'-methylthioadenosine nucleosidase	Metabolism; amino acid metabolism; arginine and proline metabolism	8	168	+		
SpESTC83	arginine deiminase	Metabolism; amino acid metabolism; arginine and proline metabolism	9	356	+	+	DD
SpESTC221	ornithine carbamoyltransferase	Metabolism; amino acid metabolism; arginine and proline metabolism	10	303	+	+	
27981826	prolyl-tRNA synthetase	Metabolism; amino acid metabolism; arginine and proline metabolism	11	175	+	+	
gZar602gT3[1]	sarcosine oxidase	Metabolism; amino acid metabolism; glycine, serine and threonine metabolism	12	218	-		EH
gZap30gT3[1]	sarcosine oxidase	Metabolism; amino acid metabolism; glycine, serine and threonine metabolism	12	218	-		EH
27982357	threonine dehydratase	Metabolism; amino acid metabolism; glycine, serine and threonine metabolism	13	269	+[4]	+	
SpESTZap946	histidinol-phosphatase (PHP family)	Metabolism; amino acid metabolism; histidine metabolism	14	113	+		
gZap84gT3[1]	alcohol dehydrogenase	Metabolism; carbohydrate metabolism; glycolysis / gluconeogenesis	15	121	+		EH
SpESTZap588[1]	alcohol dehydrogenase	Metabolism; carbohydrate metabolism; glycolysis / gluconeogenesis	16	147	+		EH
27983189	alcohol dehydrogenase	Metabolism; carbohydrate metabolism; glycolysis / gluconeogenesis	16	147	+		
27983404	alcohol dehydrogenase	Metabolism; carbohydrate metabolism; glycolysis / gluconeogenesis	16	147	+		
SpESTDH325	alcohol dehydrogenase	Metabolism; carbohydrate metabolism; glycolysis / gluconeogenesis	17	223	-		

23266714	fructose-bisphosphate aldolase, class II	Metabolism; carbohydrate metabolism; glycolysis / gluconeogenesis	18	300	+		
SpESTZap404	glucokinase	Metabolism; carbohydrate metabolism; glycolysis / gluconeogenesis	19	192	+		
18030018	glucose-6-phosphate isomerase	Metabolism; carbohydrate metabolism; glycolysis / gluconeogenesis	20	412	+	+	
gTol047bMF	phosphoglycerate mutase	Metabolism; carbohydrate metabolism; glycolysis / gluconeogenesis	21	184	+		
SpESTC275	pyruvate kinase	Metabolism; carbohydrate metabolism; glycolysis / gluconeogenesis	22	205	+[4]		
gTor983gT3	phosphoglycolate phosphatase	Metabolism; carbohydrate metabolism; glyoxylate and dicarboxylate metabolism	23	116	-		
SpESTC23	ribose 5-phosphate isomerase B	Metabolism; carbohydrate metabolism; pentose phosphate pathway	24	136	+	+	
SpESTZap360	transketolase	Metabolism; carbohydrate metabolism; pentose phosphate pathway	25	196	+		
gZap797bT7	acetyl-CoA synthetase (ADP-forming)	Metabolism; carbohydrate metabolism; pyruvate metabolism	26	121	+	+	
SpESTZap1028[1]	hydroxyacylglutathione hydrolase	Metabolism; carbohydrate metabolism; pyruvate metabolism	27	164	+		
gZap472bT7[1]	hydroxyacylglutathione hydrolase	Metabolism; carbohydrate metabolism; pyruvate metabolism	27	164	+		
SpESTC94	putative pyruvate-flavodoxin oxidoreductase	Metabolism; carbohydrate metabolism; pyruvate metabolism	28	167	+		MB
gZap510gT3	glycogen(starch) synthase	Metabolism; carbohydrate metabolism; starch and sucrose metabolism	29	111	+		
SpESTC239	carbamate kinase	Metabolism; energy metabolism; nitrogen metabolism	30	273	+		
gZap126bT7[2]	cystathionine beta-lyase	Metabolism; energy metabolism; nitrogen metabolism	31	157	-		
gZap126gT3[2]	cystathionine beta-lyase	Metabolism; energy metabolism; nitrogen metabolism	32	182	-		
SpESTC259[1]	cytochrome c biogenesis protein CcmG, thiol:disulfide interchangeprotein DsbE	Metabolism; energy metabolism; nitrogen metabolism	33	110	+		
20530910	glutamate synthase (NADPH) small chain	Metabolism; energy metabolism; nitrogen metabolism	34	243	+		
27983817	hydroxylamine reductase	Metabolism; energy metabolism; nitrogen metabolism	35	471	+		
gTol313bMF[2]	tryptophanase	Metabolism; energy metabolism; nitrogen metabolism	36	160	-		
SpESTC204[2]	tryptophanase	Metabolism; energy metabolism; nitrogen metabolism	37	164	-		
gZal229bMF	NADH dehydrogenase	Metabolism; energy metabolism; oxidative phosphorylation	38	105	+[4]		
SpESTC219	NADH dehydrogenase	Metabolism; energy metabolism; oxidative phosphorylation	39	315	+[4]		
SpESTDH113	NADH dehydrogenase	Metabolism; energy metabolism; oxidative phosphorylation	40	155	-		
11127702[1]	NADH dehydrogenase	Metabolism; energy metabolism; oxidative phosphorylation	41	303	+		EH
gTor1143gT3[1]	NADH dehydrogenase	Metabolism; energy metabolism; oxidative phosphorylation	42	155	+		EH
SpESTC115[1]	cysteine synthase	Metabolism; energy metabolism; sulfur metabolism	43	200	-		
SpESTZap1933[1]	cysteine synthase	Metabolism; energy metabolism; sulfur metabolism	44	214	-		
gZap471gT3	fatty acid desaturase	Metabolism; lipid metabolism; androgen and estrogen metabolism	45	143	-		
SpESTZap110	NAD(P)H dehydrogenase (quinone)	Metabolism; lipid metabolism; biosynthesis of steroids	46	127	+		
27983595	long-chain fatty-acid-CoA ligase	Metabolism; lipid metabolism; fatty acid metabolism	47	682	+		
SpESTZap2148	glycerol-3-phosphate dehydrogenase	Metabolism; lipid metabolism; glycerophospholipid metabolism	48	185	+		EH
gTor927gT3	phosphatidylserine synthase	Metabolism; lipid metabolism; glycerophospholipid metabolism	49	168	+		
gZar919bT7	tripeptide aminopeptidase	Metabolism; metabolism of other amino acids; glutathione metabolism	50	214	-		EH
SpESTC154	adenine phosphoribosyltransferase	Metabolism; nucleotide metabolism; purine metabolism	51	154	+		
SpESTC254	deoxyguanosine kinase	Metabolism; nucleotide metabolism; purine metabolism	52	182	+		DD
SpESTC251	deoxyguanosine kinase	Metabolism; nucleotide metabolism; purine metabolism	52	182	+		DD
gTor125bT7	myosin ATPase	Metabolism; nucleotide metabolism; purine metabolism	53	135	+		
27982526	CTP synthase	Metabolism; nucleotide metabolism; pyrimidine metabolism	54	511	-		
27984028[1]	A-type flavoprotein	Metabolism; unclassified	55	383	+	+	
SpESTZap2025[1]	A-type flavoprotein	Metabolism; unclassified	56	168	+	+	
SpESTC196[1]	carotenoid isomerase	Metabolism; unclassified	57	211	-		
gZap998gT3[1]	carotenoid isomerase	Metabolism; unclassified	57	211	-		
SpESTC282[1]	rubrerythrin	Metabolism; unclassified	58	173	-		
SpESTC267[1]	rubrerythrin	Metabolism; unclassified	58	173	-		
gTor1213bT7	conserved hypothetical protein		59	144	-		GZ
gTor541gT3	conserved hypothetical protein		60	199	+		
gTor565bT7	conserved hypothetical protein		61	160	+		

gTor916gT3	conserved hypothetical protein	62	175	+	+
gTor930bT7	conserved hypothetical protein	63	163	+	
gZap260bT7	conserved hypothetical protein	64	123	-	
gZap950bT7	conserved hypothetical protein	65	111	+	
gZar587bT72	conserved hypothetical protein	66	124	-	
gZar847bT7	conserved hypothetical protein	67	137	+	+
gZar96bT7	conserved hypothetical protein	68	147	-	
gZar978gT3[1]	conserved hypothetical protein	69	185	-	
gZar789bT7[1]	conserved hypothetical protein	69	185	-	
Sp11orf4	conserved hypothetical protein	70	586	+	
SpESTZap1004	conserved hypothetical protein	71	197	+	
SpESTZap1248	conserved hypothetical protein	72	118	+	
SpESTZap1299[1]	conserved hypothetical protein	33	110	+	
SpESTZap1913[1]	conserved hypothetical protein	33	110	+	

[1]) The phylogenetic analyses of these genes indicate that they likely have undergone duplications after the putative LGT events.
[2]) These pairs of sequences with identical annotations correspond to different parts of the protein and could represent the same gene in the genome.
[3]) Phylogenetic trees for all candidate LGT genes are shown in Additional files 4-6.
[4]) *G. lamblia* homologs for these proteins are present in the similarity searches and most likely are the closest relatives to the *S. salmonicida* sequences. However, the *G. lamblia* homologs are absent from the phylogenetic trees listed since the automated generation of datasets [1] reached the maximum number of sequences to include (200) and only selected a subset of the available homologous sequences in the databases.
[5]) A previous phylogenetic study of alanyl tRNA synthetase has both shown diplomonads and parabasalids to be monophyletic, and gene transfers between eukaryotic lineages [2]. These cases are not observed in the presented tree since the *Trichomonas*, *Entamoeba*, and ciliate homologs were excluded due to the reasons outlined above.
[6]) The putative recipient and/or donor eukaryotic lineages are indicted by DD (*Dictyostelium discoideum*), EH (*Entamoeba histolytica*), GZ (*Gibberella zeae*), and MB (*Mastigamoeba balamuthi*).

References

1. Frickey T, Lupas AN: **PhyloGenie: automated phylome generation and analysis.** *Nucleic Acids Res* 2004, **32:**5231-5238.
2. Andersson JO, Sarchfield SW, Roger AJ: **Gene transfers from Nanoarchaeota to an ancestor of diplomonads and parabasalids.** *Mol Biol Evol* 2005, **22:**85-90.
