# Supplementary material for: A genomic survey of the fish parasite Spironucleus salmonicida indicates genomic plasticity among diplomonads and significant lateral gene transfer in eukaryote genome evolution
Source: BMC Genomics. 2007 Feb 14;8:51. doi: 10.1186/1471-2164-8-51 (PMC1805757; doi:10.1186/1471-2164-8-51)

Additional file 4 - Andersson *et al.*

Phylogenetic trees 1-25 for genes putatively involved in LGT events and listed in Additional file 3.

Tree #1: SpESTZap1389

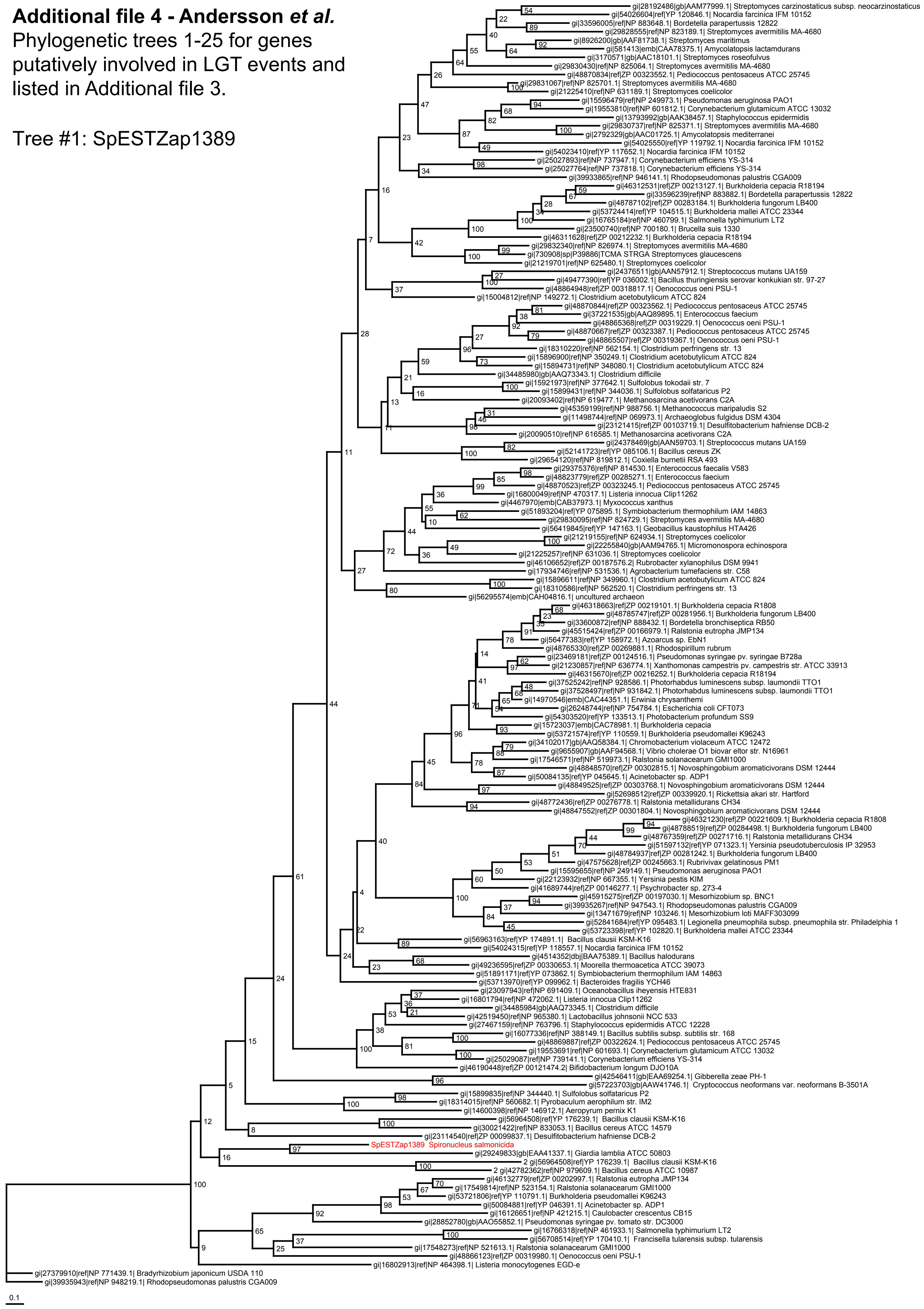

Tree #2: SpESTC18

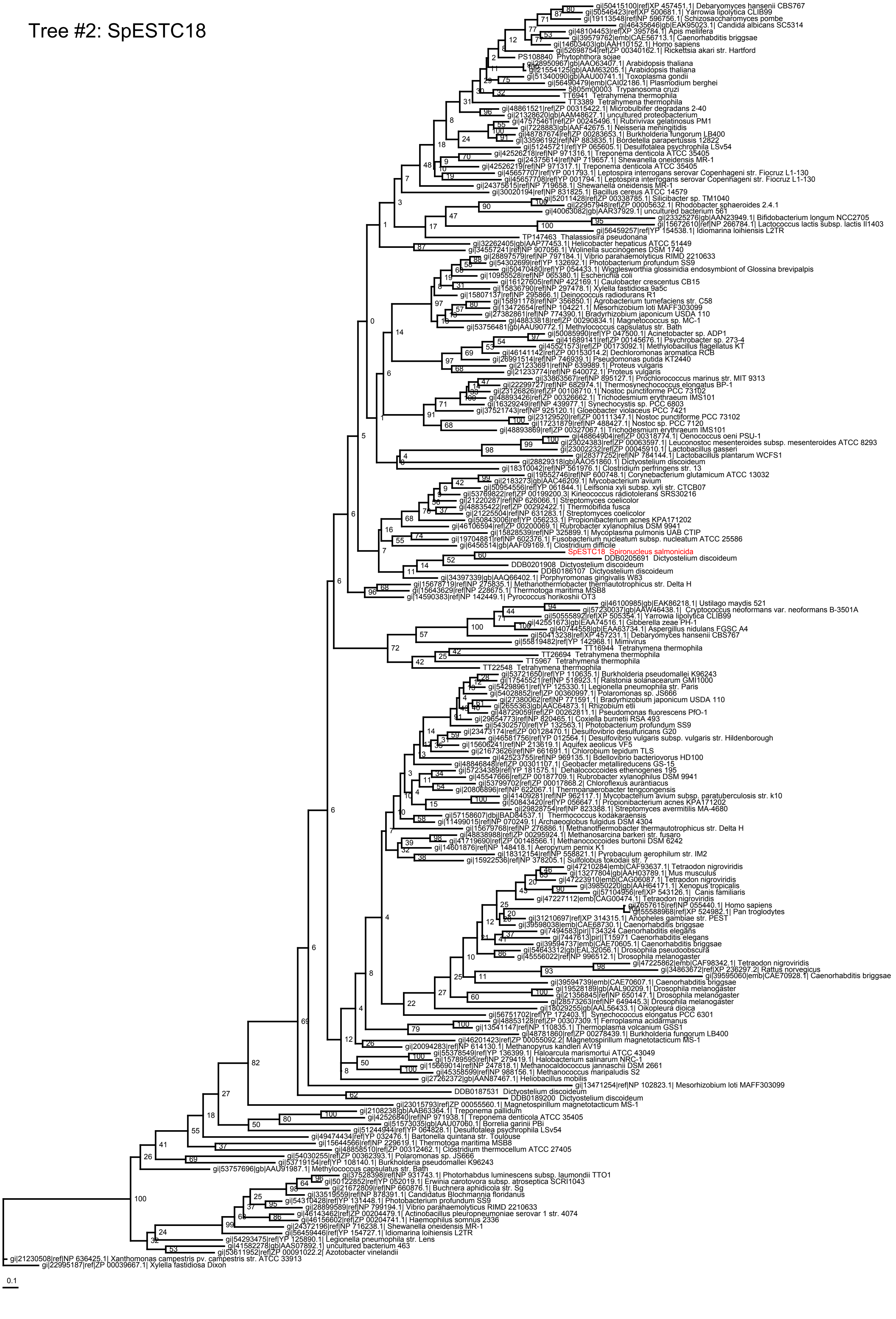

Tree #3: gZar958bT7

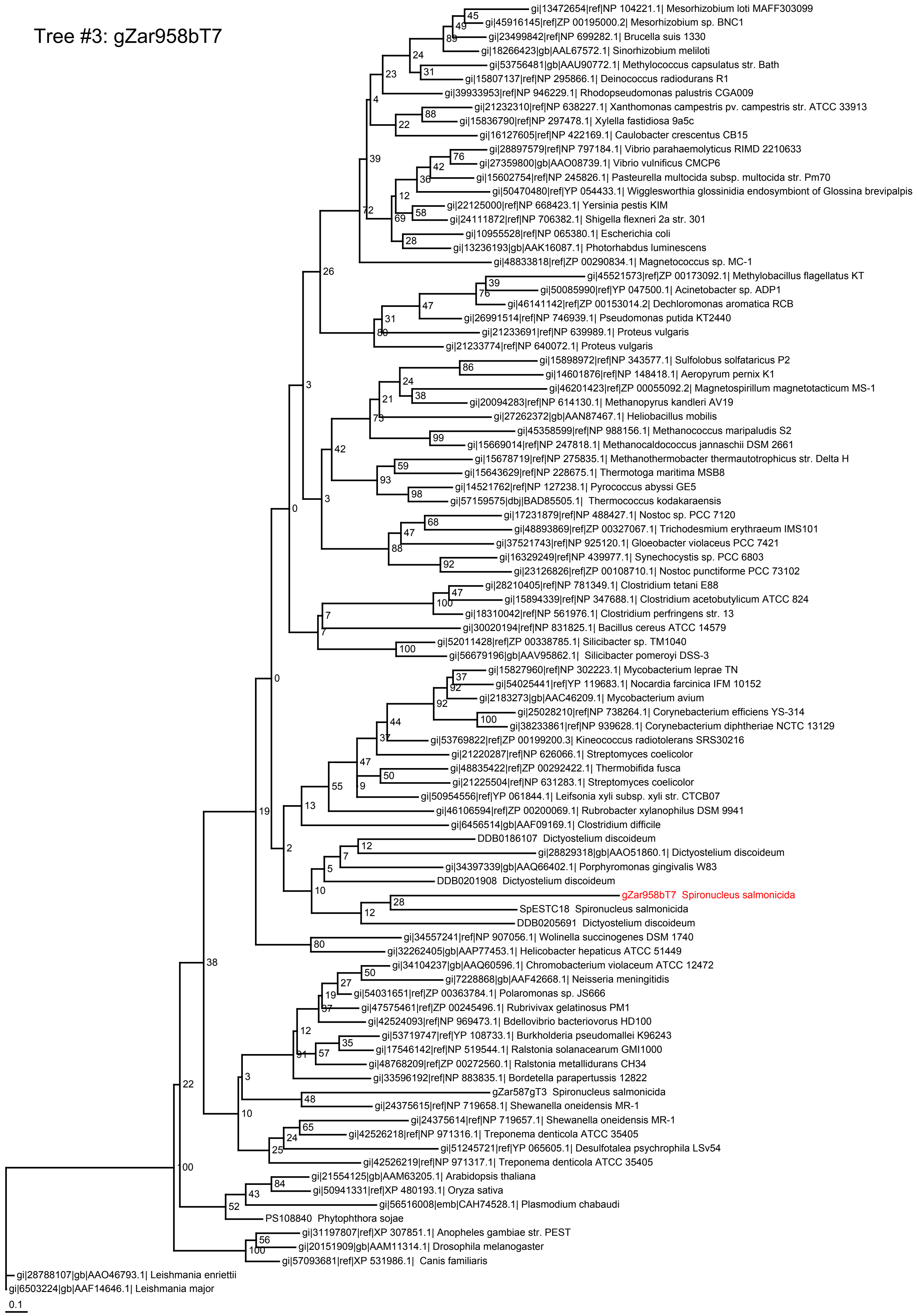

Tree #4: SpESTC43

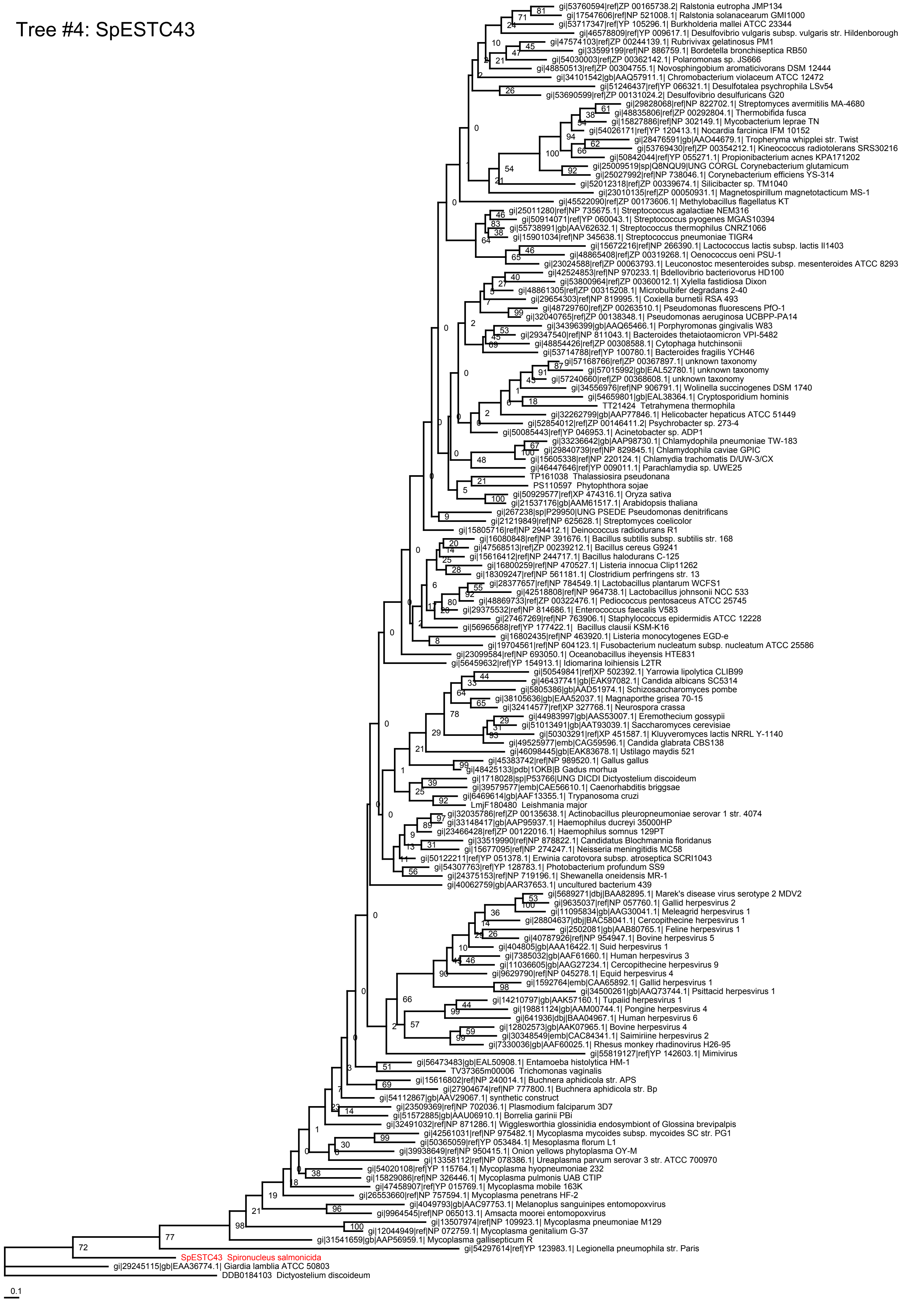

Tree #5: SpESTZap1340

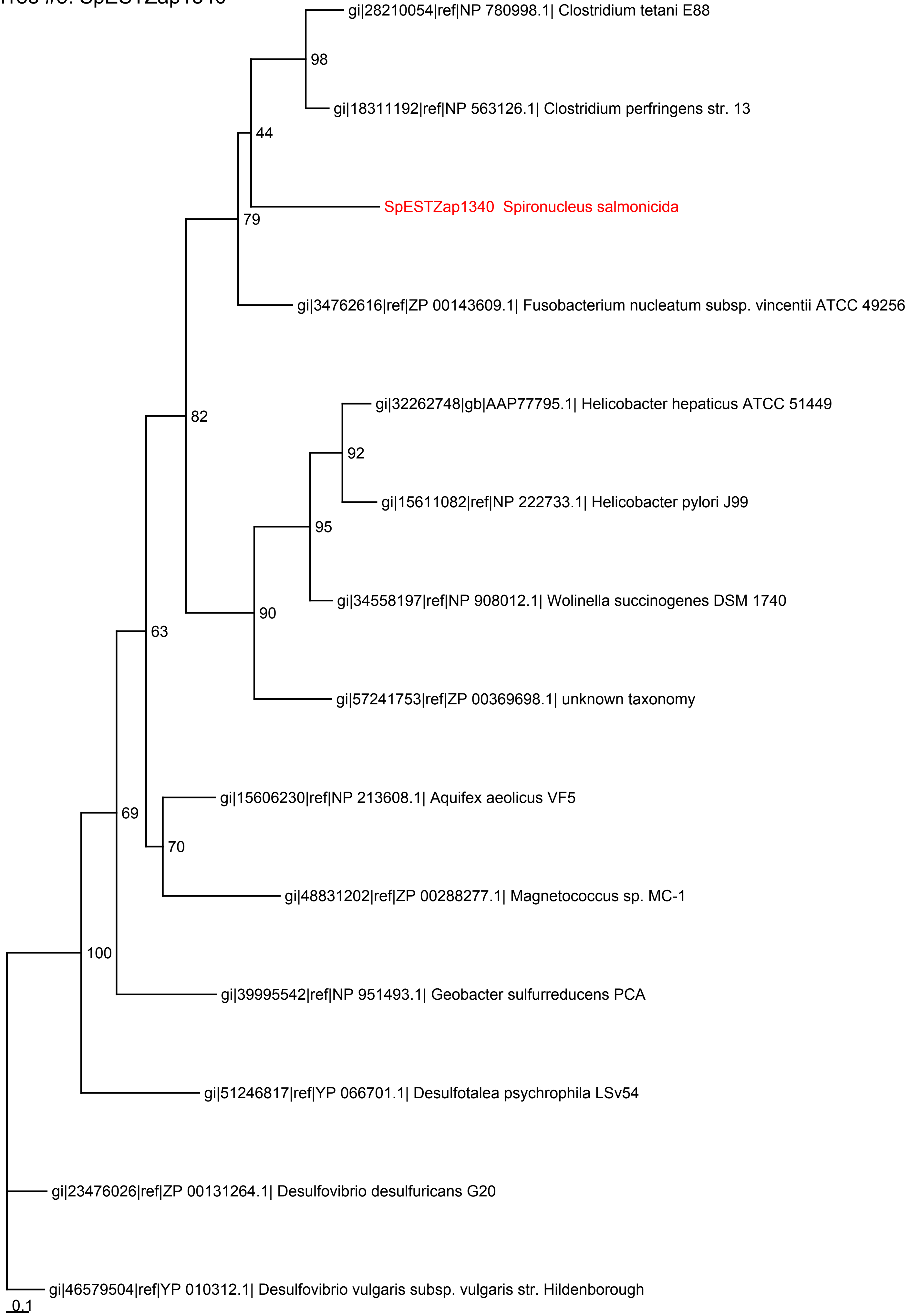

Tree #6: 27982008

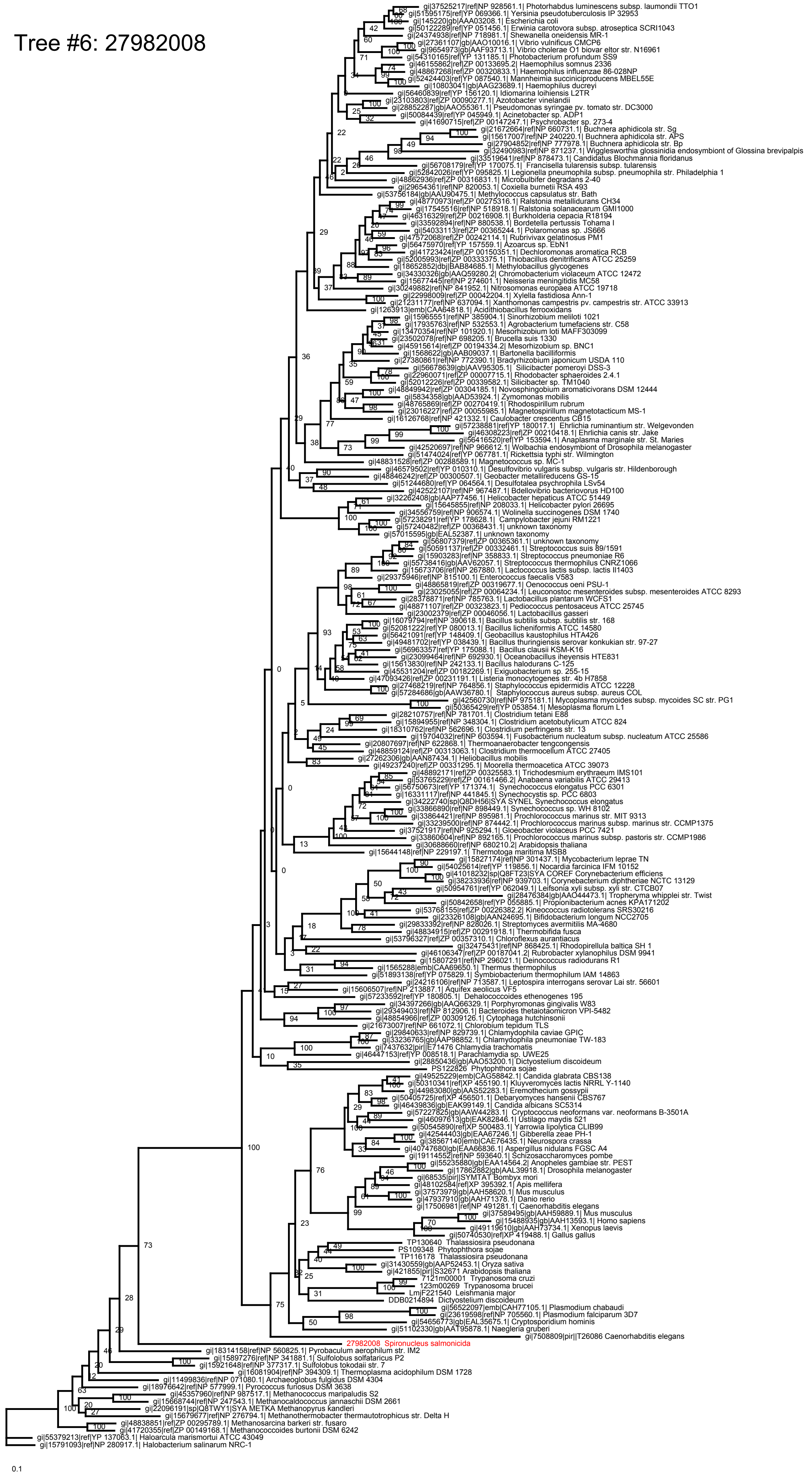

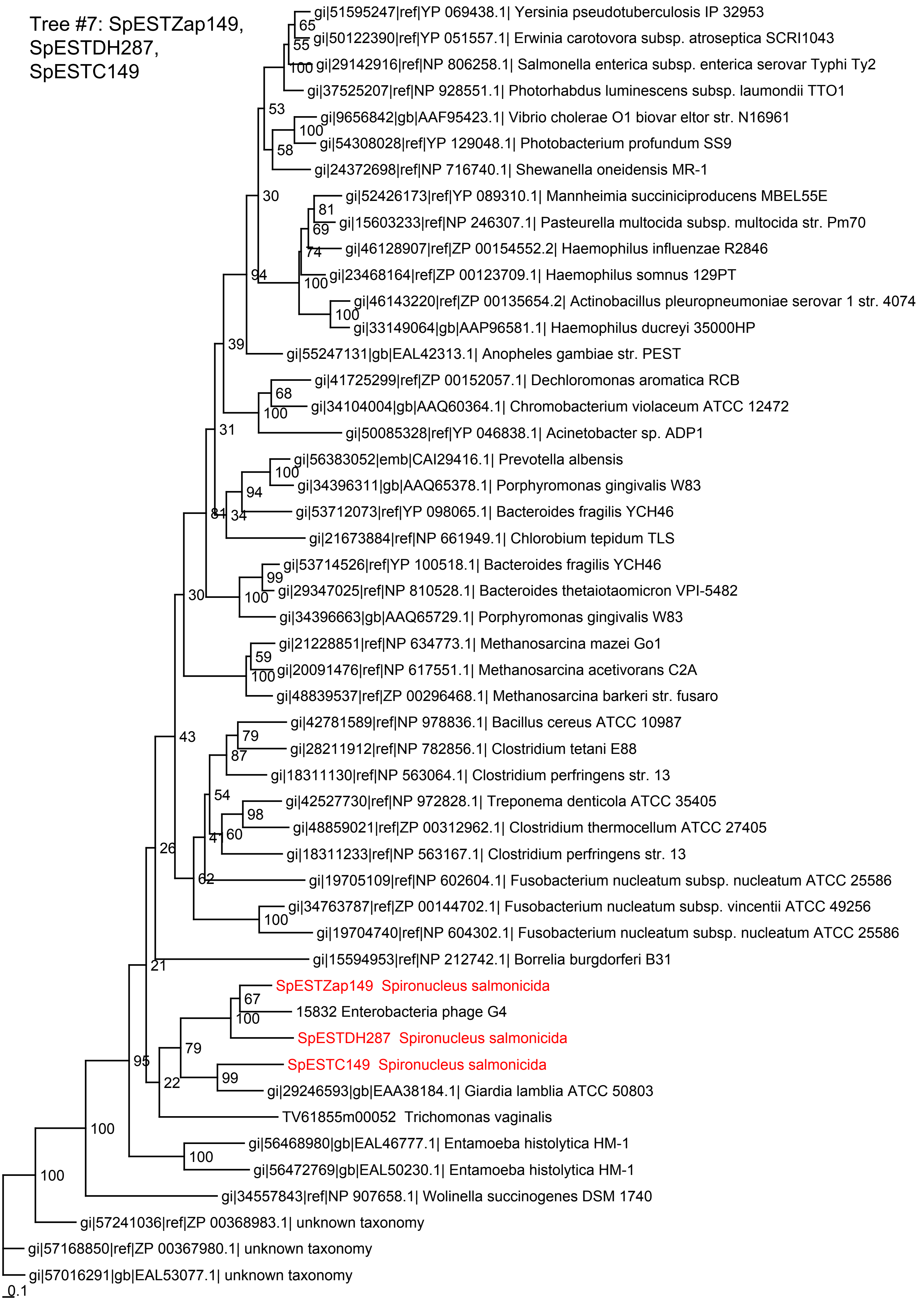
Tree #7: SpESTZap149, SpESTDH287, SpESTC149
gi|51595247|ref|YP 069438.1| Yersinia pseudotuberculosis IP 32953
gi|50122390|ref|YP 051557.1| Erwinia carotovora subsp. atroseptica SCRI1043
gi|29142916|ref|NP 806258.1| Salmonella enterica subsp. enterica serovar Typhi Ty2
gi|37525207|ref|NP 928551.1| Photorhabdus luminescens subsp. laumondii TTO1
gi|9656842|gb|AAF95423.1| Vibrio cholerae O1 biovar eltor str. N16961
gi|54308028|ref|YP 129048.1| Photobacterium profundum SS9
gi|24372698|ref|NP 716740.1| Shewanella oneidensis MR-1
gi|52426173|ref|YP 089310.1| Mannheimia succiniciproducens MBEL55E
gi|15603233|ref|NP 246307.1| Pasteurella multocida subsp. multocida str. Pm70
gi|46128907|ref|ZP 00154552.2| Haemophilus influenzae R2846
gi|23468164|ref|ZP 00123709.1| Haemophilus somnus 129PT
gi|46143220|ref|ZP 00135654.2| Actinobacillus pleuropneumoniae serovar 1 str. 4074
gi|33149064|gb|AAP96581.1| Haemophilus ducreyi 35000HP
gi|55247131|gb|EAL42313.1| Anopheles gambiae str. PEST
gi|41725299|ref|ZP 00152057.1| Dechloromonas aromatica RCB
gi|34104004|gb|AAQ60364.1| Chromobacterium violaceum ATCC 12472
gi|50085328|ref|YP 046838.1| Acinetobacter sp. ADP1
gi|56383052|emb|CAI29416.1| Prevotella albensis
gi|34396311|gb|AAQ65378.1| Porphyromonas gingivalis W83
gi|53712073|ref|YP 098065.1| Bacteroides fragilis YCH46
gi|21673884|ref|NP 661949.1| Chlorobium tepidum TLS
gi|53714526|ref|YP 100518.1| Bacteroides fragilis YCH46
gi|29347025|ref|NP 810528.1| Bacteroides thetaiotaomicron VPI-5482
gi|34396663|gb|AAQ65729.1| Porphyromonas gingivalis W83
gi|21228851|ref|NP 634773.1| Methanosarcina mazei Go1
gi|20091476|ref|NP 617551.1| Methanosarcina acetivorans C2A
gi|48839537|ref|ZP 00296468.1| Methanosarcina barkeri str. fusaro
gi|42781589|ref|NP 978836.1| Bacillus cereus ATCC 10987
gi|28211912|ref|NP 782856.1| Clostridium tetani E88
gi|18311130|ref|NP 563064.1| Clostridium perfringens str. 13
gi|42527730|ref|NP 972828.1| Treponema denticola ATCC 35405
gi|48859021|ref|ZP 00312962.1| Clostridium thermocellum ATCC 27405
gi|18311233|ref|NP 563167.1| Clostridium perfringens str. 13
gi|19705109|ref|NP 602604.1| Fusobacterium nucleatum subsp. nucleatum ATCC 25586
gi|34763787|ref|ZP 00144702.1| Fusobacterium nucleatum subsp. vincentii ATCC 49256
gi|19704740|ref|NP 604302.1| Fusobacterium nucleatum subsp. nucleatum ATCC 25586
gi|15594953|ref|NP 212742.1| Borrelia burgdorferi B31
SpESTZap149 Spironucleus salmonicida
15832 Enterobacteria phage G4
SpESTDH287 Spironucleus salmonicida
SpESTC149 Spironucleus salmonicida
gi|29246593|gb|EAA38184.1| Giardia lamblia ATCC 50803
TV61855m00052 Trichomonas vaginalis
gi|56468980|gb|EAL46777.1| Entamoeba histolytica HM-1
gi|56472769|gb|EAL50230.1| Entamoeba histolytica HM-1
gi|34557843|ref|NP 907658.1| Wolinella succinogenes DSM 1740
gi|57241036|ref|ZP 00368983.1| unknown taxonomy
gi|57168850|ref|ZP 00367980.1| unknown taxonomy
gi|57016291|gb|EAL53077.1| unknown taxonomy
0.1

Tree #8: SpESTC31

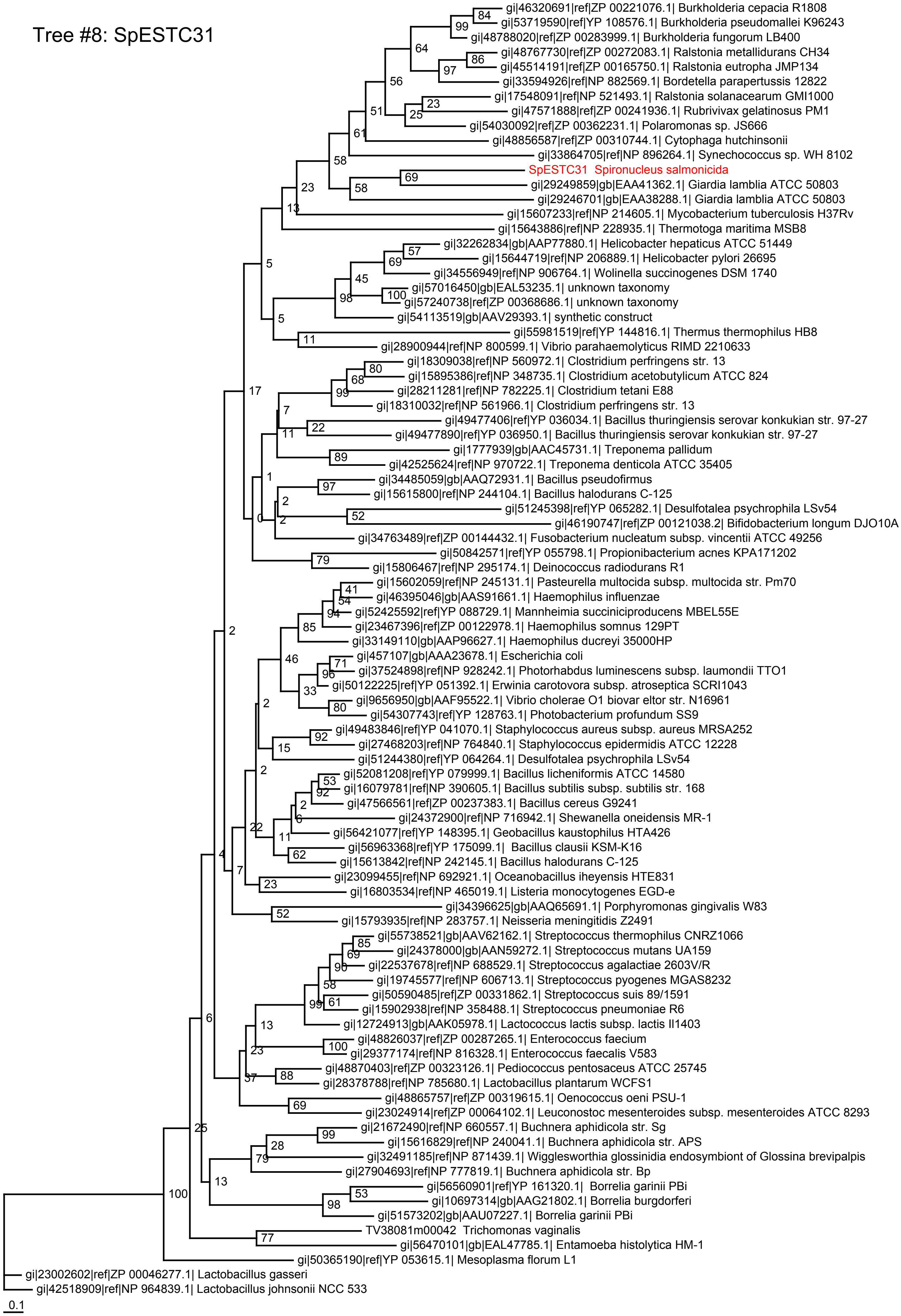

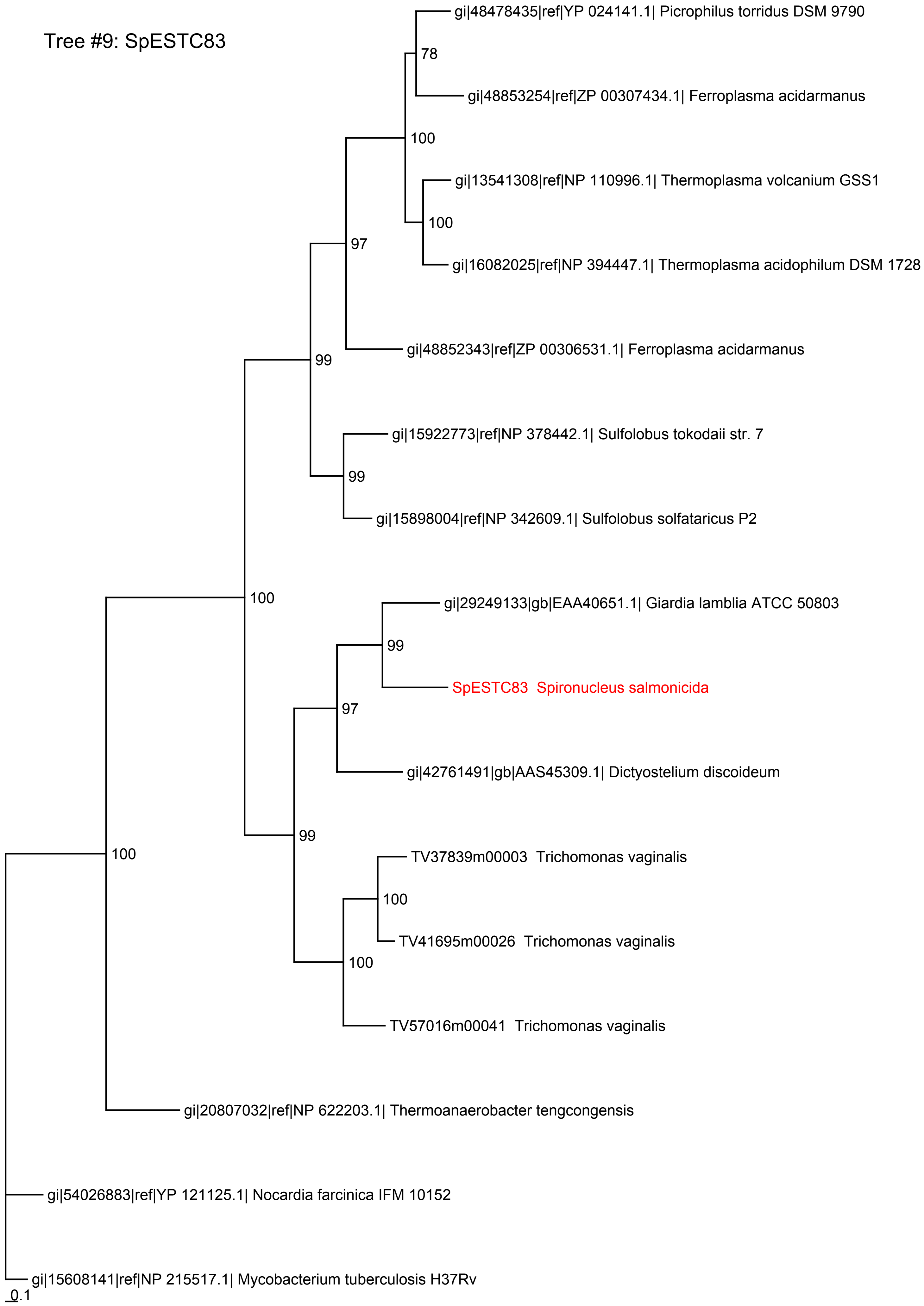
Tree #9: SpESTC83
gi|48478435|ref|YP 024141.1| Picrophilus torridus DSM 9790
78
gi|48853254|ref|ZP 00307434.1| Ferroplasma acidarmanus
100
gi|13541308|ref|NP 110996.1| Thermoplasma volcanium GSS1
100
gi|16082025|ref|NP 394447.1| Thermoplasma acidophilum DSM 1728
97
gi|48852343|ref|ZP 00306531.1| Ferroplasma acidarmanus
99
gi|15922773|ref|NP 378442.1| Sulfolobus tokodaii str. 7
99
gi|15898004|ref|NP 342609.1| Sulfolobus solfataricus P2
100
gi|29249133|gb|EAA40651.1| Giardia lamblia ATCC 50803
99
SpESTC83 Spironucleus salmonicida
97
gi|42761491|gb|AAS45309.1| Dictyostelium discoideum
99
TV37839m00003 Trichomonas vaginalis
100
TV41695m00026 Trichomonas vaginalis
100
TV57016m00041 Trichomonas vaginalis
100
gi|20807032|ref|NP 622203.1| Thermoanaerobacter tengcongensis
gi|54026883|ref|YP 121125.1| Nocardia farcinica IFM 10152
gi|15608141|ref|NP 215517.1| Mycobacterium tuberculosis H37Rv
0.1

Tree #10: SpESTC221

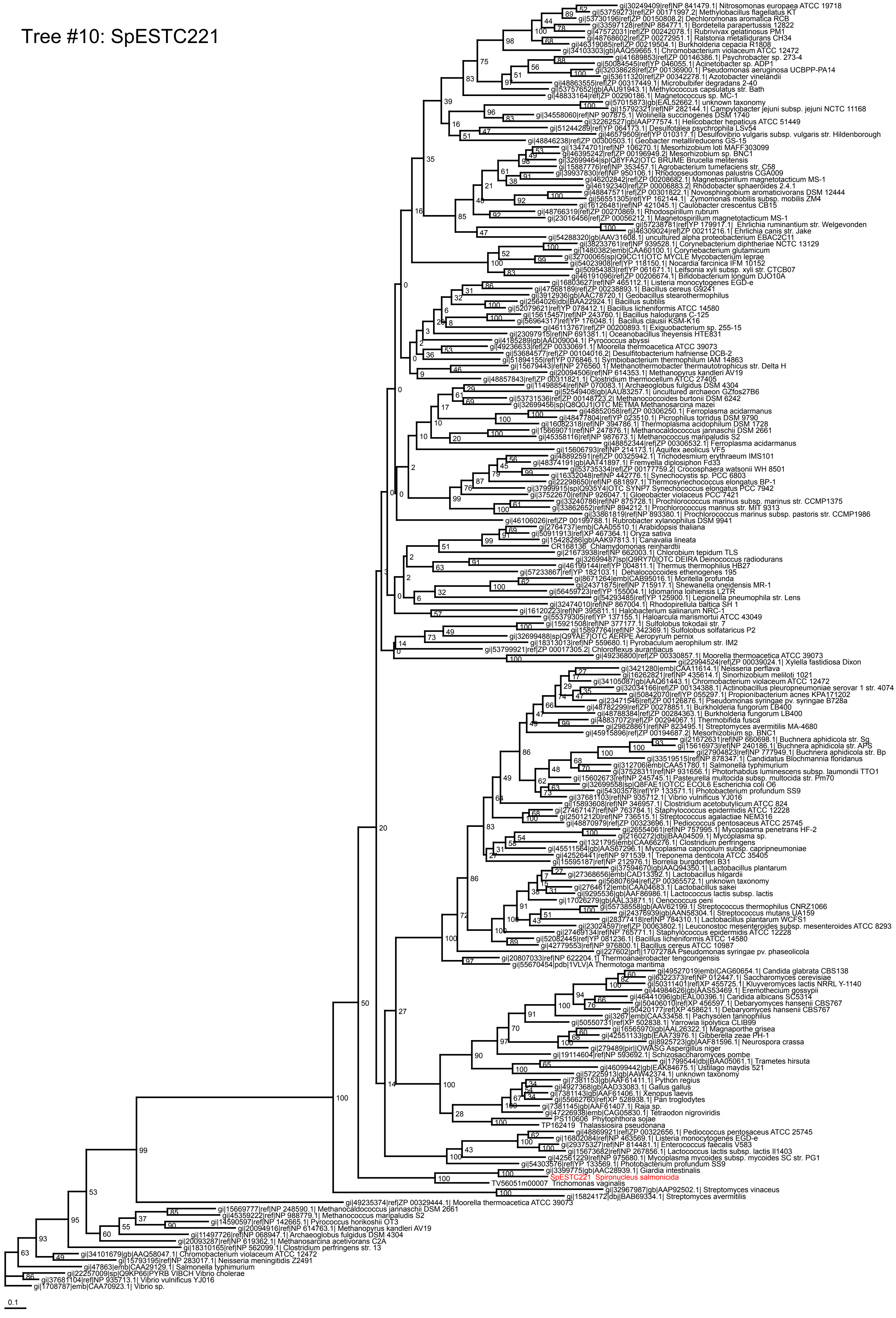

Tree #11: 27981826

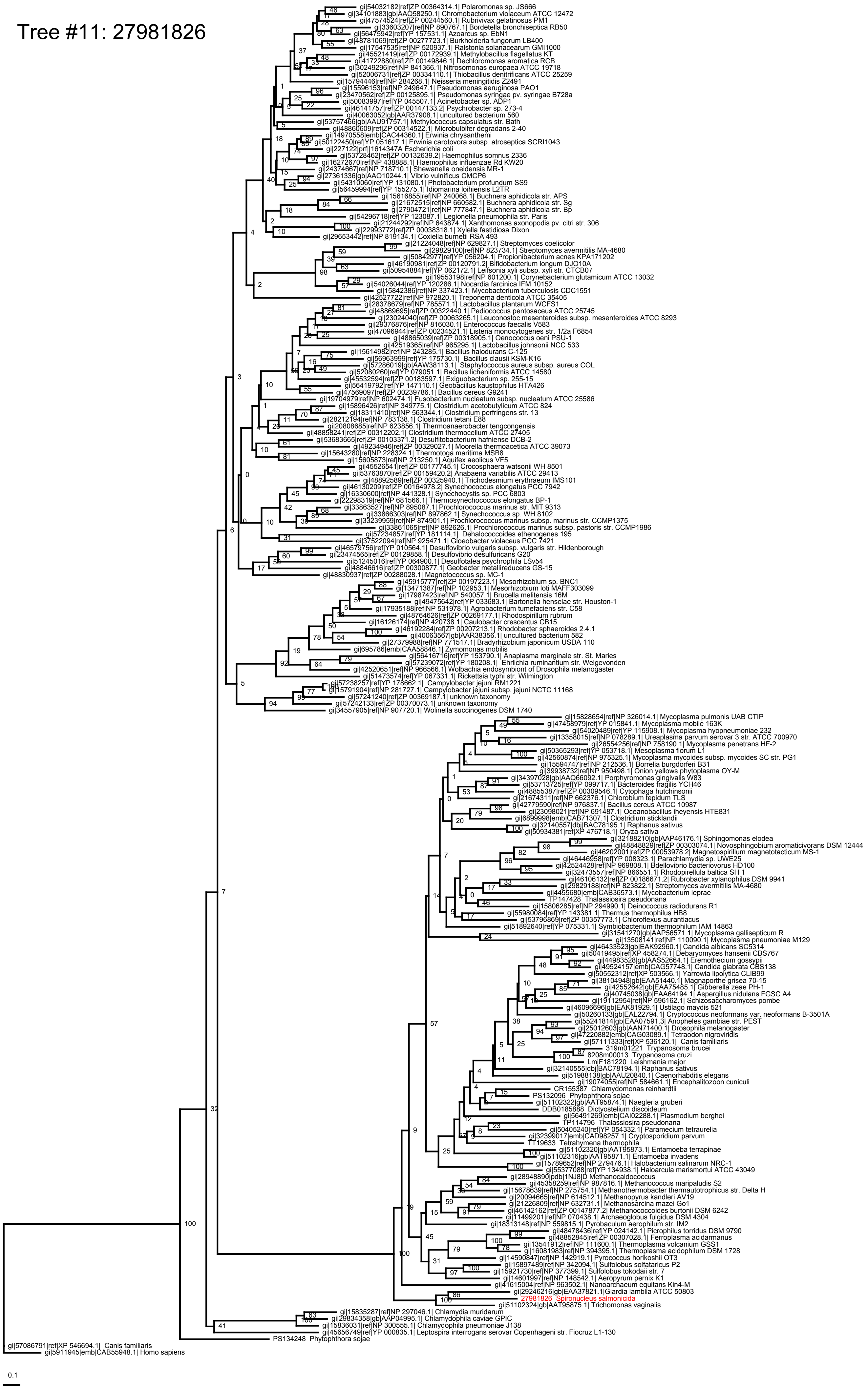

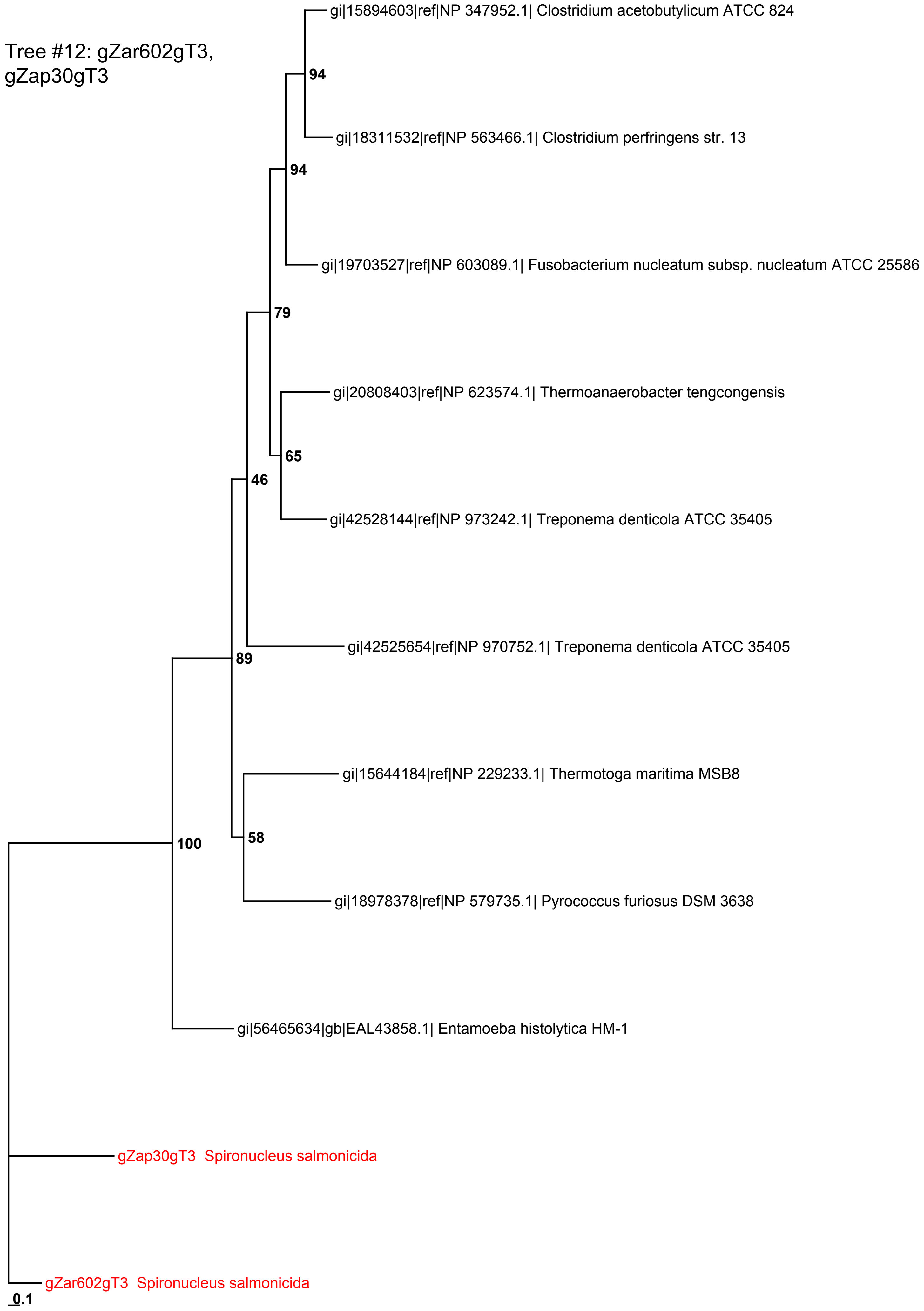
Tree #12: gZar602gT3, gZap30gT3
gi|15894603|ref|NP 347952.1| Clostridium acetobutylicum ATCC 824
94
gi|18311532|ref|NP 563466.1| Clostridium perfringens str. 13
94
gi|19703527|ref|NP 603089.1| Fusobacterium nucleatum subsp. nucleatum ATCC 25586
79
gi|20808403|ref|NP 623574.1| Thermoanaerobacter tengcongensis
65
46
gi|42528144|ref|NP 973242.1| Treponema denticola ATCC 35405
gi|42525654|ref|NP 970752.1| Treponema denticola ATCC 35405
89
gi|15644184|ref|NP 229233.1| Thermotoga maritima MSB8
58
100
gi|18978378|ref|NP 579735.1| Pyrococcus furiosus DSM 3638
gi|56465634|gb|EAL43858.1| Entamoeba histolytica HM-1
gZap30gT3 Spironucleus salmonicida
gZar602gT3 Spironucleus salmonicida
0.1

Tree #13: 27982357

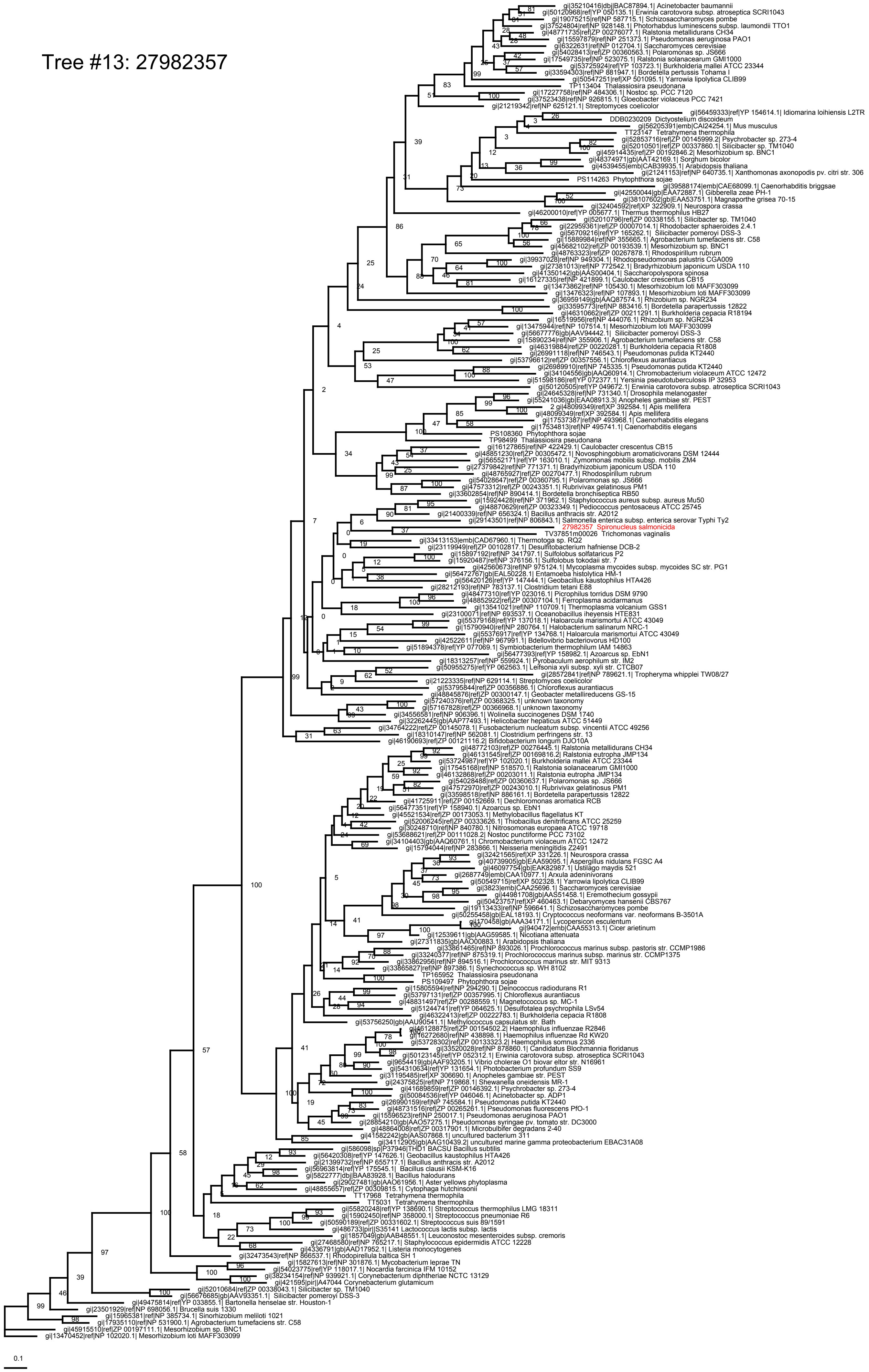

Tree #14: SpESTZap946

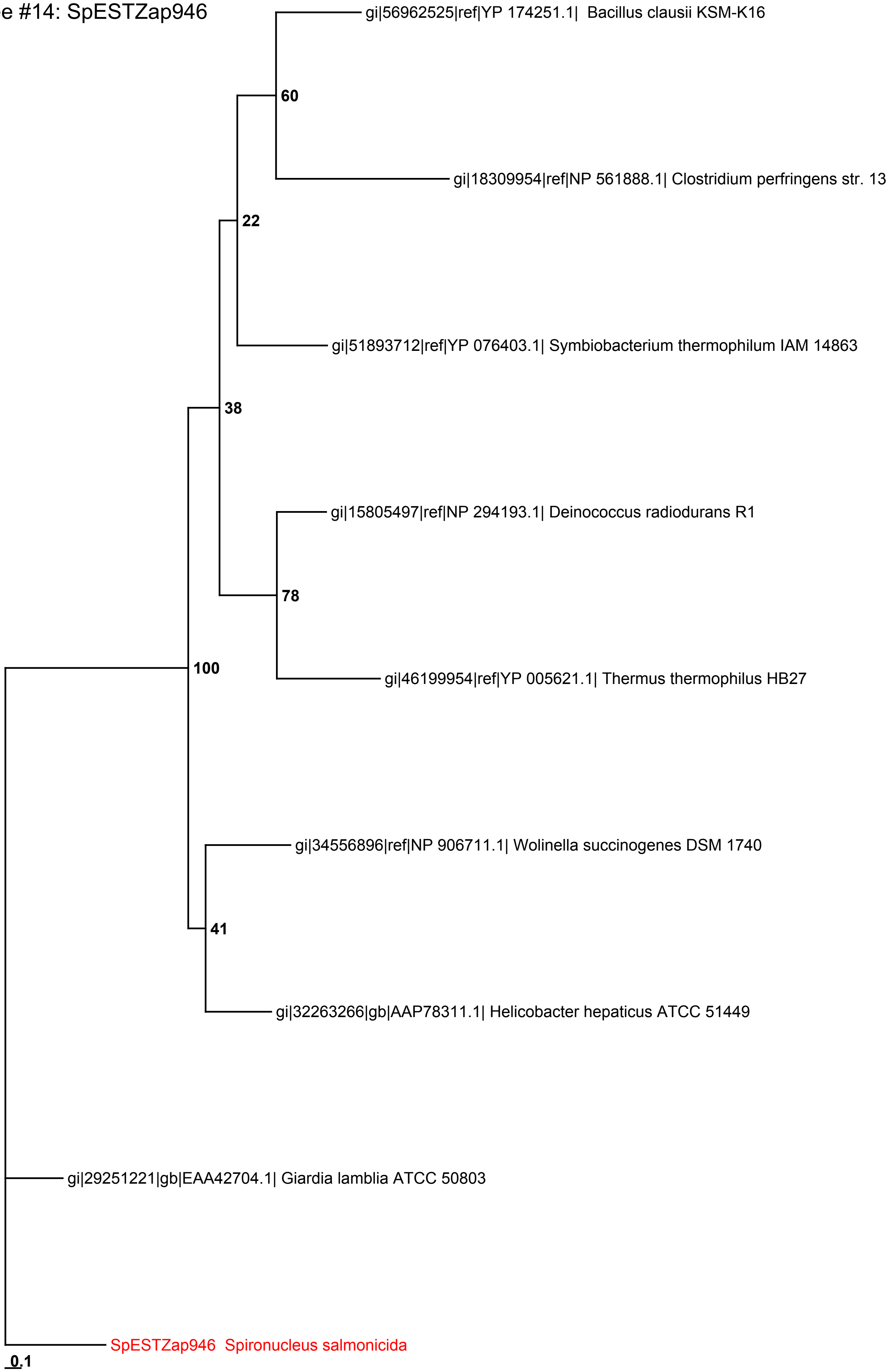

Tree #15: gZap84gT3

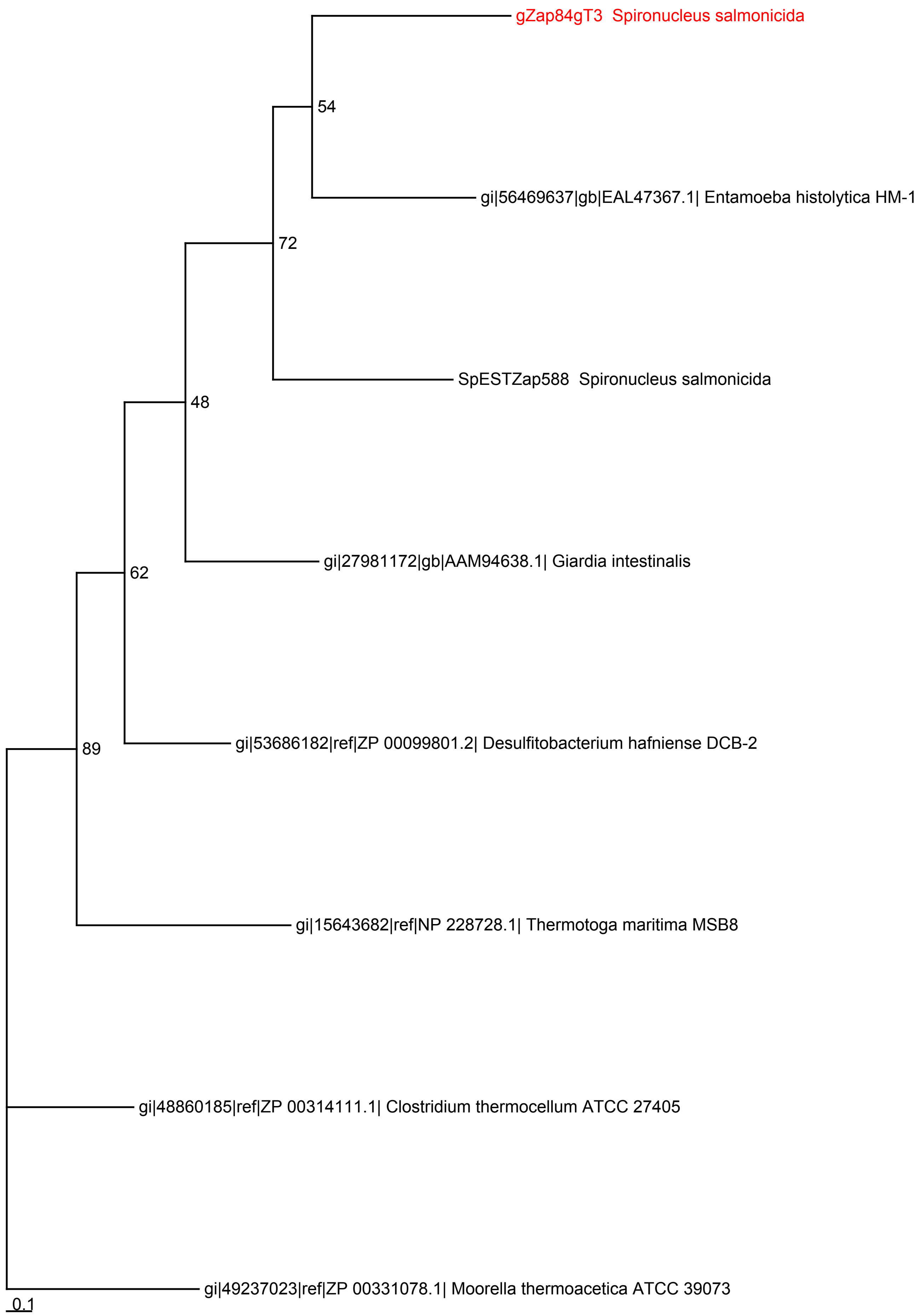

Tree #16: SpESTZap588, 27983189, 27983404

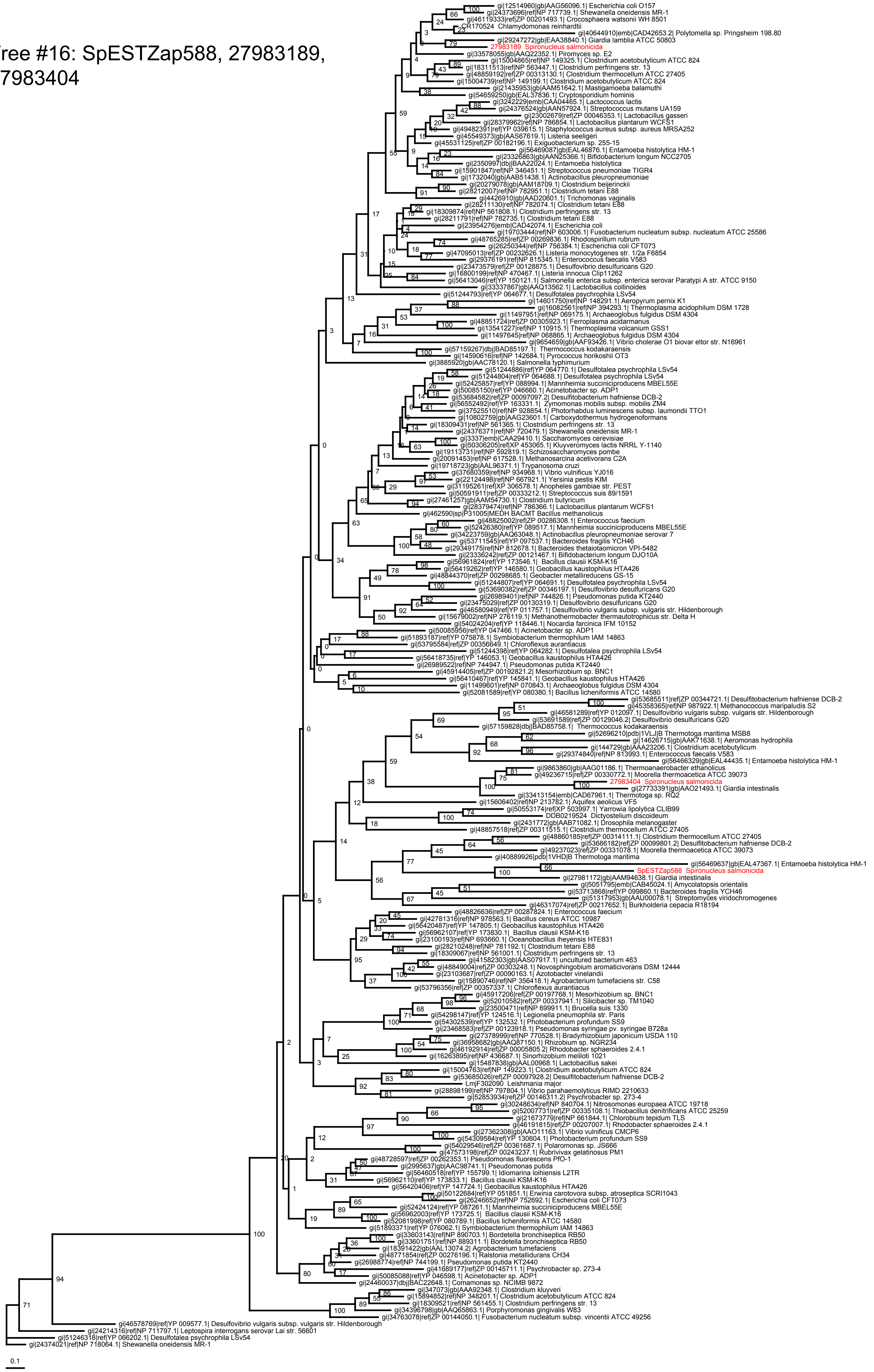

Tree 17: SpESTDH325

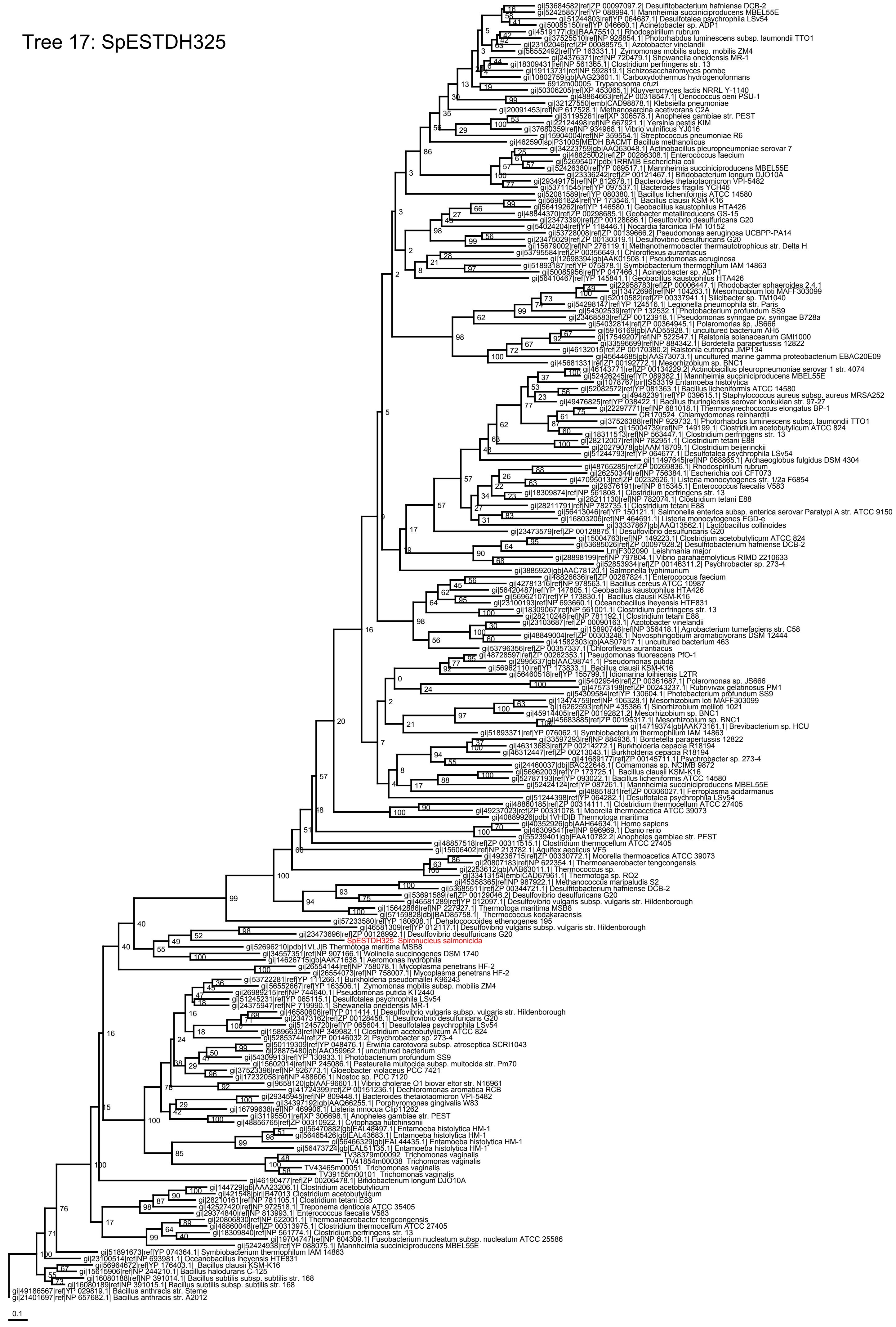

Tree #18: 23266714

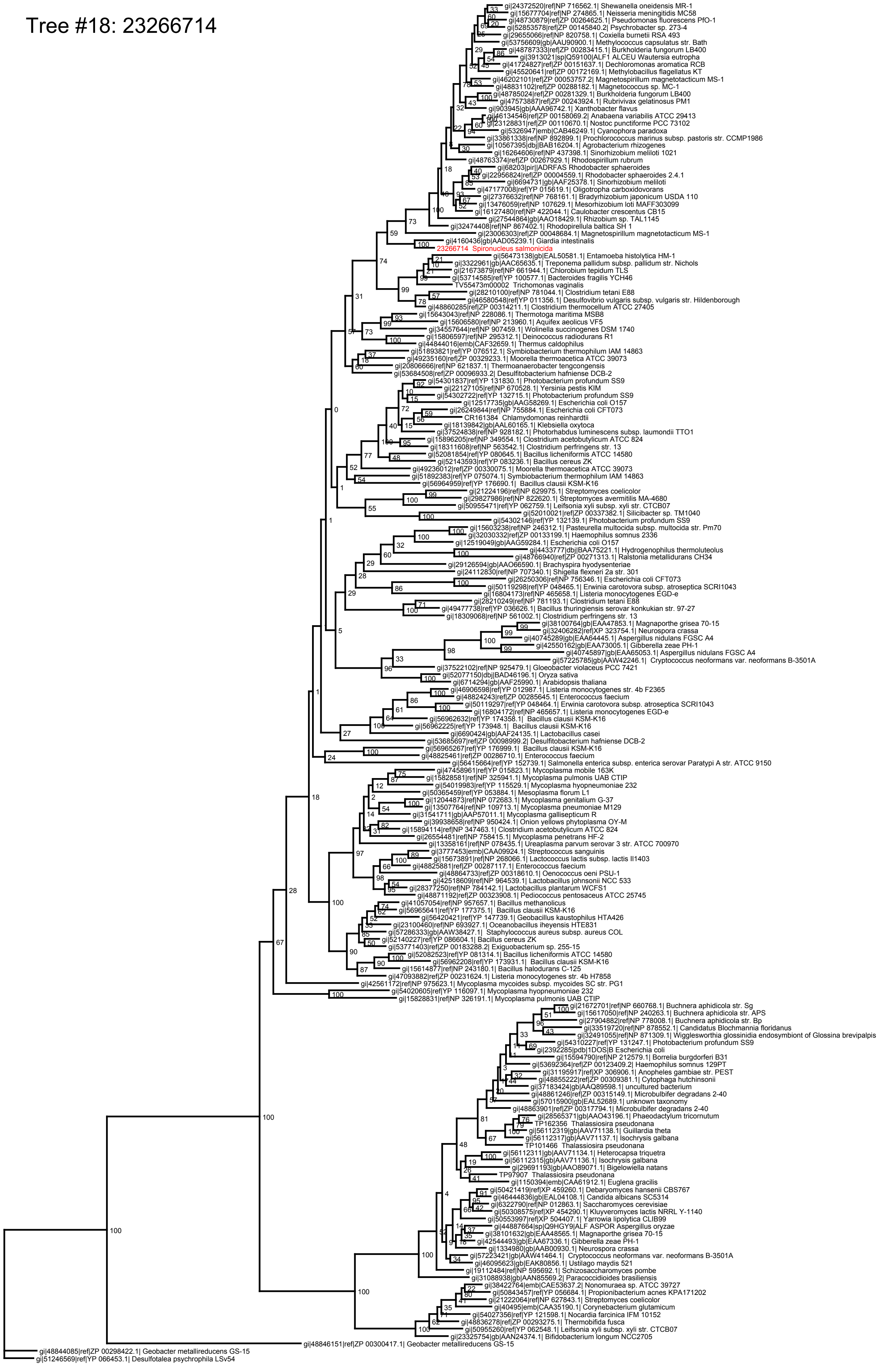

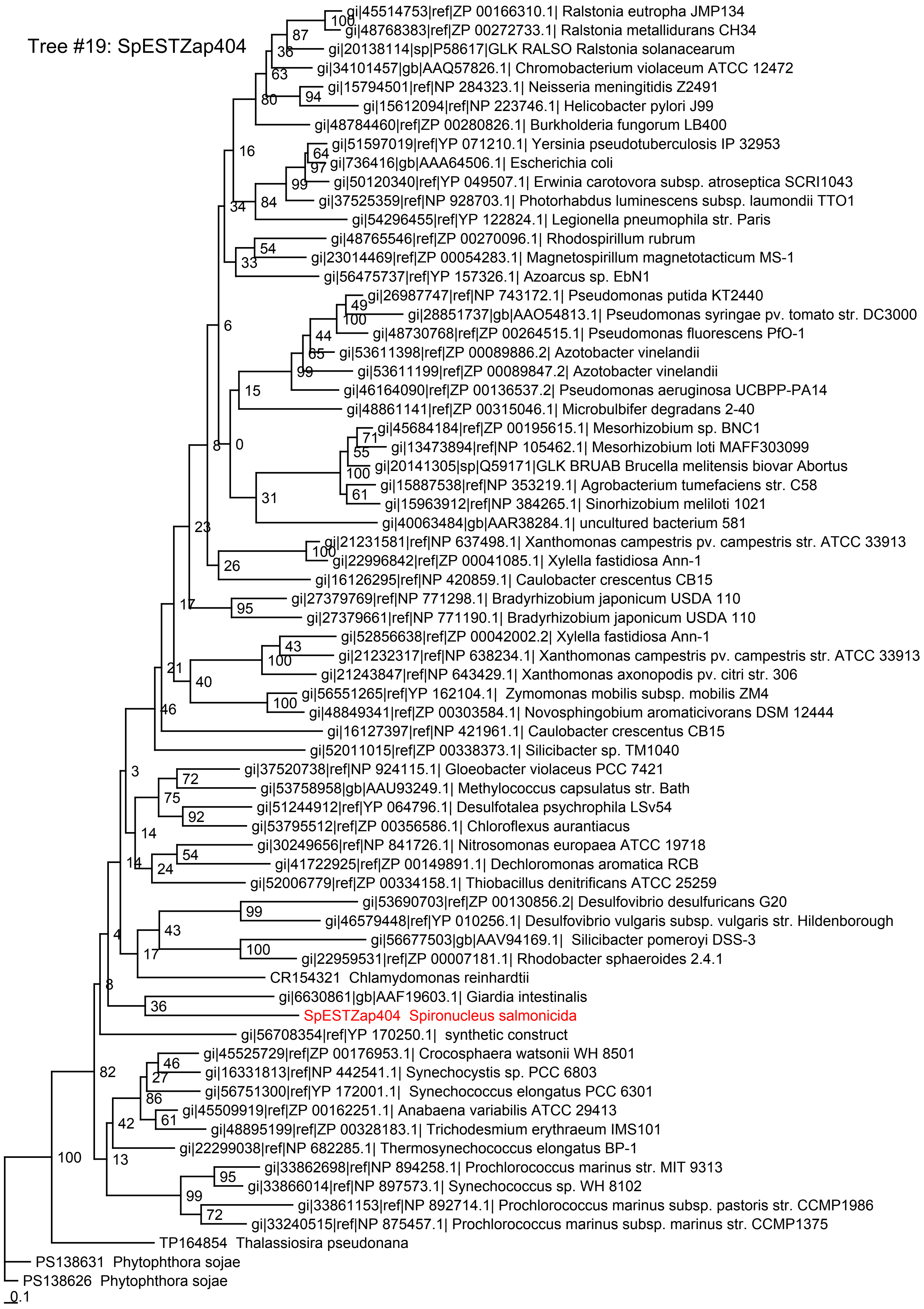
Tree #19: SpESTZap404
gi|45514753|ref|ZP 00166310.1| Ralstonia eutropha JMP134
gi|48768383|ref|ZP 00272733.1| Ralstonia metallidurans CH34
gi|20138114|sp|P58617|GLK RALSO Ralstonia solanacearum
gi|34101457|gb|AAQ57826.1| Chromobacterium violaceum ATCC 12472
gi|15794501|ref|NP 284323.1| Neisseria meningitidis Z2491
gi|15612094|ref|NP 223746.1| Helicobacter pylori J99
gi|48784460|ref|ZP 00280826.1| Burkholderia fungorum LB400
gi|51597019|ref|YP 071210.1| Yersinia pseudotuberculosis IP 32953
gi|736416|gb|AAA64506.1| Escherichia coli
gi|50120340|ref|YP 049507.1| Erwinia carotovora subsp. atroseptica SCRI1043
gi|37525359|ref|NP 928703.1| Photorhabdus luminescens subsp. laumondii TTO1
gi|54296455|ref|YP 122824.1| Legionella pneumophila str. Paris
gi|48765546|ref|ZP 00270096.1| Rhodospirillum rubrum
gi|23014469|ref|ZP 00054283.1| Magnetospirillum magnetotacticum MS-1
gi|56475737|ref|YP 157326.1| Azoarcus sp. EbN1
gi|26987747|ref|NP 743172.1| Pseudomonas putida KT2440
gi|28851737|gb|AAO54813.1| Pseudomonas syringae pv. tomato str. DC3000
gi|48730768|ref|ZP 00264515.1| Pseudomonas fluorescens PfO-1
gi|53611398|ref|ZP 00089886.2| Azotobacter vinelandii
gi|53611199|ref|ZP 00089847.2| Azotobacter vinelandii
gi|46164090|ref|ZP 00136537.2| Pseudomonas aeruginosa UCBPP-PA14
gi|48861141|ref|ZP 00315046.1| Microbulbifer degradans 2-40
gi|45684184|ref|ZP 00195615.1| Mesorhizobium sp. BNC1
gi|13473894|ref|NP 105462.1| Mesorhizobium loti MAFF303099
gi|20141305|sp|Q59171|GLK BRUAB Brucella melitensis biovar Abortus
gi|15887538|ref|NP 353219.1| Agrobacterium tumefaciens str. C58
gi|15963912|ref|NP 384265.1| Sinorhizobium meliloti 1021
gi|40063484|gb|AAR38284.1| uncultured bacterium 581
gi|21231581|ref|NP 637498.1| Xanthomonas campestris pv. campestris str. ATCC 33913
gi|22996842|ref|ZP 00041085.1| Xylella fastidiosa Ann-1
gi|16126295|ref|NP 420859.1| Caulobacter crescentus CB15
gi|27379769|ref|NP 771298.1| Bradyrhizobium japonicum USDA 110
gi|27379661|ref|NP 771190.1| Bradyrhizobium japonicum USDA 110
gi|52856638|ref|ZP 00042002.2| Xylella fastidiosa Ann-1
gi|21232317|ref|NP 638234.1| Xanthomonas campestris pv. campestris str. ATCC 33913
gi|21243847|ref|NP 643429.1| Xanthomonas axonopodis pv. citri str. 306
gi|56551265|ref|YP 162104.1| Zymomonas mobilis subsp. mobilis ZM4
gi|48849341|ref|ZP 00303584.1| Novosphingobium aromaticivorans DSM 12444
gi|16127397|ref|NP 421961.1| Caulobacter crescentus CB15
gi|52011015|ref|ZP 00338373.1| Silicibacter sp. TM1040
gi|37520738|ref|NP 924115.1| Gloeobacter violaceus PCC 7421
gi|53758958|gb|AAU93249.1| Methylococcus capsulatus str. Bath
gi|51244912|ref|YP 064796.1| Desulfotalea psychrophila LSv54
gi|53795512|ref|ZP 00356586.1| Chloroflexus aurantiacus
gi|30249656|ref|NP 841726.1| Nitrosomonas europaea ATCC 19718
gi|41722925|ref|ZP 00149891.1| Dechloromonas aromatica RCB
gi|52006779|ref|ZP 00334158.1| Thiobacillus denitrificans ATCC 25259
gi|53690703|ref|ZP 00130856.2| Desulfovibrio desulfuricans G20
gi|46579448|ref|YP 010256.1| Desulfovibrio vulgaris subsp. vulgaris str. Hildenborough
gi|56677503|gb|AAV94169.1| Silicibacter pomeroyi DSS-3
gi|22959531|ref|ZP 00007181.1| Rhodobacter sphaeroides 2.4.1
CR154321 Chlamydomonas reinhardtii
gi|6630861|gb|AAF19603.1| Giardia intestinalis
SpESTZap404 Spironucleus salmonicida
gi|56708354|ref|YP 170250.1| synthetic construct
gi|45525729|ref|ZP 00176953.1| Crocosphaera watsonii WH 8501
gi|16331813|ref|NP 442541.1| Synechocystis sp. PCC 6803
gi|56751300|ref|YP 172001.1| Synechococcus elongatus PCC 6301
gi|45509919|ref|ZP 00162251.1| Anabaena variabilis ATCC 29413
gi|48895199|ref|ZP 00328183.1| Trichodesmium erythraeum IMS101
gi|22299038|ref|NP 682285.1| Thermosynechococcus elongatus BP-1
gi|33862698|ref|NP 894258.1| Prochlorococcus marinus str. MIT 9313
gi|33866014|ref|NP 897573.1| Synechococcus sp. WH 8102
gi|33861153|ref|NP 892714.1| Prochlorococcus marinus subsp. pastoris str. CCMP1986
gi|33240515|ref|NP 875457.1| Prochlorococcus marinus subsp. marinus str. CCMP1375
TP164854 Thalassiosira pseudonana
PS138631 Phytophthora sojae
PS138626 Phytophthora sojae
0.1

Tree #20: 18030018

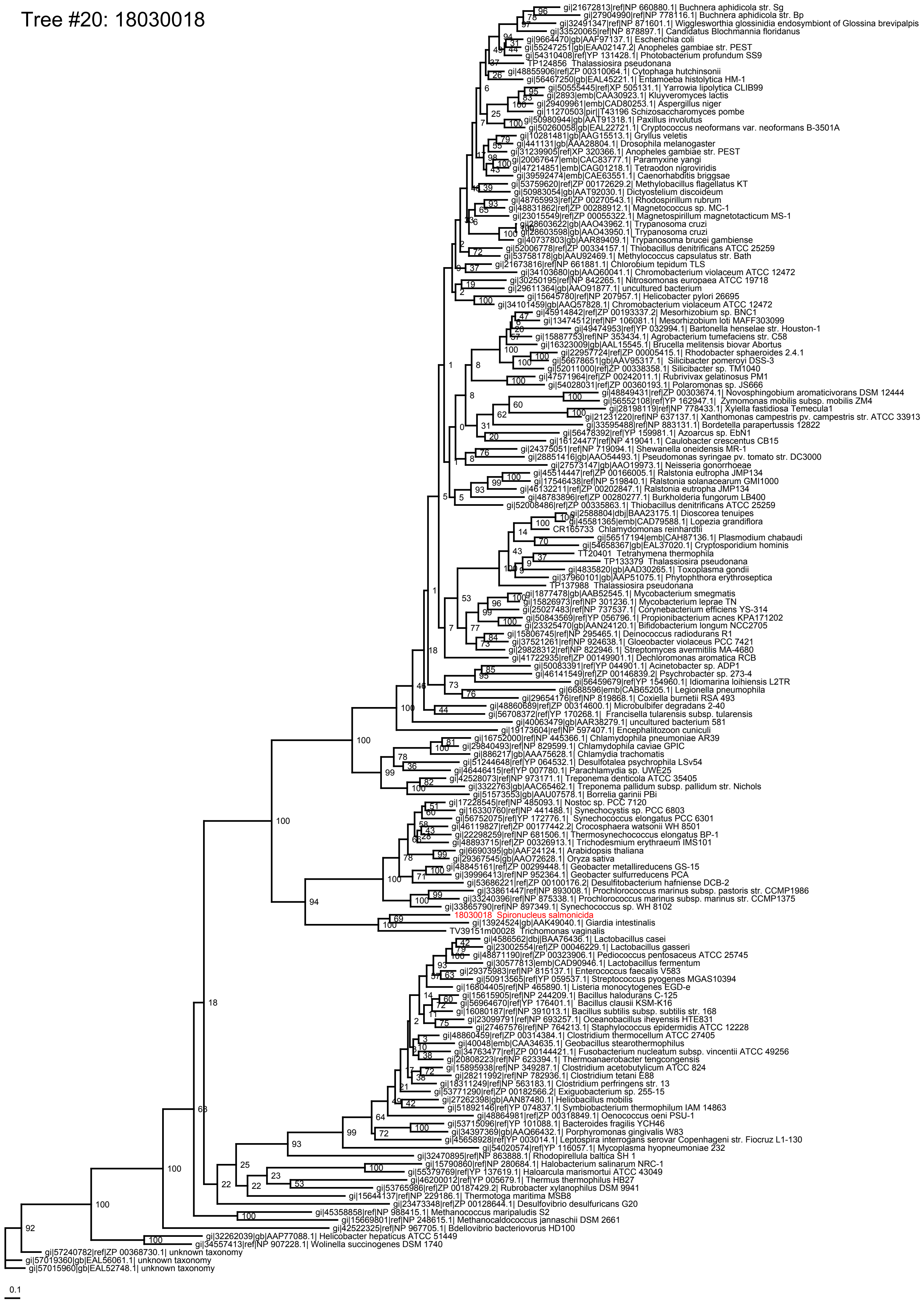

Tree #21: gTol047bMF

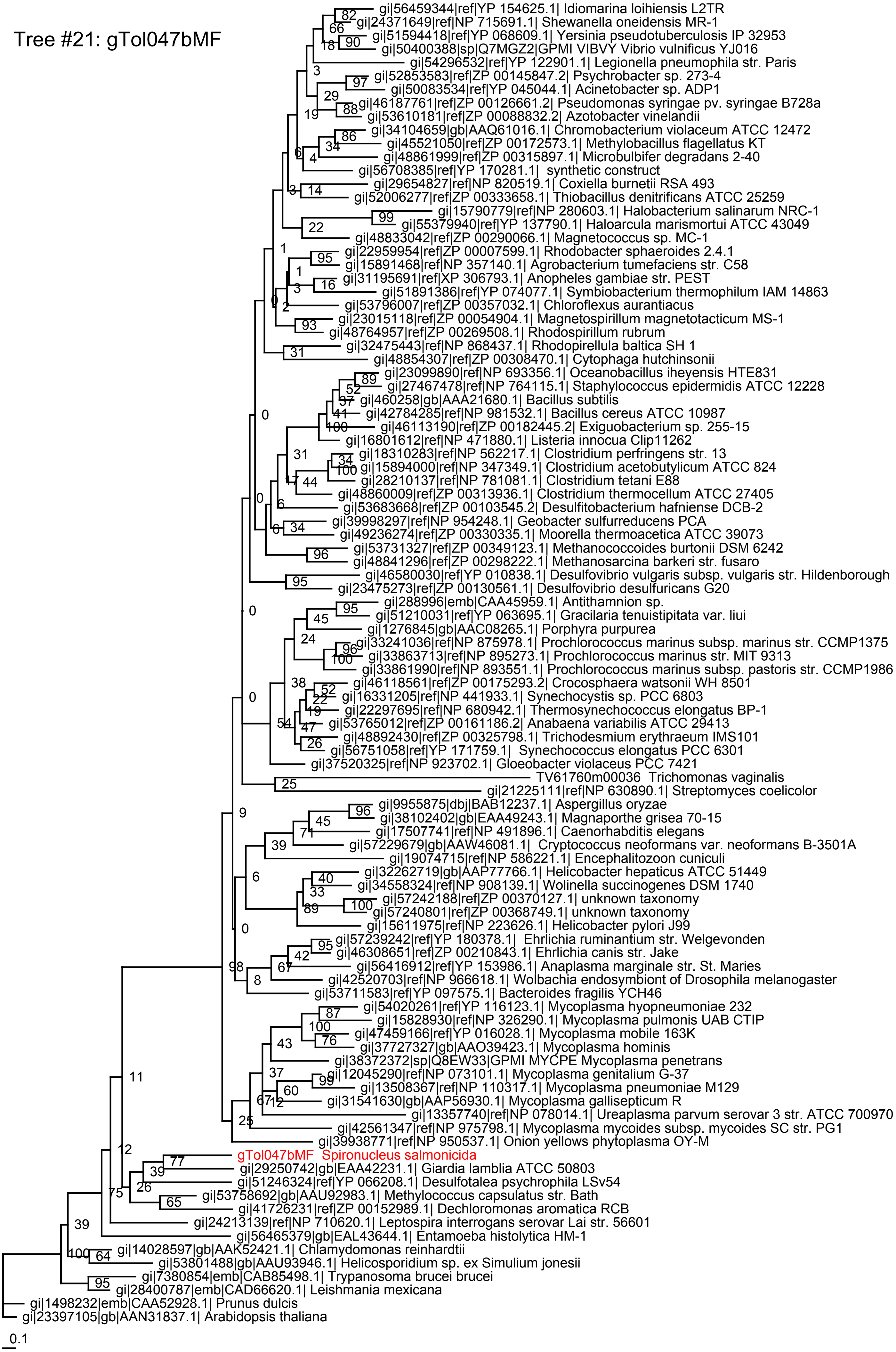

Tree #22: SpESTC275

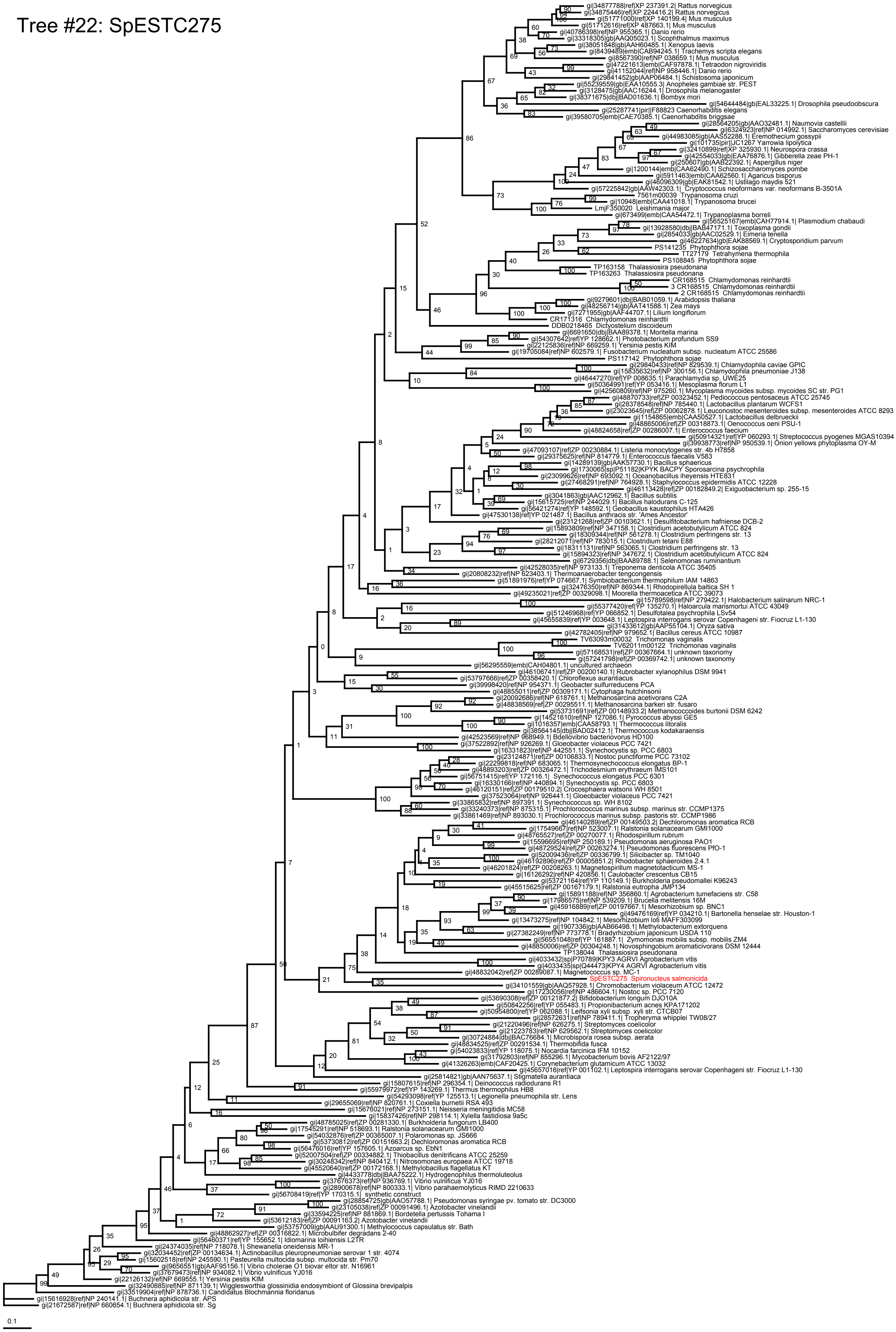

Tree #23: gTor983gT3

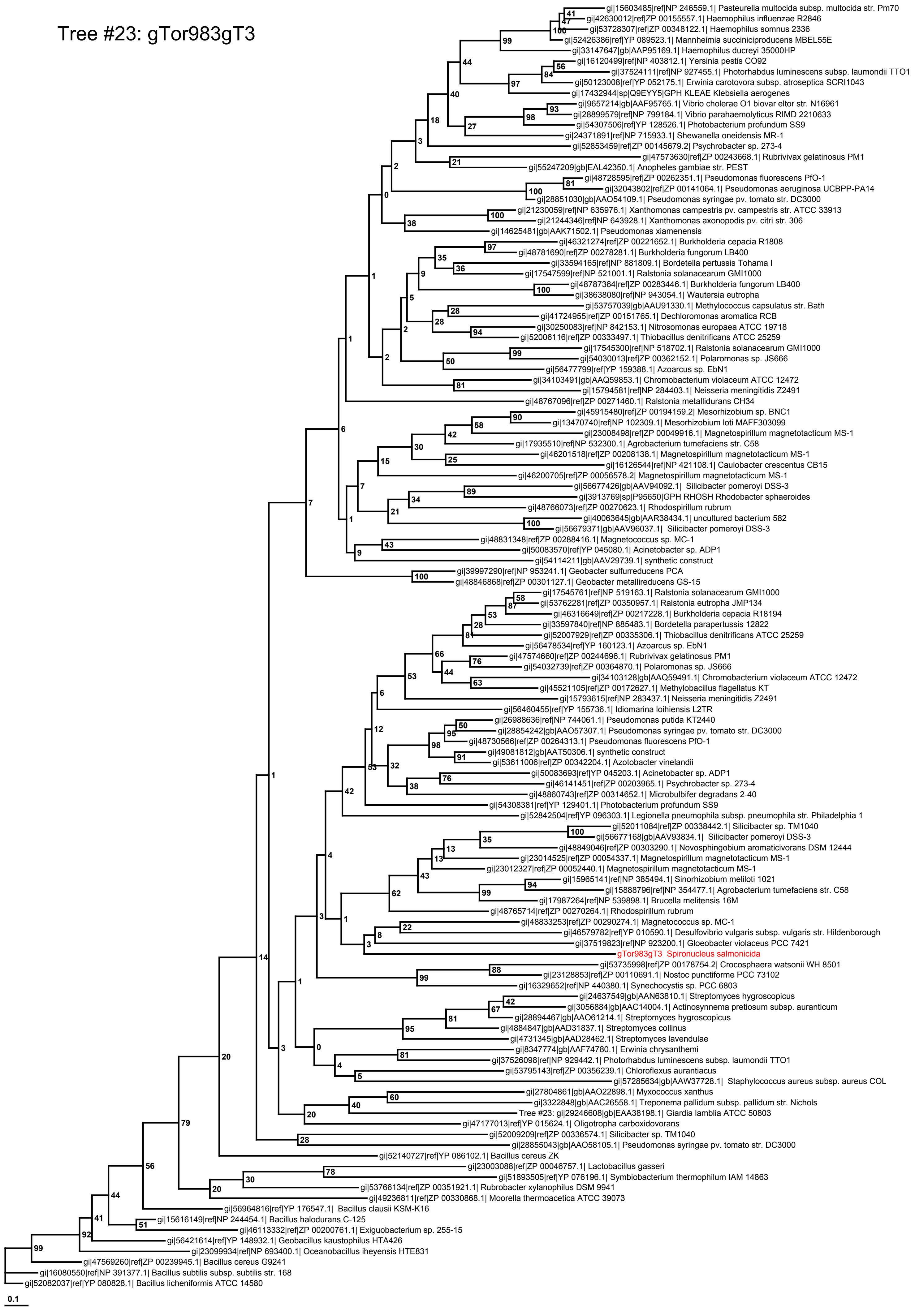

Tree #24: SpESTC23

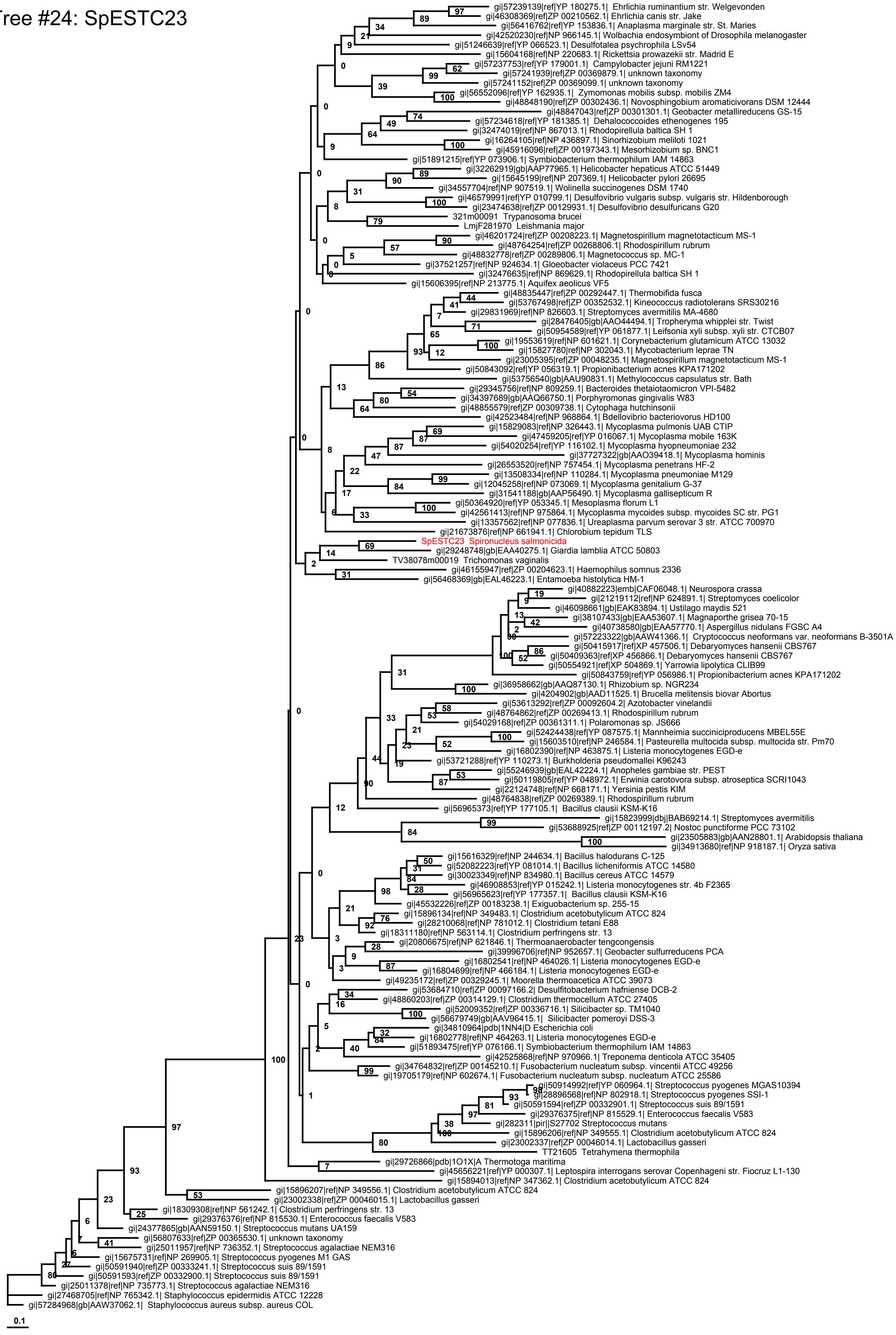

Tree #25: SpESTZap360

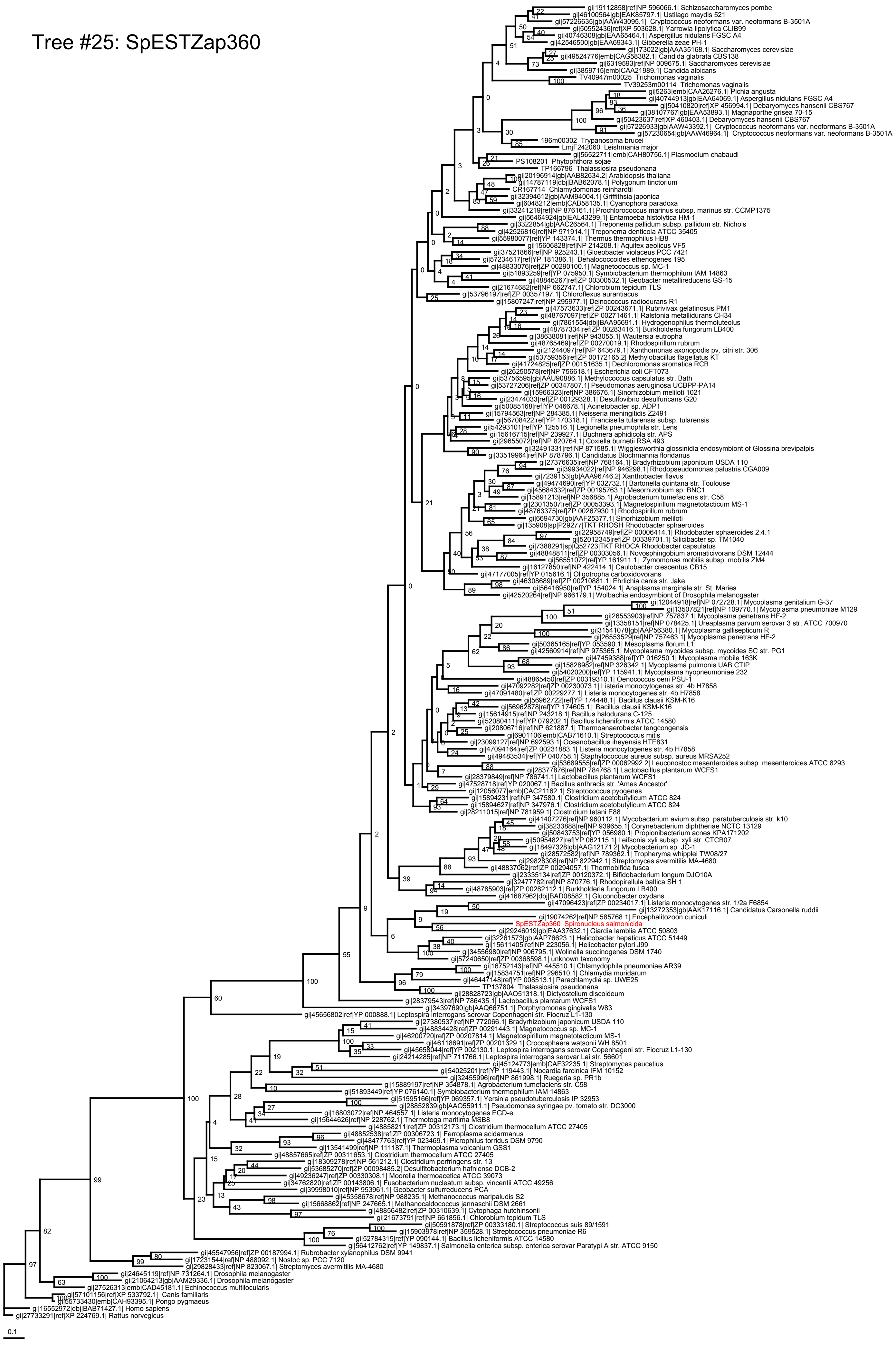

Supplement: Additional file 4 — Phylogenetic trees 1–25 for genes putatively involved in LGT events and listed in Additional file 3. [file 1471-2164-8-51-S4.pdf]
