# Supplementary material for: A genomic survey of the fish parasite Spironucleus salmonicida indicates genomic plasticity among diplomonads and significant lateral gene transfer in eukaryote genome evolution
Source: BMC Genomics. 2007 Feb 14;8:51. doi: 10.1186/1471-2164-8-51 (PMC1805757; doi:10.1186/1471-2164-8-51)

Additional file 5 - Andersson *et al.*

Phylogenetic trees 26-50 for genes putatively involved in LGT events and listed in Additional file 3.

Tree #26: gZap797bT7

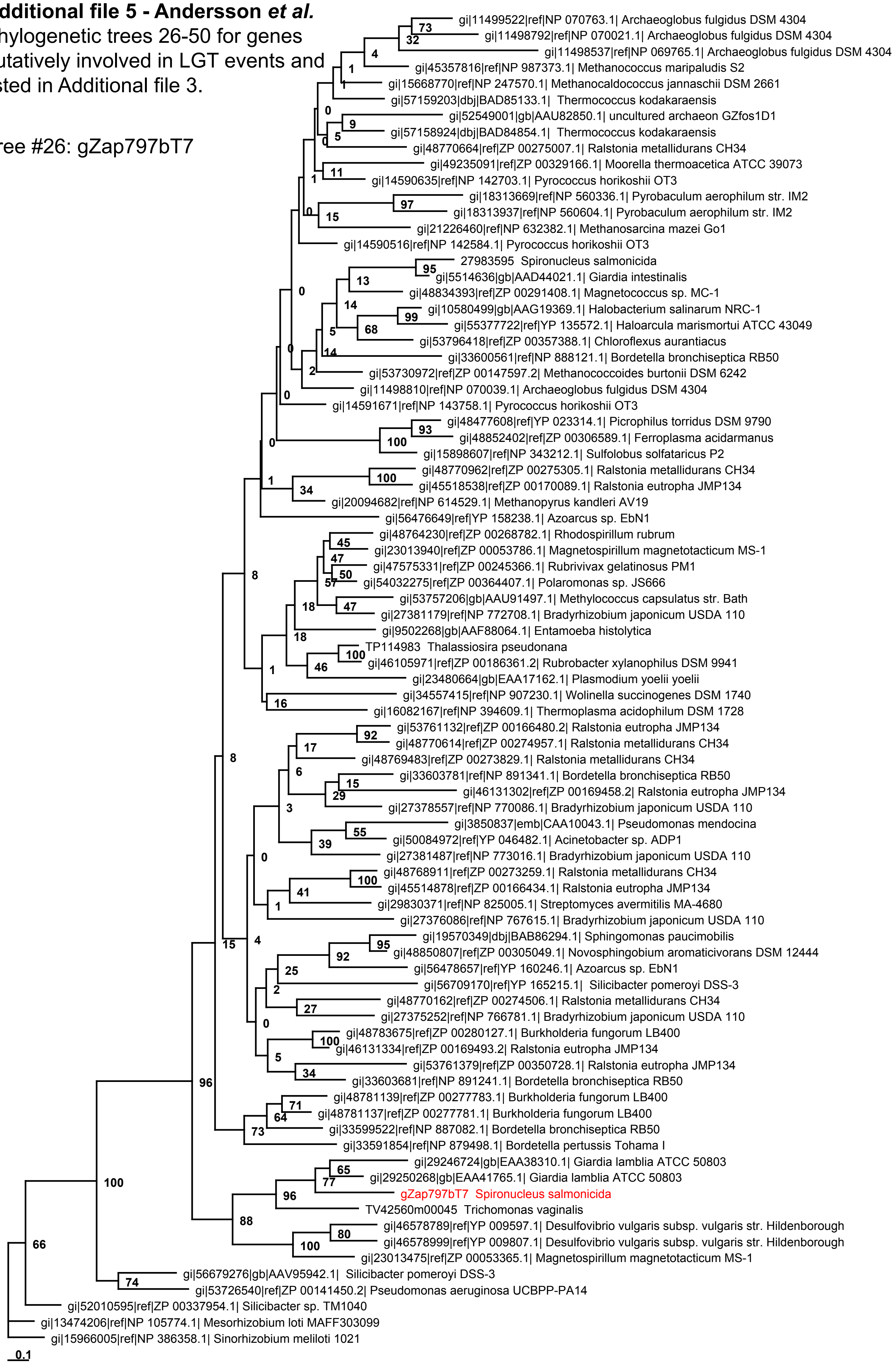

Tree #27: gZap472bT7, SpESTZap1028

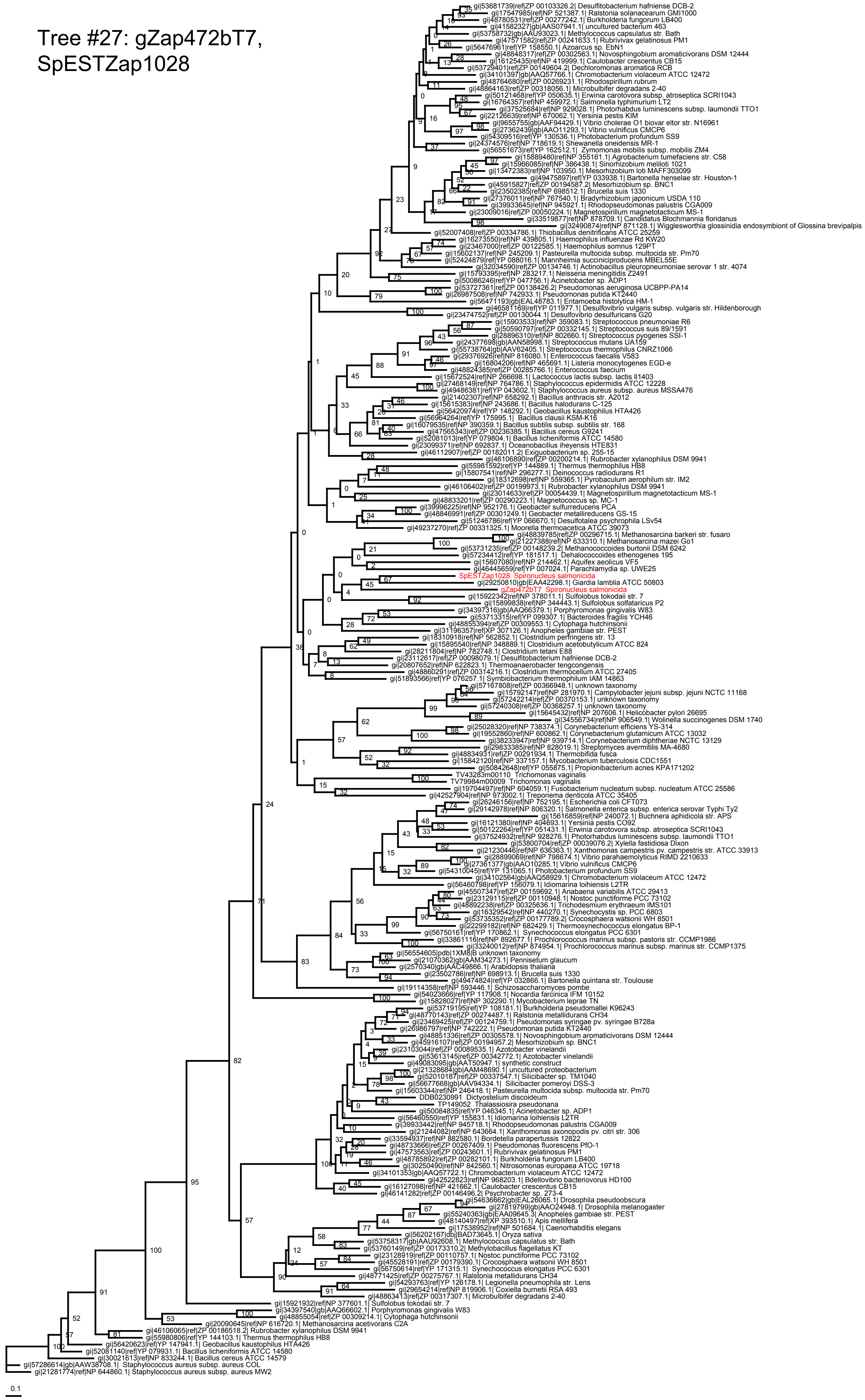

Tree #28: SpESTC94

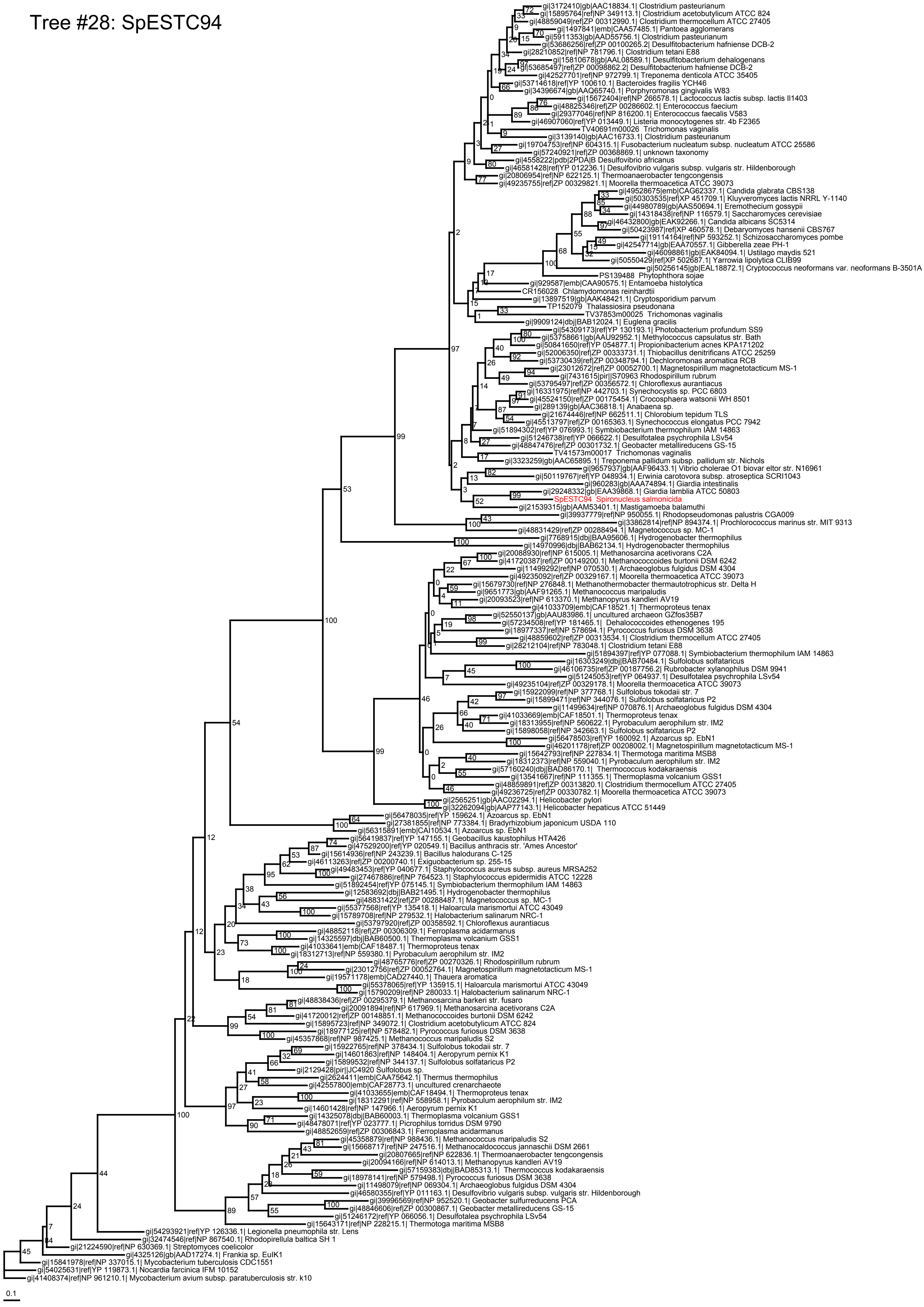

Tree #29: gZap510gT3

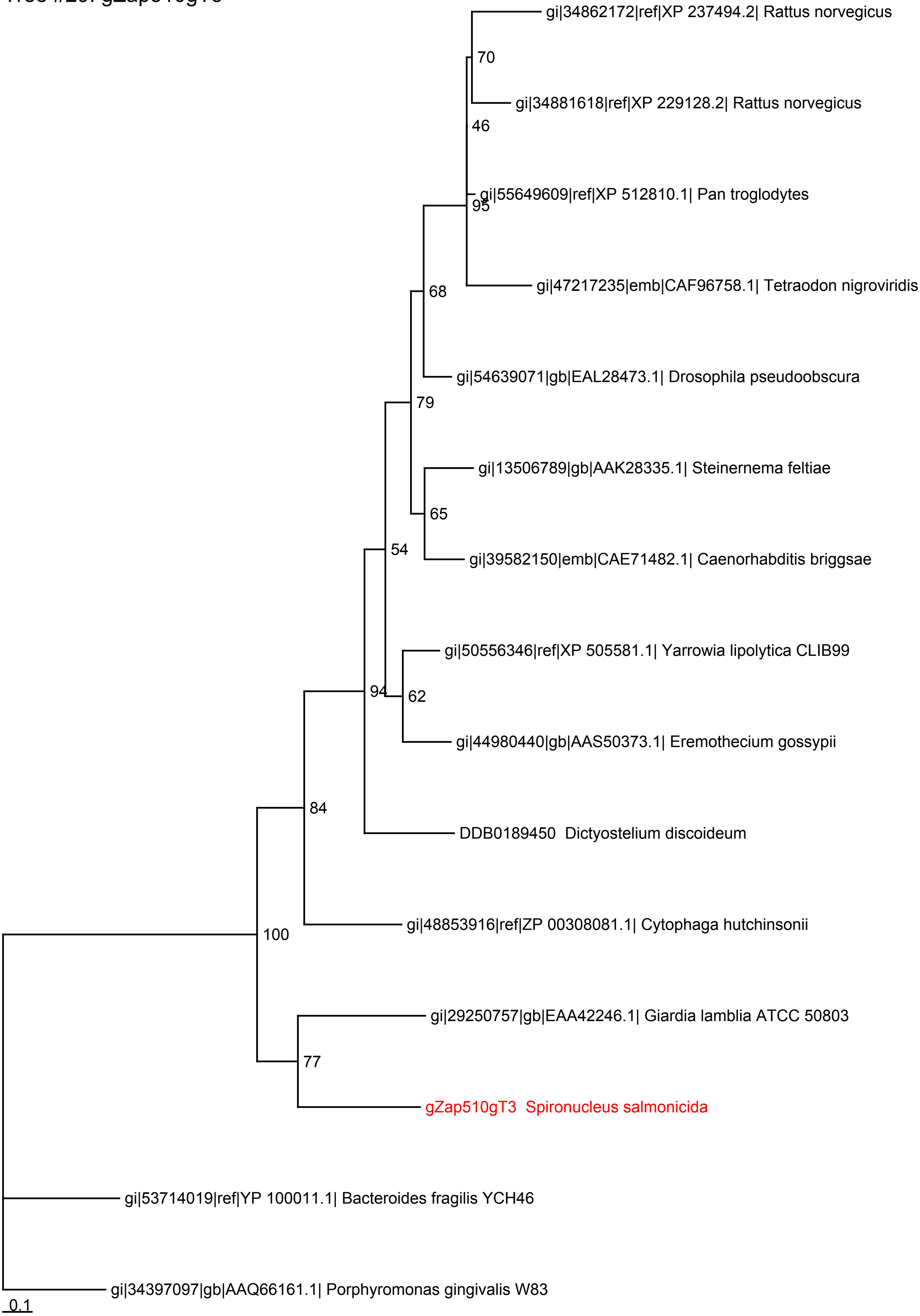

Tree #30: SpESTC239

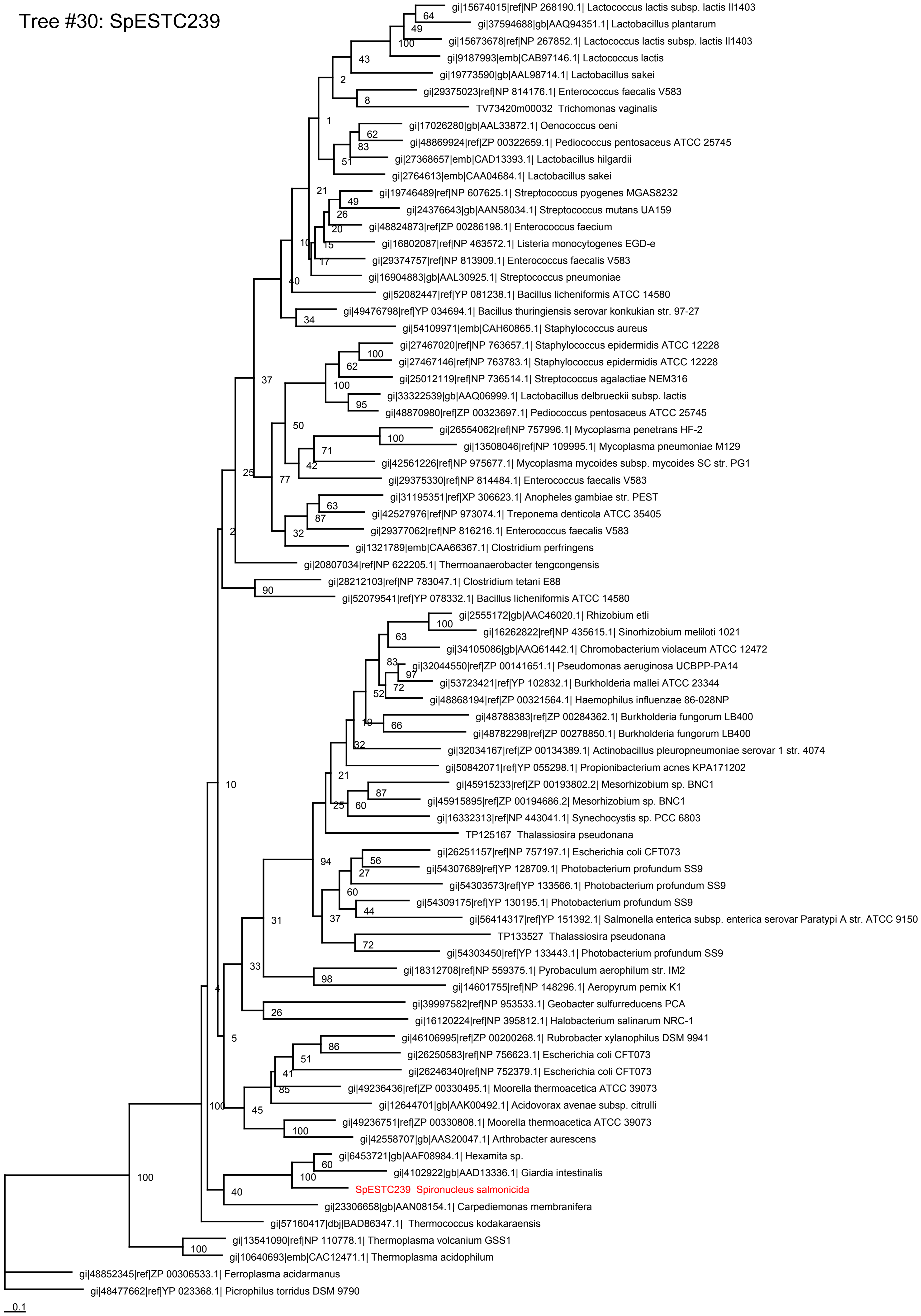

Tree #31: gZap126bT7

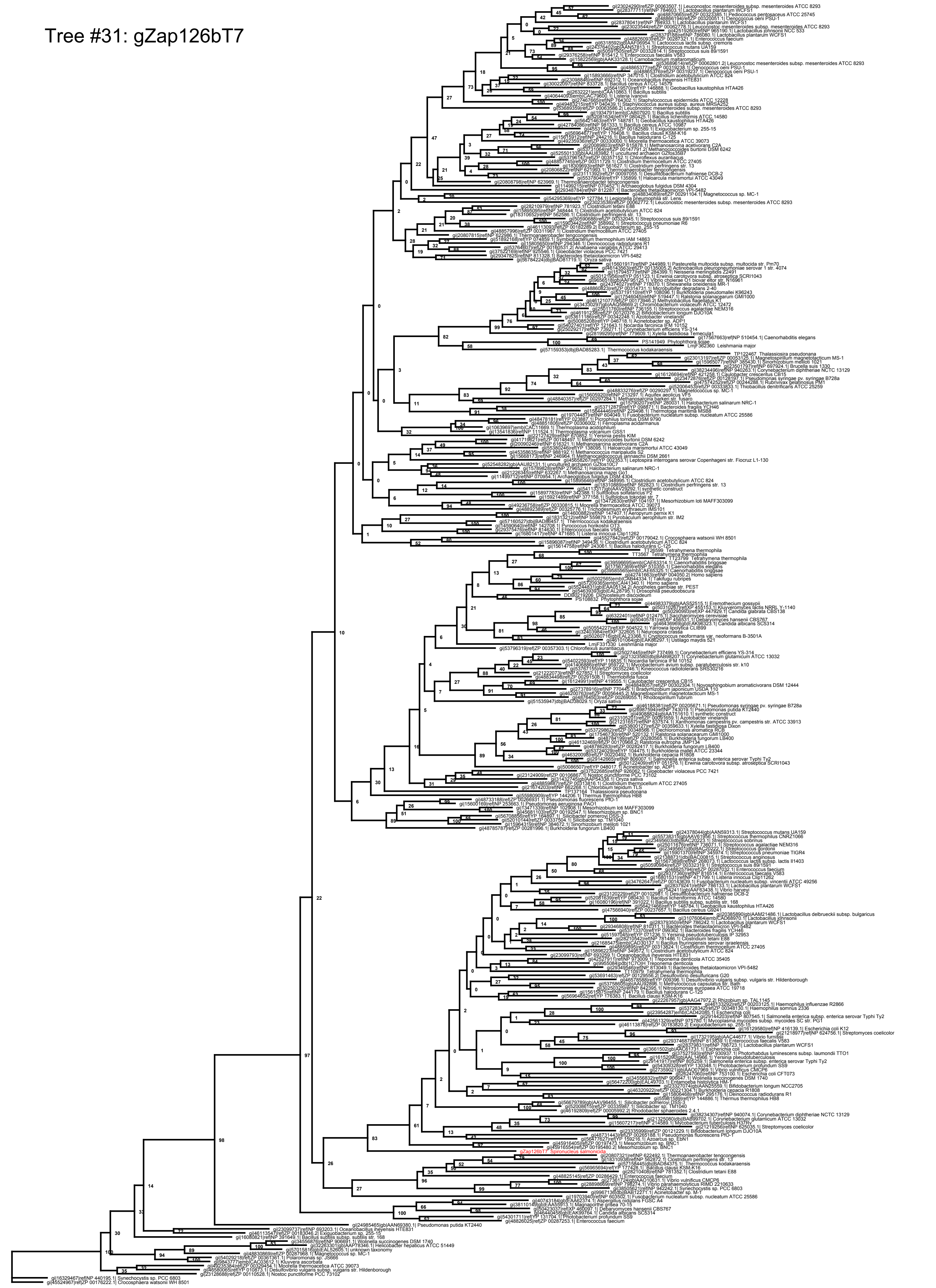

Tree #32: gZap126gT3

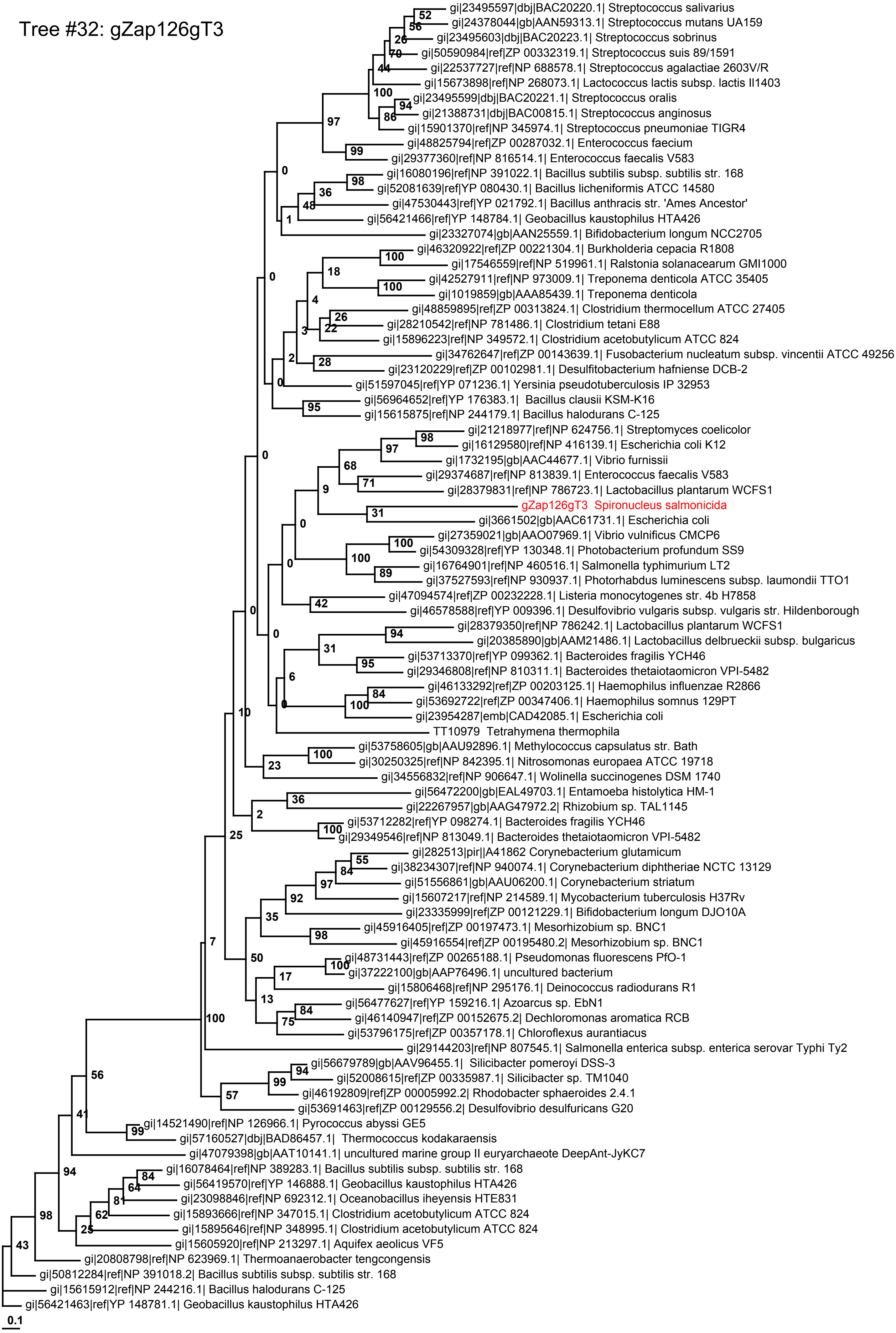

Tree #33: SpESTC259, SpEST1299, SpEST1913

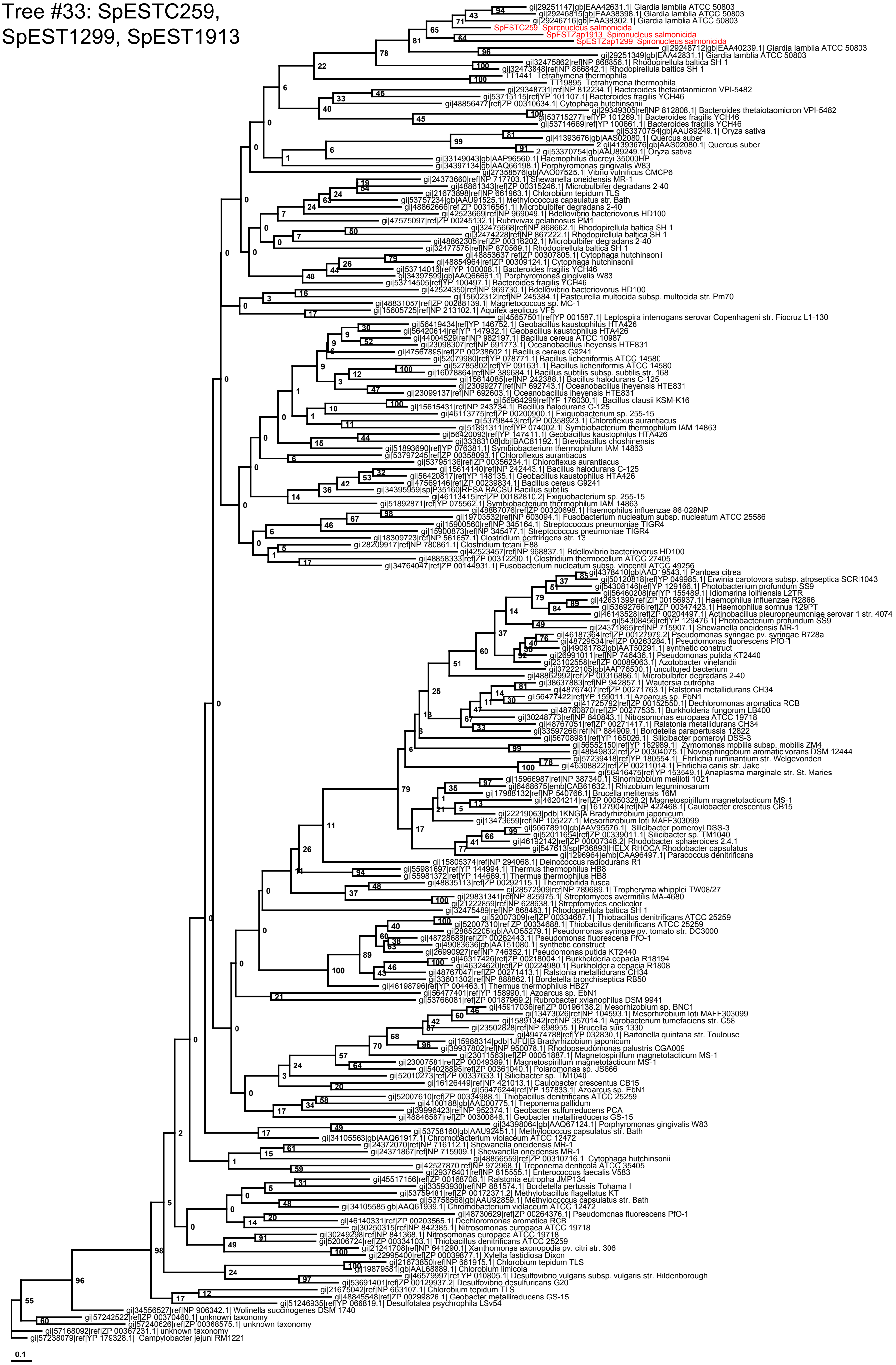

Tree #34: 20530910

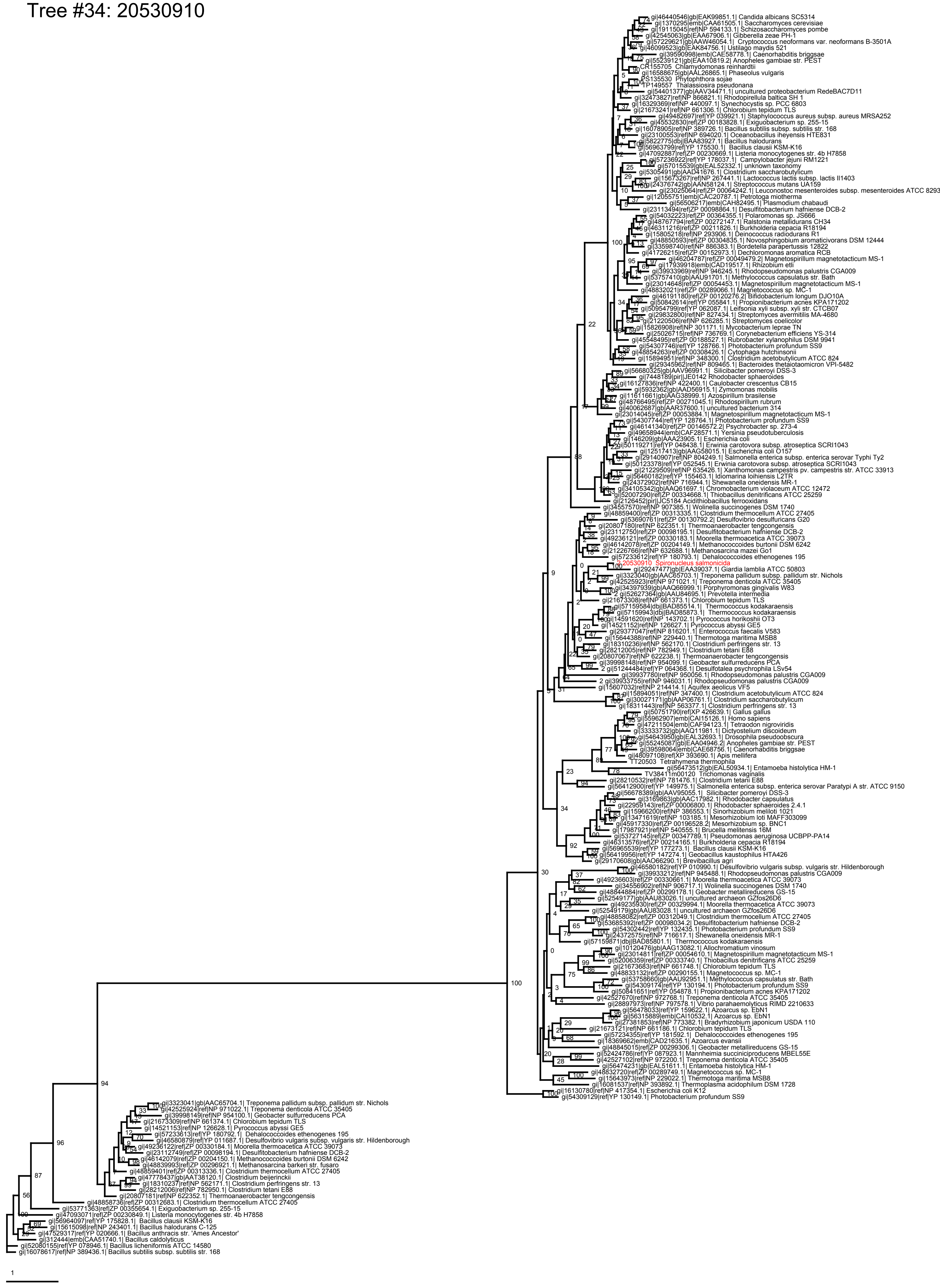

Tree #35: 27983817

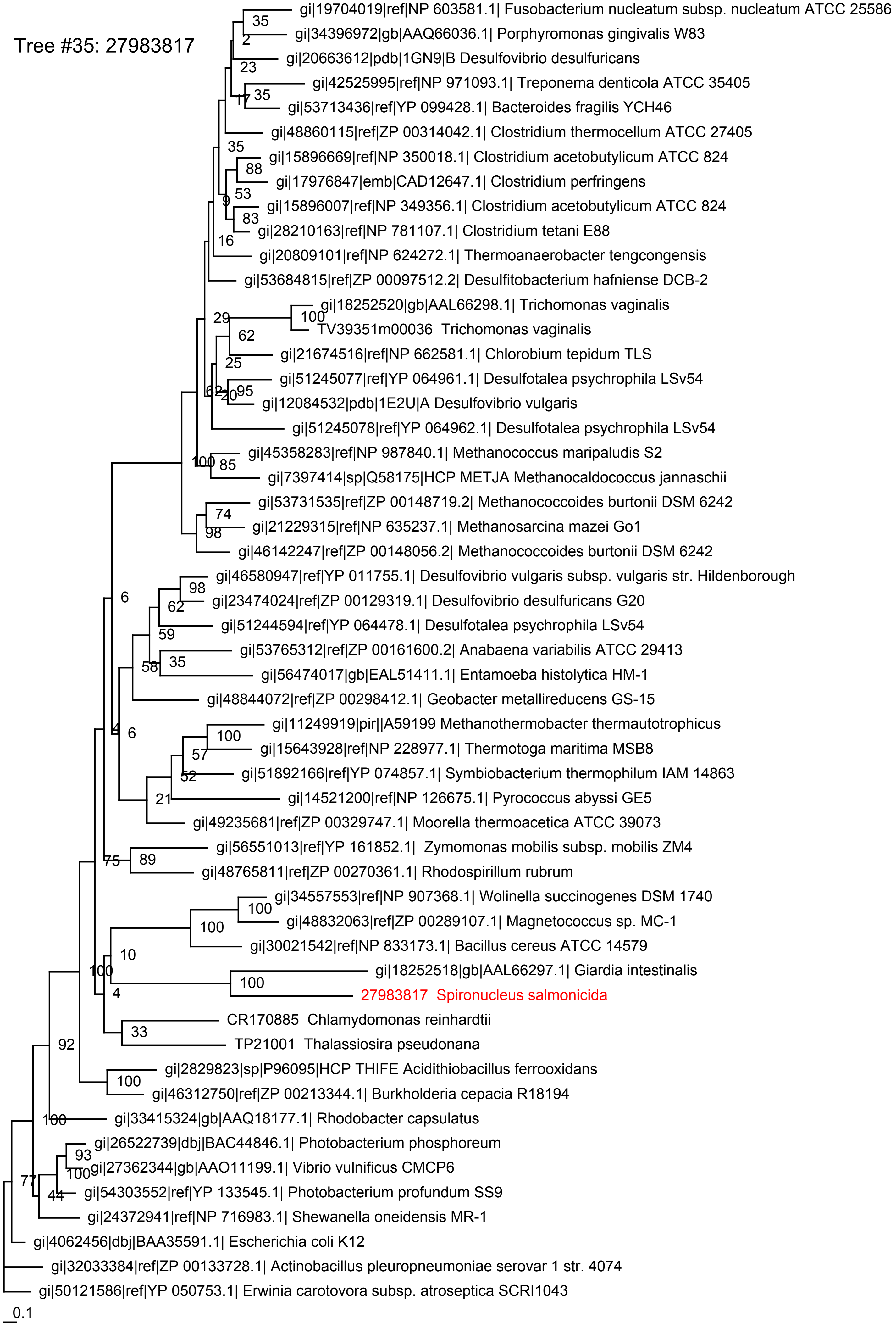

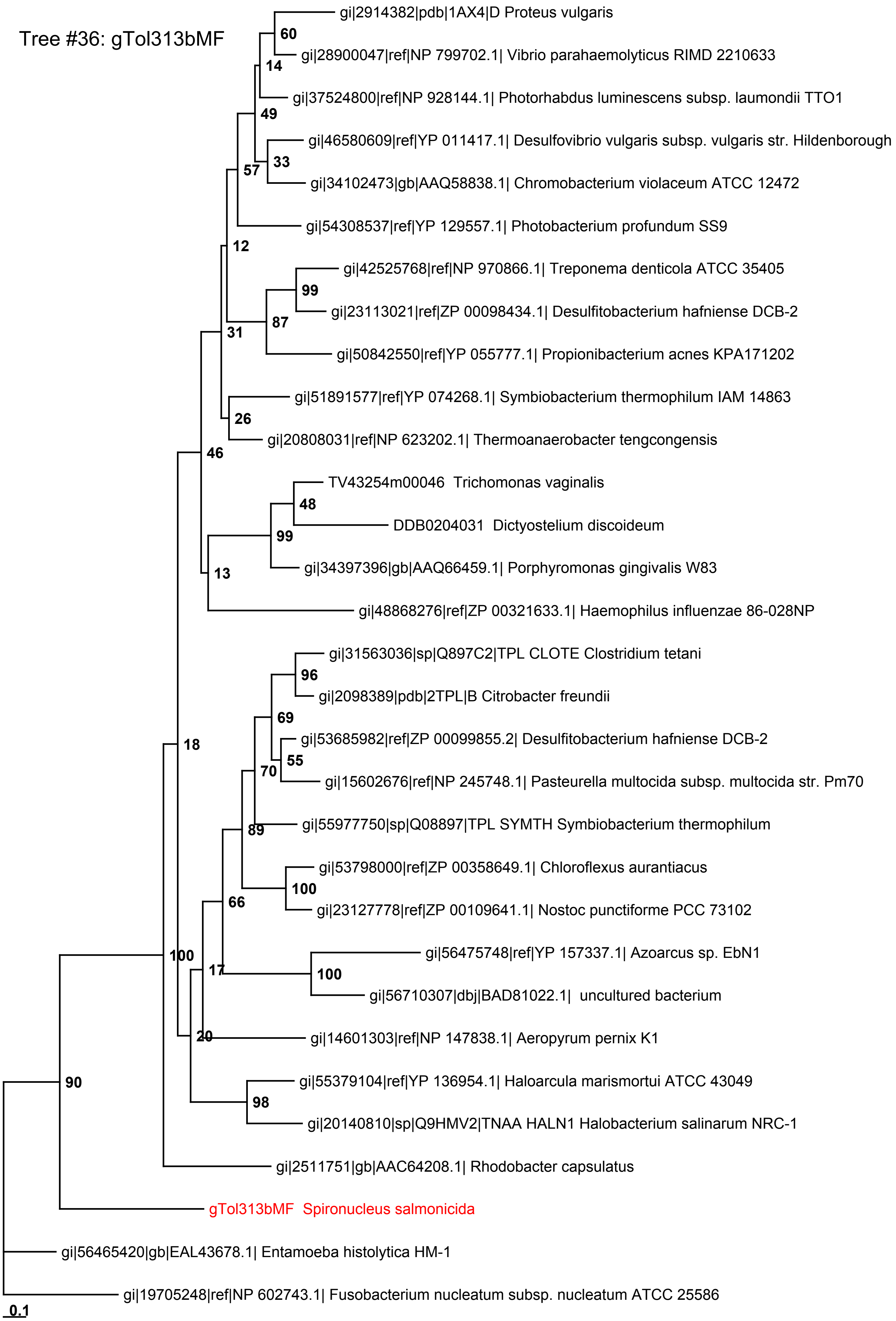
Tree #36: gTol313bMF
gi|2914382|pdb|1AX4|D Proteus vulgaris
60
gi|28900047|ref|NP 799702.1| Vibrio parahaemolyticus RIMD 2210633
14
gi|37524800|ref|NP 928144.1| Photorhabdus luminescens subsp. laumondii TTO1
49
gi|46580609|ref|YP 011417.1| Desulfovibrio vulgaris subsp. vulgaris str. Hildenborough
33
57
gi|34102473|gb|AAQ58838.1| Chromobacterium violaceum ATCC 12472
gi|54308537|ref|YP 129557.1| Photobacterium profundum SS9
12
gi|42525768|ref|NP 970866.1| Treponema denticola ATCC 35405
99
gi|23113021|ref|ZP 00098434.1| Desulfitobacterium hafniense DCB-2
87
31
gi|50842550|ref|YP 055777.1| Propionibacterium acnes KPA171202
gi|51891577|ref|YP 074268.1| Symbiobacterium thermophilum IAM 14863
26
gi|20808031|ref|NP 623202.1| Thermoanaerobacter tengcongensis
46
TV43254m00046 Trichomonas vaginalis
48
DDB0204031 Dictyostelium discoideum
99
gi|34397396|gb|AAQ66459.1| Porphyromonas gingivalis W83
13
gi|48868276|ref|ZP 00321633.1| Haemophilus influenzae 86-028NP
gi|31563036|sp|Q897C2|TPL CLOTE Clostridium tetani
96
gi|2098389|pdb|2TPL|B Citrobacter freundii
69
18
gi|53685982|ref|ZP 00099855.2| Desulfitobacterium hafniense DCB-2
55
70
gi|15602676|ref|NP 245748.1| Pasteurella multocida subsp. multocida str. Pm70
gi|55977750|sp|Q08897|TPL SYMTH Symbiobacterium thermophilum
89
gi|53798000|ref|ZP 00358649.1| Chloroflexus aurantiacus
100
66
gi|23127778|ref|ZP 00109641.1| Nostoc punctiforme PCC 73102
gi|56475748|ref|YP 157337.1| Azoarcus sp. EbN1
100
100
17
gi|56710307|dbj|BAD81022.1| uncultured bacterium
20
gi|14601303|ref|NP 147838.1| Aeropyrum pernix K1
gi|55379104|ref|YP 136954.1| Haloarcula marismortui ATCC 43049
90
98
gi|20140810|sp|Q9HMV2|TNAA HALN1 Halobacterium salinarum NRC-1
gi|2511751|gb|AAC64208.1| Rhodobacter capsulatus
gTol313bMF Spironucleus salmonicida
gi|56465420|gb|EAL43678.1| Entamoeba histolytica HM-1
gi|19705248|ref|NP 602743.1| Fusobacterium nucleatum subsp. nucleatum ATCC 25586
0.1

Tree #37: SpESTC204

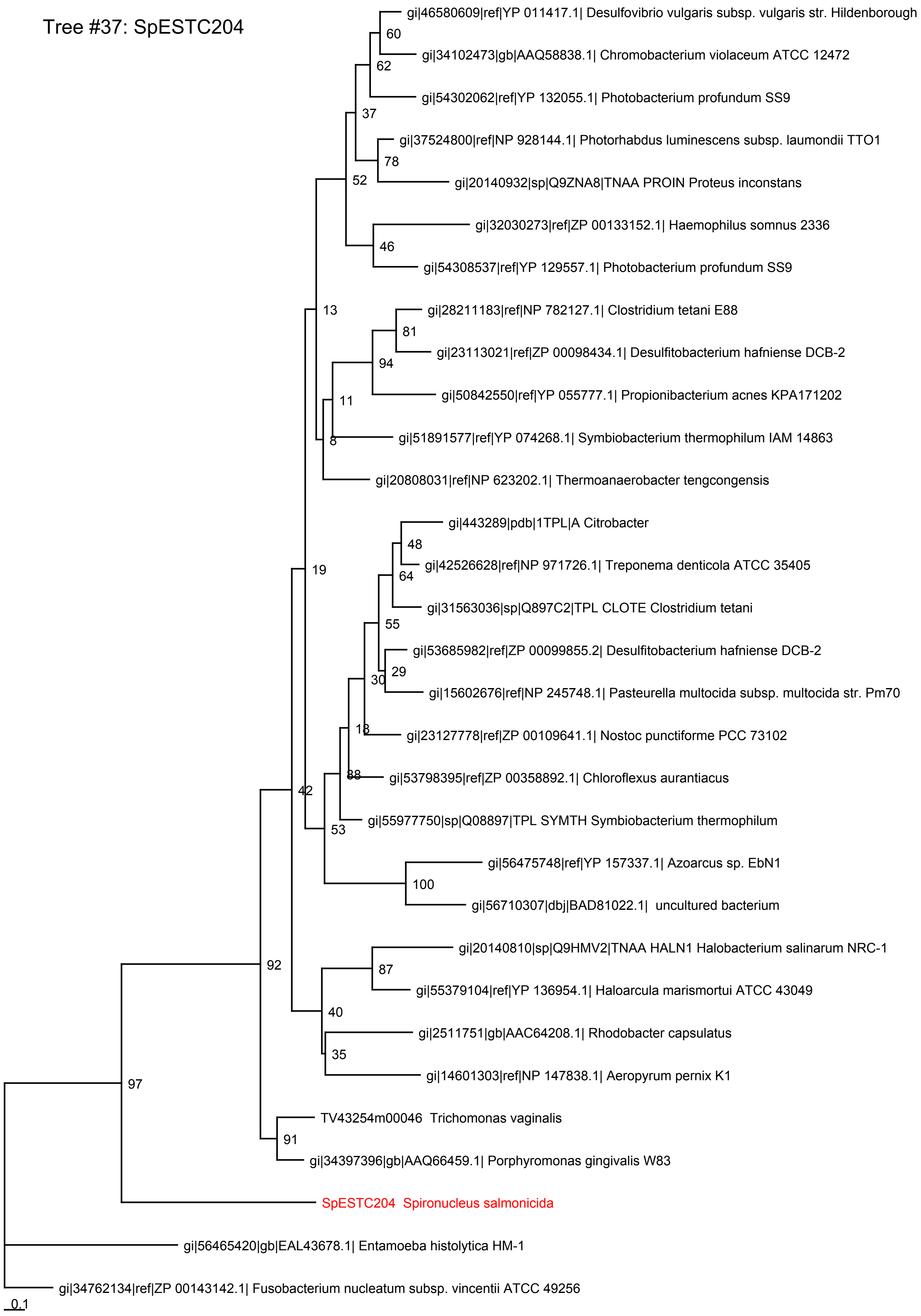

Tree #38: gZal229bMF

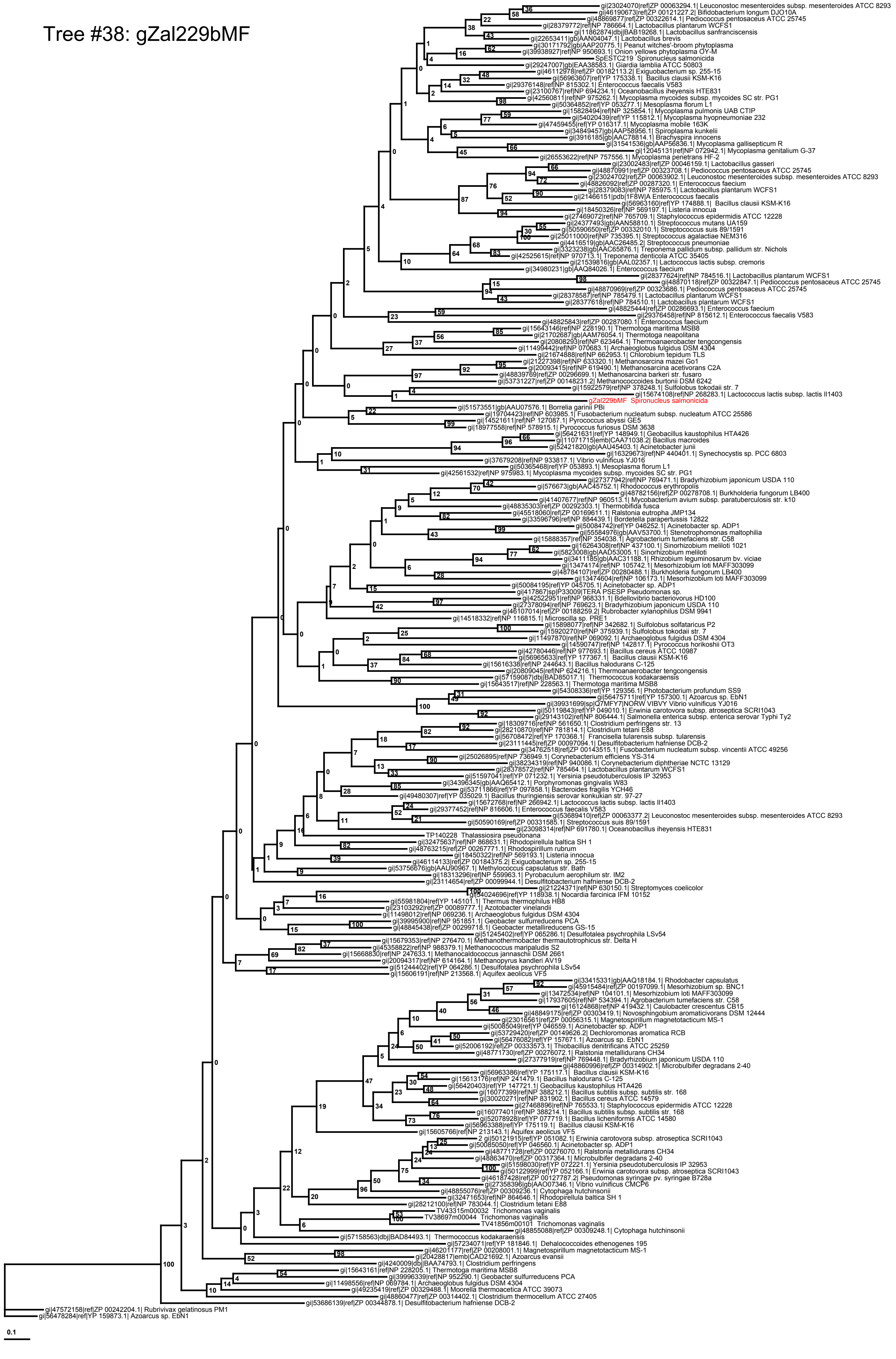

Tree #39: SpESTC219

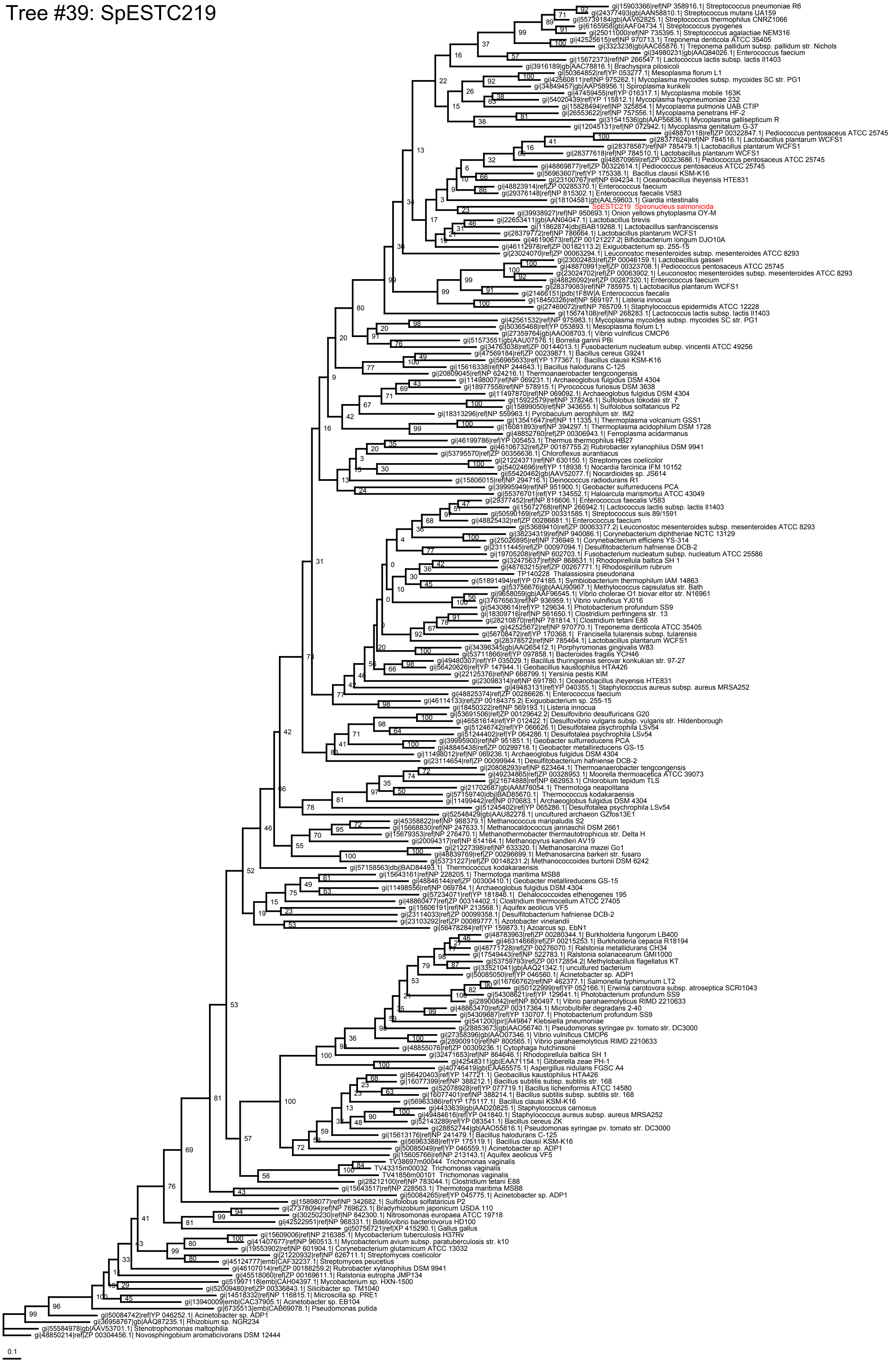

Tree #40: SpESTDH113

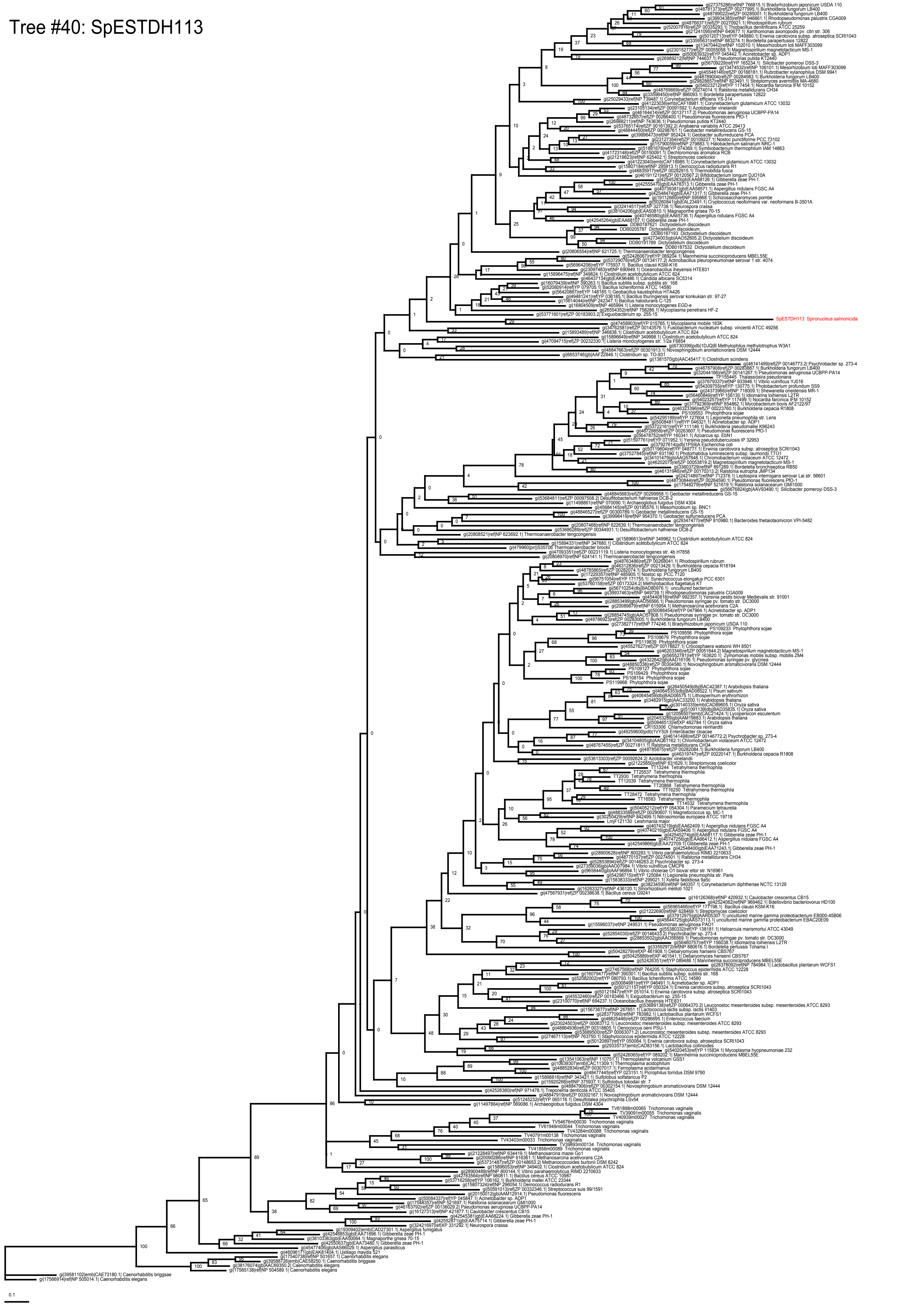

Tree #41: 11127702

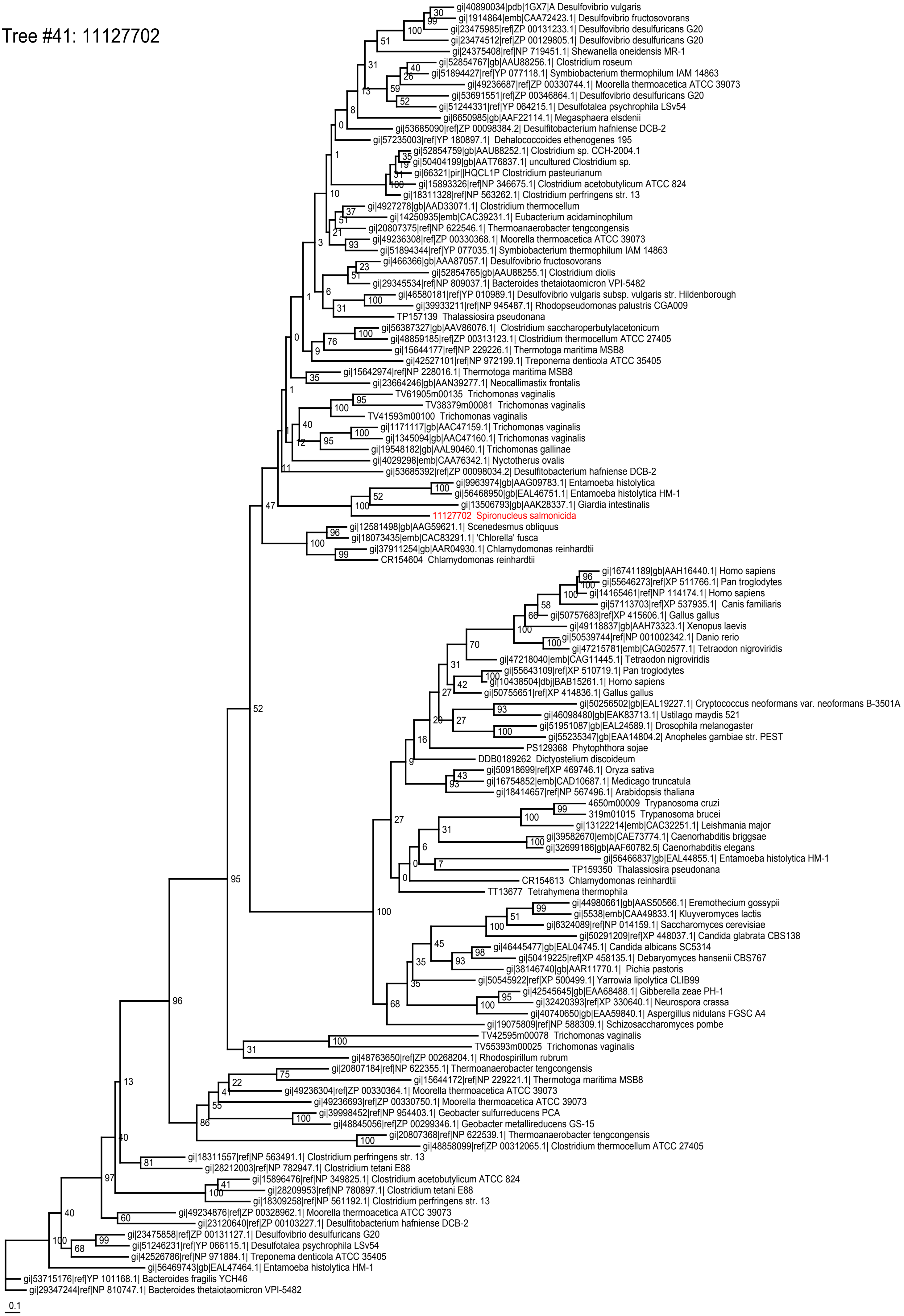

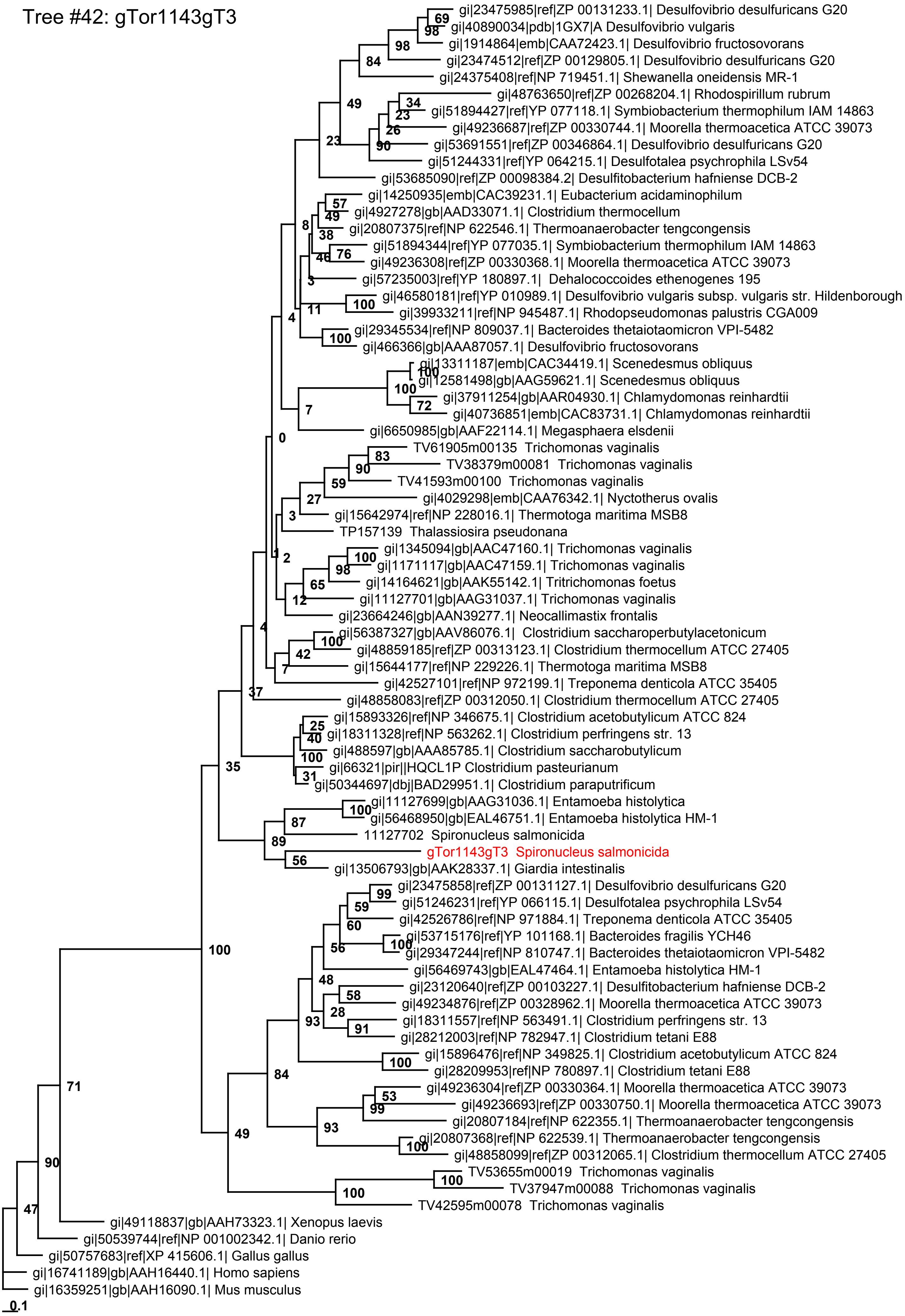
Tree #42: gTor1143gT3
gi|23475985|ref|ZP 00131233.1| Desulfovibrio desulfuricans G20
gi|40890034|pdb|1GX7|A Desulfovibrio vulgaris
gi|1914864|emb|CAA72423.1| Desulfovibrio fructosovorans
gi|23474512|ref|ZP 00129805.1| Desulfovibrio desulfuricans G20
gi|24375408|ref|NP 719451.1| Shewanella oneidensis MR-1
gi|48763650|ref|ZP 00268204.1| Rhodospirillum rubrum
gi|51894427|ref|YP 077118.1| Symbiobacterium thermophilum IAM 14863
gi|49236687|ref|ZP 00330744.1| Moorella thermoacetica ATCC 39073
gi|53691551|ref|ZP 00346864.1| Desulfovibrio desulfuricans G20
gi|51244331|ref|YP 064215.1| Desulfotalea psychrophila LSv54
gi|53685090|ref|ZP 00098384.2| Desulfitobacterium hafniense DCB-2
gi|14250935|emb|CAC39231.1| Eubacterium acidaminophilum
gi|4927278|gb|AAD33071.1| Clostridium thermocellum
gi|20807375|ref|NP 622546.1| Thermoanaerobacter tengcongensis
gi|51894344|ref|YP 077035.1| Symbiobacterium thermophilum IAM 14863
gi|49236308|ref|ZP 00330368.1| Moorella thermoacetica ATCC 39073
gi|57235003|ref|YP 180897.1| Dehalococcoides ethenogenes 195
gi|46580181|ref|YP 010989.1| Desulfovibrio vulgaris subsp. vulgaris str. Hildenborough
gi|39933211|ref|NP 945487.1| Rhodopseudomonas palustris CGA009
gi|29345534|ref|NP 809037.1| Bacteroides thetaiotaomicron VPI-5482
gi|466366|gb|AAA87057.1| Desulfovibrio fructosovorans
gi|13311187|emb|CAC34419.1| Scenedesmus obliquus
gi|12581498|gb|AAG59621.1| Scenedesmus obliquus
gi|37911254|gb|AAR04930.1| Chlamydomonas reinhardtii
gi|40736851|emb|CAC83731.1| Chlamydomonas reinhardtii
gi|6650985|gb|AAF22114.1| Megasphaera elsdenii
TV61905m00135 Trichomonas vaginalis
TV38379m00081 Trichomonas vaginalis
TV41593m00100 Trichomonas vaginalis
gi|4029298|emb|CAA76342.1| Nyctotherus ovalis
gi|15642974|ref|NP 228016.1| Thermotoga maritima MSB8
TP157139 Thalassiosira pseudonana
gi|1345094|gb|AAC47160.1| Trichomonas vaginalis
gi|1171117|gb|AAC47159.1| Trichomonas vaginalis
gi|14164621|gb|AAK55142.1| Tritrichomonas foetus
gi|11127701|gb|AAG31037.1| Trichomonas vaginalis
gi|23664246|gb|AAN39277.1| Neocallimastix frontalis
gi|56387327|gb|AAV86076.1| Clostridium saccharoperbutylacetonicum
gi|48859185|ref|ZP 00313123.1| Clostridium thermocellum ATCC 27405
gi|15644177|ref|NP 229226.1| Thermotoga maritima MSB8
gi|42527101|ref|NP 972199.1| Treponema denticola ATCC 35405
gi|48858083|ref|ZP 00312050.1| Clostridium thermocellum ATCC 27405
gi|15893326|ref|NP 346675.1| Clostridium acetobutylicum ATCC 824
gi|18311328|ref|NP 563262.1| Clostridium perfringens str. 13
gi|488597|gb|AAA85785.1| Clostridium saccharobutylicum
gi|66321|pir||HQCL1P Clostridium pasteurianum
gi|50344697|dbj|BAD29951.1| Clostridium paraputrificum
gi|11127699|gb|AAG31036.1| Entamoeba histolytica
gi|56468950|gb|EAL46751.1| Entamoeba histolytica HM-1
11127702 Spironucleus salmonicida
gTor1143gT3 Spironucleus salmonicida
gi|13506793|gb|AAK28337.1| Giardia intestinalis
gi|23475858|ref|ZP 00131127.1| Desulfovibrio desulfuricans G20
gi|51246231|ref|YP 066115.1| Desulfotalea psychrophila LSv54
gi|42526786|ref|NP 971884.1| Treponema denticola ATCC 35405
gi|53715176|ref|YP 101168.1| Bacteroides fragilis YCH46
gi|29347244|ref|NP 810747.1| Bacteroides thetaiotaomicron VPI-5482
gi|56469743|gb|EAL47464.1| Entamoeba histolytica HM-1
gi|23120640|ref|ZP 00103227.1| Desulfitobacterium hafniense DCB-2
gi|49234876|ref|ZP 00328962.1| Moorella thermoacetica ATCC 39073
gi|18311557|ref|NP 563491.1| Clostridium perfringens str. 13
gi|28212003|ref|NP 782947.1| Clostridium tetani E88
gi|15896476|ref|NP 349825.1| Clostridium acetobutylicum ATCC 824
gi|28209953|ref|NP 780897.1| Clostridium tetani E88
gi|49236304|ref|ZP 00330364.1| Moorella thermoacetica ATCC 39073
gi|49236693|ref|ZP 00330750.1| Moorella thermoacetica ATCC 39073
gi|20807184|ref|NP 622355.1| Thermoanaerobacter tengcongensis
gi|20807368|ref|NP 622539.1| Thermoanaerobacter tengcongensis
gi|48858099|ref|ZP 00312065.1| Clostridium thermocellum ATCC 27405
TV53655m00019 Trichomonas vaginalis
TV37947m00088 Trichomonas vaginalis
TV42595m00078 Trichomonas vaginalis
gi|49118837|gb|AAH73323.1| Xenopus laevis
gi|50539744|ref|NP 001002342.1| Danio rerio
gi|50757683|ref|XP 415606.1| Gallus gallus
gi|16741189|gb|AAH16440.1| Homo sapiens
gi|16359251|gb|AAH16090.1| Mus musculus
0.1

Tree #43: SpESTC115

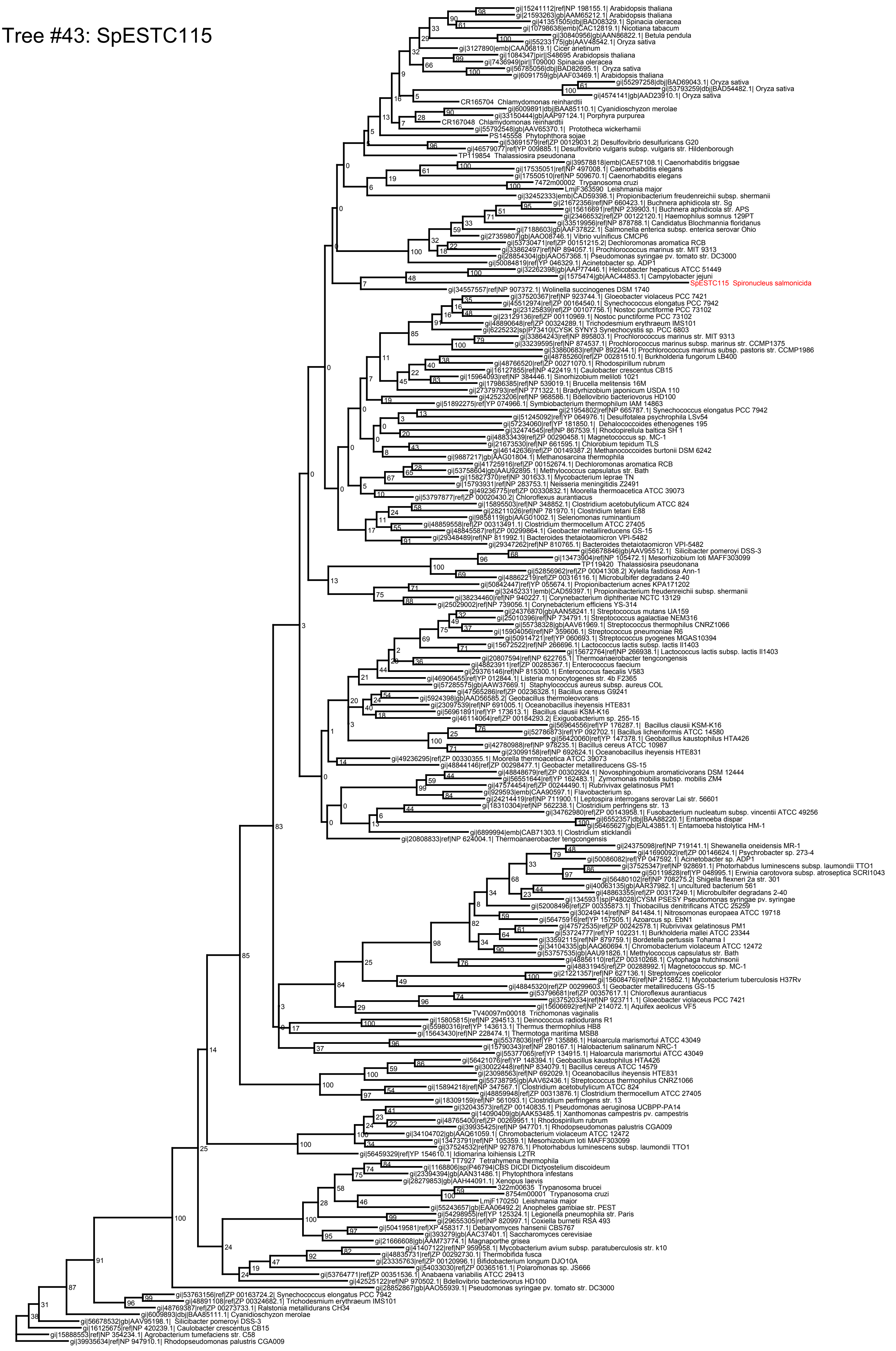

Tree #44: SpESTZap1933

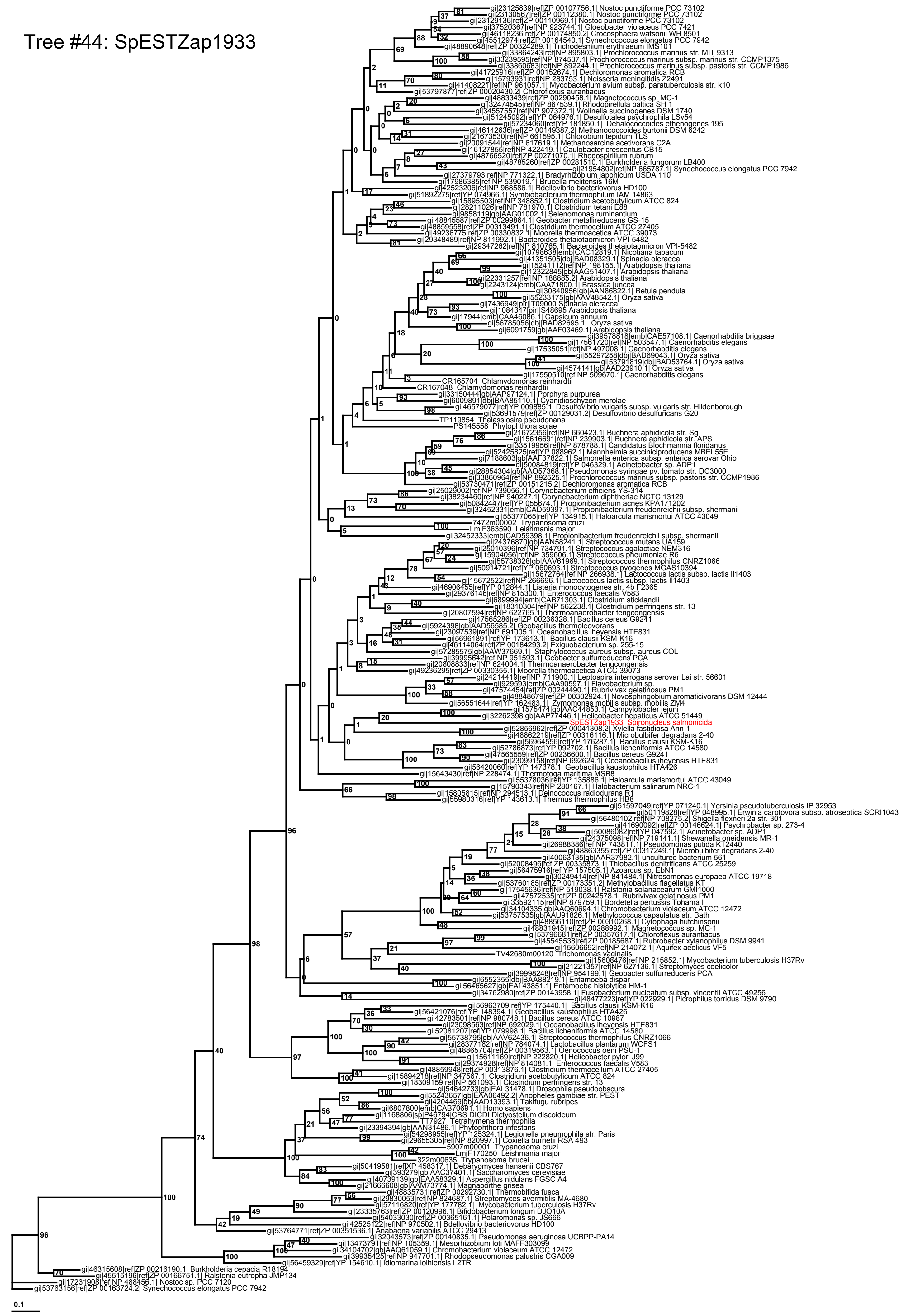

Tree #45: gZap471gT3

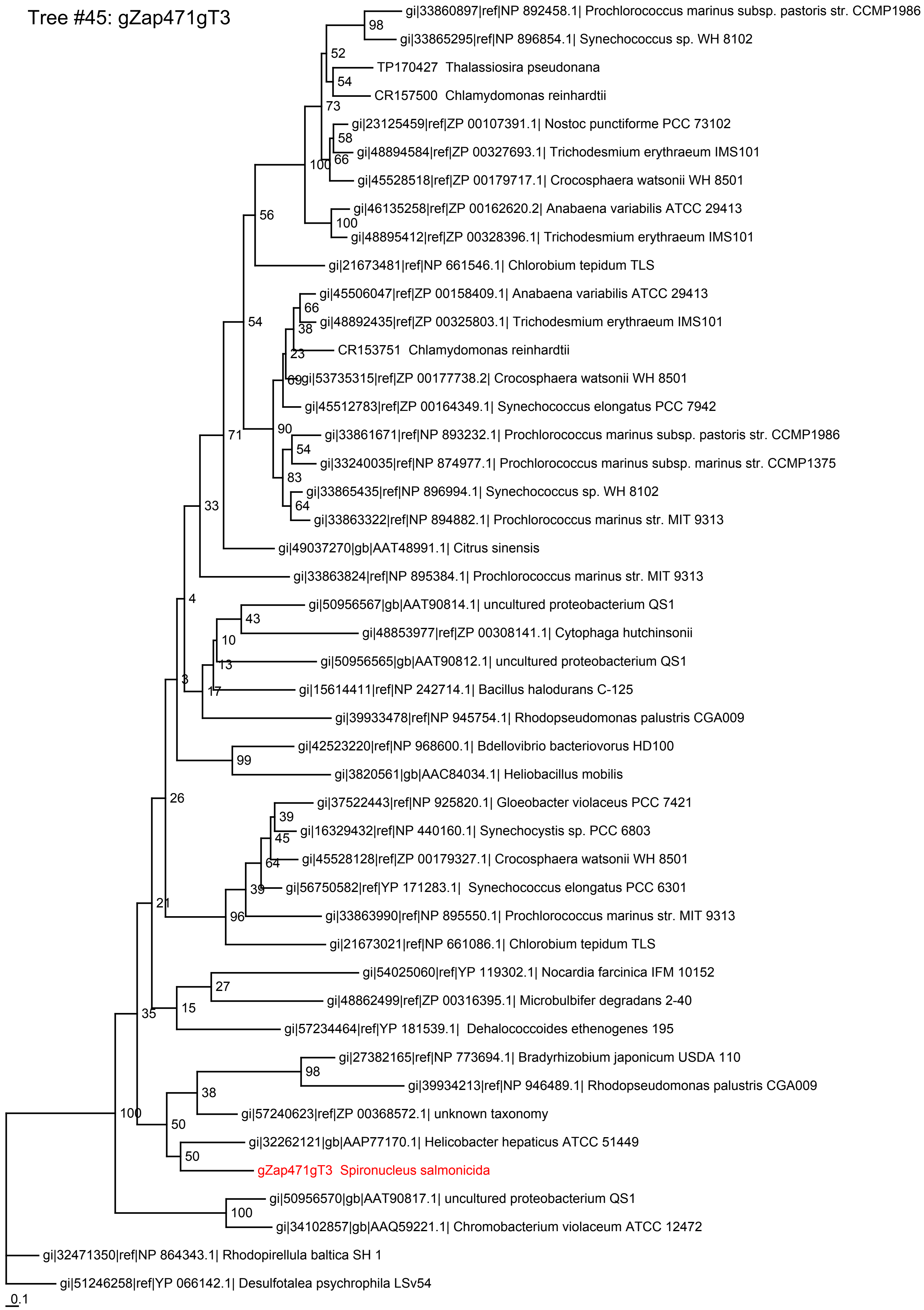

Tree #46: SpESTZap110

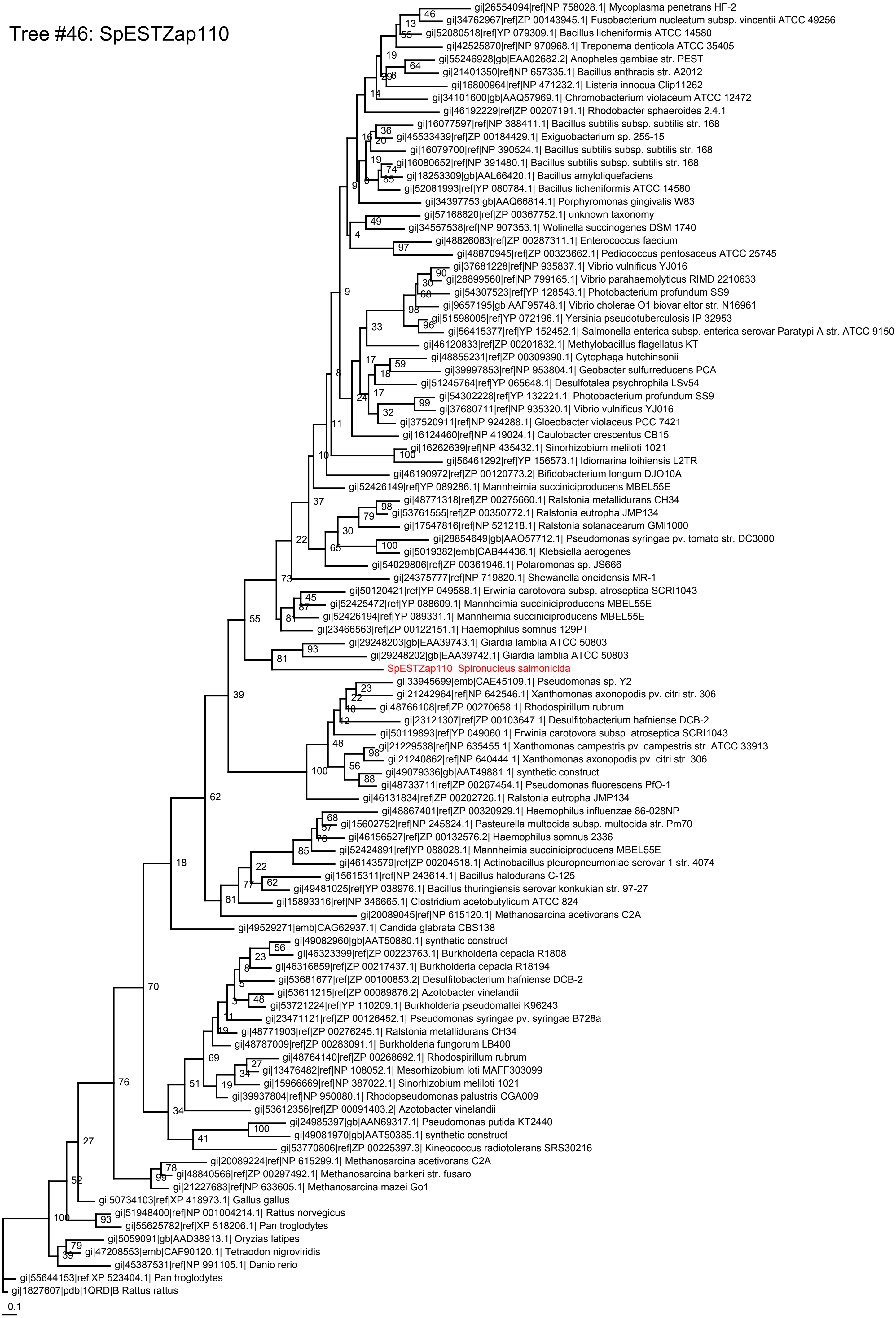

Tree #47: 27983595

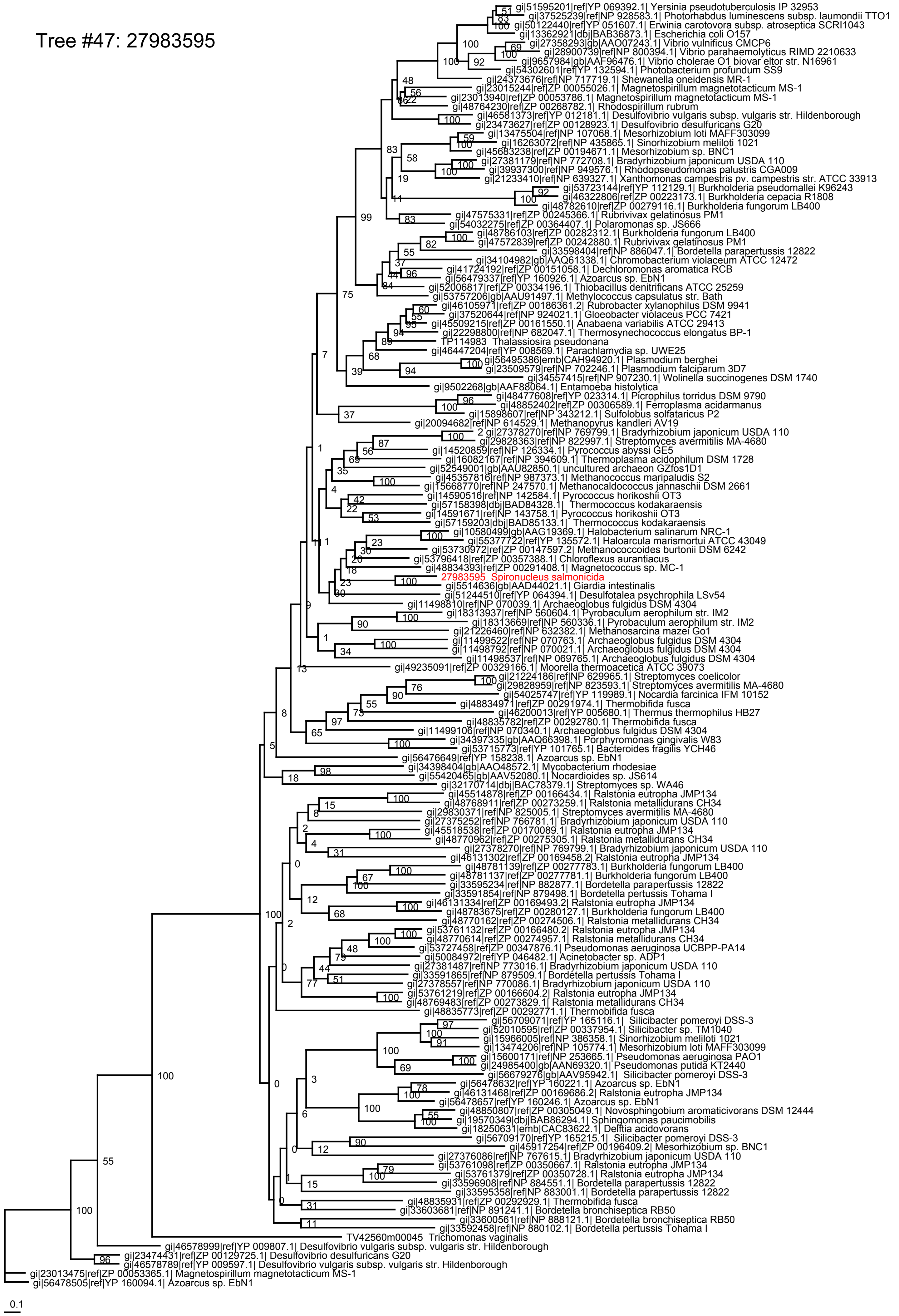

Tree #48: SpESTZap2148

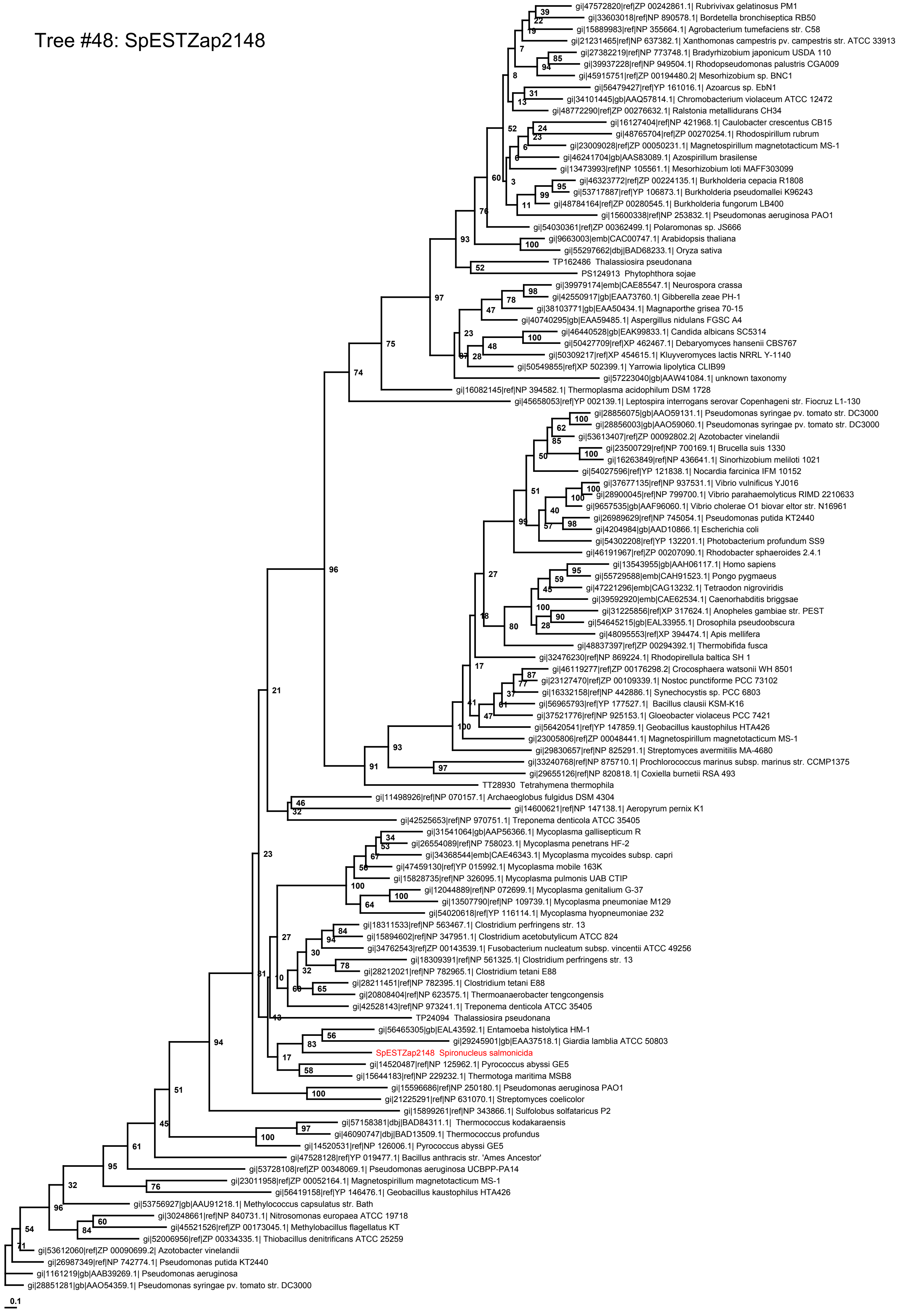

Tree #49: gTor927gT3

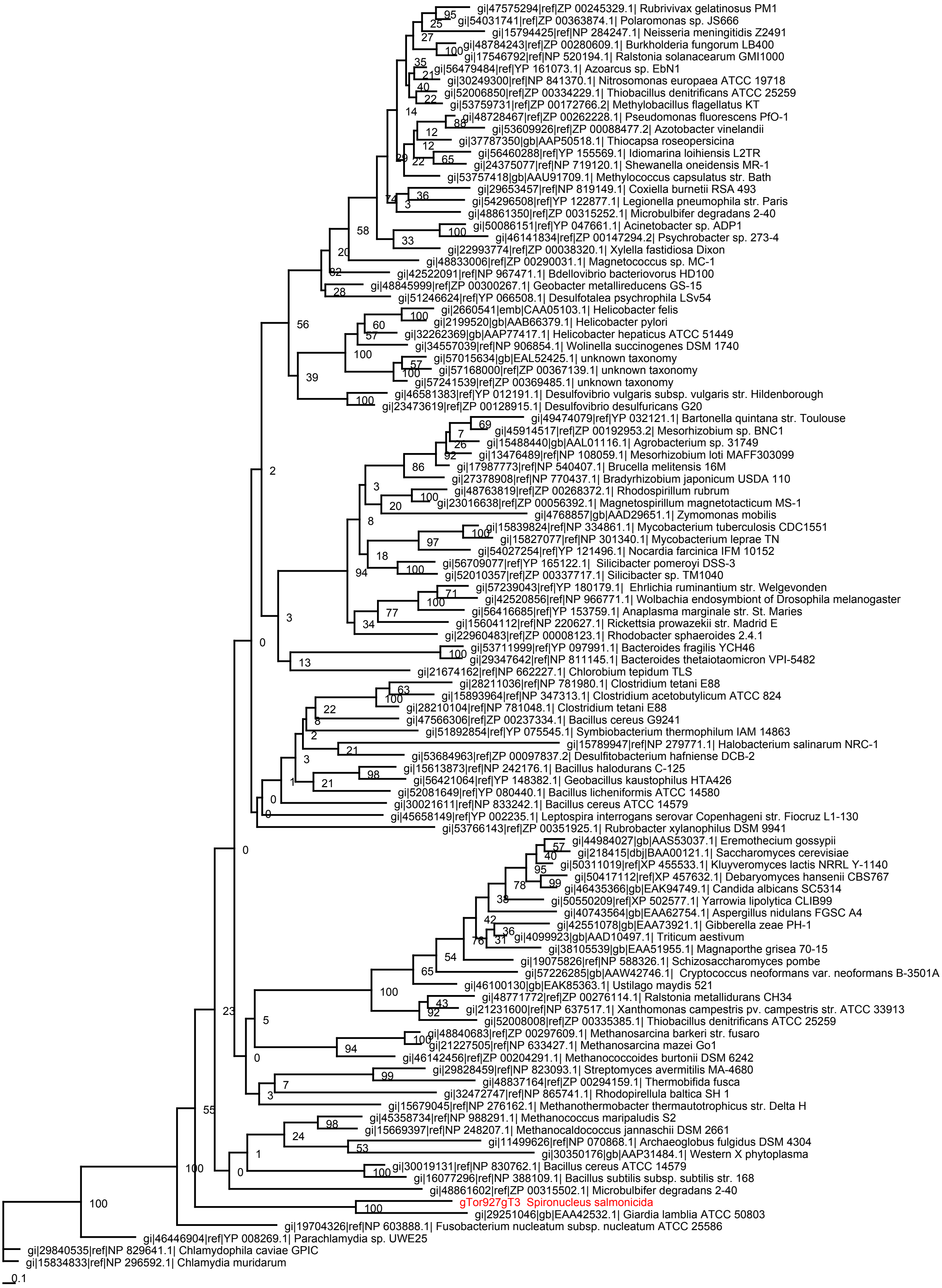

Tree #50: gZar919bT7

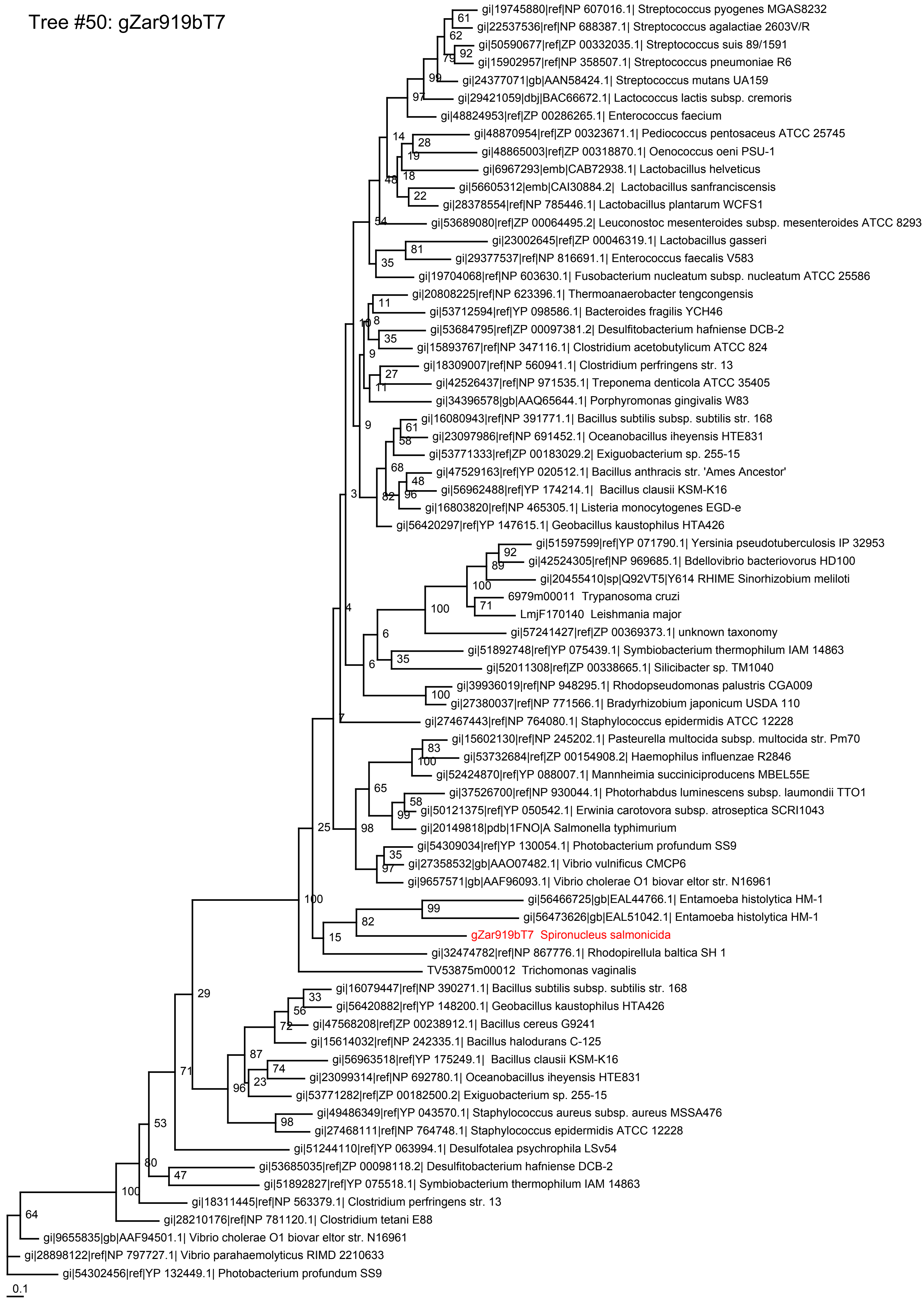

Supplement: Additional file 5 — Phylogenetic trees 26–50 for genes putatively involved in LGT events and listed in Additional file 3. [file 1471-2164-8-51-S5.pdf]
