# Supplementary material for: A genomic survey of the fish parasite Spironucleus salmonicida indicates genomic plasticity among diplomonads and significant lateral gene transfer in eukaryote genome evolution
Source: BMC Genomics. 2007 Feb 14;8:51. doi: 10.1186/1471-2164-8-51 (PMC1805757; doi:10.1186/1471-2164-8-51)

**Additional file 6 - Andersson *et al.***

Phylogenetic trees 51-72 for genes putatively involved in LGT events and listed in Additional file 3.

Tree #51: SpESTC154

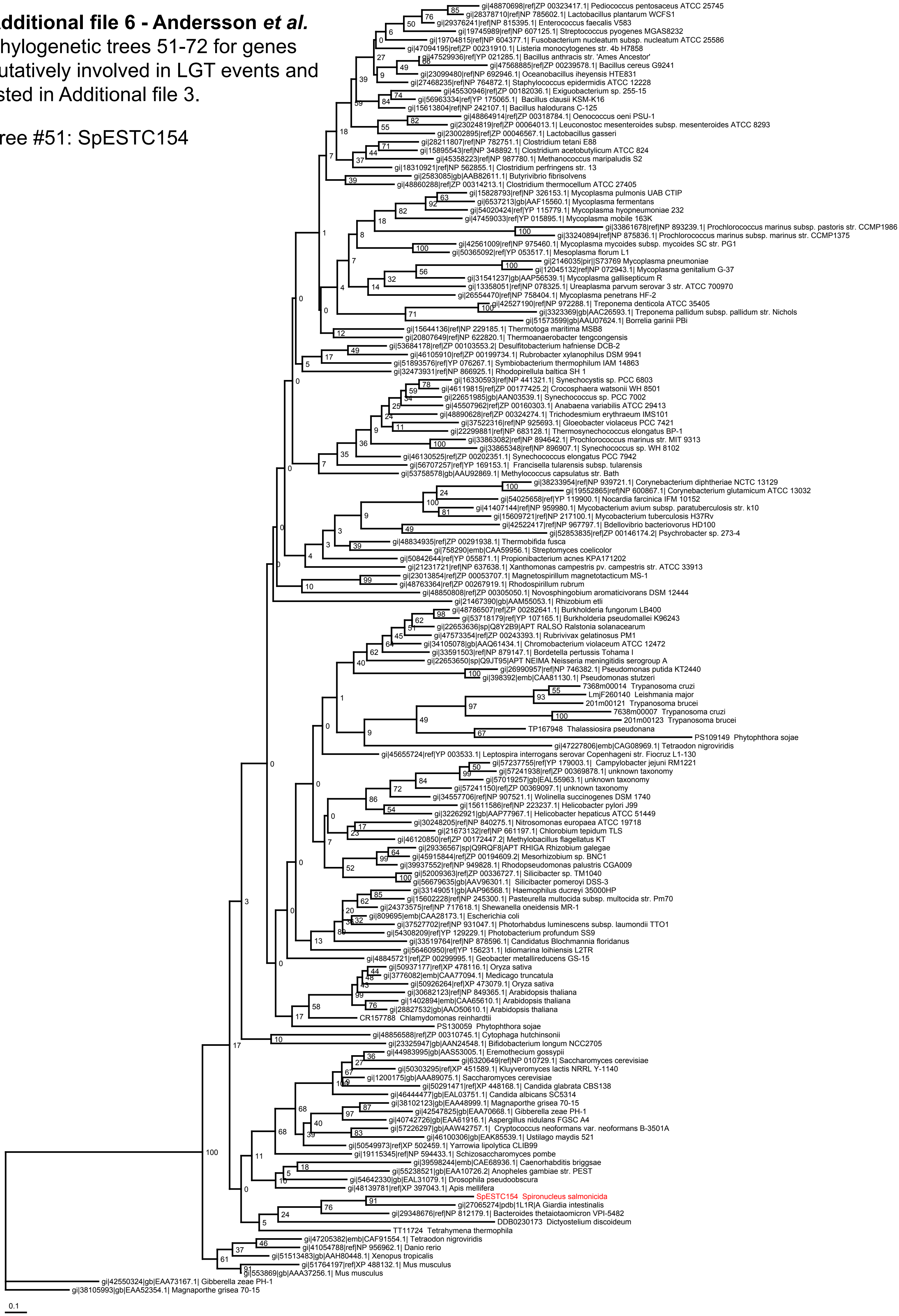

Tree #52: SpESTC251, SpESTC254

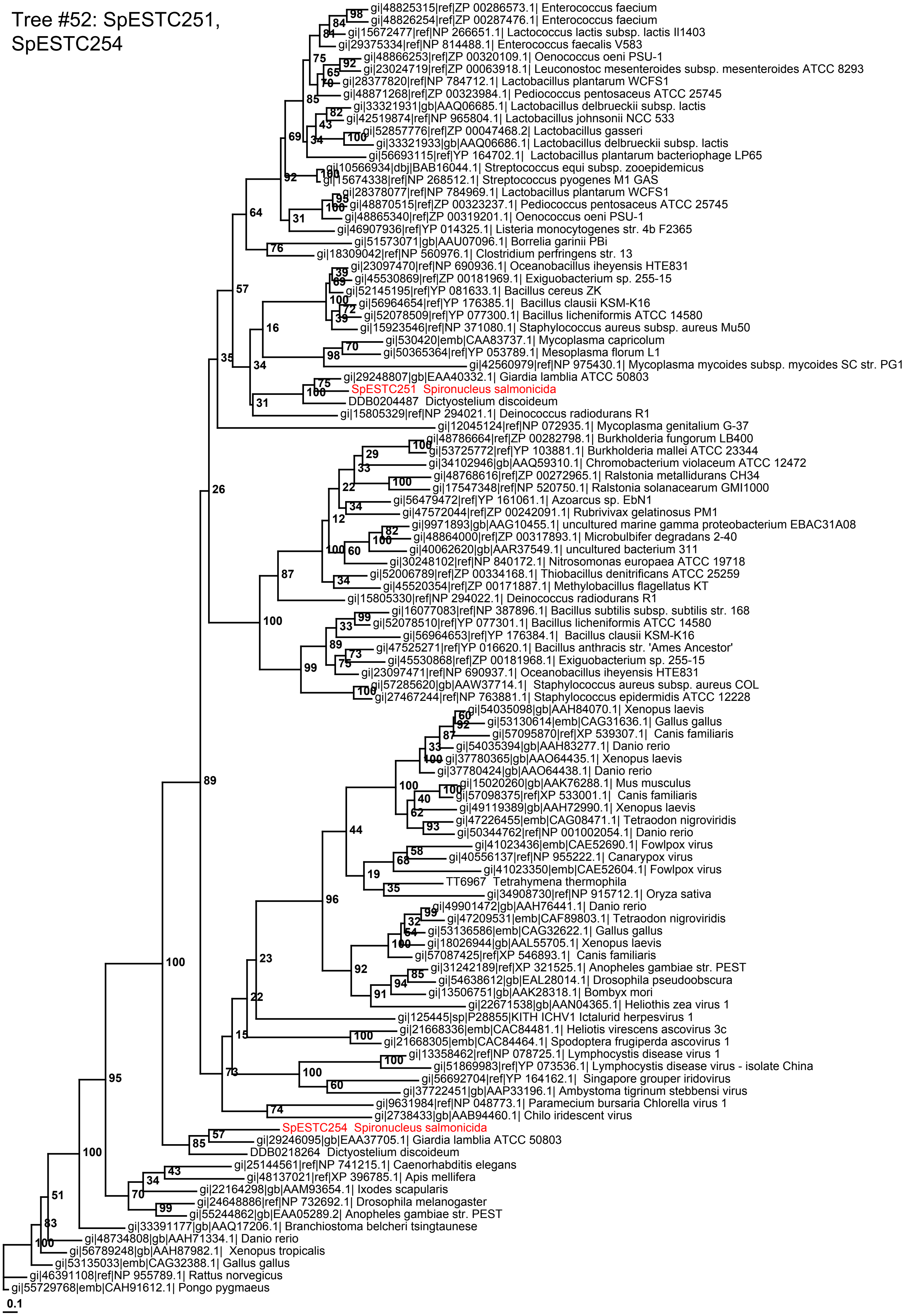

# Tree #53: gTor125bT7

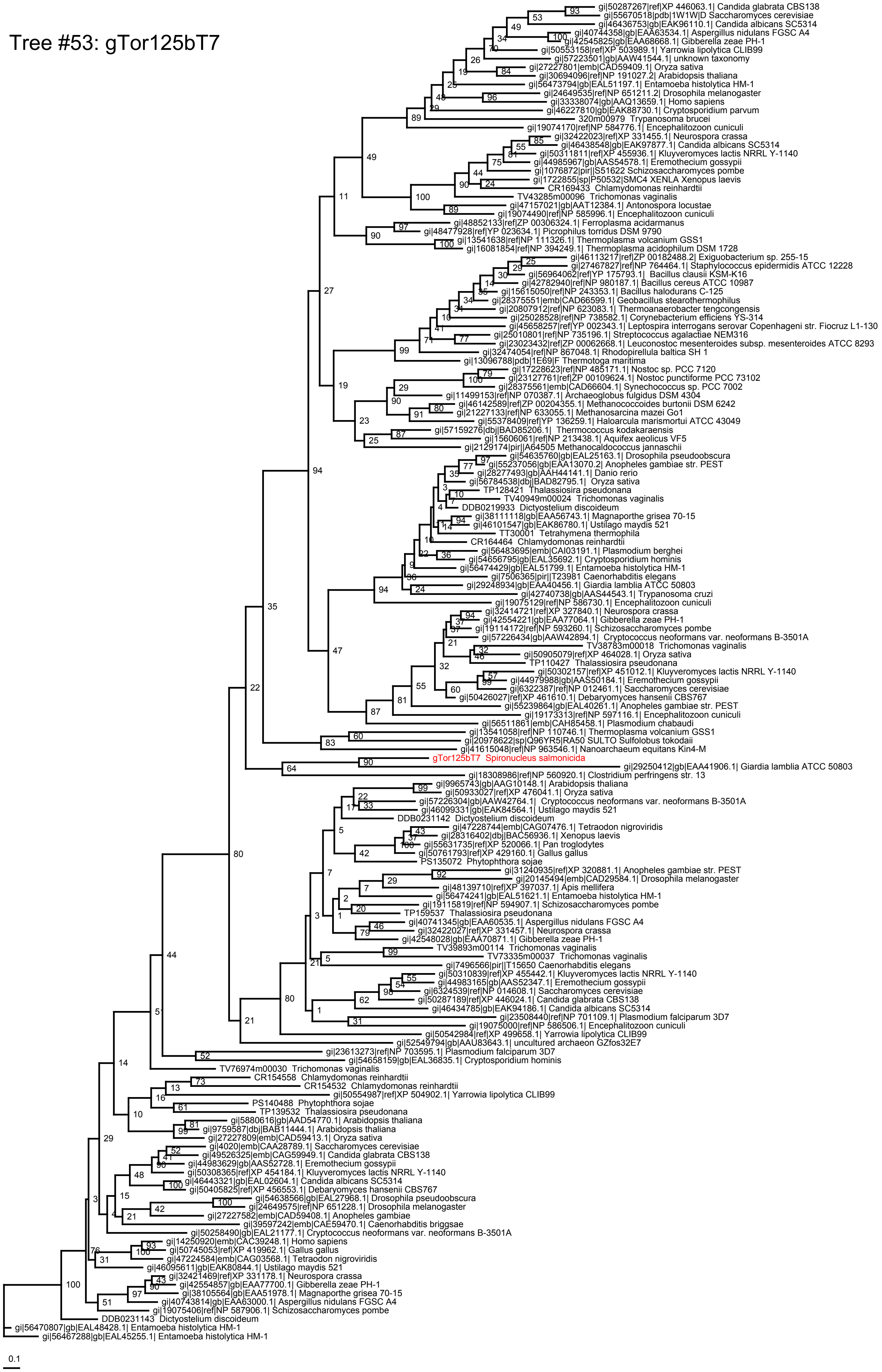

Tree #54: 27982526

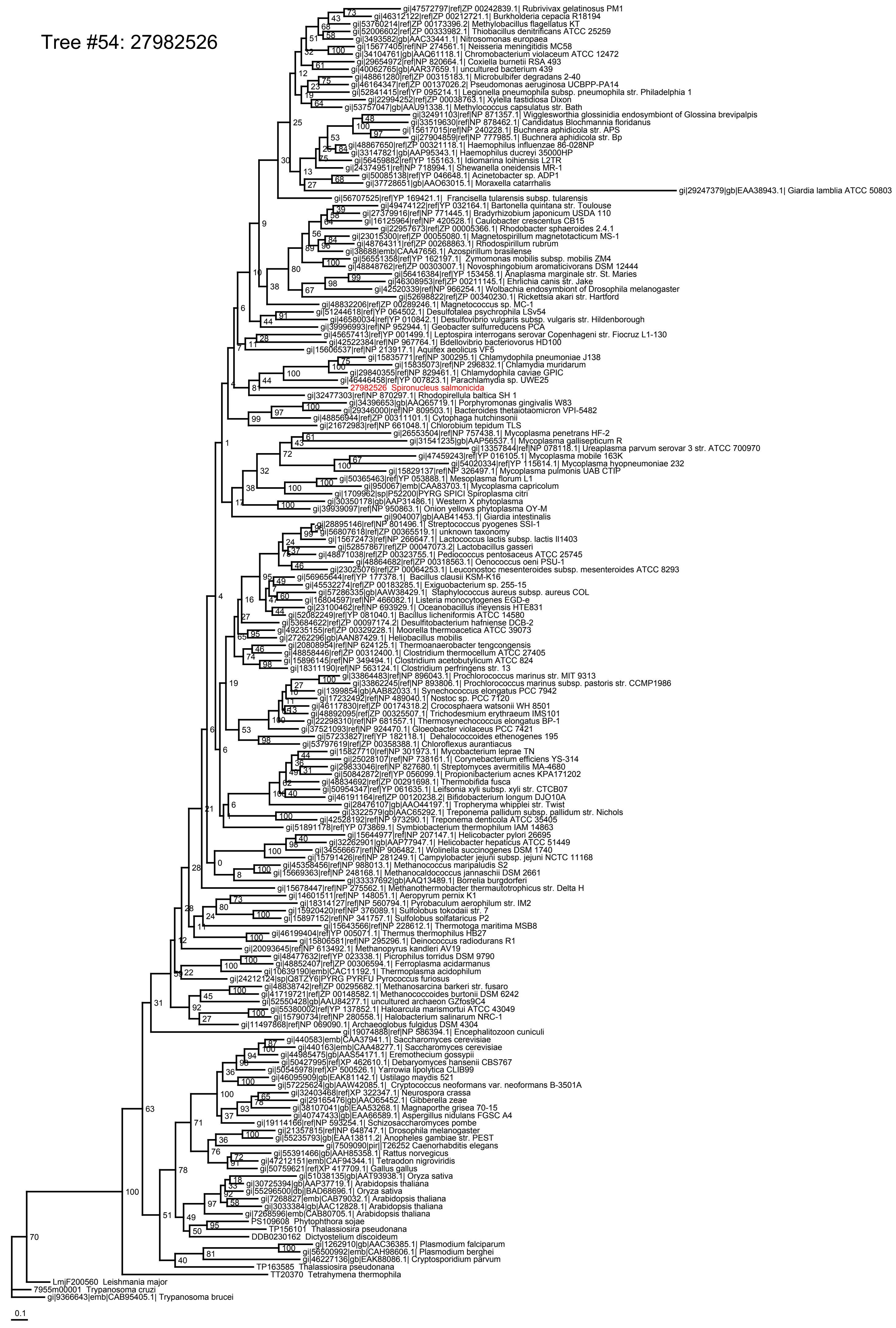

Tree #55: 27984028

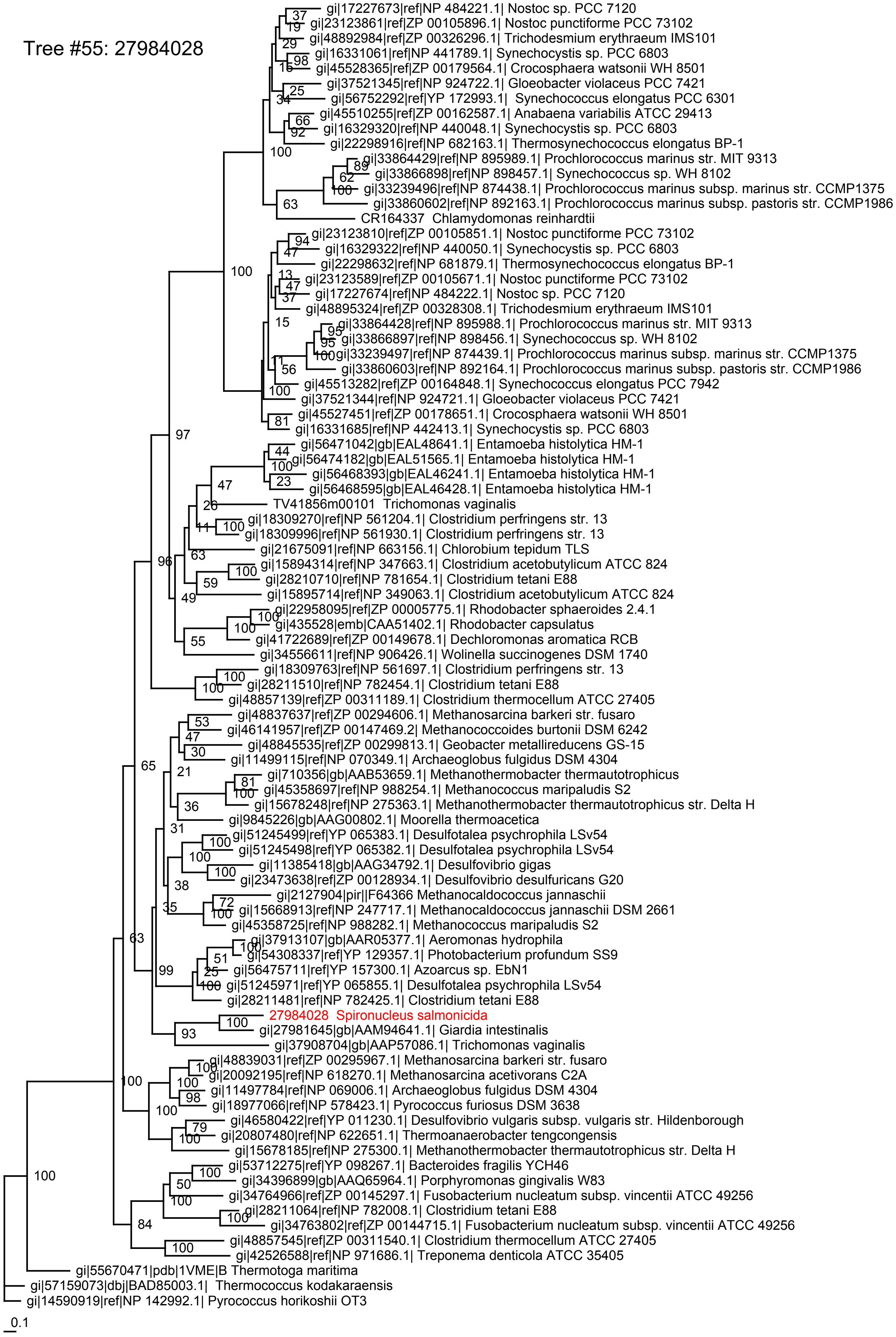

# Tree #56: SpESTZap2025

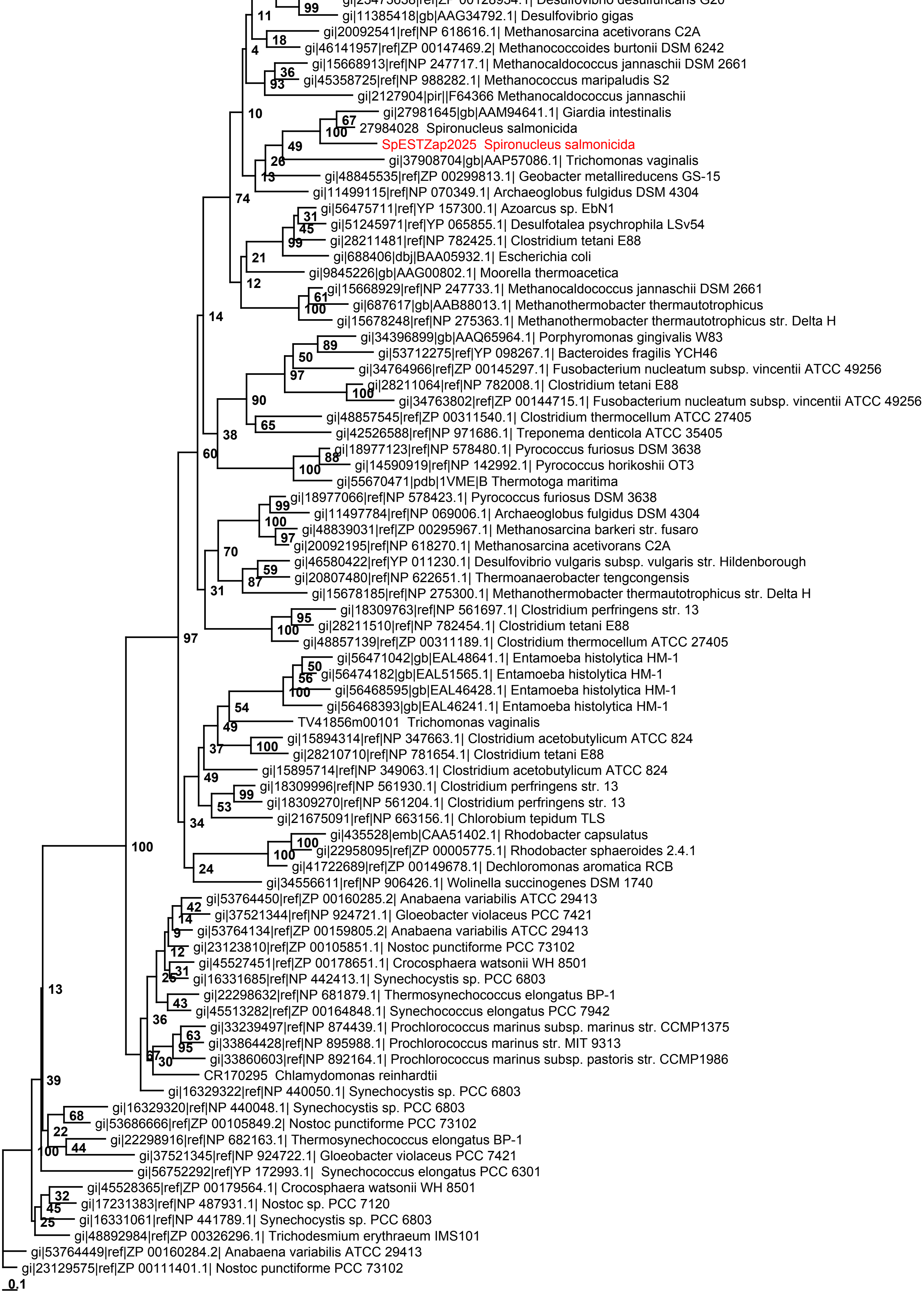

Tree #57: SpESTC196, gZap998gT3

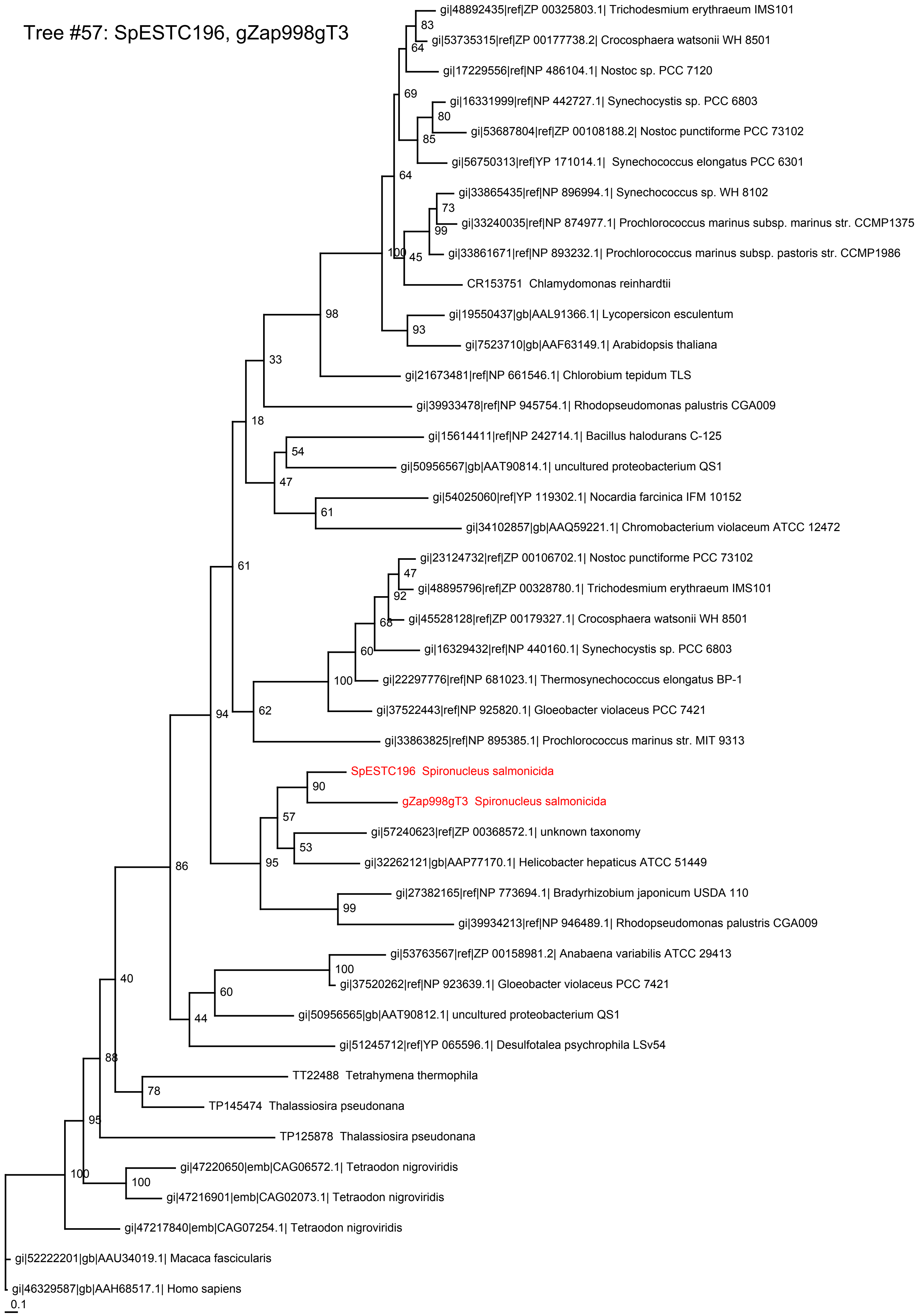

Tree #58: SpESTC267, SpESTC282

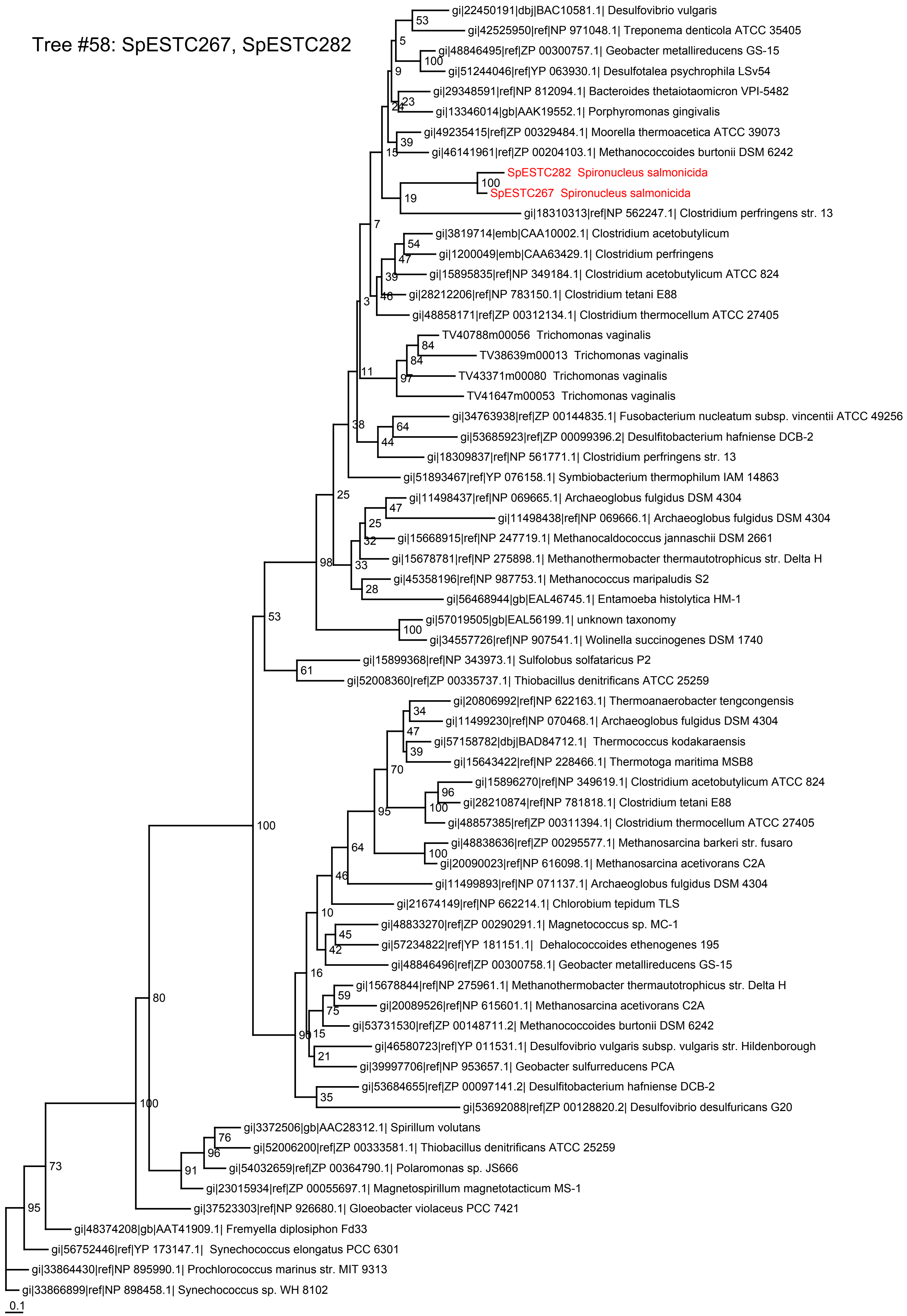

# Tree #59: gTor1213bT7

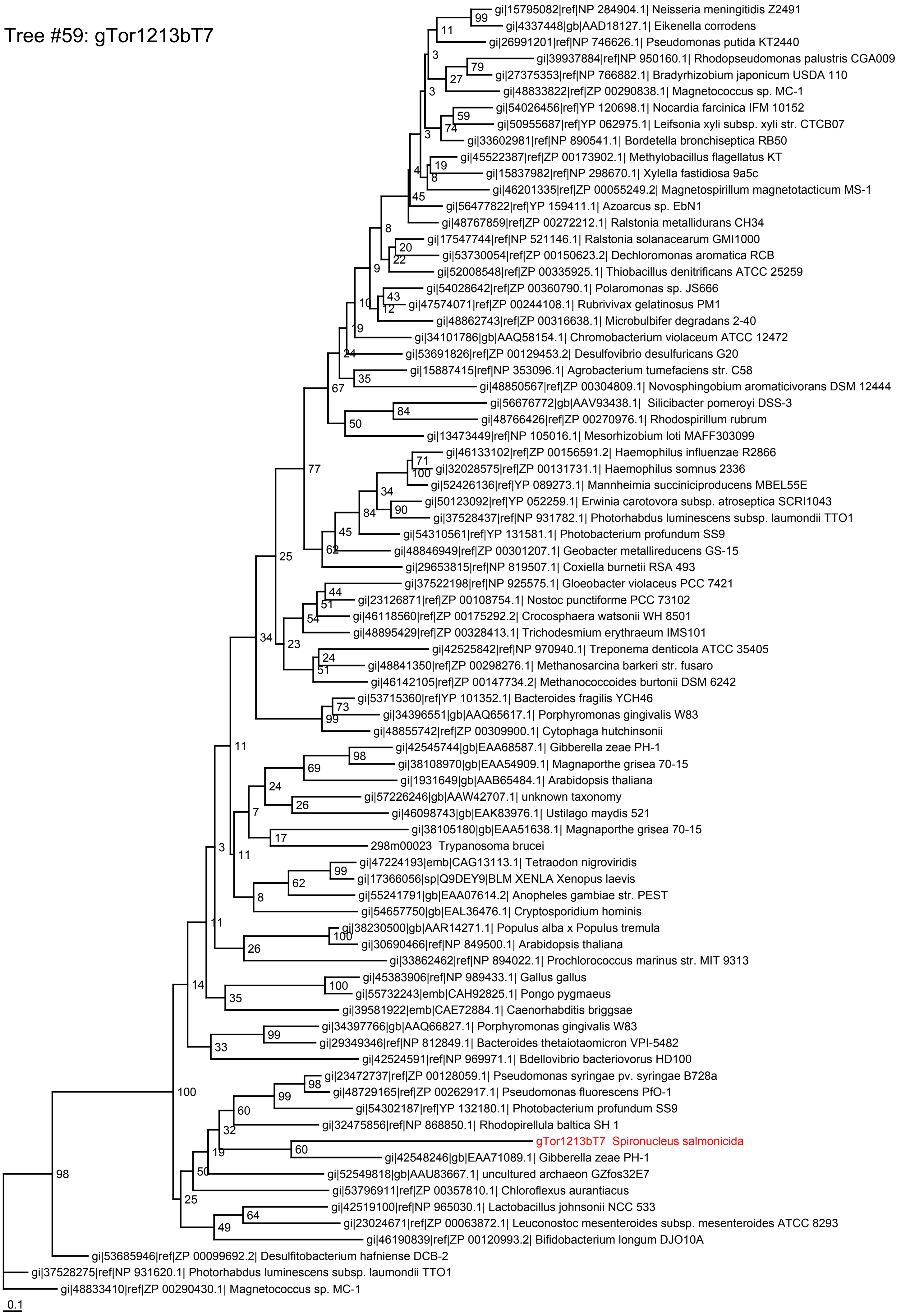

# Tree #60: gTor541gT3

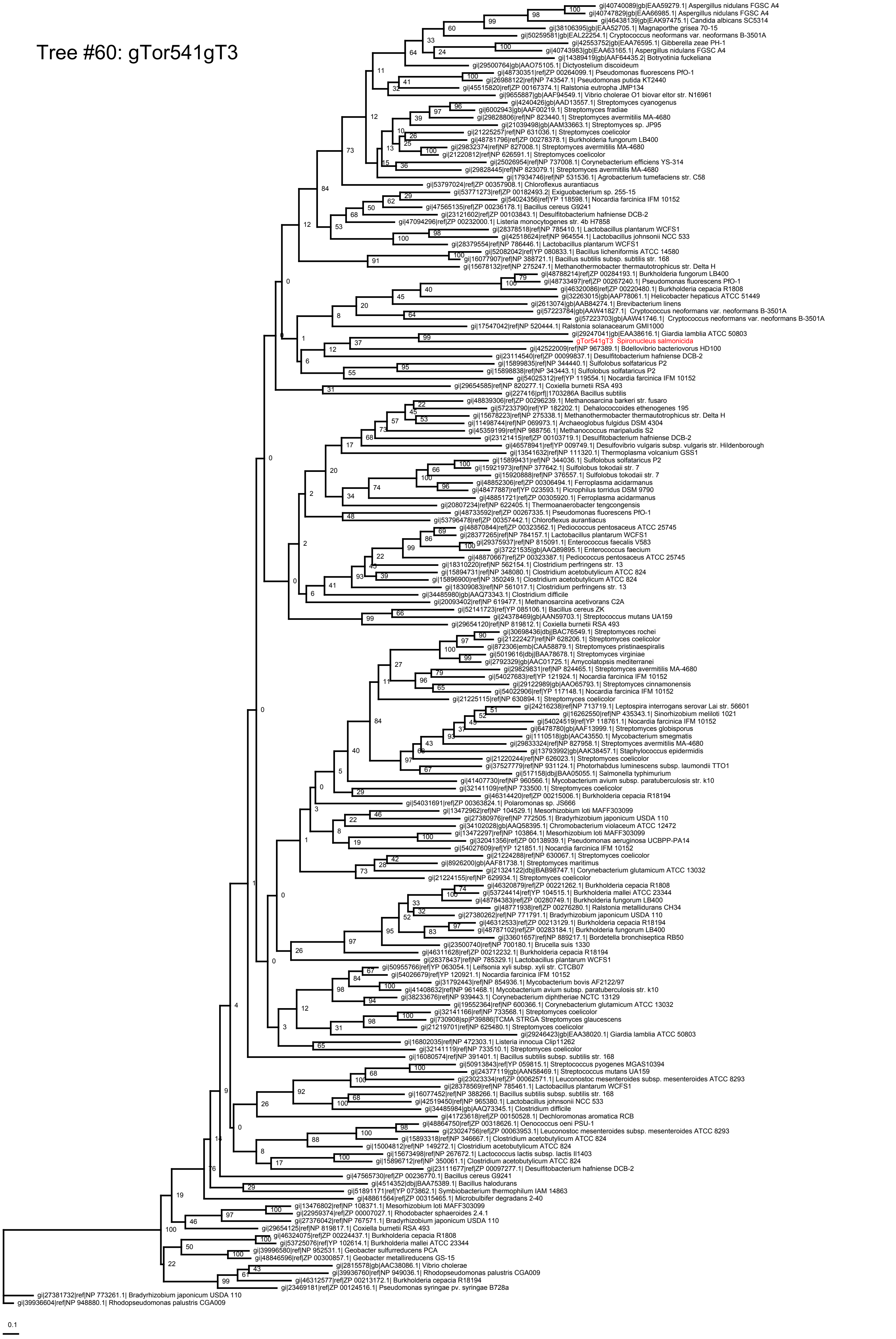

Tree #61: gTor565bT7

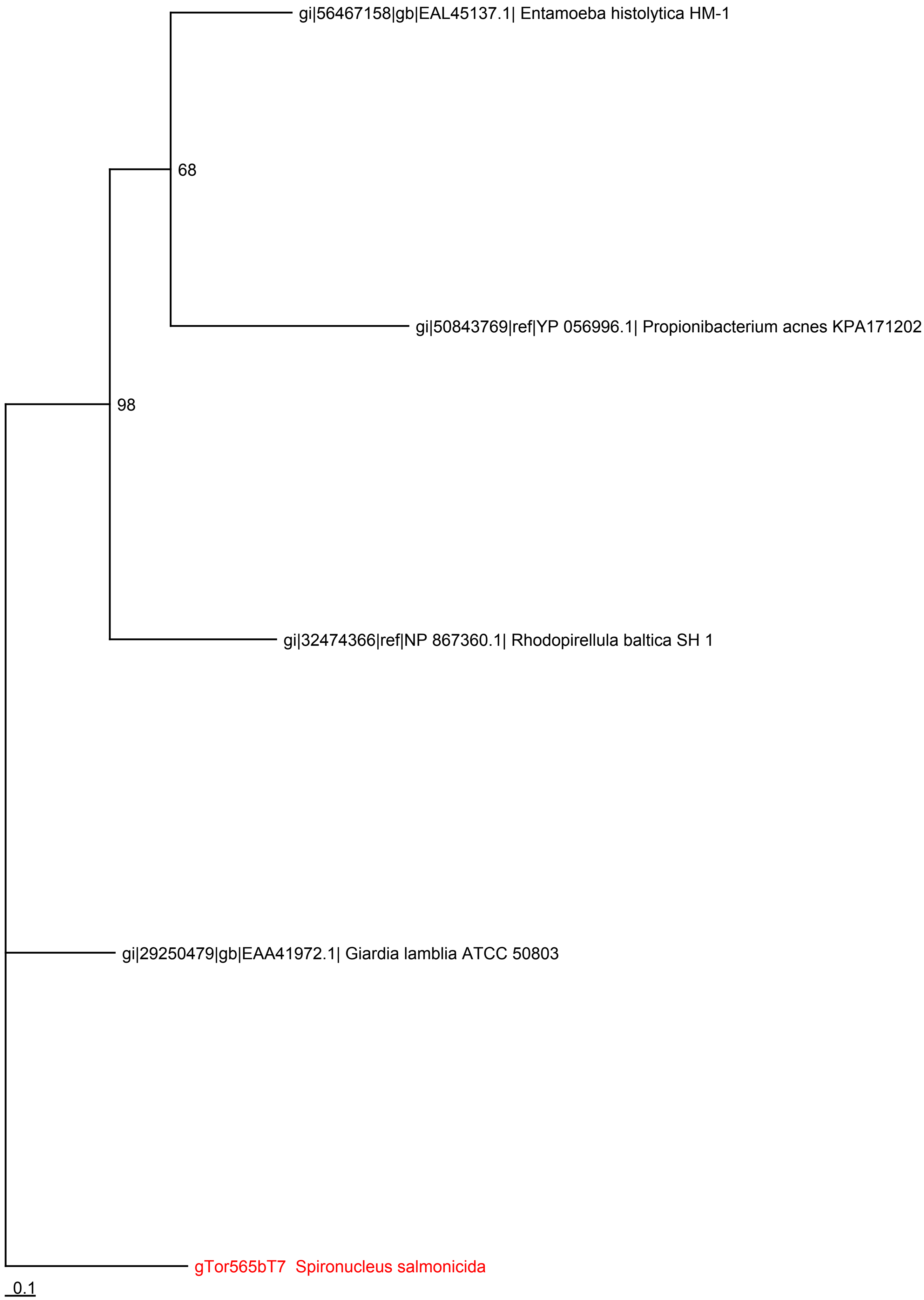

Tree #62: gTor916gT3

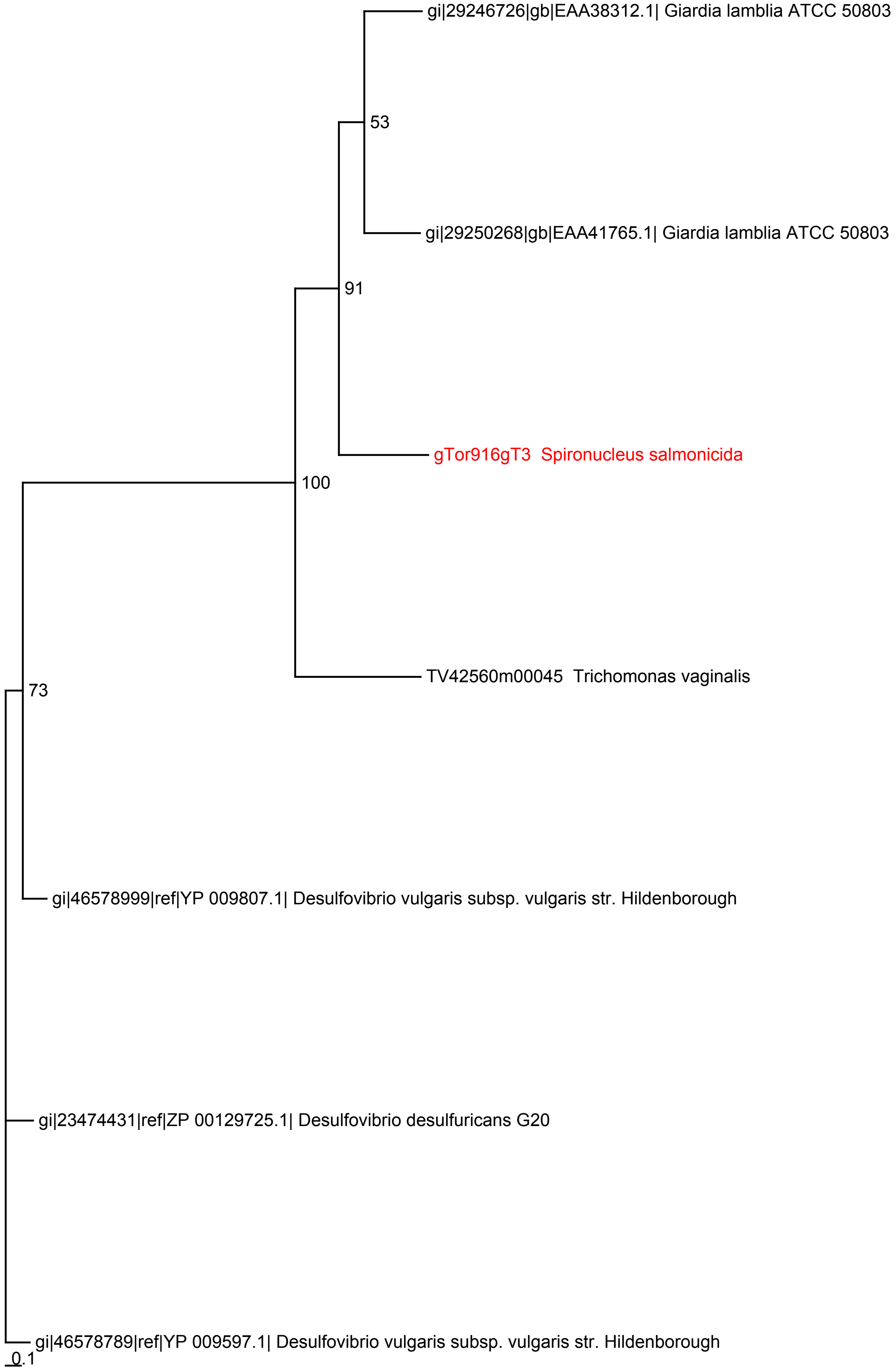

Tree #63: gTor930bT7

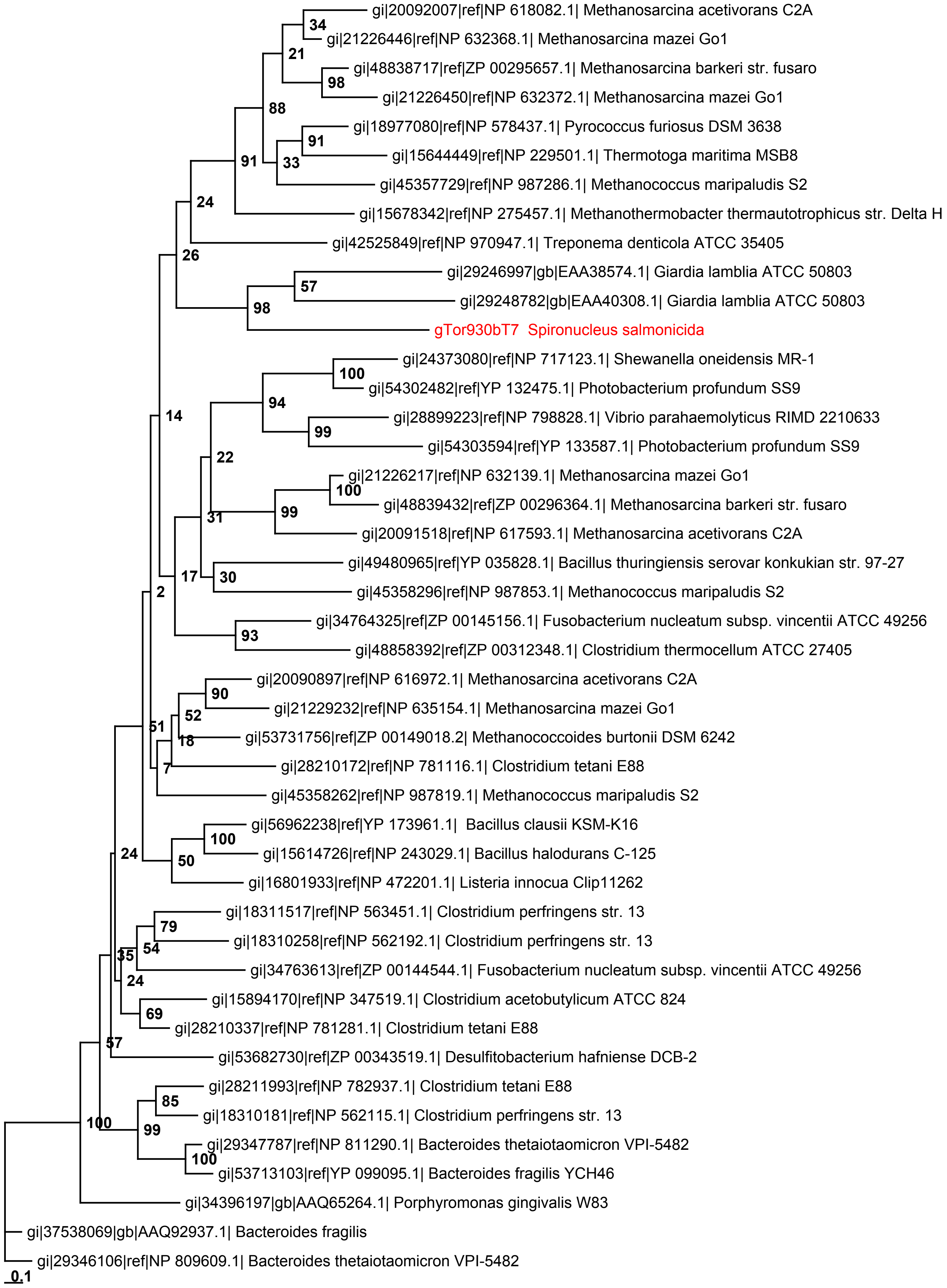

Tree #64: gZap260bT7

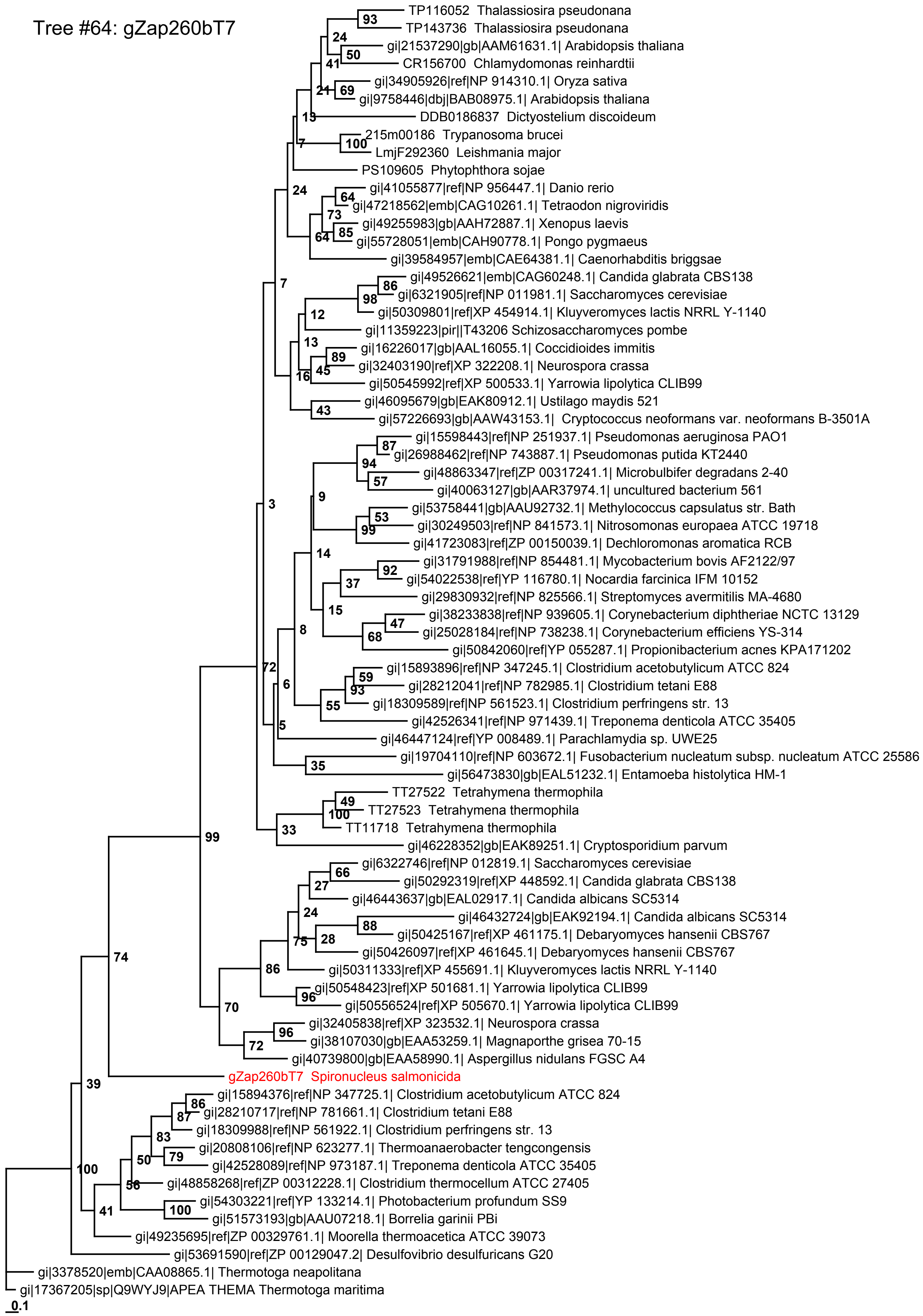

# Tree #65: gZap950bT7

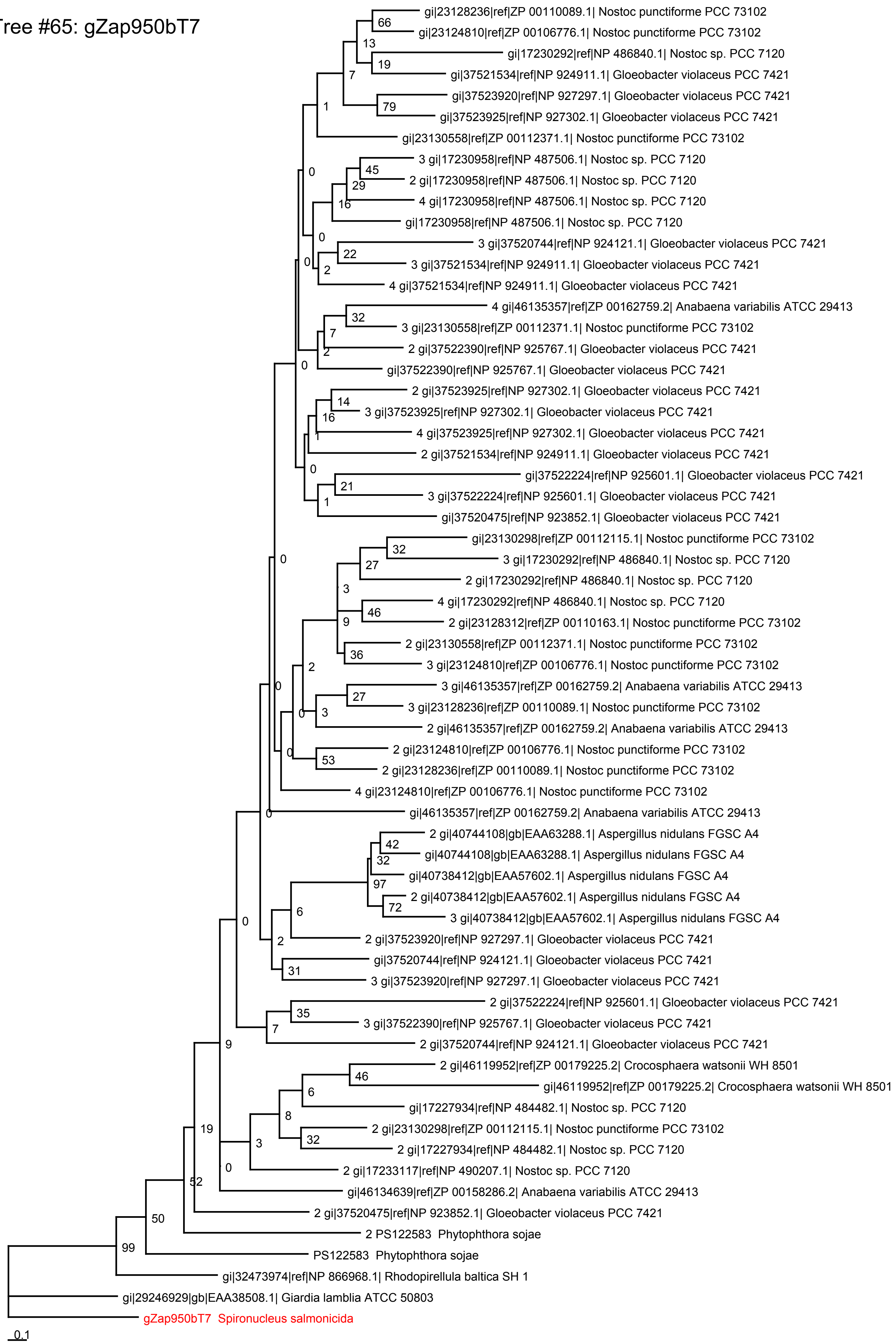

# Tree #66: gZar587bT72

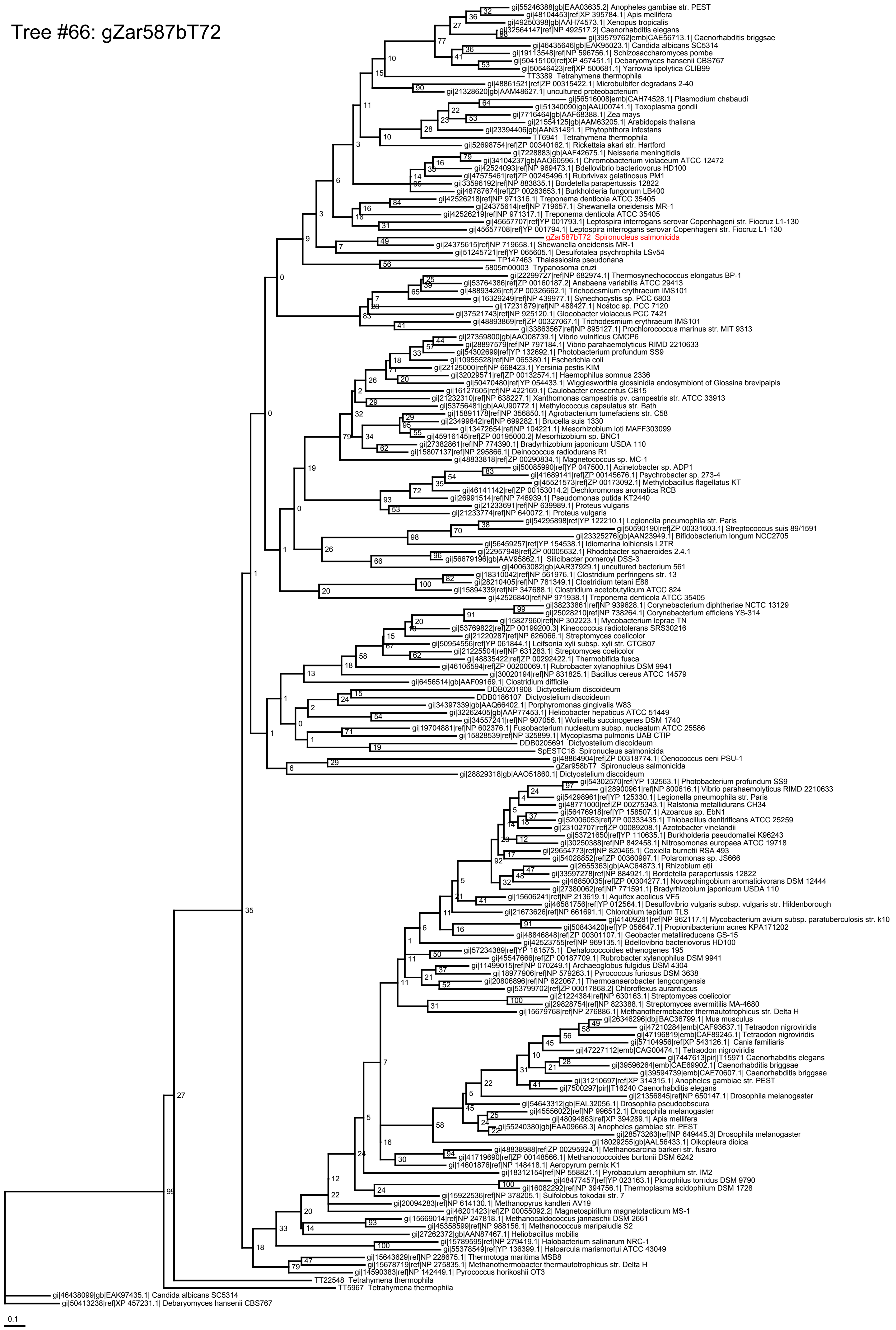

Tree #67: gZar847bT7

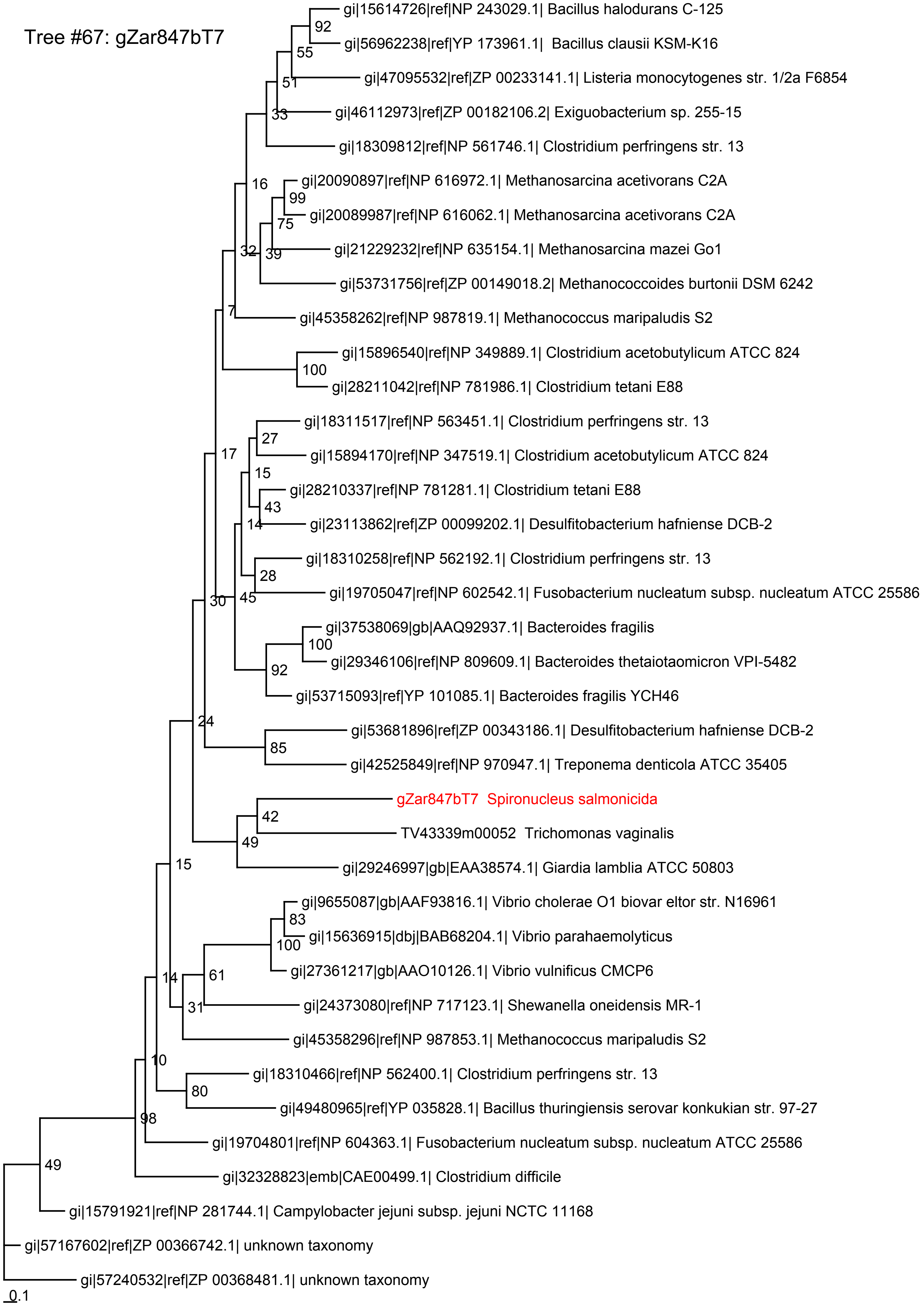

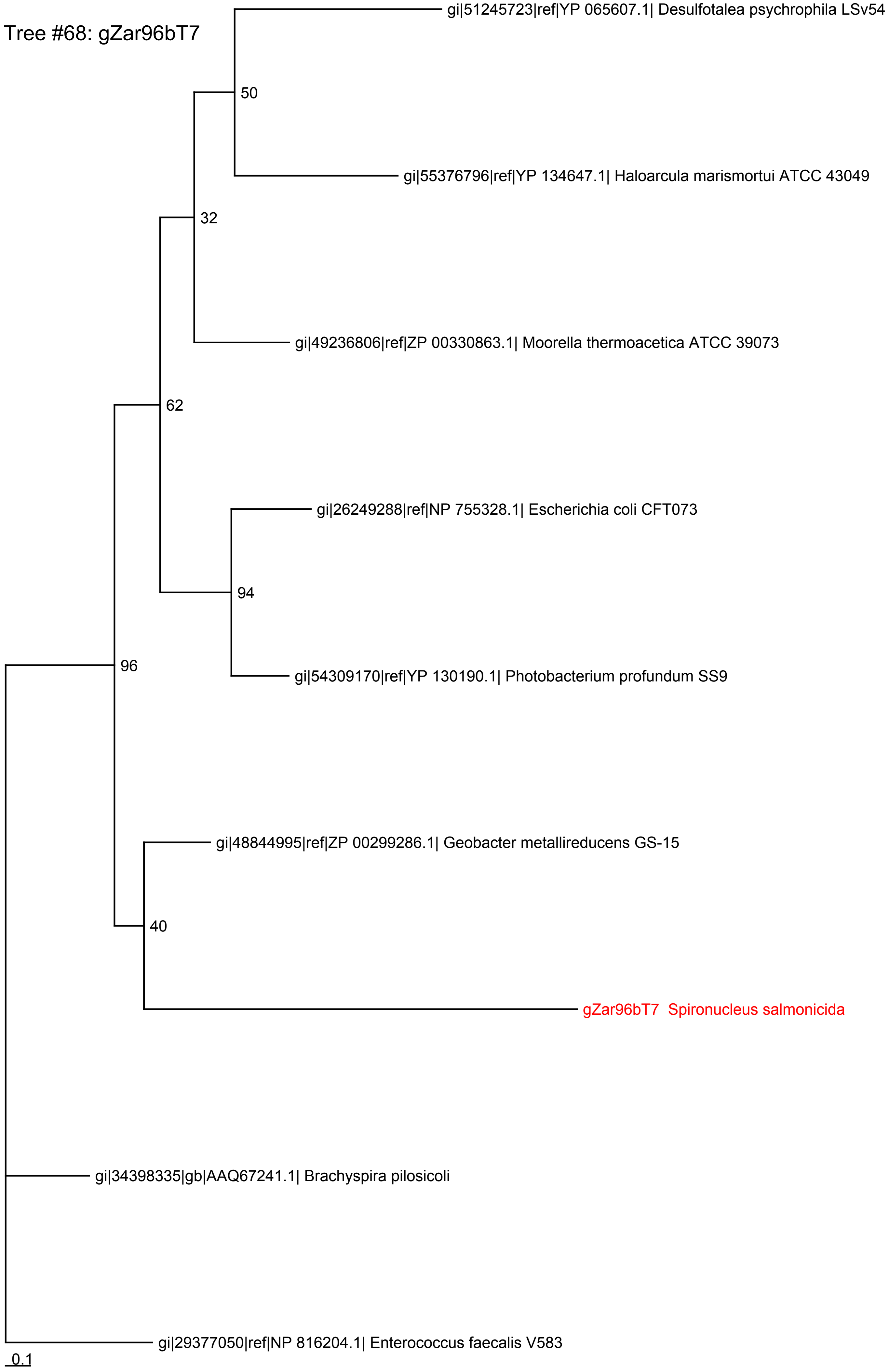
Tree #68: gZar96bT7
gi|51245723|ref|YP 065607.1| Desulfotalea psychrophila LSv54
50
gi|55376796|ref|YP 134647.1| Haloarcula marismortui ATCC 43049
32
gi|49236806|ref|ZP 00330863.1| Moorella thermoacetica ATCC 39073
62
gi|26249288|ref|NP 755328.1| Escherichia coli CFT073
94
96
gi|54309170|ref|YP 130190.1| Photobacterium profundum SS9
gi|48844995|ref|ZP 00299286.1| Geobacter metallireducens GS-15
40
gZar96bT7 Spironucleus salmonicida
gi|34398335|gb|AAQ67241.1| Brachyspira pilosicoli
gi|29377050|ref|NP 816204.1| Enterococcus faecalis V583
0.1

Tree #69: gZar978gT3, gZar789bT7

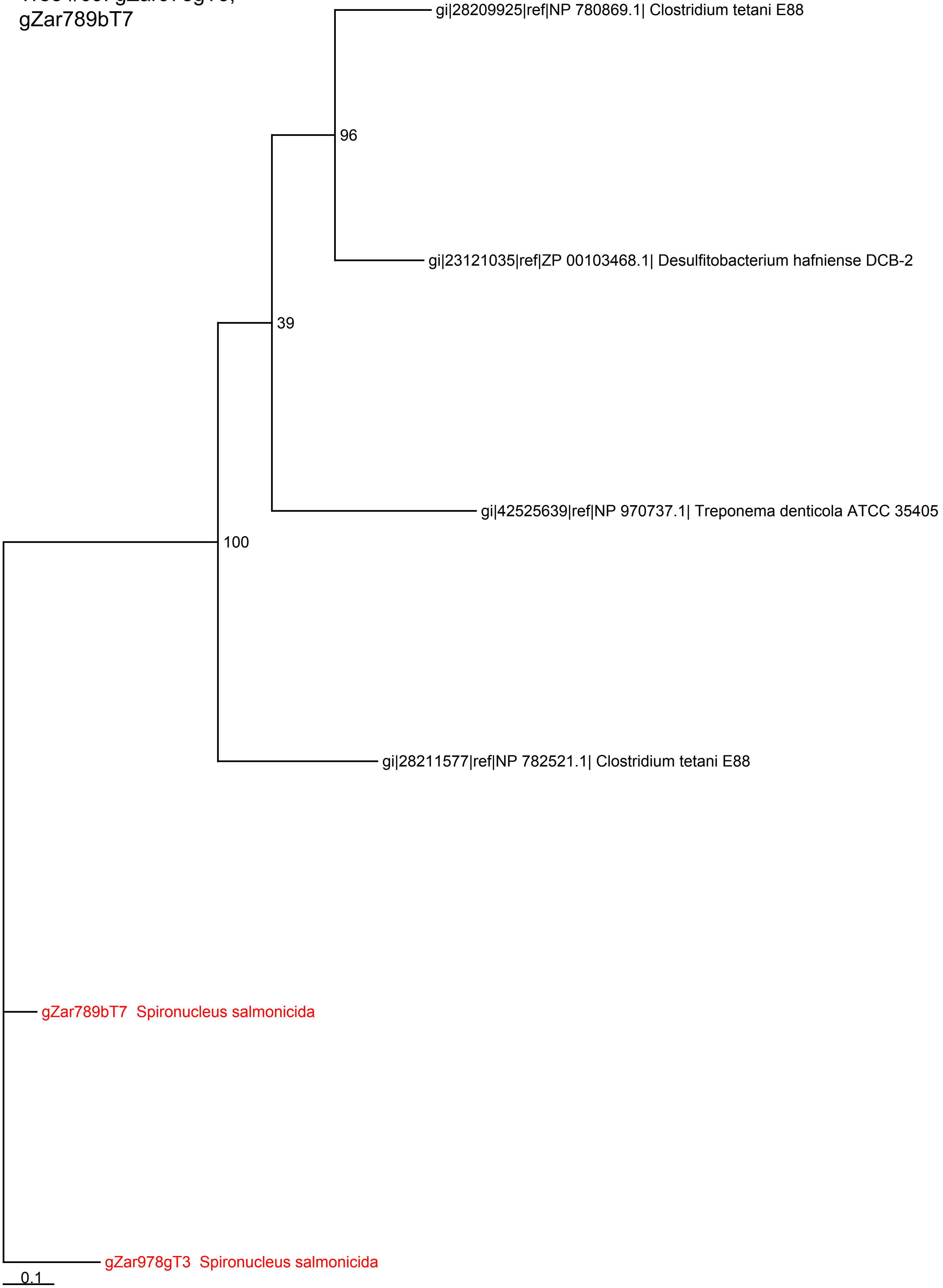

Tree #70: Sp11orf4

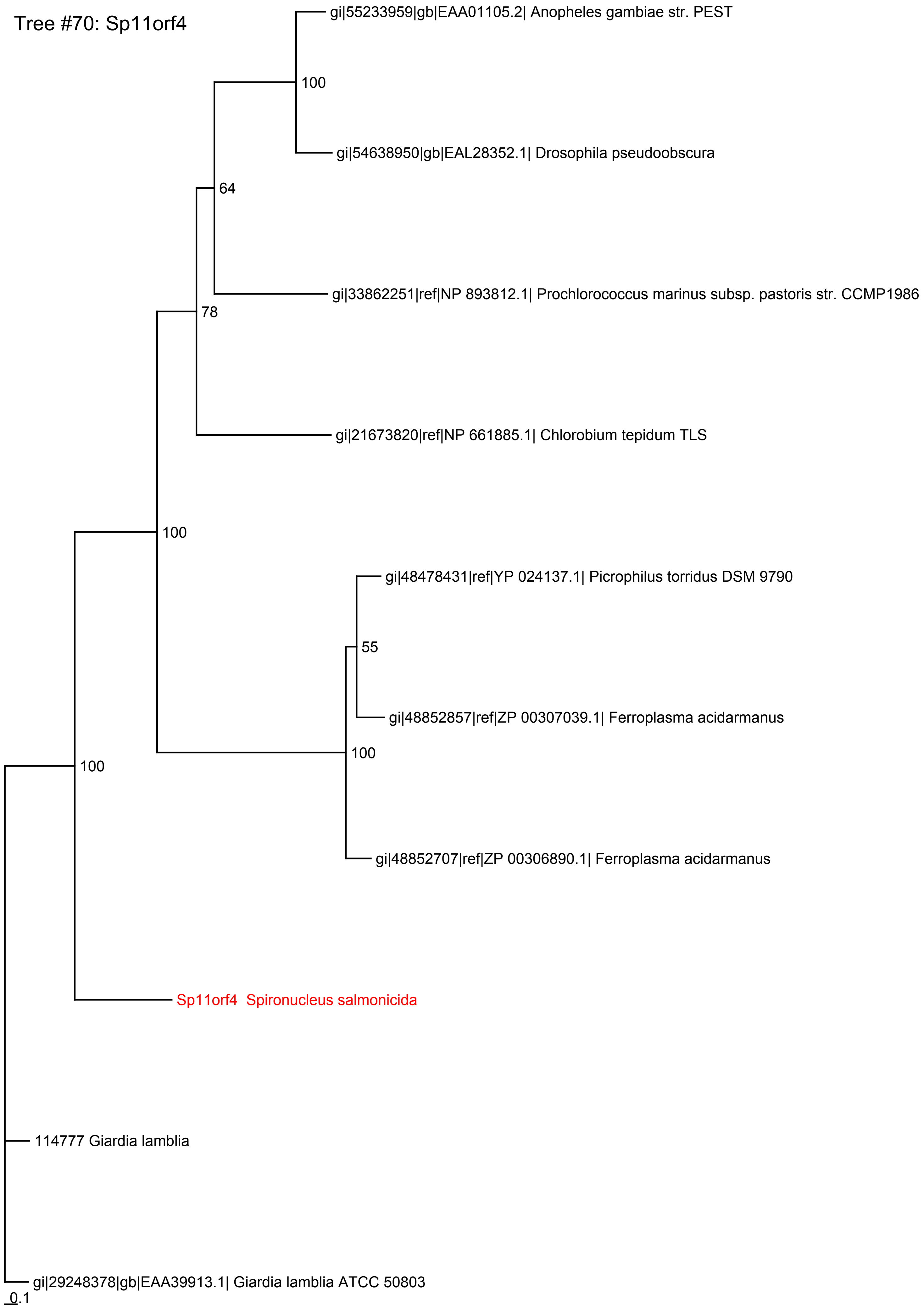

Tree #71: SpESTZap1004

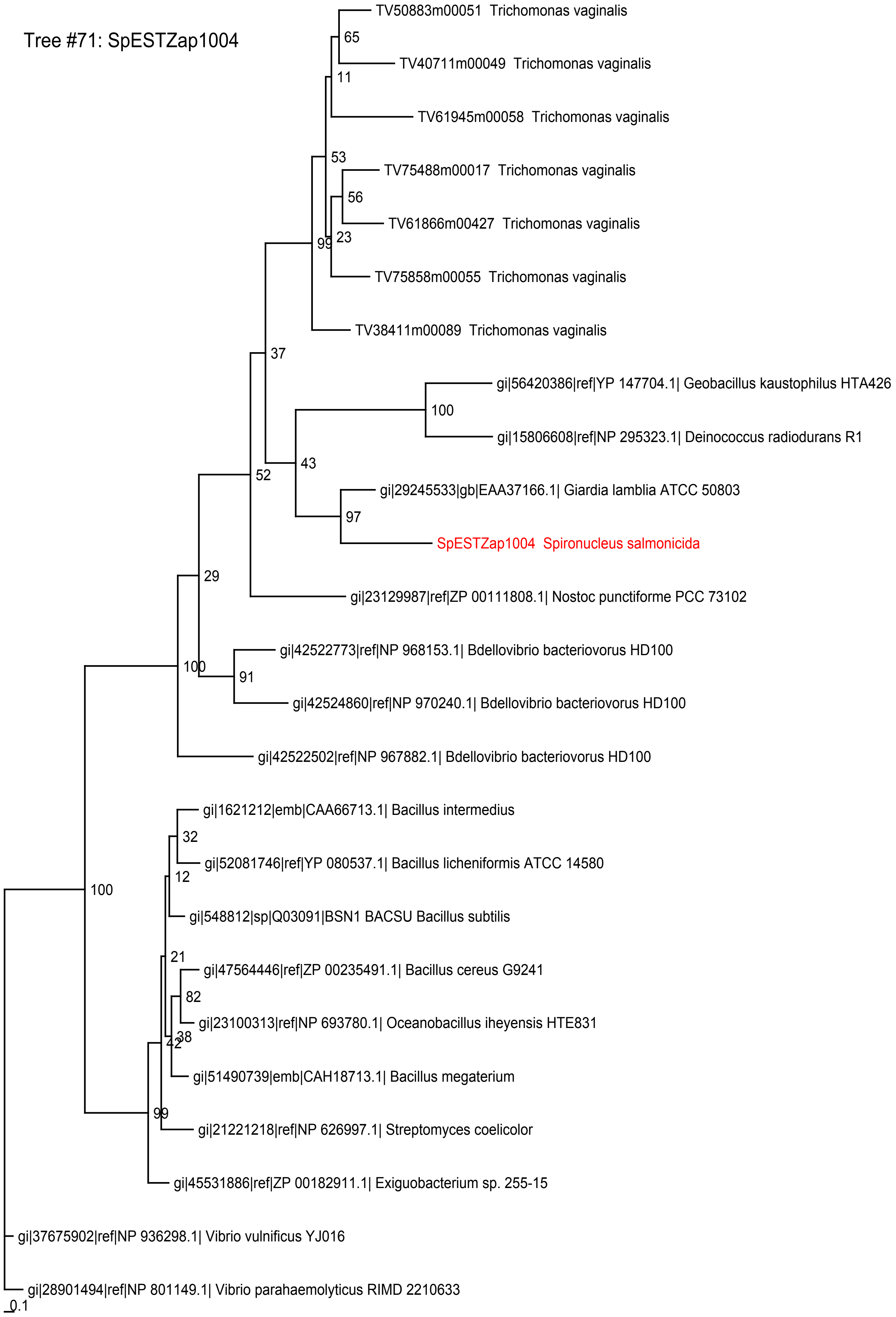

# Tree #72: SpESTZap1248

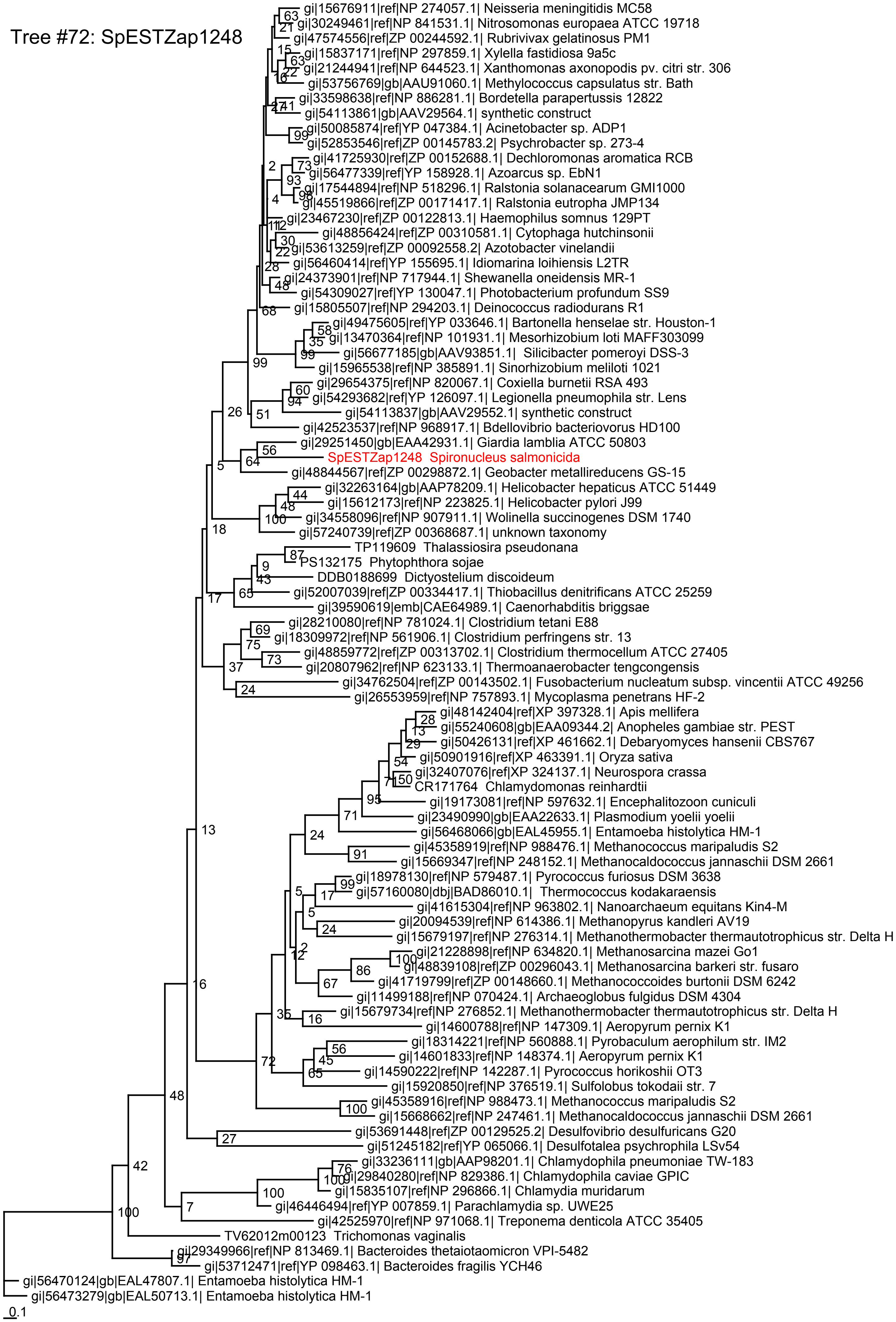

Supplement: Additional file 6 — Phylogenetic trees 51–72 for genes putatively involved in LGT events and listed in Additional file 3. [file 1471-2164-8-51-S6.pdf]
